# Ubiquitous flocculation activity and flocculation production basis of the conglutination mud from *Ruditapes philippinarum* along the coast of China

**DOI:** 10.1371/journal.pone.0256013

**Published:** 2021-11-18

**Authors:** Jun Mu, Yuxia Wang, Xia Cui, Qiao Yang

**Affiliations:** 1 School of Ecology & Environment, Hainan Tropical Ocean University, Sanya, Hainan, China; 2 School of Marine Science & Technology, Zhejiang Ocean University, Zhoushan, Zhejiang, China; Hohai University, CHINA

## Abstract

*Ruditapes philippinarum* conglutination mud (RPM) is a typical waste by-product from manila clam *R*. *philippinarum* aquaculture. However, RPM from the clam at an aquaculture farm in Zhoushan, China, has been newly reported as a promising natural bioflocculant resource that contains effective flocculating polysaccharides from the clam associated bacteria. With an intent to figure out whether RPM flocculation activity is ubiquitous to the manila clam across a wide geographical range or only the Zhoushan location, and to explore the flocculation production basis and ultimately widen its exploitation scope, in this study, an extensive survey of RPMs from four representative locations along the coast of China was performed to determine their flocculation activity, polysaccharide constitution and bacterial community composition. Frozen preserved RPM samples from Zhoushan, Dalian, Weihai and Zhanjiang exhibited comparable flocculation activities (FRs) ranging from 61.9±2.4% to 73.2±0.9% at dosage of 8 g·L^-1^; while fresh RPMs from Zhoushan exhibited a much higher flocculation activity of 91.34±1.18% than its frozen counterpart. Polysaccharide extracts from the four locations showed similar monosaccharide constitutions to some extent. The geographical distribution led to certain variation in bacterial community structures. The similarity clustering of the polysaccharide compositions coincided with that of bacterial community structures from RPMs, suggesting that polysaccharides and respective bacterial communities might be the foundation of the flocculation activity for all RPMs. The overlapping OTUs across all the RPMs accounted for 44.6–62.22% of the overall sequences in each sample and contained the vast majority of the most abundant OTUs (Operational Taxonomic Units), forming a common "core microbiome" that is probably responsible for polysaccharide production and flocculation activity development.

## Introduction

*Ruditapes philippinarum* is one of the most exploited marine bivalves worldwide with a production of approximately 4 million tons annually [1]. China produces the most *R*. *philippinarum* and accounts for approximately 90% of global production [2]. *R*. *philippinarum* is distributed widely along the coast of China and has adapted to various habitats in the Bohai Sea, South China Sea, Yellow Sea and East China Sea. *R*. *philippinarum* conglutination mud (RPM) refers specifically to settled sludge from *R*. *philippinarum* when freshly harvested clams are kept in clean seawater for mud spitting [3]. It is estimated that at least 0.36 million tons of RPM are annually produced from *R*. *philippinarum* aquaculture production in China [1, 2, 4]. Because RPM is mainly a mixture of silt and organic matter from inside the clam bodies, clams need to go through a certain RPM spitting process before entering the market [4]. RPM discarded in certain places may pollute the environment as a kind of solid waste containing organic matter, and it will also cause subsequent treatment and disposal problems when it enters the sewer.

In a long way to explore disposal and utilization approaches for RPM waste, RPM from an aquaculture farm in Zhoushan, China, has recently been found to be a potential bioflocculant resource possessing excellent flocculation activity. In laboratory applications, the RPM can efficiently flocculate kaolin clay in a sea-water assay system, and can also flocculate marine microalgae *Chlorella salina* [3]. Compared with traditional chemical flocculants, the RPM from the Zhoushan clam is nontoxic and environmentally friendly. Compared with the most well-known microbial bioflocculants, the Zhoushan RPM overcomes its high production costs and complicated fermentation/recovery processes, exhibiting easy recovery and fast settlement before and after treating pollutants [3, 5, 6]. The polysaccharides from RPM (Zhoushan) have been extracted successfully by an optimized extraction method through a Box-Behnken design [4]. Two effective flocculating polysaccharides, RPMP-1 and RPMP-2, have been identified [3]. *R*. *philippinarum*-associated bacteria have been shown to be responsible for the flocculation activity of the Zhoushan RPM through selective inhibition of bacteria in a clam farming system and subsequent flocculation activity assay of yielded RPM [7]. Furthermore, Mu et al. have isolated a variety of bacteria that can screen polysaccharide bioflocculants with high similarity of monosaccharide composition to RPMP-1 and RPMP-2, confirming the bacterial origin of the bioflocculation components for the RPM from the Zhoushan location [8]. Based on the aforementioned findings, an RPM substitute has been obtained through a fermentation approach by inoculating fresh RPM from Zhoushan clams in a fermentation system [9]. Nevertheless, to date, only the RPM from the Zhoushan location of China has been comprehensively studied [3, 4, 7–9]. The bioflocculant characteristics and flocculation production bases of RPMs from a broad geographic distribution remain unclear. Therefore, an extensive survey of the flocculation activities and flocculation production bases, which may be common features of *R*. *philippinarum* species, of RPMs from various *R*. *philippinarum* habitats is still of great concern [8].

For the sake of the wide exploitation of RPM as a bioflocculant resource, this study attempted to determine whether RPM flocculation activity is ubiquitous in manila clam across a wide geographical range, and then explored the basis of their potential flocculation activity based on chemical and microbial ecological data. RPMs from four Chinese coastal locations, including the Zhoushan location representing the East China Sea, Dalian location representing the Bohai Sea, Weihai location representing the Yellow Sea and Zhanjiang location representing the South China Sea, were inspected and comparatively analysed for their flocculation activity, polysaccharide constitution and bacterial community structure.

## Materials and methods

### *R*. *philippinarum* sampling and RPM preparation

There were no protected, threatened, or endangered species/locations involved in this study. Four *R*. *philippinarum* sampling locations were distributed across a wide geographical range along the coast of China (S1 Fig). The sampling sites are public. The Dalian sampling location (38°55′ N, 121°26′ E; 23°C) lies along the Bohai Sea, while the Zhanjiang sampling location (21°12′ N, 110°26′ E; 27°C) is situated near the South China Sea; the Weihai sampling location (37°26′ N, 122°10′ E; 29°C) is near the Yellow Sea, and the Zhoushan sampling location (29°56′ N, 122°21′ E; 30°C) is along the East China Sea. For each location, fresh *R*. *philippinarum* clams were collected from the respective aquaculture fields and placed in sterilized seawater to spit the conglutination mud at room temperature (approximately 25°C) for 12–24 h. RPMs outside of Zhoushan city were first transported back to lab under drikold-protection and then stored at -20°C; Zhoushan RPMs were divided into fresh samples and frozen samples (-20 °C). All the frozen RPMs were used for determination of the bioflocculation activity, polysaccharide constitution, and bacterial diversity. Fresh Zhoushan RPM samples were technically available and used to compare bioflocculation activities with frozen samples in order to see if the freezing treatment affects RPM bioflocculation activities. Three replicates were performed for all bacterial diversity analyses.

### Determination of flocculation activity

Flocculation activity is represented by the flocculation rate (FR) and was determined using a published method [3]. Briefly, 93 mL of 4 g L^-1^ kaolin clay suspension, 5 mL of 10 g L^-1^ CaCl_2_ and 2 mL of RPM (2 g L^-1^, 4 g L^-1^, 6 g L^-1^, 8 g L^-1^, 10 g L^-1^, 15 g L^-1^, 20 g L^-1^ of the final concentration) were successively added to a beaker (deionized water was replaced with artificial seawater to prepare the solutions). After the pH was adjusted to 7.5 with 2.0 mol L^-1^ HCl (hydrochloric acid) or 2.0 mol L^-1^ NaOH (sodium hydroxide), the mixture was quickly stirred (200 rpm) for 1 min and slowly stirred (80 rpm) for 2 min, followed by standing for 10 min. The optical density (OD value) of the clarifying supernatant was measured at 550 nm with a spectrophotometer (722 type, DR 1900–05, Hach, Shanghai, China). In the control experiment, 2 mL RPM was replaced with 2 mL artificial seawater. The FR of RPM was calculated by the following equation:

FR(%)=(A-B)/A×100 (1)

where *FR* is the flocculation rate; and A and B are the OD550 (optical density at 550 nm) of the control and sample supernatants, respectively.

### Extraction of crude polysaccharide from RPMs

RPM crude polysaccharides were extracted according to the water extraction method with some modifications [3]. The extraction conditions were as follows: the extraction temperature, 90°C; extraction time, 4.0 h; water-solid ratio, 20 mL g^-1^; stirring speed, 120 r min^-1^; and two extractions. After the RPM crude polysaccharides were extracted into the liquid phase, three volumes of cold alcohol were added and mixed; the mixture was stored at 4°C overnight. The mixture was centrifuged at 8000 ×g for 20 min (Thermo Fisher, USA), and the precipitate was lyophilized to obtain crude polysaccharide.

### Monosaccharide constitution analysis of RPM crude polysaccharides

The monosaccharide components of the crude polysaccharides were analysed by high-performance liquid chromatography (HPLC, Agilent HP 1100, Agilent Technologies, USA) according to the published method with slight modifications [10].

Then, 300 μL crude polysaccharide (3 g L^-1^) was hydrolysed with 300 μL trifluoroacetic acid (TFA, 2 M) in ampoules at 105°C for 6 h. After cooling to room temperature, the ampoules were opened and 600 μL methanol was added and evaporated to dryness on a rotary evaporator to remove the remaining TFA, and the operation was repeated three times. The residue was dissolved in 300 μL NaOH (0.3 mol L^-1^), and then 300 μL 1-phenyl-3-methyl-5-pyrazolone (PMP) methanol solution (0.5 M) was added for derivatization at 70°C for 100 min. The derivatized mixture was cooled to room temperature, and 300 μL HCl (0.3 mol L^-1^) was added to neutralize NaOH. The reaction mixture was extracted by adding an equal volume of chloroform and discarding the chloroform phase; this extraction process was repeated three times. The extracted liquid was filtered through a 0.45 μm microporous membrane for subsequent HPLC sample analysis. The mixed monosaccharide standard sample was subjected to the same hydrolysis and derivatization steps as described above.

An Agilent 1100 HPLC system (USA) with a photodiode array detector was used for chromatographic analysis of the samples and the analysis conditions were as follows: column, Agilent Zorbax Eclipse XDB-C18 (4.6×250 mm, 5 μm, Agilent Technologies, USA); column oven temperature, 30°C; mobile phase, PBS (0.1 M, pH 6.6) with a ratio of 16.2% (v/v %) acetonitrile; flow rate, 0.9 mL min^-1^; injection volume, 10 μL; and detection wavelength, 245 nm.

Monosaccharide standards of D-mannose (Man), L-rhamnose (Rham) and D-glucose (Glc) were purchased from Dr. Ehrenstorfer GmbH (Germany); the other standard monosaccharides of D-ribose (Rib), D-glucuronic acid (GlcUA), D-galacturonic acid (GalUA), D-galactosamine (GalN), D-galactose (Gal), D-xylose (Xyl), D-arabinose (Ara) and L-fucose (Fuc) were obtained from Shanghai Yuanye Biotech. Co. (China). PMP and HPLC-grade acetonitrile were purchased from Tedia Co., USA.

In order to compare polysaccharide similarity of RPMs from four Chinese coastal locations, a heat map was generated showing RPMs polysaccharide compositions clustering. The known pure bioflocculation-active RPMP-1 and RPMP-2 polysaccharides from Zhoushan RPM were also added to the heat map for composition cluster comparison [3].

### DNA extraction, PCR and Illumina MiSeq sequencing

Genomic DNA from 0.5 g RPM samples was extracted using the soil DNA kit (Omega Bio-Tek Inc, USA) by the Cetyltrimethylammonium bromide (CTAB) method [11]. The quality and quantity of total DNA were measured with a NanoDrop spectrophotometer (NanoDrop 2000, USA) and 1% agarose gel electrophoresis. The extracted total DNA was diluted to 1 ng μL^-1^ using the elution buffer and stored at -20°C before further processing. The DNA samples were PCR-amplified using the 343F (5’-TACGGRAGGCAGCAG-3’) and 798R (5’-AGGGTATCTAATCCT-3’) primers [12], which amplifies the V3-V4 regions of the 16S rRNA gene. The PCRs (30 μL final volume) contained 15 μL *Taq* Master Mix (2x) DNA polymerase (Vazyme, Nanjing, China), 1 μL (10 μM) of each primer, 1 μL DNA template (50 ng DNA) and 13 μL H_2_O. The PCR program consisted of an initial denaturation step of 94°C for 5 min followed by 25–35 cycles of denaturation at 94°C for 30 s, annealing at 60°C for 30 s and elongation at 72°C for 30 s. Cycling was completed with a final elongation step of 72°C for 7 min. Equal amounts of purified amplicons were pooled for subsequent sequencing. Amplicons of 16S rRNA were sequenced using MiSeq with PE300 (Illumina Inc., San Diego, CA; OE Biotech Company; Shanghai, China).

### Sequencing data processing

The RPM bacterial diversity was profiled using Illumina MiSeq platform sequencing. The ambiguous bases of the 16S amplicon sequences (20904 ~ 63295 tags) were preprocessed using Trimmomatic software (version 0.38) [13], while the low quality sequences with average quality score below 20 were cut off using the sliding window trimming approach. The trimmed sequences were then assembled using FLASH (Fast Length Adjustment of Short reads) software (version 1.2.11) [14]. And the parameters of assembly were 10 bp of minimal overlapping, 200 bp of maximum overlapping and 20% of maximum mismatch rate. The assembled sequences were performed further denoising using QIIME (Quantitative Insights Into Microbial Ecology) software (version 1.8.0) [15] as follows: reads with ambiguous, homologous sequences or below 200 bp were abandoned; reads with 75% of bases above Q20 were retained; then, reads with chimera were detected and removed. After error filtering, alignment and chimaera removal, the total number of 16S rRNA effective sequences obtained from 12 samples was 439,332, which were clustered into 1716 operational taxonomic units (OTUs) with at least a 97% similarity in terms of nucleotide identity using UPARSE software (version 7.1) [16]. The representative reads from each OTU were selected by the QIIME package [17]. All representative reads were annotated and blasted against Silva database (version 123) using RDP (Ribosomal Database Project, v 2.12) classifier (confidence threshold was 70%) [18], and a phylogenetic tree was constructed using Pynast (version 1.2.2) [15] and Mega (version 7.0). The 16S rRNA gene amplicon sequencing and analysis were conducted by OE Biotech Co., Ltd. (Shanghai, China). The raw sequencing reads of all the samples were submitted to the NCBI (National Center for Biotechnology Information) SRA (Sequence Read Archive) under accession no. PRJNA755854.

### Statistical analyses

The alpha diversity was calculated by QIIME [17], including the Chao1 [19], Shannon Wiener and Gini-Simpson indices [20]. Beta diversity was estimated using both the unweighted UniFrac distance and binary Jaccard distance among the RPMs from the four sampling sites. Principal coordinates analysis (PCoA) plots were generated in the “calibrate” package of R (Version 3.6.2) (https://www.r-project.org/) to visualize the similarities and differences in microbial evolution in different environmental samples [21]. The intra-group differences of the RPMs were determined by Adonis in the “vegan” package of R (Version 3.6.2) [21]. Clustering analysis of the RPM polysaccharide compositions from four Chinese coastal locations was performed using R (Version 3.6.2). Venn analysis was performed using R (Version 3.6.2) to count the number of OTUs shared and unique in multiple samples. One-way analysis of variance (ANOVA) was used to examine significant differences by SPSS Statistics software (SPSS, version 24.0, Chicago, IL, USA), and statistical significance was defined as *p* < 0.05. All data are presented as the mean ± standard deviation (SD).

## Results

### RPM flocculation activity from four coastal locations in China

**Fig 1:**
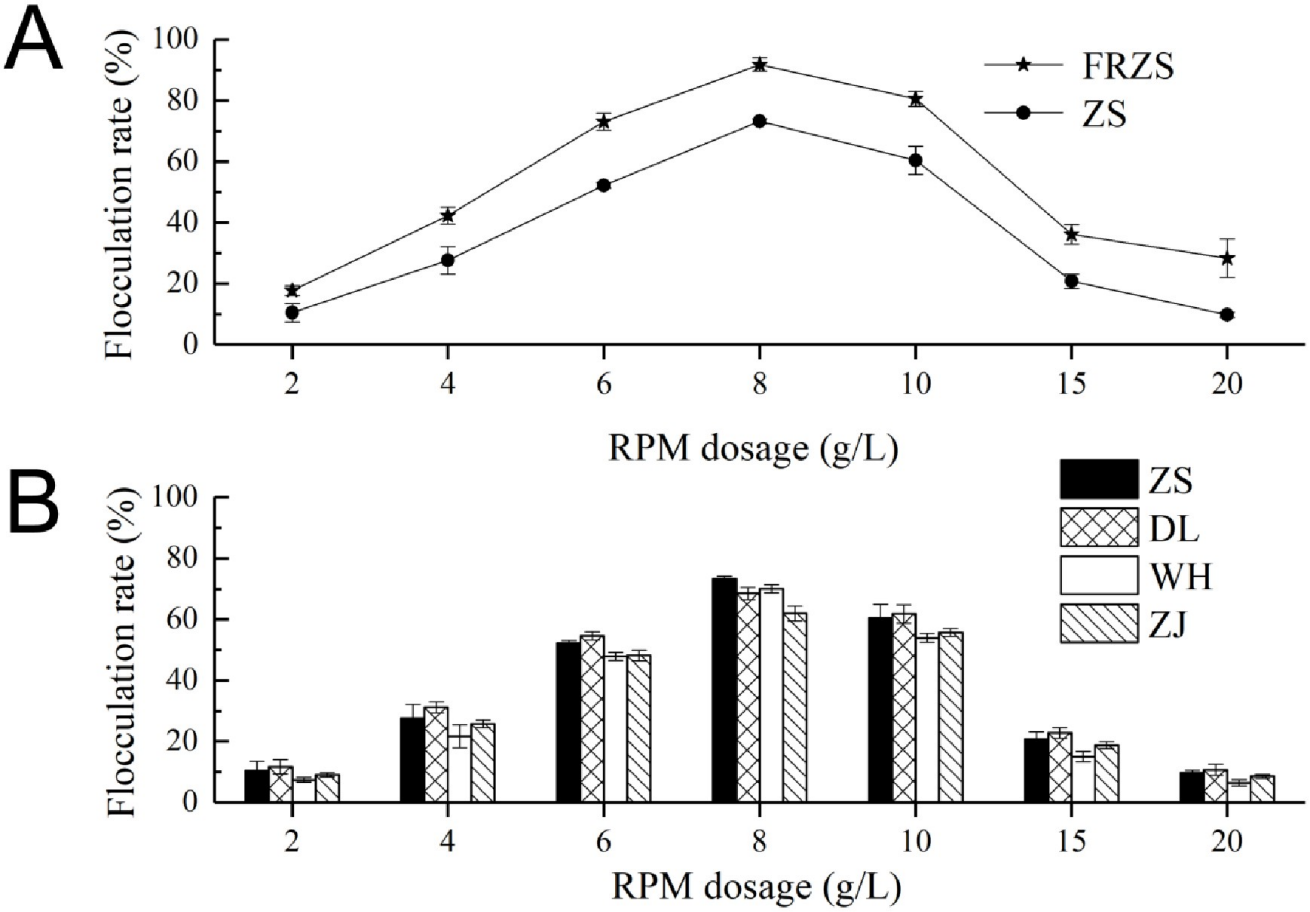
Flocculation activities of the fresh RPM and frozen RPM from Zhoushan (A), and the frozen RPMs from the four coastal locations in China (B). FRZS was fresh RPM from Zhoushan. ZS, DL, WH and ZJ were frozen RPM samples from the Zhoushan, Dalian, Weihai and Zhanjiang locations, respectively. The values are presented as the mean ± SD (n = 3).

For the RPMs from outside of Zhoushan city, a freezing preservation process was applied before flocculation activity determination; local RPMs were divided into fresh samples and frozen samples for flocculation activity comparison. Fig 1A shows that the fresh Zhoushan RPM sample exhibited a higher flocculation activity than the frozen sample and reached an FR value of 91.34±1.18% at an 8 g·L^-1^ dose. From Fig 1B, the FR of the RPMs that received the freezing treatment from all the locations showed a similar changing trend. The comparable maximum FR reached 73.2±0.9% (Zhoushan), 68.5±2.0% (Dalian), 70.0±1.3% (Weihai) and 61.9±2.4% (Zhanjiang) at 8 g·L^-1^ RPM, and declined gradually at lower or higher concentrations of the RPMs.

### The monosaccharide constitution similarity of RPM crude polysaccharides

The RPM crude polysaccharides from four Chinese coastal locations were all heteropolysaccharides composed of Man, Rib, Rham, GlcUA, GalUA, GalN, Glc, Gal, Xyl, Ara and Fuc, and shared similar monosaccharide components but varied in terms of their contents to some extent (Table 1). Gal was the most abundant monosaccharide in DL (41.08%), WH (54.25%) and ZJ (31.36%), while Glc was the dominant monosaccharide in ZS (64.79%). Neutral sugars (including Man, Rib, Rham, Glc, Gal, Xyl, Ara, and Fuc) accounted for a bulk mass proportion of 95.55% for DL, 95.46% for WH, 97.03% for ZS and 92.82% for ZJ. Uronic sugars (GlcUA and GalUA) accounted for 0.97%, 1.64%, 1.62% and 3.66% for DL, WH, ZS and ZJ, respectively (no GalUA in DL). Amino sugars (GalN) were responsible for 3.47%, 2.90%, 1.36% and 3.52% of the DL, WH, ZS and ZJ constituents, respectively.

**Table 1:** Monosaccharide constitutions of RPM crude polysaccharides from four Chinese coastal locations. DL, WH, ZJ and ZS represent for crude polysaccharides from the Dalian, Weihai, Zhanjiang and Zhoushan RPMs respectively.

Monosaccharide constituents	DL		WH		ZS		ZJ	
	Mass ration	Content in%	Mass ration	Content in%	Mass ration	Content in%	Mass ration	Content in%
Man	1.00	7.29	1.00	5.13	1.00	5.37	1.00	7.76
Rib	0.82	5.98	1.23	6.30	0.86	4.60	1.29	10.00
Rham	0.15	1.08	0.17	0.87	0.25	1.34	0.28	2.18
GlcUA	0.13	0.97	0.26	1.34	0.25	1.35	0.37	2.89
GalUA	0	0	0.06	0.30	0.05	0.27	0.10	0.77
GalN	0.48	3.47	0.56	2.90	0.25	1.36	0.45	3.52
Glc	3.25	23.70	2.25	11.56	12.07	64.79	1.48	11.47
Gal	5.64	41.08	10.57	54.25	2.29	12.26	4.04	31.36
Xyl	0.45	3.30	0.67	4.43	0.56	2.99	1.22	9.48
Ara	0.13	0.97	0.16	0.83	0.26	1.38	0.40	3.13
Fuc	1.67	12.15	2.55	13.08	0.80	4.29	2.25	17.44

**Fig 2:**
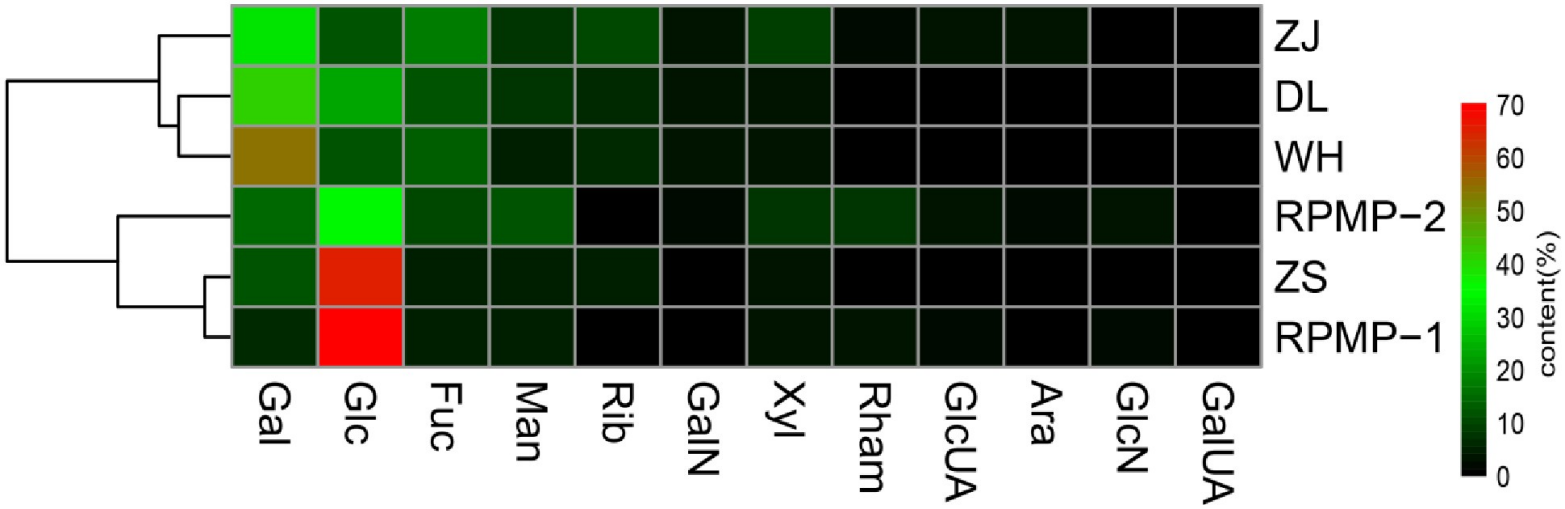
Heat map showing the clustering of the RPM polysaccharide compositions from four Chinese coastal locations.

Fig 2 shows the similarity clustering of the polysaccharide compositions of the RPMs from the four Chinese coastal locations. DL and WH shared the most similar monosaccharide constitutions with each other, and both were similar to ZJ. ZS was positioned in a separate clade and was closest to the known pure bioflocculants RPMP-1 and RPMP-2 from the Zhoushan RPM (Mu et al. 2018). Since ZS was a crude polysaccharide extract, and RPMP-1 and RPMP-2 were pure polysaccharide components isolated from ZS, the common origin reasonably clustered the three of them together.

### Similarity in the overall bacterial community structure of RPM samples across Chinese coastal stations

The taxonomy of all the OTUs is shown in S1 Table. Rarefaction curves confirmed saturation at this depth across the dataset, which meant that a reasonable sequencing depth was attained (S2 Fig). The values of coverage which also suggested that the sequencing depth agreed with this claim.

The observed OTUs, and Chao1 (Chao 1984), Shannon-Wiener, and Gini-Simpson (Jost 2006) indices were used to evaluate the richness and diversity of the bacterial communities in the RPM samples (S2 Table). Chao1 analysis showed that the bacterial richness order was WH > ZS > ZJ > DL (p < 0.05 based on ANOVA). The Shannon-Wiener and Gini-Simpson indices demonstrated that the order of bacterial diversity (species richness and evenness) was WH > ZS > DL > ZJ (p < 0.05 based on ANOVA). Adonis indicated that there was a significant difference among the WH, DL, ZS and ZJ bacterial communities (binary Jaccard: R^2^ = 0.6035, p < 0.001; unweighted UniFrac: R^2^ = 0.6215, p < 0.001).

**Fig 3:**
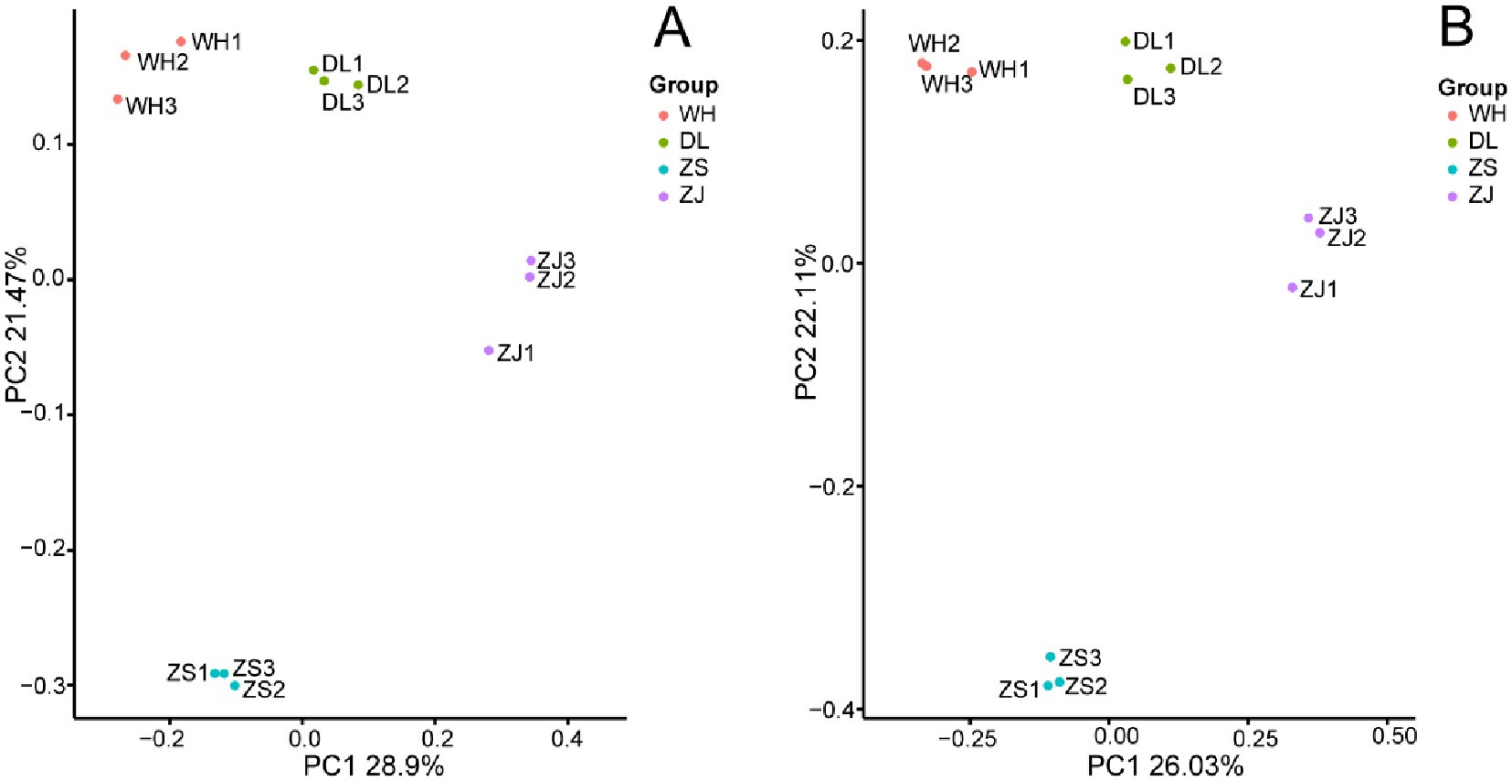
Comparison of the bacterial community structures in samples from different RPMs. Principal coordinates analysis (PCoA) based on the unweighted UniFrac distances (A) and binary Jaccard distances (B). PCoA analysis was performed with the OTUs (at 97% similarity) present in the RPM samples.

The similarity clustering of the bacterial community structures of the RPM groups was presented in Fig 3. From the PCoA based on the unweighted UniFrac distances (Fig 3A) and binary Jaccard distances (Fig 3B), each RPM bacterial community group clustered well and separated clearly from the others. The structure of the WH group was similar to that of the DL group, and both were closer to that of the ZJ group than that of the ZS group. The ZS and ZJ groups exhibited the maximum dissimilarity among the four groups.

### Overlapping OTUs in the bacterial taxonomic distribution of the RPM samples from the Chinese locations

**Fig 4:**
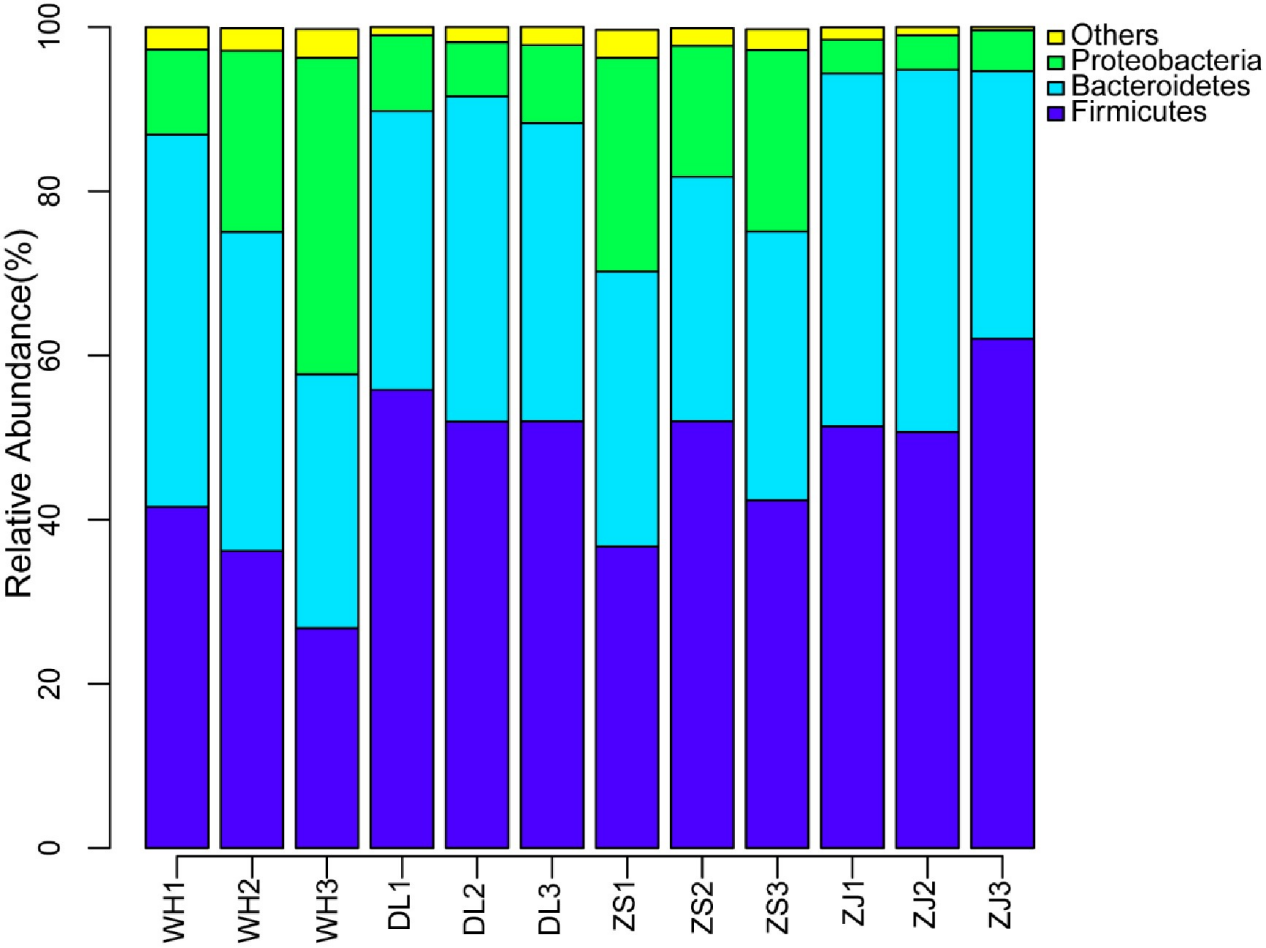
Relative abundance of different bacterial phyla of the RPMs from four coastal locations in China.

The OTU relative abundance at the phylum level was estimated across the different samples. Twenty-six phyla were identified, including some unknown groups. We found that the most dominant phyla in all the samples were Firmicutes, Bacteroidetes and Proteobacteria, composing 96.85% to 99.0% of the mean relative abundance for the four locations (Fig 4). Firmicutes was more abundant in ZJ (54.68%) and DL (53.22%) than in ZS (43.68%) and WH (34.82%). Bacteroidetes was richer in ZJ (39.89%) and WH (38.37%) than in DL (36.65%) and ZS (31.99%). The abundances of Proteobacteria were higher in WH (23.66%) and ZS (21.37%) than in DL (8.44%) and ZJ (4.43%).

**Fig 5:**
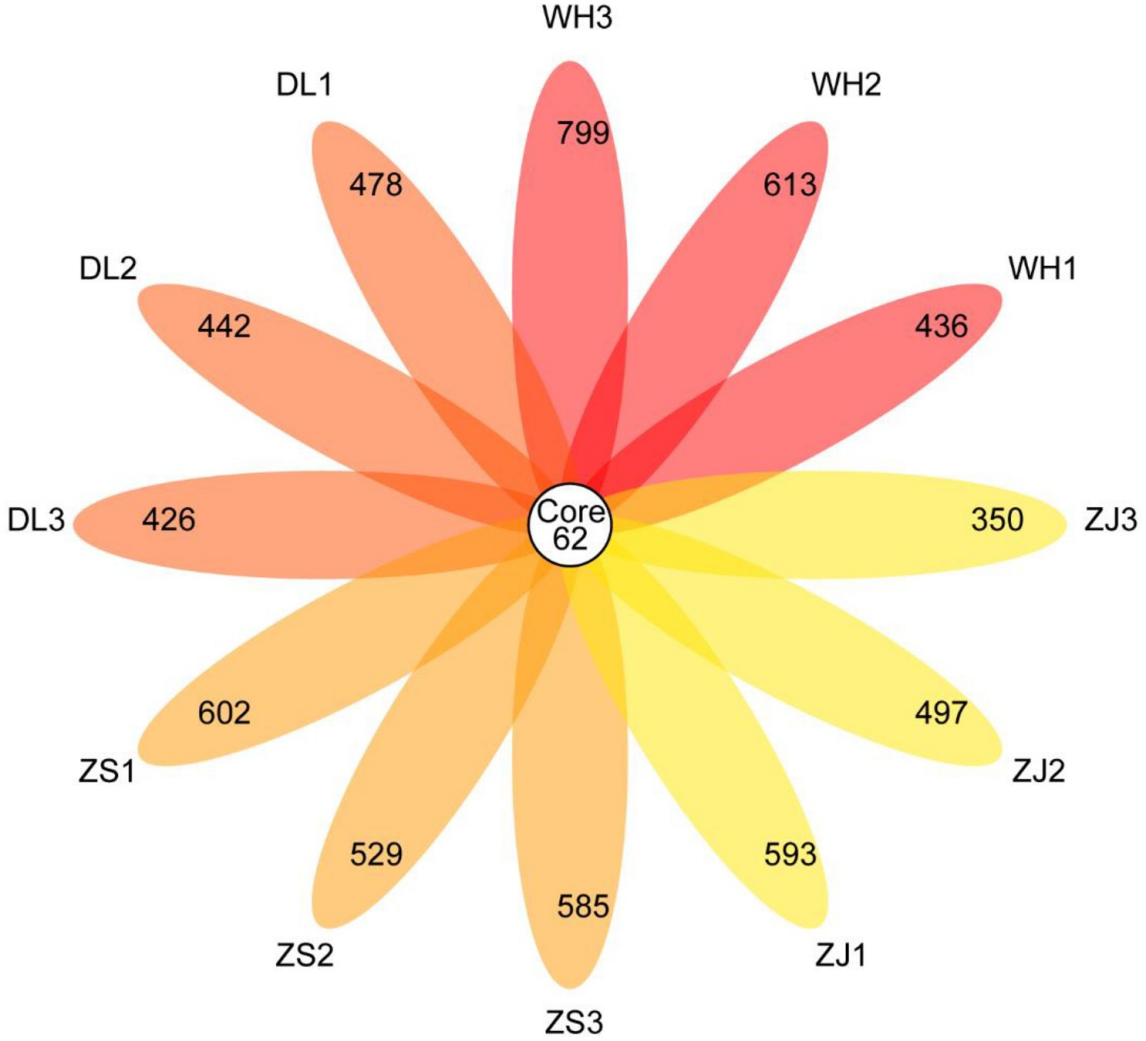
Venn plot showing overlapping OTUs across all the RPM samples.

**Fig 6:**
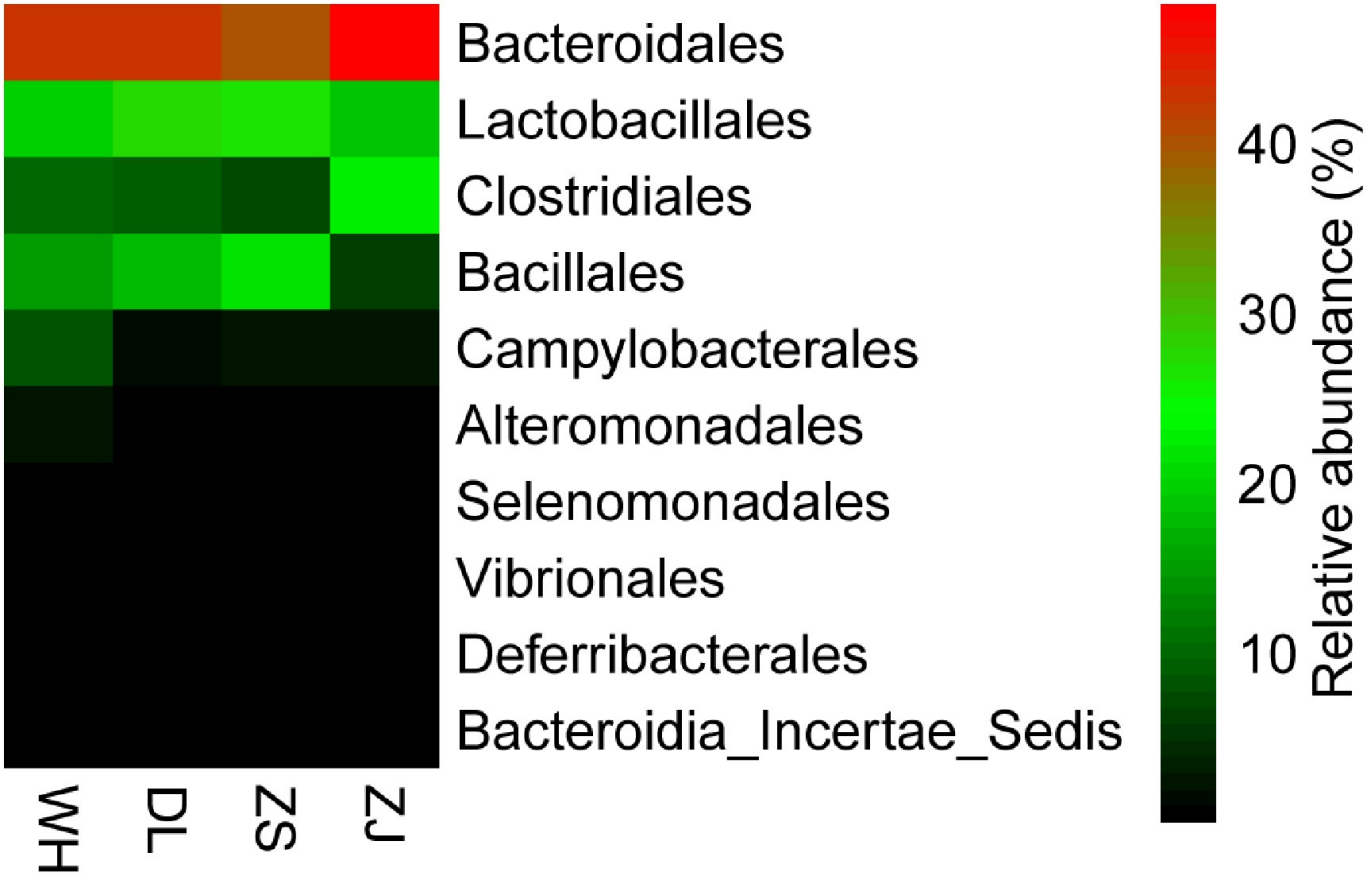
Abundance of the overlapping orders of the RPMs from four coastal locations in China.

Overlapping OTUs were further extracted to analyse the common features of the bacterial communities in the RPMs. There were 62 bacterial OTUs overlapping in all samples, although 3.61% (62/1716) of the total OTU taxa accounted for 44.6–62.22% of the abundance of the respective mean sequences within the four RPM groups (Fig 5). The overlapping order abundance is displayed in Fig 6. The taxonomy of 62 overlapping OTUs is shown in S3 Table. Bacteroidales and Lactobacillales were the top two comparable abundant orders, followed by Bacillales and Clostridiales. Of note, the top 25 most abundant OTUs of all the bacterial communities were in the overlapping portion, and the top 50 most abundant OTUs covered 82% of the overlapping OTUs. The most abundant OTUs (20.59%) were assigned to the *Bacteroidales* family S24_7group_undefined genus, followed by *Prevotella* (14.88%), *Bacillus* (8.89%), *Lachnospiraceae* NK4A136_ group, (7.67%), *Staphylococcus* (6.11%), *Lactobacillus* (5.21%), *Lactococcus* (4.40%), *Alloprevotella* (4.34%) and other genera.

## Discussion

*R*. *philippinarum* conglutination mud was verified to have ubiquitous flocculation activity across a wide geographical range along the coast of China in this study. The scattered *R*. *philippinarum* sampling locations spanned across the China northernmost Bohai near seashore and southernmost South China Sea coast, with a distance of 2244.59 Km (from Dalian to Zhanjiang) as the crow flies. The 17° of latitude difference and 11° of longitude difference lead to vast habitat and environmental changes in the four representative sampling locations, regardless of the local temperatures, aquaculture water quality and seasonal variation. The unchanging characteristics were their comparable flocculation activities. The fresh RPM from the Zhoushan location had higher flocculation activities than the frozen RPM, demonstrating that the physical condition of RPM may be one of the key factors that influence RPM flocculation activity, since the freezing-treatment probably destroyed RPM floc structures to different degrees leading to a lower performance of flocculation activity. It has been reported that RPM from an aquaculture farm in Zhoushan of China is a promising natural bioflocculant resource [3]. The naturally collected RPM exhibits high flocculation activities to kaolin clay in both the deionized water assay system and the seawater assay system, and can effectively flocculate the marine microalgae *Chlorella salina*. The findings from this study could help widen the scope of potential RPM exploitation to the entire coast of China.

Polysaccharides are most likely the foundation of RPM flocculation activity. To date, an increasing number of polysaccharides have been found to be the main flocculation components within the majority of bioflocculants [22–24]. A previous study showed that polysaccharides from the Zhoushan RPM have been suggested to be responsible for RPM flocculation, with RPMP-1 and RPMP-2 as the main bioflocculation components [3]. In the present study, crude RPM polysaccharides from four locations were prepared and compared on a monosaccharide constitution analysis basis. The polysaccharide extracts from the four locations showed similar monosaccharide constitutions to some extent. Similar monosaccharide components are common and specific characteristics attributed to RPMs, and as far as we know, no polysaccharide bioflocculants with similar monosaccharide components have been reported other than RPMs [22–24]. Similar polysaccharides reasonably have the same activity.

The bacteria in RPM are mostly responsible for flocculation activity. The similar polysaccharides from the RPMs for the four locations suggested similar reproductive origins. *R*. *philippinarum*-associated bacteria have been found to be the origin of the flocculation components in the Zhoushan RPM [3, 7–9]. Therefore, similar bacterial community structures, especially common bacterial species as overlapping OTUs across all the RPMs, became the first search target. For the bacterial community, the "core microbiome" and community activity have been discussed in several references [25, 26]. A core microbiome is typically defined as the suite of members shared among microbial consortia from similar habitats, and is represented by the overlapping areas of circles in Venn diagrams [26]. In the present study, the overlapping OTUs accounted for 44.6–62.22% of the abundance of all the RPM groups and significantly converged the vast majority of the most abundant OTUs, which could be taken as a "core microbiome", most likely providing the common flocculation activity and polysaccharide traits for all the RPMs. Among the most abundant OTUs corresponding to taxa in the "core microbiome", *Bacteroidales* family *S24_7*_group (20.59%) and *Lachnospiraceae NK4A136*_group (7.67%) belong to uncultured species and their capacity for extracellular polysaccharide production is currently unknown. However, many EPF-producing microorganisms (EPF, extracellular polysaccharide flocculants) have been reported to belong to *Bacillus* (8.89%) and *Staphylococcus* (6.11%) [22, 24, 27]. *Prevotella* (14.88%), *Lactobacillus* (5.21%) and *Lactococcus* (4.40%), all of which are host biofilm- producing species [28–30]. Although it is hard to deduce that these genera of OTUs produced flocculation-active polysaccharides since some physiological characteristics are present under strain basis and subjected to environmental conditions, analysis of an unknown community at a higher taxa level could still present some rough information on the potential for flocculation-active polysaccharide production.

The geographical separation of the locations caused variations in both the RPM bacterial community structures and RPM polysaccharide constitutions. Remarkably, the similarity clustering of the RPM bacterial community structures from the four locations coincided with the clustering of the RPM polysaccharide constitution from the corresponding sites. The DL polysaccharide constitution was most similar to the WH polysaccharide constitution, and the community structures of the DL group appeared to be the same as that of the WH group; DL and WH were closer to ZJ than to ZS, as were the community structures of DL and WH to ZJ and ZS; ZS was positioned in a separate clade, and likewise, the community structures of ZS group exhibited the maximum dissimilarity to that of the other three groups. Since the same portion of the bacterial community may produce the same secondary metabolites, while different parts of the bacterial community may cause variation, the similarity clustering correlation for the RPM bacterial community structures with the RPM polysaccharide constitutions further indicated that the common "core microbiome" was probably responsible for the polysaccharide production and flocculation activity development of all the RPMs.

The ubiquitous flocculation activity and similar flocculation production basis of RPM might shed light on the ecological effect of the behavior of *R*. *philippinarum*. The manila clam *R*. *philippinarum* is mostly conditioned to burrow in sandy soil with two siphons stretching out, one for taking in food and the other for expelling waste, such as RPM. The filter-feeding mode of the clam may cause significant bioturbation to its surroundings [31–33]. This bioturbation plays an important role in the biogeochemical processes of marine and lacustrine environments, which can resuspend benthic sediments and promote vertical downward movement of surface sediment [32, 34, 35]. Accompanying bioturbation, it has been observed that *R*. *philippinarum* can purify seawater quality through its filtering effect [31], and further research has shown that bioturbation itself leads to water purification [32]. Our research clearly explained the water purification mechanism by which *R*. *philippinarum* expels RPM and effectively flocculates suspended particles as well as other pollutants. Thus, the highly efficient flocculation activity of RPM has endowed bioturbation with the characteristic of the speeded biological sedimentation. Undoubtedly, the *R*. *philippinarum*-associated "core microbiome" and correspondingly produced extracellular polysaccharides with flocculation activity make the ecological effect of *R*. *philippinarum* behavior possible. In addition, other filter-feeding shellfish with siphons tend to produce pseudofaeces somewhat similar to RPM. Whether pseudofaeces perform a comparable function and use a similar mechanism are of interest.

## Conclusion

RPMs from four representative locations (Zhoushan, Dalian, Weihai and Zhanjiang) all exhibited comparable flocculation activities, demonstrating that RPM bioflocculation function is ubiquitous from *R*. *philippinarum* aquaculture along the coast of China. This finding will considerably widen the scope of RPM exploitation and its potential application. A "core microbiome" existed in RPM bacterial communities, and this "core microbiome" is probably responsible for polysaccharide production and flocculation activity development.

## Supporting information

S1 Fig The *R*. *philippinarum* sampling locations along China coasts. Red circles showed four locations of Dalian, Weihai, Zhoushan and Zhanjiang, scattering near the seasides of Bohai Sea, Yellow Sea, East China Sea and South China Sea respectively.
(PDF)

S2 Fig Rarefaction curve of the MiSeq 16S rDNA sequencing of the RPM samples. (PDF)

S1 Table The taxonomical classification and abundance of all OTUs from RPMs of four Chinese coastal locations. (PDF)

S2 Table Richness and diversity estimation of the 16S rRNA sequencing libraries from the MiSeq sequencing analysis. WH, DL, ZS and ZJ represent bacterial communities from the Weihai, Dalian, Zhoushan and Zhanjiang RPMs respectively. The values are presented as mean ± SD (n = 3).
(PDF)

S3 Table The taxonomical classification and abundance of all overlapping OTUs from RPMs of four Chinese coastal locations. (PDF)
