# Supplementary material for: Ubiquitous flocculation activity and flocculation production basis of the conglutination mud from Ruditapes philippinarum along the coast of China
Source: PLoS One. 2021 Nov 18;16(11):e0256013. doi: 10.1371/journal.pone.0256013 (PMC8601509; doi:10.1371/journal.pone.0256013)

S1 Fig. The *R. philippinarum* sampling locations along China coasts. Red circles showed four locations of Dalian, Weihai, Zhoushan and Zhanjiang, scattering near the seasides of Bohai Sea, Yellow Sea, East China Sea and South China Sea respectively.

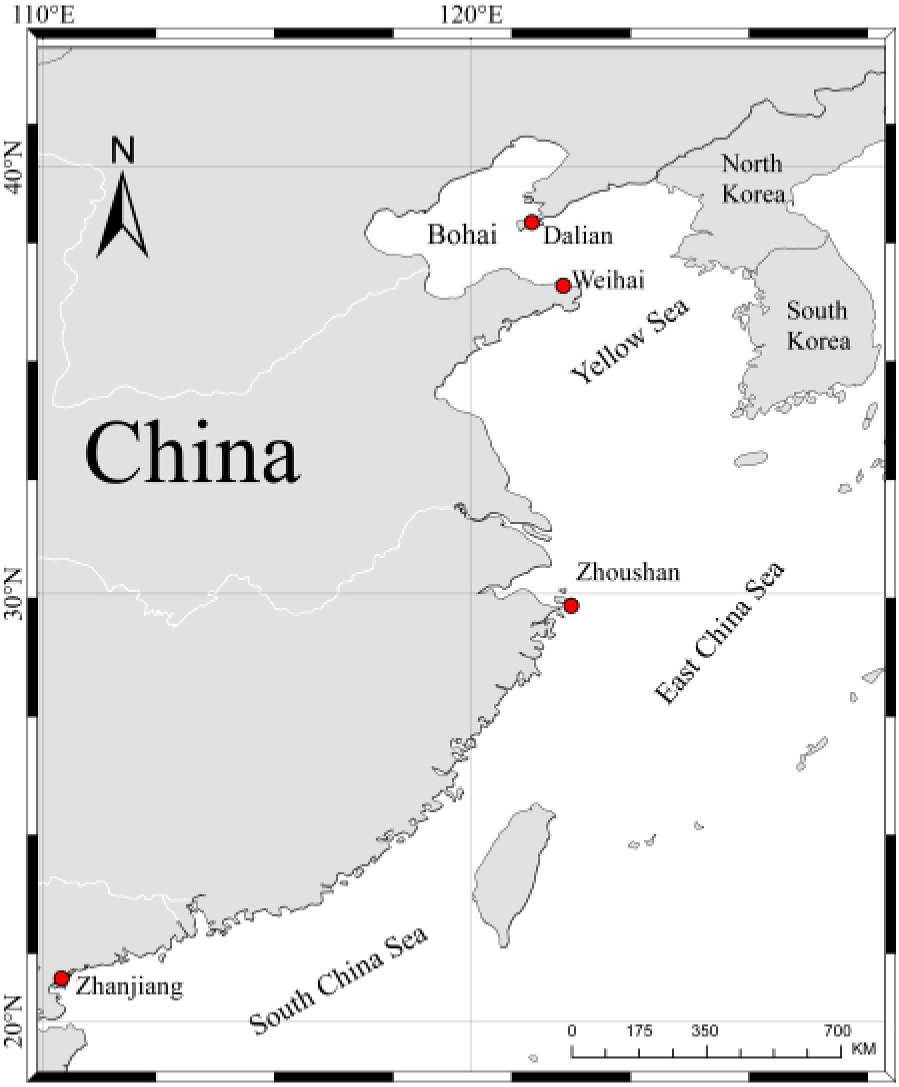

Supplement: S1 Fig — Red circles showed four locations of Dalian, Weihai, Zhoushan and Zhanjiang, scattering near the seasides of Bohai Sea, Yellow Sea, East China Sea and South China Sea respectively. (PDF) [file pone.0256013.s001.pdf]
