# Supplementary figures and images for: Ubiquitous flocculation activity and flocculation production basis of the conglutination mud from Ruditapes philippinarum along the coast of China
Source: PLoS One. 2021 Nov 18;16(11):e0256013. doi: 10.1371/journal.pone.0256013 (PMC8601509; doi:10.1371/journal.pone.0256013)

**S2 Fig. Rarefaction curve of the MiSeq 16S rDNA sequencing of the RPM samples.**

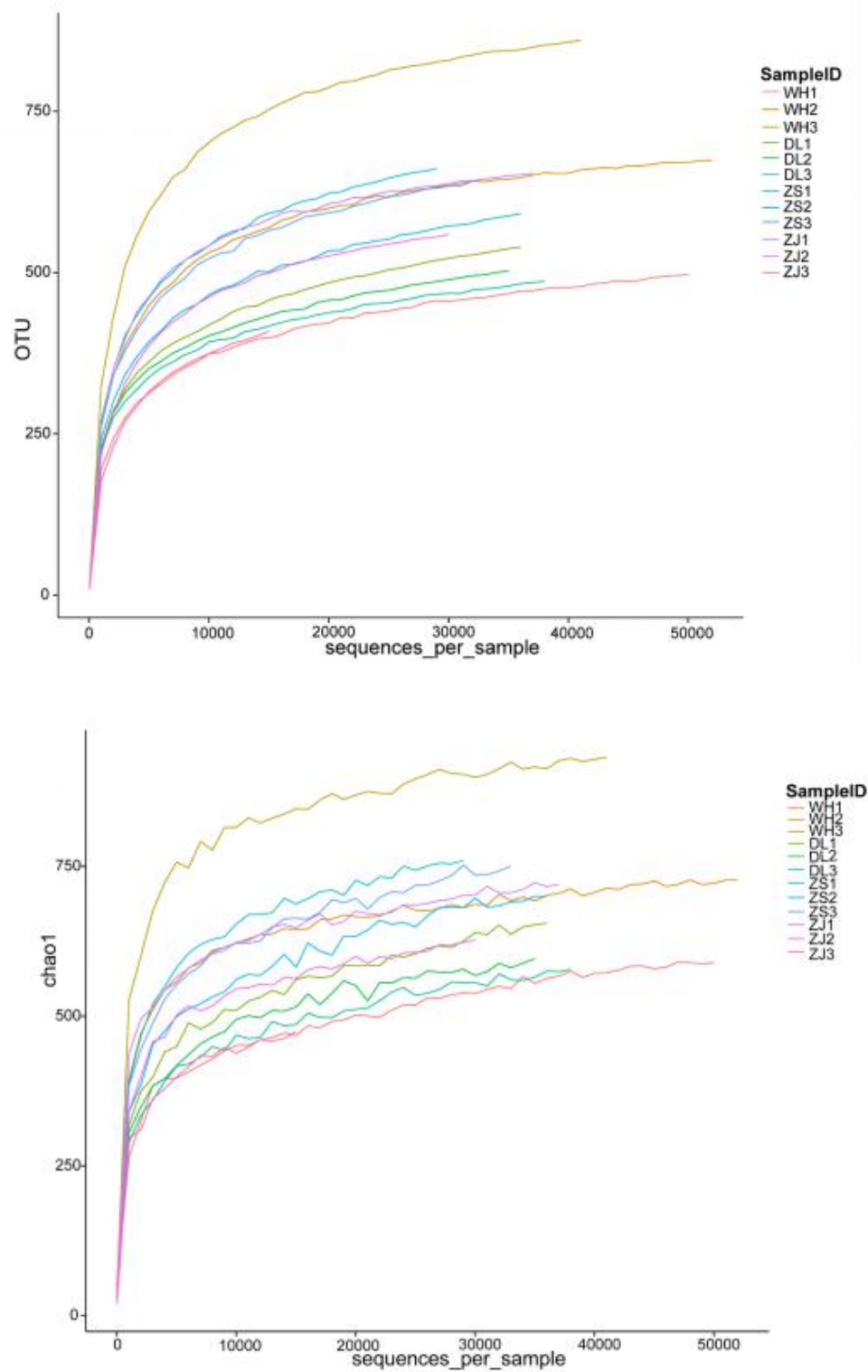

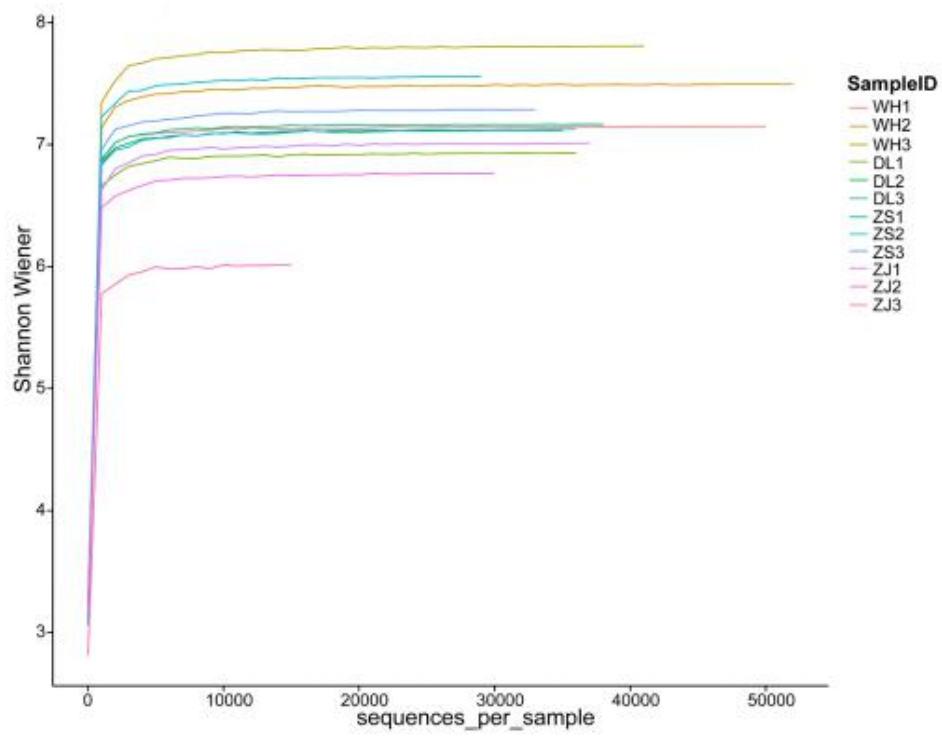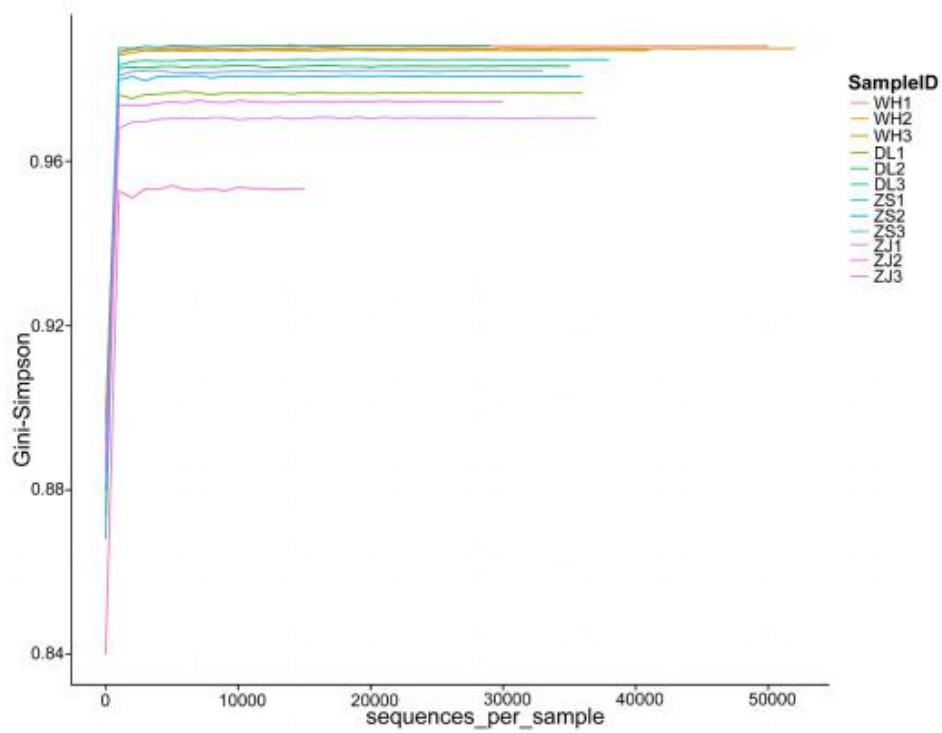

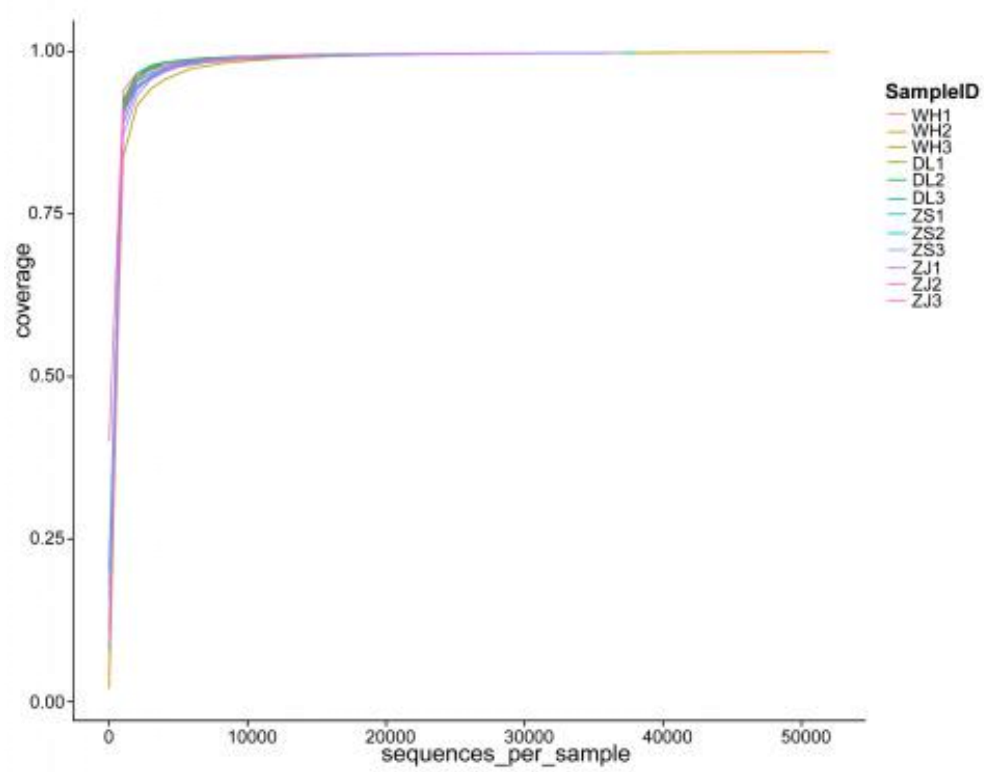

Supplement: S2 Fig — (PDF) [file pone.0256013.s002.pdf]
