# Supplementary material for: Ubiquitous flocculation activity and flocculation production basis of the conglutination mud from Ruditapes philippinarum along the coast of China
Source: PLoS One. 2021 Nov 18;16(11):e0256013. doi: 10.1371/journal.pone.0256013 (PMC8601509; doi:10.1371/journal.pone.0256013)
Supplement: S1 Table — (PDF) [file pone.0256013.s003.pdf]

S1 Table The taxonomical classification and abundance of all OTUs from RPMs of four Chinese coastal locations

OTU_ID	WH1	WH2	WH3	DL1	DL2	DL3	ZS1	ZS2	ZS3	ZJ1	ZJ2	ZJ3	taxonomy
880	7	5	10	0	1	0	2	0	5	0	0	0	k__Bacteria; p__Proteobacteria; c__Deltaproteobacteria; o__Desulfobacterales; f__Desulfobulbaceae; g__Desulfobulbus
470	21	2	32	3	3	0	0	0	1	0	0	0	k__Bacteria; p__Proteobacteria; c__Gammaproteobacteria; o__Chromatiales; f__Ectothiorhodospiraceae; g__Thiogranum; s__uncultured_gamma_proteobacterium
881	1	105	0	0	0	0	0	0	0	0	0	0	k__Bacteria; p__Actinobacteria; c__Coriobacteriia; o__Coriobacteriales; f__Coriobacteriaceae; g__Coriobacteriaceae_UCG_002; s__uncultured_bacterium
840	11	11	39	0	0	0	1	0	0	0	0	0	k__Bacteria; p__Actinobacteria; c__Acidimicrobiia; o__Acidimicrobiales; f__OM1_clade; Ambiguous_taxa; Ambiguous_taxa
471	0	0	0	0	29	0	0	0	0	11	0	0	k__Bacteria; p__Firmicutes; c__Clostridia; o__Clostridiales; f__Ruminococcaceae; g__Oscillibacter; s__uncultured_bacterium
430	0	0	0	23	0	0	0	0	0	0	2	5	k__Bacteria; p__Firmicutes; c__Clostridia; o__Clostridiales; f__Ruminococcaceae; g__Oscillibacter; s__uncultured_bacterium
882	0	3	0	0	0	0	0	0	0	0	0	0	k__Bacteria; p__Proteobacteria; c__Gammaproteobacteria; o__Xanthomonadales; f__JTB255_marine_benthic_group
841	10	4	19	3	0	0	0	0	0	0	0	0	k__Bacteria; p__Proteobacteria; c__Deltaproteobacteria; o__Desulfobacterales; f__Desulfobulbaceae; g__SEEP_SRB4; s__uncultured_delta_proteobacterium

800	11	39	24	0	3	0	0	2	0	0	0	0	k__Bacteria; p__Bacteroidetes; c__Bacteroidetes_VC2.1_Bac22; Ambiguous_taxa; Ambiguous_taxa; Ambiguous_taxa; Ambiguous_taxa
472	1	0	4	0	3	1	1	0	4	0	0	0	k__Bacteria; p__Bacteroidetes; c__Sphingobacteriia; o__Sphingobacteriales; f__Saprospiraceae; g__Portibacter; Ambiguous_taxa
431	0	0	0	3	0	1	0	0	0	0	0	0	k__Bacteria; p__Bacteroidetes; c__Bacteroidia; o__Bacteroidales; f__Marinilabiaceae; g__Marinifilum
883	0	7	0	0	0	0	2	0	1	1	0	0	k__Bacteria; p__Bacteroidetes; c__Bacteroidia; o__Bacteroidales; f__Marinilabiaceae; g__uncultured; Ambiguous_taxa
842	8	2	19	0	0	0	9	7	1	0	0	0	k__Bacteria; p__Proteobacteria; c__Gammaproteobacteria; o__Cellvibrionales; f__Halieaceae
801	15	0	0	0	0	0	0	0	0	0	0	0	k__Bacteria; p__Acidobacteria; c__Subgroup_22; o__uncultured_bacterium; f__uncultured_bacterium; g__uncultured_bacterium; s__uncultured_bacterium
473	0	0	2	0	33	0	0	0	0	0	10	3	k__Bacteria; p__Actinobacteria; c__Coriobacteriia; o__Coriobacteriales; f__Coriobacteriaceae; g__Enterorhabdus; Ambiguous_taxa
432	0	0	0	29	0	0	0	0	0	6	11	0	k__Bacteria; p__Actinobacteria; c__Coriobacteriia; o__Coriobacteriales; f__Coriobacteriaceae; g__Enterorhabdus; s__uncultured_bacterium
884	0	114	0	0	0	0	0	0	0	0	13	3	k__Bacteria; p__Firmicutes; c__Clostridia; o__Clostridiales; f__Clostridiales_vadinBB60_group
843	10	4	1	2	0	0	0	0	12	19	9	3	k__Bacteria; p__Firmicutes; c__Clostridia; o__Clostridiales; f__Family_XII; g__Fusibacter

802	4	1	4	0	0	0	133	121	133	1	0	0	k__Bacteria; p__Proteobacteria; c__Gammaproteobacteria; o__Alteromonadales; f__Pseudoalteromonadaceae; g__Psychrosphaera
474	0	0	0	0	38	1	0	0	0	9	7	0	k__Bacteria; p__Firmicutes; c__Clostridia; o__Clostridiales; f__Lachnospiraceae; g__uncultured; s__unidentified
433	0	0	0	25	0	0	0	0	0	0	0	0	k__Bacteria; p__Proteobacteria; c__Deltaproteobacteria; o__Desulfuromonadales; f__GR_WP33_58; g__uncultured_rumen_bacterium; s__uncultured_rumen_bacterium
885	0	150	0	0	0	0	0	0	0	5	0	2	k__Bacteria; p__Firmicutes; c__Clostridia; o__Clostridiales; f__Lachnospiraceae; g__uncultured
844	15	17	17	0	0	0	4	15	2	0	0	0	k__Bacteria; p__Proteobacteria; c__Alphaproteobacteria; o__Rhizobiales; f__Phyllobacteriaceae; g__Hoeflea; Ambiguous_taxa
803	4	0	9	0	0	0	29	18	4	0	0	0	k__Bacteria; p__Proteobacteria; c__Gammaproteobacteria; o__Oceanospirillales; f__Oceanospirillaceae
475	0	0	0	0	42	1	0	0	0	0	0	0	k__Bacteria; p__Firmicutes; c__Clostridia; o__Clostridiales; f__Lachnospiraceae; g__uncultured
434	0	0	0	16	5	5	0	0	0	0	0	0	k__Bacteria; p__Proteobacteria; c__Deltaproteobacteria; o__Desulfobacterales; f__Desulfobulbaceae
886	0	3	0	0	0	0	0	0	0	0	0	0	k__Bacteria; p__Bacteroidetes; c__Flavobacteriia; o__Flavobacteriales; f__Cryomorphaceae; g__Fluviicola
845	1	0	1	0	0	0	0	0	0	0	0	0	k__Bacteria; p__Bacteroidetes; c__Flavobacteriia; o__Flavobacteriales; f__Flavobacteriaceae; g__Gramella; Ambiguous_taxa

804	9	12	4	0	1	1	0	0	0	0	0	0	k__Bacteria; p__Bacteroidetes; c__Flavobacteriia; o__Flavobacteriales; f__Flavobacteriaceae; g__Winogradskyella; Ambiguous_taxa
476	0	0	0	0	43	1	1	2	2	315	7	11	k__Bacteria; p__Firmicutes; c__Clostridia; o__Clostridiales; f__Clostridiaceae_1; g__Clostridium_sensu_stricto_1; s__uncultured_bacterium
435	0	0	0	3	0	0	0	0	0	0	0	0	k__Bacteria; p__Proteobacteria; c__Gammaproteobacteria
887	0	110	0	0	0	0	0	0	0	8	0	0	k__Bacteria; p__Bacteroidetes; c__Flavobacteriia; o__Flavobacteriales; f__Flavobacteriaceae; g__NS3a_marine_group; Ambiguous_taxa
846	7	0	0	0	0	0	0	0	0	0	0	0	k__Bacteria; p__Bacteroidetes; c__Flavobacteriia; o__Flavobacteriales; f__Cryomorphaceae; g__Brumimicrobium; s__uncultured_bacterium
805	12	1	3	0	0	0	0	0	0	0	0	0	k__Bacteria; p__Bacteroidetes; c__Bacteroidetes_VC2.1_Bac22
477	0	0	0	0	38	60	0	0	0	3	0	0	k__Bacteria; p__Proteobacteria; c__Alphaproteobacteria; o__Rhodospirillales; f__Rhodospirillaceae; g__Thalassospira; s__Azospirillum_sp._CAG:260
436	0	2	20	12	0	0	0	0	0	0	0	0	k__Bacteria; p__Bacteroidetes; c__Sphingobacteriia; o__Sphingobacteriales; f__Saprospiraceae; g__uncultured; s__uncultured_Bacteroidetes_bacterium
888	0	10	9	1	0	1	1	0	3	0	0	0	k__Bacteria; p__Fibrobacteres; c__Fibrobacteria; o__Fibrobacteria_Incertae_Sedis; f__Unknown_Family; g__possible_genus_03; s__uncultured_bacterium
847	3	4	15	0	0	0	0	0	0	0	0	0	k__Bacteria; p__Proteobacteria; c__Deltaproteobacteria; o__Desulfobacterales; f__Desulfobulbaceae

806	11	1	1	0	0	1	3	0	0	0	0	0	k__Bacteria; p__Bacteroidetes; c__Flavobacteriia; o__Flavobacteriales; f__Cryomorphaceae; g__Crocinitomix; Ambiguous_taxa
478	0	5	5	0	5	2	0	0	0	7	3	2	k__Bacteria; p__Tenericutes; c__Mollicutes; o__NB1_n
437	10	7	22	2	0	0	0	2	0	0	0	0	k__Bacteria; p__Proteobacteria; c__Deltaproteobacteria; o__Desulfuromonadales; f__Sva1033
889	2	24	9	0	0	0	0	0	0	0	0	0	k__Bacteria; p__Proteobacteria; c__Epsilonproteobacteria; o__Campylobacterales; f__Campylobacteraceae; g__Arcobacter
848	19	9	23	0	0	0	0	2	1	4	1	0	k__Bacteria; p__Fusobacteria; c__Fusobacteriia; o__Fusobacteriales; f__Leptotrichiaceae; g__uncultured; Ambiguous_taxa
807	12	0	3	0	0	0	0	0	0	1	0	0	k__Bacteria; p__Proteobacteria; c__Gammaproteobacteria; o__HOC36
479	0	0	0	0	36	124	33	1	21	25	48	18	k__Bacteria; p__Firmicutes; c__Clostridia; o__Clostridiales; f__Clostridiales_vadinBB60_group
438	0	0	0	5	1	0	0	0	0	0	0	0	k__Bacteria; p__Proteobacteria; c__Deltaproteobacteria; o__Desulfobacterales; f__Desulfobulbaceae; g__Desulfobulbus; s__uncultured_delta_proteobacterium
849	9	5	15	0	0	0	0	2	4	0	0	0	k__Bacteria; p__Bacteroidetes; c__Bacteroidia; o__Bacteroidia_Incertae_Sedis; f__Draconibacteriaceae; g__Draconibacterium
808	10	3	7	0	0	0	0	0	5	0	0	0	k__Bacteria; p__Bacteroidetes; c__Flavobacteriia; o__Flavobacteriales; f__Flavobacteriaceae; g__Lutibacter; Ambiguous_taxa
439	0	0	0	8	0	1	0	0	0	0	0	0	k__Bacteria; p__Proteobacteria; c__Gammaproteobacteria; o__Oceanospirillales; f__Oceanospirillaceae; g__Reinekea

809	9	0	5	0	0	0	0	1	0	0	0	0	k__Bacteria; p__Proteobacteria; c__Gammaproteobacteria; o__Run_SP154; f__uncultured_gamma_proteobacterium; g__uncultured_gamma_proteobacterium; s__uncultured_gamma_proteobacterium
1590	0	0	0	0	0	0	9	11	0	0	0	0	k__Bacteria; p__Spirochaetae; c__Spirochaetes; o__Spirochaetales; f__Spirochaetaceae; g__M2PT2_76_termite_group; s__uncultured_bacterium
1180	0	1	9	1	0	0	0	0	0	0	0	0	k__Bacteria; p__Proteobacteria; c__Deltaproteobacteria; o__Sh765B_TzT_29; f__uncultured_prokaryote; g__uncultured_prokaryote; s__uncultured_prokaryote
1591	0	0	0	0	0	0	24	19	9	0	0	0	k__Bacteria; p__Bacteroidetes; c__Flavobacteriia; o__Flavobacteriales; f__Cryomorphaceae; g__Owenweeksia
1550	0	0	0	0	0	0	6	2	8	0	0	0	k__Bacteria; p__Proteobacteria; c__Gammaproteobacteria; o__Chromatiales; f__Granulosicoccaceae; g__Granulosicoccus; s__uncultured_bacterium
1181	0	0	5	0	0	0	3	0	1	0	0	0	k__Bacteria
1140	0	0	21	0	0	0	0	0	0	0	0	0	k__Bacteria; p__Proteobacteria; c__Betaproteobacteria; o__Burkholderiales; f__Burkholderiaceae; g__Cupriavidus
1592	0	0	0	0	0	0	5	0	0	0	0	0	k__Bacteria; p__Chloroflexi; c__Dehalococcoidia
1551	0	0	0	0	0	0	40	16	34	0	0	0	k__Bacteria; p__Bacteroidetes; c__Bacteroidia; o__Bacteroidales; f__Marinilabiaceae; g__uncultured
1510	0	0	0	0	0	0	8	4	5	0	0	0	k__Bacteria; p__Proteobacteria; c__Deltaproteobacteria; o__Desulfobacterales; f__Desulfobulbaceae
1182	0	0	5	0	4	0	0	0	0	0	0	0	k__Bacteria; p__Bacteroidetes; c__Sphingobacteriia; o__Sphingobacteriales; f__WCHB1_69

1141	0	0	5	0	0	0	0	0	0	0	0	0	k__Bacteria; p__Bacteroidetes; c__Bacteroidetes_VC2.1_Bac22; o__uncultured_Bacteroidetes_bacterium; f__uncultured_Bacteroidetes_bacterium; g__uncultured_Bacteroidetes_bacterium; s__uncultured_Bacteroidetes_bacterium
1100	0	0	45	0	0	1	0	1	0	16	3	1	k__Bacteria; p__Firmicutes; c__Clostridia; o__Clostridiales; f__Ruminococcaceae; g__Ruminococcaceae_NK4A214_group
1593	0	0	0	0	0	0	3	0	0	0	0	0	k__Bacteria; p__Proteobacteria; c__Alphaproteobacteria; o__Rhodospirillales; f__Rhodospirillaceae; g__Magnetovibrio
1552	0	2	2	0	0	0	15	3	6	0	0	0	k__Bacteria
1511	0	0	0	0	0	0	14	16	45	0	0	0	k__Bacteria; p__Bacteroidetes; c__Cytophagia; o__Cytophagales; f__Flammeovirgaceae; g__Reichenbachiella
1183	0	0	7	0	1	2	7	1	0	0	0	0	k__Bacteria; p__Proteobacteria; c__Deltaproteobacteria; o__Desulfobacterales; f__Desulfobulbaceae; g__Desulfobulbus; Ambiguous_taxa
1142	0	0	19	0	0	0	0	0	0	0	0	0	k__Bacteria; p__Proteobacteria; c__Gammaproteobacteria; o__HOC36; f__uncultured_sediment_bacterium; g__uncultured_sediment_bacterium; s__uncultured_sediment_bacterium
1101	0	0	7	0	0	0	0	0	1	0	0	0	k__Bacteria; p__Gracilibacteria
1594	0	0	0	0	4	0	23	42	34	0	0	0	k__Bacteria; p__Firmicutes; c__Clostridia; o__Clostridiales; f__Clostridiaceae_1
1553	0	0	0	0	0	0	19	13	15	0	0	0	k__Bacteria; p__Proteobacteria; c__Deltaproteobacteria; o__Desulfobacterales; f__Desulfobacteraceae
1512	5	0	0	0	0	0	14	20	9	0	0	0	k__Bacteria; p__Proteobacteria; c__Epsilonproteobacteria; o__Campylobacterales; f__Helicobacteraceae; g__Sulfurimonas

1184	0	0	13	0	0	0	0	0	0	0	0	0	k__Bacteria; p__Bacteroidetes; c__Sphingobacteriia; o__Sphingobacteriales; f__Saprospiraceae
1143	0	0	15	0	0	0	0	0	0	0	0	0	k__Bacteria; p__Acidobacteria; c__Subgroup_22
1102	0	0	7	0	0	2	0	0	0	3	0	0	k__Bacteria; p__Proteobacteria; c__Alphaproteobacteria; o__Rhodobacterales; f__Rhodobacteraceae; g__Gemmobacter; Ambiguous_taxa
1595	0	0	0	0	0	0	16	0	0	0	0	0	k__Bacteria; p__Proteobacteria; c__Gammaproteobacteria; o__Alteromonadales; f__Pseudoalteromonadaceae
1554	0	0	0	0	0	0	11	4	3	0	0	0	k__Bacteria; p__Proteobacteria; c__Gammaproteobacteria; o__Chromatiales
1513	0	0	0	0	0	0	3	0	0	0	0	0	k__Bacteria; p__Proteobacteria; c__Gammaproteobacteria
1185	0	0	10	0	0	0	0	0	0	0	0	0	k__Bacteria; p__WCHB1_60; c__uncultured_Candidatus_Saccharibacteria_bacterium; o__uncultured_Candidatus_Saccharibacteria_bacterium; f__uncultured_Candidatus_Saccharibacteria_bacterium; g__uncultured_Candidatus_Saccharibacteria_bacterium; s__uncultured_Candidatus_Saccharibacteria_bacterium
1144	0	0	8	2	0	0	0	6	2	0	0	0	k__Bacteria; p__Nitrospirae; c__Nitrospira; o__Nitrospirales; f__Nitrospiraceae; g__Nitrospira; Ambiguous_taxa
1103	0	0	8	0	0	7	0	0	0	0	0	0	k__Bacteria; p__Proteobacteria; c__Deltaproteobacteria; o__Myxococcales; f__PS_B29; g__uncultured_bacterium; s__uncultured_bacterium
1596	0	0	1	0	0	0	6	18	3	0	0	0	k__Bacteria; p__Proteobacteria; c__Deltaproteobacteria; o__Desulfobacterales; f__Desulfobacteraceae
1555	0	0	0	0	0	0	22	2	12	0	0	0	k__Bacteria; p__Proteobacteria; c__Gammaproteobacteria; o__Oceanospirillales; f__Alcanivoracaceae; g__Kangiella

1514	0	0	0	0	0	0	31	8	7	0	0	0	k__Bacteria; p__Bacteroidetes
1186	0	0	8	0	0	0	0	0	0	0	0	0	k__Bacteria; p__Bacteroidetes
1145	0	0	5	0	0	0	0	0	2	0	0	0	k__Bacteria; p__Bacteroidetes; c__Bacteroidia; o__Bacteroidales; f__Marinilabiaceae; g__uncultured
1104	0	0	68	0	0	0	68	80	51	8	1	0	k__Bacteria; p__Firmicutes; c__Clostridia; o__Clostridiales; f__Ruminococcaceae; g__Ruminococcaceae_UCG_014
1597	0	0	0	0	0	0	9	7	0	0	0	0	k__Bacteria; p__Proteobacteria; c__Deltaproteobacteria; o__Desulfobacterales; f__Desulfobacteraceae
1556	0	0	0	0	0	1	9	23	15	0	0	0	k__Bacteria; p__Actinobacteria; c__Actinobacteria; o__Propionibacteriales; f__Nocardioidaceae; g__Nocardioides; Ambiguous_taxa
1515	6	1	0	10	2	0	21	25	36	1	0	0	k__Bacteria; p__Proteobacteria; c__Gammaproteobacteria
1187	0	0	10	0	0	0	0	0	0	0	0	0	k__Bacteria; p__Acidobacteria; c__Subgroup_22; o__uncultured_bacterium; f__uncultured_bacterium; g__uncultured_bacterium; s__uncultured_bacterium
1146	0	0	2	0	0	0	0	0	0	0	0	0	k__Bacteria; p__Spirochaetae; c__Spirochaetes; o__Spirochaetales; f__Brevinemataceae; g__Brevinema
1105	0	118	81	41	68	81	27	0	45	45	75	15	k__Bacteria; p__Bacteroidetes; c__Bacteroidia; o__Bacteroidales; f__Bacteroidales_S24_7_group; g__uncultured_bacterium; s__uncultured_bacterium
1598	0	0	0	0	0	0	7	1	0	0	0	0	k__Bacteria; p__Bacteroidetes; c__Flavobacteriia; o__Flavobacteriales; f__Cryomorphaceae
1557	0	0	0	0	0	0	9	0	0	0	0	0	k__Bacteria; p__Chlorobi; c__Ignavibacteria; o__Ignavibacteriales; f__IheB3_7
1516	0	0	0	0	0	0	23	0	0	0	0	0	k__Bacteria; p__Proteobacteria; c__Deltaproteobacteria; o__Myxococcales; f__VHS_B4_70;

													g__uncultured_delta_proteobacterium; s__uncultured_delta_proteobacterium
1188	0	3	3	0	0	0	0	0	0	0	0	0	k__Bacteria; p__Proteobacteria; c__Gammaproteobacteria; o__Pseudomonadales; f__Pseudomonadaceae; g__Pseudomonas
1147	0	0	4	0	0	0	0	0	0	0	4	3	k__Bacteria; p__Firmicutes; c__Clostridia; o__Clostridiales; f__Lachnospiraceae
1106	0	0	3	0	0	0	0	0	0	0	0	0	k__Bacteria; p__Bacteroidetes; c__Cytophagia; o__Cytophagales; f__Flammeovirgaceae; g__Reichenbachiella; s__uncultured_bacterium
1599	2	1	8	0	0	0	9	9	5	0	0	0	k__Bacteria; p__Proteobacteria; c__Gammaproteobacteria; o__Cellvibrionales; f__BD2_7; g__uncultured_gamma_proteobacterium; s__uncultured_gamma_proteobacterium
1558	0	0	2	0	0	0	12	7	1	0	0	0	k__Bacteria; p__Proteobacteria; c__Alphaproteobacteria; o__Rhodobacterales; f__Rhodobacteraceae
1517	0	0	0	0	0	0	8	0	14	0	0	0	k__Bacteria; p__Bacteroidetes; c__Cytophagia; o__Cytophagales; f__Flammeovirgaceae; g__uncultured
1189	0	5	6	0	0	0	0	0	0	0	0	0	k__Bacteria; p__Bacteroidetes; c__Flavobacteriia; o__Flavobacteriales; f__Cryomorphaceae; g__Crocinitomix; s__uncultured_bacterium
1148	0	0	2	0	0	0	0	0	0	0	0	0	k__Bacteria; p__Cloacimonetes
1107	0	0	6	1	0	0	0	1	7	0	0	0	k__Bacteria; p__Proteobacteria; c__Gammaproteobacteria; o__Gammaproteobacteria_Incertae_Sedis; f__Unknown_Family; g__Sedimenticola; s__uncultured_gamma_proteobacterium
1559	0	0	0	3	0	10	204	15	54	0	0	0	k__Bacteria; p__Proteobacteria; c__Gammaproteobacteria; o__Alteromonadales; f__Colwelliaceae; g__Colwellia

1518	0	0	0	1	0	0	27	10	55	0	0	0	k__Bacteria; p__Proteobacteria; c__Alphaproteobacteria; o__Rhodobacterales; f__Rhodobacteraceae
1149	0	0	6	0	0	0	0	0	0	0	0	0	k__Bacteria; p__Bacteroidetes; c__Sphingobacteriia; o__Sphingobacteriales; f__Saprospiraceae
1108	1	2	13	0	2	0	0	0	0	0	0	0	k__Bacteria; p__Proteobacteria; c__Milano_WF1B_44; Ambiguous_taxa; Ambiguous_taxa; Ambiguous_taxa; Ambiguous_taxa
1519	0	0	2	1	0	0	32	13	18	0	0	0	k__Bacteria; p__Proteobacteria; c__Gammaproteobacteria; o__Thiotrichales; f__Thiotrichaceae; g__uncultured
1109	0	0	2	0	0	0	0	0	0	0	0	0	k__Bacteria; p__Acidobacteria; c__Subgroup_22
1	136	0	45	0	114	68	0	76	63	52	102	39	k__Bacteria; p__Bacteroidetes; c__Bacteroidia; o__Bacteroidales; f__Bacteroidales_S24_7_group; g__uncultured_bacterium; s__uncultured_bacterium
2	146	105	9	27	34	66	5	1	69	30	8	3	k__Bacteria; p__Firmicutes; c__Negativicutes; o__Selenomonadales; f__Veillonellaceae; g__Anaerovibrio; s__uncultured_bacterium
3	387	492	114	269	309	92	110	209	42	116	213	106	k__Bacteria; p__Firmicutes; c__Clostridia; o__Clostridiales; f__Clostridiales_vadinBB60_group; Ambiguous_taxa; Ambiguous_taxa
4	441	2010	2934	3181	1466	2062	1663	3231	3078	331	69	64	k__Bacteria; p__Firmicutes; c__Bacilli; o__Bacillales; f__Bacillaceae; g__Bacillus; s__Bacillus_cereus
5	293	70	48	72	106	141	39	152	30	59	38	8	k__Bacteria; p__Bacteroidetes; c__Bacteroidia; o__Bacteroidales; f__Prevotellaceae; g__Prevotella_2; s__uncultured_bacterium
6	441	274	30	257	98	173	143	176	136	5194	53	9	k__Bacteria; p__Bacteroidetes; c__Bacteroidia; o__Bacteroidales; f__Prevotellaceae; g__Prevotella_9;

													s__uncultured_bacterium
8	43	386	139	256	259	187	29	269	1	119	224	29	k__Bacteria; p__Firmicutes; c__Clostridia; o__Clostridiales; f__Lachnospiraceae; g__Lachnospiraceae_NK4A136_group; s__uncultured_bacterium
9	262	554	141	518	629	955	407	425	805	292	601	189	k__Bacteria; p__Firmicutes; c__Bacilli; o__Lactobacillales; f__Lactobacillaceae; g__Lactobacillus; s__Lactobacillus_vaginalis
770	6	7	7	2	3	0	0	0	0	0	0	0	k__Bacteria; p__Actinobacteria; c__Acidimicrobiia; o__Acidimicrobiales; f__OM1_clade
360	0	0	0	76	66	0	0	0	0	2	19	20	k__Bacteria; p__Firmicutes; c__Clostridia; o__Clostridiales; f__Lachnospiraceae; g__uncultured; s__uncultured_bacterium
771	15	25	30	0	1	0	0	0	0	0	0	0	k__Bacteria; p__Proteobacteria; c__Gammaproteobacteria; o__Alteromonadales; f__Alteromonadaceae; g__Agarivorans; Ambiguous_taxa
730	0	36	10	3	0	0	0	0	0	2	0	0	k__Bacteria; p__Proteobacteria; c__Gammaproteobacteria; o__Oceanospirillales; f__Oceanospirillaceae; g__Amphritea; Ambiguous_taxa
361	0	0	0	7	0	0	0	0	0	0	0	0	k__Bacteria; p__Bacteroidetes; c__Sphingobacteriia; o__Sphingobacteriales; f__Saprospiraceae; g__Portibacter; s__uncultured_Bacteroidetes_bacterium
320	0	0	0	58	42	74	0	0	0	0	0	0	k__Bacteria; p__Proteobacteria; c__Gammaproteobacteria; o__Alteromonadales; f__Colwelliaceae; g__Colwellia
772	100	1	0	0	0	0	2	1	0	0	3	0	k__Bacteria; p__Firmicutes; c__Clostridia; o__Clostridiales; f__Lachnospiraceae; g__Butyrivibrio; s__uncultured_bacterium
731	5	0	4	0	1	0	0	0	0	0	0	0	k__Bacteria; p__Proteobacteria; c__Gammaproteobacteria;

													o__Chromatiales; f__Ectothiorhodospiraceae; g__Thiogranum
362	0	0	3	3	0	0	0	0	0	0	0	0	k__Bacteria; p__Bacteroidetes; c__Bacteroidetes_BD2_2; o__uncultured_bacterium; f__uncultured_bacterium; g__uncultured_bacterium; s__uncultured_bacterium
321	0	12	5	95	120	113	0	0	0	0	0	0	k__Bacteria; p__Proteobacteria; c__Gammaproteobacteria; o__Vibrionales; f__Vibrionaceae; g__Vibrio; Ambiguous_taxa
773	206	0	0	0	0	0	0	0	0	0	0	0	k__Bacteria; p__Actinobacteria; c__Actinobacteria; o__Kineosporiales; f__Kineosporiaceae; g__Quadrisphaera
732	0	1	1	0	0	0	0	0	0	0	0	0	k__Bacteria; p__Bacteroidetes
363	0	0	47	32	87	0	65	0	29	49	10	1	k__Bacteria; p__Firmicutes; c__Clostridia; o__Clostridiales; f__Ruminococcaceae; g__Ruminococcus_1; Ambiguous_taxa
322	0	120	0	0	0	0	0	0	0	6	2	4	k__Bacteria; p__Bacteroidetes
774	222	0	0	0	0	1	0	0	0	0	4	2	k__Bacteria; p__Firmicutes; c__Clostridia; o__Clostridiales; f__Lachnospiraceae; g__Coprococcus_1; s__uncultured_bacterium
733	3	6	1	0	0	0	2	0	0	0	0	0	k__Bacteria; p__Proteobacteria; c__Deltaproteobacteria; o__Bdellovibrionales; f__Bacteriovoracaceae; g__Peredibacter
364	0	0	2	11	10	34	4	2	0	0	0	0	k__Bacteria; p__Proteobacteria; c__Gammaproteobacteria; o__Alteromonadales; f__Alteromonadaceae
323	0	0	0	110	19	0	0	0	65	4	39	104	k__Bacteria; p__Firmicutes; c__Clostridia; o__Clostridiales; f__Lachnospiraceae
775	174	0	0	0	0	0	0	1	0	0	1	1	k__Bacteria; p__Bacteroidetes; c__Bacteroidia; o__Bacteroidales; f__uncultured; g__uncultured_bacterium; s__uncultured_bacterium
734	4	42	19	0	0	0	0	0	0	0	0	0	k__Bacteria; p__Proteobacteria; c__Gammaproteobacteria;

													o__Oceanospirillales; f__Oceanospirillaceae; g__Reinekea; Ambiguous_taxa
365	0	0	0	72	0	0	17	0	47	9	47	29	k__Bacteria; p__Firmicutes; c__Clostridia; o__Clostridiales; f__Lachnospiraceae; g__Lachnospiraceae_UCG_008; s__uncultured_Clostridiales_bacterium
324	0	0	0	7	3	9	0	0	0	0	0	0	k__Bacteria; p__Proteobacteria; c__Gammaproteobacteria; o__Thiotrichales; f__Piscirickettsiaceae; g__endosymbionts
776	6	10	3	0	0	0	0	5	0	0	0	0	k__Bacteria; p__Bacteroidetes; c__Flavobacteriia; o__Flavobacteriales; f__Cryomorphaceae
735	4	6	6	0	0	0	0	0	0	0	0	0	k__Bacteria; p__Bacteroidetes; c__Flavobacteriia; o__Flavobacteriales; f__Flavobacteriaceae; g__Nonlabens; Ambiguous_taxa
366	0	0	0	59	0	0	0	0	0	11	11	14	k__Bacteria; p__Firmicutes; c__Clostridia; o__Clostridiales; f__Ruminococcaceae; g__Ruminococcaceae_UCG_010
325	0	0	0	6	0	0	0	0	0	0	0	0	k__Bacteria; p__Proteobacteria; c__Gammaproteobacteria; o__Xanthomonadales; f__Solimonadaceae; g__Polycyclovorans; Ambiguous_taxa
777	20	0	1	0	0	0	0	0	0	0	0	0	k__Bacteria; p__Bacteroidetes; c__Bacteroidia; o__Bacteroidales; f__Marinilabiaceae; g__Marinifilum; Ambiguous_taxa
736	0	4	18	0	0	4	0	0	0	0	0	0	k__Bacteria; p__Proteobacteria; c__Gammaproteobacteria; o__Cellvibrionales; f__Halieaceae; Ambiguous_taxa; Ambiguous_taxa
367	0	0	1	56	0	146	0	0	1	63	13	0	k__Bacteria; p__Firmicutes; c__Clostridia; o__Clostridiales; f__Ruminococcaceae; g__Ruminococcaceae_UCG_014; Ambiguous_taxa

326	0	0	4	5	0	3	2	0	0	0	0	0	k__Bacteria; p__Bacteroidetes; c__Flavobacteriia; o__Flavobacteriales
778	190	0	0	0	1	0	1	1	0	87	0	1	k__Bacteria; p__Firmicutes; c__Clostridia; o__Clostridiales; f__Lachnospiraceae
737	0	16	11	0	0	0	0	0	0	0	0	0	k__Bacteria; p__Proteobacteria; c__Gammaproteobacteria; o__Pseudomonadales; f__Moraxellaceae; g__Acinetobacter
368	36	7	34	16	11	15	0	0	0	0	0	0	k__Bacteria; p__Bacteroidetes; c__Bacteroidia; o__Bacteroidia_Incertae_Sedis; f__Draconibacteriaceae; g__Draconibacterium; Ambiguous_taxa
327	0	0	1	69	2	0	56	1	0	34	15	1	k__Bacteria; p__Firmicutes; c__Clostridia; o__Clostridiales; f__Ruminococcaceae; g__Ruminococcaceae_UCG_014
779	8	0	6	0	0	0	0	0	0	0	0	0	k__Bacteria; p__Proteobacteria; c__Deltaproteobacteria
738	0	0	13	0	0	0	0	0	0	0	0	0	k__Bacteria; p__Proteobacteria; c__Gammaproteobacteria; o__Cellvibrionales; f__Halieaceae; g__Haliea
369	0	0	0	92	12	0	0	42	4	17	12	1	k__Bacteria; p__Firmicutes; c__Clostridia; o__Clostridiales; f__Ruminococcaceae; g__Ruminococcaceae_UCG_005
328	196	0	66	33	49	18	35	1	152	47	76	38	k__Bacteria; p__Bacteroidetes; c__Bacteroidia; o__Bacteroidales; f__Bacteroidales_S24_7_group; Ambiguous_taxa; Ambiguous_taxa
739	0	10	2	0	0	0	0	0	1	0	0	0	k__Bacteria; p__Bacteroidetes; c__Bacteroidetes_VC2.1_Bac22; o__uncultured_Bacteroidetes_bacterium; f__uncultured_Bacteroidetes_bacterium; g__uncultured_Bacteroidetes_bacterium; s__uncultured_Bacteroidetes_bacterium
329	0	0	0	36	0	0	0	0	0	4	0	0	k__Bacteria; p__Firmicutes; c__Negativicutes; o__Selenomonadales; f__Veillonellaceae; g__uncultured;

													s__uncultured_rumen_bacterium_4C28d_2
1480	8	0	1	0	0	0	70	1	5	0	0	0	k__Bacteria; p__Proteobacteria; c__Gammaproteobacteria; o__Alteromonadales; f__Colwelliaceae; g__Thalassotalea
1070	0	0	13	0	0	4	0	0	0	0	0	0	k__Bacteria; p__Bacteroidetes; c__Sphingobacteriia; o__Sphingobacteriales; f__WCHB1_69
1481	0	0	0	0	0	0	25	55	90	0	0	0	k__Bacteria; p__Proteobacteria; c__Alphaproteobacteria; o__Rickettsiales; f__SAR116_clade
1440	0	0	0	0	0	0	0	0	0	7	0	0	k__Bacteria; p__Proteobacteria; c__Betaproteobacteria; o__Nitrosomonadales; f__Nitrosomonadaceae; g__uncultured
1030	17	22	83	4	8	6	0	8	1	0	0	0	k__Bacteria; p__Proteobacteria; c__Gammaproteobacteria; o__Order_Incertae_Sedis; f__Family_Incertae_Sedis; g__Marinicella
1071	0	0	3	0	0	0	0	0	0	0	0	0	k__Bacteria; p__Bacteroidetes; c__Bacteroidia; o__Bacteroidales; f__Marinilabiaceae; g__Carboxylicivirga; s__uncultured_bacterium
1482	0	0	4	0	0	0	36	49	57	0	0	0	k__Bacteria; p__Proteobacteria; c__Deltaproteobacteria; o__Desulfobacterales; f__Desulfobacteraceae; g__Desulfosarcina
1441	0	0	0	0	0	0	0	0	0	54	0	0	k__Bacteria; p__Firmicutes; c__Clostridia; o__Clostridiales; f__Ruminococcaceae; g__Ruminococcaceae_UCG_014
1400	0	0	0	0	0	0	1	0	0	33	0	0	k__Bacteria; p__Firmicutes; c__Clostridia; o__Clostridiales; f__Ruminococcaceae; g__Oscillospira; s__uncultured_bacterium
1031	0	7	1	0	1	6	0	0	0	0	1	0	k__Bacteria; p__Proteobacteria; c__Deltaproteobacteria; o__Desulfobacterales; f__Desulfobacteraceae; g__Desulfobacula; s__uncultured_delta_proteobacterium

1072	3	0	21	0	0	0	0	0	0	0	0	0	k__Bacteria; p__Chlorobi; c__Ignavibacteria; o__Ignavibacteriales; f__IheB3_7; Ambiguous_taxa; Ambiguous_taxa
1483	0	0	0	0	0	0	43	0	0	0	0	0	k__Bacteria
1442	0	0	0	0	0	0	0	0	1	3	4	2	k__Bacteria; p__Firmicutes; c__Erysipelotrichia; o__Erysipelotrichales; f__Erysipelotrichaceae; g__Erysipelotrichaceae_UCG_004; s__uncultured_bacterium
1401	0	0	0	0	0	0	1	0	0	94	3	0	k__Bacteria; p__Actinobacteria; c__Coriobacteriia; o__Coriobacteriales; f__Coriobacteriaceae; g__Collinsella; s__uncultured_bacterium
1032	0	4	9	0	0	0	0	1	3	3	0	2	k__Bacteria; p__Tenericutes; c__Mollicutes; o__NB1_n
1073	0	0	4	1	0	0	0	0	0	0	0	0	k__Bacteria; p__Proteobacteria; c__Gammaproteobacteria
1484	0	0	0	0	0	0	6	0	6	0	0	0	k__Bacteria; p__Proteobacteria; c__Gammaproteobacteria; o__Cellvibrionales; f__BD2_7; g__uncultured_gamma_proteobacterium; s__uncultured_gamma_proteobacterium
1443	0	0	0	0	0	0	0	0	0	10	0	0	k__Bacteria; p__Firmicutes; c__Clostridia; o__Clostridiales; f__Ruminococcaceae
1402	0	0	0	0	0	0	0	0	0	26	0	0	k__Bacteria; p__Bacteroidetes; c__Bacteroidia; o__Bacteroidales; f__Bacteroidaceae; g__Bacteroides; s__uncultured_bacterium
1033	0	12	0	0	0	0	0	0	0	0	0	0	k__Bacteria; p__Proteobacteria; c__Gammaproteobacteria; o__NKB5
1074	0	0	1	1	0	0	0	0	0	0	0	0	k__Bacteria; p__Firmicutes; c__Clostridia; o__Clostridiales; f__Kazan_2B_17; Ambiguous_taxa; Ambiguous_taxa
1485	0	0	0	0	0	0	17	13	13	0	0	0	k__Bacteria; p__Tenericutes; c__Mollicutes;

												o__Mycoplasmatales; f__Mycoplasmataceae
1444	0	0	0	0	0	0	0	0	0	9	4	6	k__Bacteria; p__Firmicutes; c__Clostridia; o__Clostridiales; f__Ruminococcaceae; g__Faecalibacterium; s__uncultured_bacterium
1403	0	0	0	9	0	0	0	0	1	68	0	0	k__Bacteria; p__Firmicutes; c__Clostridia; o__Clostridiales; f__Lachnospiraceae
1034	0	1	35	0	0	0	0	0	0	0	6	3	k__Bacteria; p__Proteobacteria; c__Deltaproteobacteria; o__Desulfovibrionales; f__Desulfovibrionaceae; g__Desulfovibrio; Ambiguous_taxa
1075	1	0	10	0	1	0	0	0	0	0	0	0	k__Bacteria; p__Proteobacteria; c__Gammaproteobacteria; o__Cellvibrionales; f__Halieaceae
1486	0	1	0	0	0	0	14	1	23	0	0	0	k__Bacteria; p__Proteobacteria; c__Gammaproteobacteria; o__Oceanospirillales; f__Oceanospirillaceae; g__Neptunomonas; s__gamma_proteobacterium_HS6(2014)
1445	0	0	0	0	0	0	0	0	0	6	0	0	k__Bacteria; p__Firmicutes; c__Clostridia; o__Clostridiales; f__Lachnospiraceae; g__Coprococcus_1
1404	0	0	0	0	0	0	0	0	0	17	0	0	k__Bacteria; p__Firmicutes; c__Clostridia; o__Clostridiales; f__Ruminococcaceae; g__Ruminococcaceae_UCG_005
1035	0	2	25	0	0	0	0	0	0	0	0	0	k__Bacteria; p__Proteobacteria; c__Deltaproteobacteria; o__Desulfobacterales; f__Desulfobulbaceae; g__MSBL7; s__uncultured_delta_proteobacterium
1076	0	0	16	0	0	1	0	3	2	0	0	0	k__Bacteria; p__Proteobacteria; c__Alphaproteobacteria; o__Sphingomonadales; f__Sphingomonadaceae; g__Sphingomonas
1487	0	0	0	0	0	0	36	34	51	0	0	0	k__Bacteria; p__Proteobacteria; c__Gammaproteobacteria; o__Alteromonadales; f__Colwelliaceae

1446	0	0	0	0	0	0	0	0	0	2	0	0	k__Bacteria; p__Firmicutes; c__Clostridia; o__Clostridiales; f__Family_XII; g__Fusibacter
1405	0	0	0	0	0	0	0	0	0	17	0	0	k__Bacteria; p__Firmicutes; c__Clostridia; o__Clostridiales; f__Ruminococcaceae
1036	0	4	0	0	0	0	0	0	0	0	0	0	k__Bacteria; p__Proteobacteria; c__Deltaproteobacteria; o__Myxococcales; f__UASB_TL25; g__uncultured_delta_proteobacterium; s__uncultured_delta_proteobacterium
1077	0	2	20	5	0	3	0	0	0	0	0	0	k__Bacteria; p__Proteobacteria; c__Deltaproteobacteria; o__Desulfuromonadales
1488	0	0	0	6	0	0	182	89	168	0	0	0	k__Bacteria; p__Proteobacteria; c__Gammaproteobacteria; o__Cellvibrionales; f__Cellvibrionaceae; g__uncultured
1447	0	0	0	0	0	0	1	0	0	21	0	0	k__Bacteria; p__Firmicutes; c__Clostridia; o__Clostridiales; f__Ruminococcaceae; g__Anaerotruncus
1406	0	0	0	0	0	0	0	0	0	6	0	0	k__Bacteria; p__Bacteroidetes; c__Bacteroidia; o__Bacteroidales; f__Bacteroidales_S24_7_group
1037	1	16	7	0	0	0	0	0	0	0	0	0	k__Bacteria; p__Proteobacteria; c__Deltaproteobacteria; o__Myxococcales; f__Sandaracinaceae; g__uncultured
1078	0	0	7	0	0	0	0	0	0	0	0	0	k__Bacteria; p__Actinobacteria; c__Nitriliruptoria; o__Nitriliruptorales; f__Nitriliruptoraceae; g__Nitriliruptor
1489	0	0	1	0	0	0	181	176	220	1	0	0	k__Bacteria; p__Proteobacteria; c__Deltaproteobacteria; o__Oligoflexales; f__Oligoflexaceae; g__uncultured_bacterium; s__uncultured_bacterium
1448	0	0	0	0	0	0	0	0	0	23	0	0	k__Bacteria; p__Firmicutes; c__Clostridia; o__Clostridiales; f__Lachnospiraceae; g__Anaerostipes; s__uncultured_bacterium
1407	0	0	0	0	0	0	1	0	0	6	2	0	k__Bacteria; p__Firmicutes; c__Clostridia; o__Clostridiales;

													f__Ruminococcaceae; g__Ruminococcus_2
1038	0	7	0	3	0	1	0	0	0	0	0	0	k__Bacteria; p__Gracilibacteria
1079	0	0	46	0	0	0	0	0	0	0	0	0	k__Bacteria; p__Proteobacteria; c__Deltaproteobacteria; o__Bdellovibrionales; f__Bdellovibrionaceae; g__OM27_clade; s__uncultured_organism
1449	0	0	0	0	0	0	0	0	0	11	0	0	k__Bacteria; p__Firmicutes; c__Clostridia; o__Clostridiales; f__Lachnospiraceae; g__Lachnoclostridium
1408	0	0	0	0	0	0	0	0	0	24	0	0	k__Bacteria; p__Firmicutes; c__Erysipelotrichia; o__Erysipelotrichales; f__Erysipelotrichaceae; Ambiguous_taxa; Ambiguous_taxa
1039	2	6	15	0	0	0	0	0	0	0	0	0	k__Bacteria; p__Proteobacteria; c__Gammaproteobacteria; o__Cellvibrionales; f__Spongiibacteraceae; g__BD1_7_clade
1409	0	0	0	0	0	0	0	0	0	27	0	0	k__Bacteria; p__Bacteroidetes; c__Bacteroidia; o__Bacteroidales; f__Porphyromonadaceae; g__Parabacteroides; s__Porphyromonadaceae_bacterium_DJF_B175
290	0	38	0	0	63	0	0	0	48	45	15	16	k__Bacteria; p__Firmicutes; c__Clostridia; o__Clostridiales; f__Lachnospiraceae
660	0	0	0	0	0	0	3	0	0	0	4	0	k__Bacteria; p__Firmicutes; c__Clostridia; o__Clostridiales; f__Lachnospiraceae
291	7	20	59	40	9	29	24	41	13	2	2	0	k__Bacteria; p__Proteobacteria; c__Gammaproteobacteria; o__Vibrionales; f__Vibrionaceae; g__Vibrio
250	157	0	0	0	41	33	89	54	43	21	7	5	k__Bacteria; p__Firmicutes; c__Clostridia; o__Clostridiales; f__Lachnospiraceae
661	105	159	358	0	0	0	0	0	0	0	0	0	k__Bacteria; p__Bacteroidetes; c__Flavobacteriia; o__Flavobacteriales; f__Flavobacteriaceae

620	0	0	57	0	0	0	0	102	0	23	47	14	k__Bacteria; p__Firmicutes; c__Clostridia; o__Clostridiales; f__Ruminococcaceae; g__Ruminococcaceae_UCG_009; s__uncultured_bacterium
292	0	0	30	0	19	0	1	2	0	76	23	2	k__Bacteria; p__Firmicutes; c__Clostridia; o__Clostridiales; f__Lachnospiraceae
251	0	0	0	55	48	99	63	0	23	31	42	28	k__Bacteria; p__Firmicutes; c__Clostridia; o__Clostridiales; f__Ruminococcaceae; g__Anaerotruncus; s__unidentified
210	344	97	17	0	74	121	28	82	1	66	119	38	k__Bacteria; p__Firmicutes; c__Clostridia; o__Clostridiales; f__Lachnospiraceae; g__Lachnospiraceae_NK4A136_group
662	1	6	8	0	0	0	0	0	0	0	0	0	k__Bacteria; p__Proteobacteria; c__Gammaproteobacteria
621	0	0	0	0	0	0	0	0	0	0	0	1	k__Bacteria; p__Firmicutes; c__Clostridia; o__Clostridiales; f__Ruminococcaceae; g__[Eubacterium]_coprostanoligenes_group
293	0	0	0	16	7	0	0	0	0	3	16	1	k__Bacteria; p__Firmicutes; c__Clostridia; o__Clostridiales; f__Lachnospiraceae; g__Lachnospiraceae_UCG_008; s__uncultured_Clostridiales_bacterium
252	230	0	50	0	0	0	46	0	0	5	28	26	k__Bacteria; p__Firmicutes; c__Clostridia; o__Clostridiales; f__Lachnospiraceae
211	270	219	0	134	222	85	165	25	56	35	87	15	k__Bacteria; p__Bacteroidetes; c__Bacteroidia; o__Bacteroidales; f__Bacteroidales_S24_7_group; g__uncultured_bacterium; s__uncultured_bacterium
663	0	12	0	0	0	0	0	0	0	0	0	0	k__Bacteria; p__Actinobacteria; c__Nitriliruptoria; o__Nitriliruptorales; f__Nitriliruptoraceae; g__Nitriliruptor; Ambiguous_taxa
622	0	0	0	0	0	0	0	0	0	3	9	5	k__Bacteria; p__Firmicutes; c__Clostridia; o__Clostridiales; f__Lachnospiraceae

294	159	127	6	92	95	72	0	0	14	33	36	4	k__Bacteria; p__Firmicutes; c__Bacilli; o__Lactobacillales; f__Streptococcaceae; g__Streptococcus
253	0	106	0	87	53	0	0	67	39	51	28	15	k__Bacteria; p__Firmicutes; c__Clostridia; o__Clostridiales; f__Lachnospiraceae; g__Lachnospiraceae_NK4A136_group; Ambiguous_taxa
212	1	0	0	88	22	0	39	1	12	77	15	1	k__Bacteria; p__Firmicutes; c__Clostridia; o__Clostridiales; f__Lachnospiraceae
664	0	4	9	0	0	0	1	0	0	1	1	0	k__Bacteria; p__Firmicutes
623	0	0	0	0	0	0	0	0	0	10	0	2	k__Bacteria; p__Firmicutes; c__Clostridia; o__Clostridiales; f__Christensenellaceae; g__uncultured; Ambiguous_taxa
295	1	42	25	7	2	0	0	0	0	0	0	0	k__Bacteria; p__Bacteroidetes; c__Bacteroidia; o__Bacteroidia_Incertae_Sedis; f__Draconibacteriaceae; g__Draconibacterium; s__uncultured_bacterium
254	0	1	16	11	1	0	0	2	0	0	0	0	k__Bacteria; p__Proteobacteria; c__Alphaproteobacteria; o__Rhodobacterales; f__Rhodobacteraceae
213	0	146	44	0	0	81	0	0	0	26	3	4	k__Bacteria; p__Firmicutes; c__Clostridia; o__Clostridiales; f__Lachnospiraceae
665	11	17	44	0	0	0	0	0	0	0	0	0	k__Bacteria; p__Bacteroidetes; c__Sphingobacteriia; o__Sphingobacteriales; f__Saprospiraceae; g__uncultured
624	0	0	0	0	0	0	28	0	0	7	10	3	k__Bacteria; p__Firmicutes; c__Clostridia; o__Clostridiales; f__Lachnospiraceae
296	395	1652	1535	33	1	8	31	22	39	65	39	22	k__Bacteria; p__Proteobacteria; c__Epsilonproteobacteria; o__Campylobacterales; f__Campylobacteraceae; g__Arcobacter; Ambiguous_taxa
255	0	0	37	0	85	0	0	0	0	28	21	4	k__Bacteria; p__Firmicutes; c__Clostridia; o__Clostridiales; f__Lachnospiraceae; g__uncultured; s__uncultured_bacterium

214	255	0	22	25	0	69	0	53	0	0	9	12	k__Bacteria; p__Firmicutes; c__Clostridia; o__Clostridiales; f__Ruminococcaceae; g__Ruminiclostridium_5
666	0	5	9	0	0	0	0	0	0	0	0	0	k__Bacteria; p__Bacteroidetes; c__Flavobacteriia; o__Flavobacteriales; f__Flavobacteriaceae; g__Flavobacterium; s__Flavobacterium_sp._NBRC_101627
625	641	168	162	477	236	292	47	519	68	137	779	1470	k__Bacteria; p__Firmicutes; c__Clostridia; o__Clostridiales; f__Lachnospiraceae; g__Lachnospiraceae_NK4A136_group; s__uncultured_bacterium
297	103	0	0	25	0	1	0	55	0	84	7	0	k__Bacteria; p__Firmicutes; c__Clostridia; o__Clostridiales; f__Ruminococcaceae; g__Ruminococcaceae_UCG_014
215	32	78	113	12	6	1	89	60	116	119	48	14	k__Bacteria; p__Proteobacteria; c__Gammaproteobacteria; o__Vibrionales; f__Vibrionaceae; g__Vibrio
667	159	486	490	0	0	0	0	0	0	5	3	2	k__Bacteria; p__Proteobacteria; c__Epsilonproteobacteria; o__Campylobacterales; f__Campylobacteraceae; g__Arcobacter; Ambiguous_taxa
626	0	0	0	0	0	0	0	0	0	0	3	9	k__Bacteria; p__Firmicutes; c__Bacilli; o__Lactobacillales; f__Streptococcaceae; g__Streptococcus; s__Streptococcus_acidominimus
298	0	0	1	0	0	0	2	0	0	59	14	0	k__Bacteria; p__Firmicutes; c__Clostridia; o__Clostridiales; f__Ruminococcaceae; g__Ruminococcus_2; s__uncultured_bacterium
257	0	1	0	0	0	72	0	0	2	0	2	0	k__Bacteria; p__Firmicutes; c__Clostridia; o__Clostridiales; f__Lachnospiraceae; g__Lachnospiraceae_NK4A136_group
216	0	0	0	0	0	0	0	1	1	0	3	0	k__Bacteria; p__Firmicutes; c__Clostridia; o__Clostridiales; f__Ruminococcaceae; g__Subdoligranulum; Ambiguous_taxa
668	2	29	62	1	0	0	0	0	0	0	0	0	k__Bacteria; p__Proteobacteria; c__Gammaproteobacteria

627	0	0	1	0	0	0	0	0	0	0	6	3	k__Bacteria; p__Proteobacteria; c__Epsilonproteobacteria; o__Campylobacterales; f__Helicobacteraceae; g__Helicobacter; s__uncultured_bacterium
299	0	0	36	68	39	1	117	134	58	9	34	3	k__Bacteria; p__Firmicutes; c__Clostridia; o__Clostridiales; f__Lachnospiraceae
258	0	0	0	0	0	1	0	0	0	8	6	0	k__Bacteria; p__Bacteroidetes; c__Bacteroidia; o__Bacteroidales; f__Prevotellaceae
217	2	47	39	59	33	71	0	0	0	0	0	0	k__Bacteria; p__Proteobacteria; c__Deltaproteobacteria; o__Desulfobacterales; f__Desulfobulbaceae
669	0	0	1	1	0	0	0	0	0	0	0	1	k__Bacteria; p__Bacteroidetes; c__Bacteroidetes_BD2_2
628	0	0	0	0	0	0	0	83	0	6	17	1	k__Bacteria; p__Firmicutes; c__Clostridia; o__Clostridiales; f__Clostridiales_vadinBB60_group; g__uncultured_bacterium; s__uncultured_bacterium
259	196	0	108	0	27	0	22	2	0	5	16	16	k__Bacteria; p__Actinobacteria; c__Coriobacteriia; o__Coriobacteriales; f__Coriobacteriaceae; g__Enterorhabdus; s__uncultured_bacterium
218	3	65	74	145	41	47	113	171	140	9	2	2	k__Bacteria; p__Firmicutes; c__Bacilli; o__Lactobacillales; f__Carnobacteriaceae; g__Carnobacterium; Ambiguous_taxa
629	0	0	0	0	0	0	47	0	0	25	14	12	k__Bacteria; p__Firmicutes; c__Clostridia; o__Clostridiales; f__Ruminococcaceae; g__Anaerotruncus
219	0	0	0	0	0	0	31	0	25	1	0	0	k__Bacteria; p__Proteobacteria; c__Gammaproteobacteria; o__Aeromonadales; f__Succinivibrionaceae; g__Succinivibrio
1780	0	0	7	0	0	0	2	0	3	1	0	0	k__Bacteria; p__Proteobacteria; c__Deltaproteobacteria; o__Desulfobacterales; f__Desulfobulbaceae; g__Desulfobulbus
1781	0	0	0	0	0	0	0	4	5	0	0	0	k__Bacteria; p__Proteobacteria; c__Alphaproteobacteria; o__Rhodospirillales; f__Rhodospirillaceae; g__Pelagibius

1740	0	0	0	0	0	0	0	30	0	0	10	0	k__Bacteria; p__Firmicutes; c__Clostridia; o__Clostridiales; f__Clostridiales_vadinBB60_group; g__uncultured_bacterium; s__uncultured_bacterium
1371	1	0	0	0	0	0	0	0	0	0	0	0	k__Bacteria; p__Firmicutes; c__Clostridia; o__Clostridiales; f__Lachnospiraceae; g__Lachnospiraceae_NK4A136_group; Ambiguous_taxa
1330	0	0	0	0	0	0	0	0	0	1	1	0	k__Bacteria; p__Proteobacteria; c__Deltaproteobacteria; o__Oligoflexales; f__Oligoflexaceae
1782	0	0	0	0	0	0	0	0	25	0	0	0	k__Bacteria; p__Actinobacteria; c__Acidimicrobiia; o__Acidimicrobiales; f__Acidimicrobiaceae; g__CL500_29_marine_group
1741	0	0	0	0	0	0	0	79	0	0	0	0	k__Bacteria; p__Firmicutes; c__Clostridia; o__Clostridiales; f__Ruminococcaceae; g__Ruminococcaceae_UCG_010
1700	0	0	0	0	0	0	1	0	0	0	0	0	k__Bacteria; p__Proteobacteria; c__Deltaproteobacteria; o__Sva0485
1372	0	0	0	2	0	0	5	0	3	0	0	0	k__Bacteria; p__Firmicutes; c__Clostridia; o__Clostridiales; f__Family_XII; g__Fusibacter
1331	0	0	0	0	0	0	1	2	5	0	0	0	k__Bacteria; p__Proteobacteria; c__Gammaproteobacteria; o__Cellvibrionales; f__Spongiibacteraceae
1783	0	0	0	0	0	0	0	0	10	0	0	0	k__Bacteria; p__Gracilibacteria
1742	0	0	0	0	0	0	0	42	0	0	0	0	k__Bacteria; p__Bacteroidetes; c__Sphingobacteriia; o__Sphingobacteriales; f__NS11_12_marine_group; g__uncultured_Sphingobacterium_sp.; s__uncultured_Sphingobacterium_sp.
1373	0	0	0	0	0	0	0	0	0	0	8	0	k__Bacteria; p__Firmicutes; c__Clostridia; o__Clostridiales; f__Lachnospiraceae; g__Coprococcus_2

1332	0	0	0	0	0	0	0	2	3	1	0	0	k__Bacteria; p__Actinobacteria; c__Actinobacteria; o__PeM15; Ambiguous_taxa; Ambiguous_taxa; Ambiguous_taxa
1784	0	0	0	0	0	0	0	0	48	0	0	0	k__Bacteria; p__Proteobacteria; c__Alphaproteobacteria; o__Rhodospirillales; f__AT_s3_44; g__uncultured_bacterium; s__uncultured_bacterium
1743	0	0	0	0	0	0	0	16	0	0	0	0	k__Bacteria; p__Proteobacteria; c__Deltaproteobacteria; o__Desulfobacterales; f__Desulfobacteraceae; g__Desulfofrigus; Ambiguous_taxa
1702	0	5	6	0	0	0	182	119	101	0	0	0	k__Bacteria; p__Proteobacteria; c__Epsilonproteobacteria; o__Campylobacterales; f__Helicobacteraceae; g__Sulfurovum
1333	1	1	0	0	0	0	0	0	0	0	0	1	k__Bacteria; p__Gracilibacteria
1785	0	0	0	0	0	0	4	7	25	0	0	0	k__Bacteria; p__Proteobacteria; c__Gammaproteobacteria
1744	0	0	0	0	0	0	4	13	0	0	0	0	k__Bacteria; p__Acidobacteria; c__Acidobacteria; o__Subgroup_3; f__PAUC26f; Ambiguous_taxa; Ambiguous_taxa
1786	0	0	0	0	0	0	5	0	12	0	0	0	k__Bacteria; p__Proteobacteria; c__Epsilonproteobacteria; o__Campylobacterales; f__Helicobacteraceae; g__Sulfurimonas
1745	0	0	0	0	0	0	0	7	0	0	0	0	k__Bacteria; p__Spirochaetae; c__Spirochaetes; o__Spirochaetales; f__Spirochaetaceae; g__Spirochaeta_2
1704	0	0	2	0	0	0	1	18	21	0	0	0	k__Bacteria; p__Proteobacteria; c__Gammaproteobacteria; o__Alteromonadales; f__Alteromonadaceae; g__Aestuariibacter; s__uncultured_Aestuariibacter_sp.
1335	0	0	0	0	0	1	0	0	0	0	0	0	k__Bacteria; p__Proteobacteria; c__Gammaproteobacteria; o__Xanthomonadales; f__Xanthomonadales_Incertae_Sedis; g__Acidibacter; s__uncultured_proteobacterium

1787	0	0	0	0	0	0	0	0	5	0	0	0	k__Bacteria; p__Proteobacteria; c__Deltaproteobacteria; o__Desulfarculales; f__Desulfarculaceae; g__Desulfatiglans
1746	0	5	1	1	0	0	2	6	8	0	0	0	k__Bacteria; p__Proteobacteria; c__Gammaproteobacteria
1377	0	0	1	0	0	0	1	0	0	0	0	0	k__Bacteria; p__Proteobacteria; c__Deltaproteobacteria; o__Desulfovibrionales; f__Desulfovibrionaceae; g__Desulfovibrio
1336	0	0	0	0	0	1	0	0	0	0	0	0	k__Bacteria; p__Actinobacteria; c__Acidimicrobiia; o__Acidimicrobiales; f__Acidimicrobiaceae; g__CL500_29_marine_group
1788	0	0	0	0	0	0	0	0	8	0	0	0	k__Bacteria; p__Proteobacteria; c__Deltaproteobacteria; o__Desulfobacterales; f__Desulfobulbaceae
1747	0	0	0	0	0	0	0	12	1	0	0	0	k__Bacteria; p__Proteobacteria; c__Gammaproteobacteria
1706	0	0	0	0	0	0	1	1	0	0	0	0	k__Bacteria; p__Spirochaetae; c__Spirochaetes; o__Spirochaetales; f__Spirochaetaceae
1378	0	0	0	0	0	0	0	0	1	1	0	0	k__Bacteria; p__Bacteroidetes
1337	0	0	0	0	0	0	0	0	0	2	0	0	k__Bacteria; p__Firmicutes; c__Bacilli; o__Lactobacillales; f__Lactobacillaceae; g__Lactobacillus
1789	0	0	0	0	0	0	0	0	5	0	0	0	k__Bacteria; p__Bacteroidetes; c__Bacteroidia; o__Bacteroidales; f__Marinilabiaceae; g__Carboxylicivirga
1748	0	0	0	0	0	0	0	61	0	10	0	0	k__Bacteria; p__Firmicutes; c__Clostridia; o__Clostridiales; f__Lachnospiraceae; g__Oribacterium; s__uncultured_bacterium
1707	0	0	0	0	0	0	0	0	1	0	0	0	k__Bacteria; p__Proteobacteria; c__Alphaproteobacteria; o__Magnetococcales; f__Magnetococcaceae; g__Magnetococcus
1379	0	0	5	4	0	0	0	0	1	2	0	0	k__Bacteria; p__Proteobacteria; c__Gammaproteobacteria; o__Oceanospirillales; f__Oceanospirillaceae; g__Marinobacterium

1338	0	0	0	0	0	0	0	0	0	15	7	0	k__Bacteria; p__Firmicutes; c__Clostridia; o__Clostridiales; f__Lachnospiraceae
1749	0	0	0	0	0	0	0	7	0	0	0	0	k__Bacteria; p__Gracilibacteria
1708	0	0	0	0	0	0	1	0	4	0	0	0	k__Bacteria; p__Spirochaetae; c__Spirochaetes; o__Spirochaetales; f__Spirochaetaceae
1339	0	0	0	0	0	0	0	0	0	0	0	1	k__Bacteria; p__Firmicutes; c__Clostridia; o__Clostridiales; f__Ruminococcaceae; g__Ruminococcaceae_UCG_005; Ambiguous_taxa
1709	0	0	0	0	0	0	0	1	1	0	0	0	k__Bacteria; p__Bacteroidetes; c__Cytophagia; o__Cytophagales
70	321	165	39	147	271	242	26	57	193	183	113	13	k__Bacteria; p__Firmicutes; c__Clostridia; o__Clostridiales; f__Lachnospiraceae; g__Lachnospiraceae_NK4A136_group; s__uncultured_bacterium
71	59	0	0	50	54	0	0	0	0	15	31	33	k__Bacteria; p__Bacteroidetes; c__Bacteroidia; o__Bacteroidales; f__Bacteroidales_S24_7_group; g__uncultured_bacterium; s__uncultured_bacterium
590	0	0	0	0	0	6	0	0	0	0	0	0	k__Bacteria; p__Proteobacteria; c__Gammaproteobacteria; o__Cellvibrionales; f__Cellvibrionaceae; g__Saccharophagus; Ambiguous_taxa
30	0	99	58	147	75	346	0	157	0	75	42	9	k__Bacteria; p__Firmicutes; c__Clostridia; o__Clostridiales; f__Lachnospiraceae; g__uncultured; s__Lachnospiraceae_bacterium_6_1
180	0	0	0	16	0	0	4	4	1	16	8	1	k__Bacteria; p__Firmicutes; c__Clostridia; o__Clostridiales
960	1	8	6	0	0	0	0	0	0	0	0	0	k__Bacteria; p__Bacteroidetes; c__Flavobacteriia; o__Flavobacteriales; f__Cryomorphaceae; g__Salinirepens; s__uncultured_Bacteroidetes_bacterium

72	235	0	0	22	99	0	55	0	58	12	38	9	k__Bacteria; p__Bacteroidetes; c__Bacteroidia; o__Bacteroidales; f__Bacteroidales_S24_7_group; Ambiguous_taxa; Ambiguous_taxa
591	0	0	0	0	0	14	0	0	0	0	0	0	k__Bacteria; p__Proteobacteria; c__Deltaproteobacteria; o__Myxococcales; f__Sandaracinaceae; g__Sandaracinus
550	0	0	0	0	0	71	3	62	9	12	11	0	k__Bacteria; p__Bacteroidetes; c__Bacteroidia; o__Bacteroidales; f__Prevotellaceae
31	155	211	49	200	98	148	197	94	104	62	118	20	k__Bacteria; p__Bacteroidetes; c__Bacteroidia; o__Bacteroidales; f__Bacteroidales_S24_7_group; g__uncultured_bacterium; s__uncultured_bacterium
181	160	135	48	296	212	117	78	51	29	108	270	116	k__Bacteria; p__Bacteroidetes; c__Bacteroidia; o__Bacteroidales; f__Rikenellaceae; g__Alistipes; Ambiguous_taxa
140	203	388	121	241	355	298	135	110	92	64	273	109	k__Bacteria; p__Bacteroidetes; c__Bacteroidia; o__Bacteroidales; f__Bacteroidaceae; g__Bacteroides
961	1	5	9	0	0	0	0	0	0	0	0	0	k__Bacteria; p__Bacteroidetes; c__Flavobacteriia; o__Flavobacteriales; f__Cryomorphaceae; g__Crocinitomix; s__uncultured_Flavobacteriales_bacterium
920	1	39	1	0	0	0	0	0	0	0	0	0	k__Bacteria; p__Proteobacteria; c__Deltaproteobacteria; o__Desulfobacterales; f__Desulfobulbaceae
592	37	15	44	1	8	5	0	2	0	0	0	0	k__Bacteria; p__Bacteroidetes; c__Cytophagia; o__Cytophagales; f__Flammeovirgaceae; g__uncultured; s__uncultured_sediment_bacterium
551	0	130	0	0	1	86	46	0	19	15	10	2	k__Bacteria; p__Tenericutes; c__Mollicutes; o__Anaeroplasmatales; f__Anaeroplasmataceae; g__Anaeroplasma; s__uncultured_organism

510	0	0	0	0	41	0	0	0	0	0	1	1	k__Bacteria; p__Bacteroidetes; c__Bacteroidia; o__Bacteroidales; f__Bacteroidaceae; g__Bacteroides
32	0	0	0	0	54	0	0	82	17	16	23	0	k__Bacteria; p__Firmicutes; c__Clostridia; o__Clostridiales; f__Lachnospiraceae; g__Lachnospiraceae_NK4A136_group; Ambiguous_taxa
182	0	0	0	0	0	0	0	0	0	4	0	0	k__Bacteria; p__Firmicutes; c__Clostridia; o__Clostridiales; f__Ruminococcaceae; g__Ruminococcaceae_UCG_014; s__uncultured_Ruminococcaceae_bacterium
141	0	104	165	0	82	59	0	0	52	41	39	3	k__Bacteria; p__Bacteroidetes; c__Bacteroidia; o__Bacteroidales; f__Bacteroidales_S24_7_group; g__uncultured_bacterium; s__uncultured_bacterium
100	0	0	1	32	17	0	90	0	26	15	29	20	k__Bacteria; p__Bacteroidetes; c__Bacteroidia; o__Bacteroidales; f__Rikenellaceae; g__Alistipes; s__uncultured_bacterium
962	0	3	0	0	0	0	0	0	0	0	0	0	k__Bacteria; p__Bacteroidetes; c__Bacteroidia; o__Bacteroidia_Incertae_Sedis; f__Draconibacteriaceae; g__Draconibacterium
921	0	4	0	0	0	0	1	0	0	0	0	0	k__Bacteria; p__Acidobacteria; c__Holophagae; o__Acanthopleuribacterales; f__Acanthopleuribacteraceae; g__Acanthopleuribacter; s__uncultured_bacterium
74	115	0	0	36	0	63	70	0	0	14	21	7	k__Bacteria; p__Firmicutes; c__Clostridia; o__Clostridiales; f__Ruminococcaceae
593	11	7	14	1	16	4	0	0	0	0	0	0	k__Bacteria; p__Proteobacteria; c__Gammaproteobacteria; o__Pseudomonadales; f__Moraxellaceae; g__Acinetobacter
552	10	14	28	0	1	6	46	57	51	2	0	0	k__Bacteria; p__Proteobacteria; c__Deltaproteobacteria; o__Desulfobacterales; f__Desulfobacteraceae; g__uncultured

511	0	0	0	0	33	0	1	0	0	6	12	14	k__Bacteria; p__Firmicutes; c__Clostridia; o__Clostridiales; f__Lachnospiraceae
33	0	0	0	55	61	44	2	1	0	35	4	0	k__Bacteria; p__Firmicutes; c__Clostridia; o__Clostridiales; f__Ruminococcaceae; g__Ruminococcus_1
183	0	0	0	26	9	0	5	59	0	1	9	0	k__Bacteria; p__Proteobacteria; c__Epsilonproteobacteria; o__Campylobacterales; f__Campylobacteraceae; g__Campylobacter
142	0	0	0	0	0	0	0	0	0	5	0	0	k__Bacteria; p__Bacteroidetes; c__Bacteroidia; o__Bacteroidales; f__Bacteroidales_S24_7_group; g__uncultured_bacterium; s__uncultured_bacterium
101	0	0	0	0	0	0	0	0	0	9	0	0	k__Bacteria; p__Firmicutes; c__Clostridia; o__Clostridiales; f__Ruminococcaceae; g__Faecalibacterium
963	0	15	8	0	0	0	86	29	100	1	1	0	k__Bacteria; p__Fibrobacteres; c__Fibrobacteria; o__Fibrobacteria_Incertae_Sedis; f__Unknown_Family; g__possible_genus_03
922	0	10	3	0	0	0	0	0	0	0	0	0	k__Bacteria; p__Bacteroidetes; c__Bacteroidia; o__Bacteroidia_Incertae_Sedis; f__Draconibacteriaceae; g__Draconibacterium
75	24	55	140	261	91	203	0	0	0	0	0	0	k__Bacteria; p__Bacteroidetes; c__Sphingobacteriia; o__Sphingobacteriales; f__Saprospiraceae; g__uncultured; s__uncultured_bacterium
594	9	0	7	3	0	11	1	1	16	0	0	0	k__Bacteria; p__Proteobacteria; c__Gammaproteobacteria; o__Enterobacteriales; f__Enterobacteriaceae; g__Enterobacter; s__Cronobacter_pulveris
553	32	172	122	2	4	5	11	5	8	13	4	3	k__Bacteria; p__Proteobacteria; c__Gammaproteobacteria; o__Vibrionales; f__Vibrionaceae; g__Vibrio

512	0	1	5	0	12	14	1	0	0	0	0	0	k__Bacteria; p__Proteobacteria; c__Alphaproteobacteria; o__Rhodobacterales; f__Rhodobacteraceae
34	96	0	36	90	10	0	11	0	4	12	13	1	k__Bacteria; p__Bacteroidetes; c__Bacteroidia; o__Bacteroidales; f__Prevotellaceae; g__Alloprevotella; s__uncultured_bacterium
184	0	0	40	0	41	73	0	0	0	10	11	10	k__Bacteria; p__Proteobacteria; c__Epsilonproteobacteria; o__Campylobacterales; f__Helicobacteraceae; g__Helicobacter; Ambiguous_taxa
143	117	0	25	32	44	9	45	0	0	14	164	99	k__Bacteria; p__Bacteroidetes; c__Bacteroidia; o__Bacteroidales; f__Rikenellaceae; g__Alistipes
102	0	1	7	125	109	97	0	0	0	0	0	0	k__Bacteria; p__Proteobacteria; c__Gammaproteobacteria; o__Alteromonadales; f__Shewanellaceae; g__Shewanella
964	0	38	7	0	2	0	0	0	0	0	0	0	k__Bacteria; p__Proteobacteria; c__Gammaproteobacteria; o__Pseudomonadales; f__Pseudomonadaceae; g__Pseudomonas; s__Pseudomonas_stutzeri
923	0	22	0	0	0	0	0	0	0	0	0	0	k__Bacteria; p__Proteobacteria; c__Deltaproteobacteria; o__Desulfuromonadales; f__Geobacteraceae; g__Geobacter; s__uncultured_organism
76	0	47	112	6	11	10	0	0	0	1	1	0	k__Bacteria; p__Proteobacteria; c__Gammaproteobacteria; o__Oceanospirillales; f__Hahellaceae; g__Endozoicomonas
595	1	2	0	1	0	4	3	4	4	0	0	0	k__Bacteria; p__Bacteroidetes; c__Cytophagia; o__Cytophagales; f__Flammeovirgaceae; g__uncultured
554	0	0	0	0	1	98	0	0	0	27	0	0	k__Bacteria; p__Bacteroidetes; c__Bacteroidia; o__Bacteroidales; f__Prevotellaceae; g__Prevotella_1; Ambiguous_taxa
513	0	0	0	0	6	57	0	16	0	15	0	0	k__Bacteria; p__Bacteroidetes; c__Bacteroidia;

													o__Bacteroidales; f__Prevotellaceae; g__Alloprevotella
35	1310	649	584	1002	935	1127	341	609	652	1550	249	53	k__Bacteria; p__Bacteroidetes; c__Bacteroidia; o__Bacteroidales; f__Prevotellaceae; g__Prevotella_9; s__uncultured_bacterium
185	0	0	22	68	93	1	0	1	124	36	86	9	k__Bacteria; p__Firmicutes; c__Clostridia; o__Clostridiales; f__Lachnospiraceae
144	0	167	0	157	0	0	0	0	54	26	28	3	k__Bacteria; p__Firmicutes; c__Clostridia; o__Clostridiales; f__Lachnospiraceae; g__uncultured; s__unidentified
103	0	0	0	0	0	0	0	0	39	0	7	8	k__Bacteria; p__Firmicutes; c__Clostridia; o__Clostridiales; f__Ruminococcaceae; g__Ruminococcaceae_UCG_013
965	0	13	3	0	0	0	0	1	9	0	0	0	k__Bacteria; p__Proteobacteria; c__Alphaproteobacteria; o__Rhizobiales; f__Rhodobiaceae; g__Anderseniella; Ambiguous_taxa
924	1	24	0	0	0	0	0	0	0	0	0	0	k__Bacteria; p__Proteobacteria; c__Gammaproteobacteria
77	0	0	0	0	42	0	1	1	50	25	0	0	k__Bacteria; p__Bacteroidetes; c__Bacteroidia; o__Bacteroidales; f__Prevotellaceae; g__Prevotellaceae_NK3B31_group
596	0	0	0	0	4	7	0	0	0	0	0	0	k__Bacteria; p__Proteobacteria; c__Gammaproteobacteria; o__Alteromonadales; f__Colwelliaceae; g__Colwellia
555	0	0	0	0	0	83	0	0	0	6	0	4	k__Bacteria; p__Firmicutes; c__Clostridia; o__Clostridiales; f__Lachnospiraceae
514	132	0	0	1	26	0	0	0	0	6	0	0	k__Bacteria; p__Firmicutes; c__Bacilli; o__Lactobacillales; f__Streptococcaceae; g__Streptococcus
36	0	0	0	122	0	41	1	3	0	114	6	2	k__Bacteria; p__Bacteroidetes; c__Bacteroidia; o__Bacteroidales; f__Prevotellaceae; g__uncultured; s__uncultured_bacterium

186	0	0	1	21	34	0	0	83	33	3	19	10	k__Bacteria; p__Firmicutes; c__Clostridia; o__Clostridiales; f__Lachnospiraceae; g__uncultured; s__uncultured_bacterium
145	0	0	0	0	0	0	1	0	0	4	0	0	k__Bacteria; p__Firmicutes; c__Clostridia; o__Clostridiales; f__Lachnospiraceae; g__Coprococcus_1; Ambiguous_taxa
104	0	0	0	0	43	0	31	0	0	5	9	5	k__Bacteria; p__Firmicutes; c__Clostridia; o__Clostridiales; f__Ruminococcaceae; g__uncultured
966	1	22	11	0	0	1	11	4	3	0	0	0	k__Bacteria; p__Bacteroidetes; c__Sphingobacteriia; o__Sphingobacteriales; f__WCHB1_69
925	0	30	15	3	0	3	0	0	0	0	0	0	k__Bacteria; p__Acidobacteria; c__Holophagae; o__Subgroup_10; f__CA002
78	0	0	0	113	0	2	0	0	0	34	32	32	k__Bacteria; p__Firmicutes; c__Clostridia; o__Clostridiales; f__Ruminococcaceae; g__Oscillibacter; s__uncultured_bacterium
597	0	0	2	0	0	4	0	0	0	0	0	0	k__Bacteria; p__Actinobacteria; c__Acidimicrobiia; o__Acidimicrobiales; f__Sva0996_marine_group; g__uncultured_marine_bacterium; s__uncultured_marine_bacterium
556	0	0	0	0	0	54	0	0	0	0	0	0	k__Bacteria; p__Proteobacteria; c__Alphaproteobacteria; o__Rhodospirillales; f__Acetobacteraceae; g__Roseomonas; s__uncultured_bacterium
515	0	0	0	1	2	9	108	42	36	0	0	0	k__Bacteria; p__Proteobacteria; c__Epsilonproteobacteria; o__Campylobacterales; f__Helicobacteraceae; g__Sulfurimonas; Ambiguous_taxa
37	1	233	36	23	69	34	106	135	97	89	54	18	k__Bacteria; p__Firmicutes; c__Clostridia; o__Clostridiales; f__Lachnospiraceae; g__uncultured; s__uncultured_bacterium

187	173	0	0	23	2	101	0	0	63	13	55	13	k__Bacteria; p__Firmicutes; c__Clostridia; o__Clostridiales; f__Lachnospiraceae; g__Lachnospiraceae_NK4A136_group; s__uncultured_bacterium
146	0	0	0	0	0	0	0	0	0	0	6	0	k__Bacteria; p__Firmicutes; c__Clostridia; o__Clostridiales; f__Clostridiales_vadinBB60_group; g__uncultured_bacterium; s__uncultured_bacterium
105	1	1	109	0	96	111	46	22	153	21	109	134	k__Bacteria; p__Bacteroidetes; c__Bacteroidia; o__Bacteroidales; f__Bacteroidaceae; g__Bacteroides
967	0	6	6	0	0	0	0	0	0	0	0	0	k__Bacteria; p__Proteobacteria; c__Deltaproteobacteria; o__Desulfobacterales; f__Desulfobulbaceae; g__Desulfobulbus
926	0	4	0	0	0	0	0	0	0	0	0	0	k__Bacteria; p__Bacteroidetes; c__Bacteroidetes_VC2.1_Bac22; Ambiguous_taxa; Ambiguous_taxa; Ambiguous_taxa; Ambiguous_taxa
79	418	246	1	139	188	139	180	178	16	1679	50	19	k__Bacteria; p__Firmicutes; c__Clostridia; o__Clostridiales; f__Lachnospiraceae; g__Pseudobutyrivibrio; s__uncultured_bacterium
598	0	0	0	0	5	3	0	0	0	0	0	0	k__Bacteria; p__Bacteroidetes; c__Flavobacteriia; o__Flavobacteriales; f__Flavobacteriaceae; g__Sufflavibacter; s__uncultured_delta_proteobacterium
557	0	0	0	0	0	79	0	0	0	10	1	0	k__Bacteria; p__Actinobacteria; c__Actinobacteria; o__Frankiales; f__Sporichthyaceae; g__hgcI_clade; s__uncultured_bacterium
516	0	0	0	0	45	0	0	0	0	0	0	0	k__Bacteria; p__Firmicutes; c__Clostridia; o__Clostridiales; f__Clostridiales_vadinBB60_group; g__uncultured_bacterium; s__uncultured_bacterium
38	0	13	0	2	0	0	3	4	47	57	14	7	k__Bacteria; p__Firmicutes; c__Bacilli; o__Lactobacillales;

													f__Lactobacillaceae; g__Lactobacillus
188	0	121	26	36	0	36	0	0	84	16	63	35	k__Bacteria; p__Bacteroidetes; c__Bacteroidia; o__Bacteroidales; f__Bacteroidales_S24_7_group; g__uncultured_bacterium; s__uncultured_bacterium
147	0	0	1	56	1	0	44	0	2	23	0	0	k__Bacteria; p__Bacteroidetes; c__Bacteroidia; o__Bacteroidales; f__Rikenellaceae; g__Rikenellaceae_RC9_gut_group; s__uncultured_bacterium
106	395	99	114	124	350	124	25	109	176	98	228	68	k__Bacteria; p__Bacteroidetes; c__Bacteroidia; o__Bacteroidales; f__Bacteroidales_S24_7_group; g__uncultured_bacterium; s__uncultured_bacterium
968	0	10	1	0	0	0	0	0	0	0	0	0	k__Bacteria; p__Proteobacteria; c__Gammaproteobacteria; o__Xanthomonadales; f__JTB255_marine_benthic_group
927	0	7	21	0	0	0	0	0	0	0	0	0	k__Bacteria; p__Actinobacteria; c__Acidimicrobiia; o__Acidimicrobiales; f__OM1_clade
599	0	2	0	0	0	9	1	0	0	0	0	0	k__Bacteria; p__Proteobacteria; c__Deltaproteobacteria; o__Desulfobacterales; f__Desulfobulbaceae; g__Desulfobulbus
558	0	0	0	0	0	37	0	0	38	48	3	0	k__Bacteria; p__Firmicutes; c__Clostridia; o__Clostridiales; f__Lachnospiraceae
517	0	0	0	1	8	0	0	0	0	0	0	0	k__Bacteria; p__Proteobacteria; c__Gammaproteobacteria; o__Oceanospirillales; f__Hahellaceae; g__Kistimonas; Ambiguous_taxa
39	559	919	482	628	705	801	290	429	565	315	1074	443	k__Bacteria; p__Bacteroidetes; c__Bacteroidia; o__Bacteroidales; f__Bacteroidales_S24_7_group; g__uncultured_Bacteroidales_bacterium; s__uncultured_Bacteroidales_bacterium
189	0	94	1	0	17	0	0	0	0	0	10	0	k__Bacteria; p__Firmicutes; c__Clostridia; o__Clostridiales;

													f__Lachnospiraceae; g__uncultured
148	0	0	0	0	0	3	54	0	0	398	8	0	k__Bacteria; p__Bacteroidetes; c__Bacteroidia; o__Bacteroidales; f__Prevotellaceae; g__Alloprevotella; s__uncultured_bacterium
107	0	0	0	0	0	0	0	0	0	0	1	1	k__Bacteria; p__Bacteroidetes; c__Bacteroidia; o__Bacteroidales; f__Prevotellaceae; g__uncultured; s__uncultured_bacterium
969	2	48	53	0	0	0	0	2	2	0	0	0	k__Bacteria; p__Bacteroidetes; c__Flavobacteriia; o__Flavobacteriales; f__Cryomorphaceae; g__Owenweeksia
928	0	12	1	1	0	0	6	3	12	0	0	0	k__Bacteria; p__Bacteroidetes; c__Bacteroidetes_VC2.1_Bac22
559	0	0	0	0	21	27	0	0	35	9	4	7	k__Bacteria; p__Firmicutes; c__Clostridia; o__Clostridiales; f__Ruminococcaceae; g__Oscillibacter
518	0	42	0	0	19	10	0	0	0	0	5	16	k__Bacteria; p__Firmicutes; c__Clostridia; o__Clostridiales; f__Clostridiales_vadinBB60_group; g__uncultured_bacterium; s__uncultured_bacterium
149	1	161	3	53	35	0	59	0	0	0	22	0	k__Bacteria; p__Firmicutes; c__Clostridia; o__Clostridiales; f__Lachnospiraceae; g__Roseburia; s__uncultured_Clostridiales_bacterium
108	0	68	0	84	279	243	36	1	56	75	135	13	k__Bacteria; p__Firmicutes; c__Clostridia; o__Clostridiales; f__Lachnospiraceae; g__Lachnospiraceae_NK4A136_group; s__uncultured_Clostridiales_bacterium
929	0	6	17	0	0	0	0	1	1	0	0	0	k__Bacteria; p__Spirochaetae; c__Spirochaetes; o__Spirochaetales; f__Spirochaetaceae; g__Spirochaeta_2; s__uncultured_organism
519	0	0	0	0	19	0	0	24	70	8	9	2	k__Bacteria; p__Firmicutes; c__Clostridia; o__Clostridiales; f__Family_XIII; g__[Eubacterium]_nodatum_group

109	0	20	0	2	2	1	29	0	0	7	1	0	k__Bacteria; p__Proteobacteria; c__Alphaproteobacteria; o__SAR11_clade; f__LD12_freshwater_group; g__uncultured_bacterium; s__uncultured_bacterium
1671	0	0	0	0	0	0	4	1	0	0	0	0	k__Bacteria; p__Chlorobi; c__Chlorobia; o__Chlorobiales; f__OPB56
1630	0	0	0	0	0	0	1	0	0	0	0	0	k__Bacteria; p__Proteobacteria; c__Deltaproteobacteria; o__Desulfarculales; f__Desulfarculaceae; g__Desulfatiglans; Ambiguous_taxa
1261	1	4	0	3	9	37	43	0	9	43	14	1	k__Bacteria; p__Firmicutes; c__Clostridia; o__Clostridiales; f__Lachnospiraceae; g__Lachnospiraceae_NK4A136_group
1220	0	0	1	6	0	0	2	0	4	0	0	0	k__Bacteria; p__Fusobacteria; c__Fusobacteriia; o__Fusobacteriales; f__boneC3G7; g__uncultured_Fusobacterium_sp.; s__uncultured_Fusobacterium_sp.
1631	0	0	0	0	0	0	0	0	4	0	0	0	k__Bacteria; p__Bacteroidetes; c__Cytophagia; o__Cytophagales; f__Flammeovirgaceae; g__uncultured
1262	0	0	0	0	0	0	0	0	0	7	8	6	k__Bacteria; p__Firmicutes; c__Clostridia; o__Clostridiales; f__Lachnospiraceae
1221	1	0	2	0	0	0	5	0	4	0	0	0	k__Bacteria; p__Bacteroidetes; c__Flavobacteriia; o__Flavobacteriales; f__Cryomorphaceae; g__Fluviicola
1632	0	0	0	0	0	0	2	8	6	0	0	0	k__Bacteria; p__Proteobacteria; c__Deltaproteobacteria; o__Syntrophobacterales; f__Syntrophaceae; g__Desulfomonile; s__uncultured_bacterium
1263	135	0	17	2	24	0	0	0	0	42	35	91	k__Bacteria; p__Firmicutes; c__Clostridia; o__Clostridiales; f__Lachnospiraceae; g__Coprococcus_1; s__uncultured_bacterium

1222	0	0	1	2	0	0	0	0	0	0	0	0	k__Bacteria; p__Firmicutes; c__Bacilli; o__Bacillales; f__Planococcaceae; g__Lysinibacillus; Ambiguous_taxa
1674	0	0	0	0	1	0	10	12	24	0	0	0	k__Bacteria; p__Proteobacteria; c__Alphaproteobacteria; o__SAR11_clade
1264	0	0	0	0	0	0	0	0	0	28	0	0	k__Bacteria; p__Firmicutes; c__Erysipelotrichia; o__Erysipelotrichales; f__Erysipelotrichaceae; g__Erysipelotrichaceae_UCG_001; s__uncultured_bacterium
1223	0	0	7	0	0	0	10	17	4	0	0	0	k__Bacteria; p__Proteobacteria; c__Proteobacteria_Incertae_Sedis; o__Unknown_Order; f__Unknown_Family; g__Candidatus_Thiobios; Ambiguous_taxa
1675	0	0	0	0	0	0	0	0	0	8	10	0	k__Bacteria; p__Firmicutes; c__Clostridia; o__Clostridiales; f__Lachnospiraceae; g__Roseburia
1265	0	0	0	0	0	0	0	0	0	6	4	3	k__Bacteria; p__Firmicutes; c__Clostridia; o__Clostridiales; f__Ruminococcaceae; g__Anaerotruncus
1224	0	0	0	0	0	0	0	0	0	0	2	0	k__Bacteria; p__Proteobacteria; c__Deltaproteobacteria; o__Desulfovibrionales; f__Desulfovibrionaceae; g__Desulfovibrio
1676	0	0	3	0	0	0	0	0	0	0	0	0	k__Bacteria; p__Firmicutes; c__Clostridia; o__Clostridiales; f__GoM_GC232_4463_Bac1
1635	24	112	113	0	0	0	110	97	177	3	1	0	k__Bacteria; p__Proteobacteria; c__Gammaproteobacteria; o__Vibrionales; f__Vibrionaceae; g__Vibrio
1266	0	0	0	0	0	0	0	0	1	9	3	0	k__Bacteria; p__Firmicutes; c__Clostridia; o__Clostridiales; f__Ruminococcaceae; g__Anaerotruncus
1225	0	0	0	0	0	0	4	0	1	18	0	0	k__Bacteria; p__Bacteroidetes; c__Bacteroidia; o__Bacteroidales; f__Prevotellaceae; g__Alloprevotella;

													s__uncultured_bacterium
1677	0	0	0	0	0	0	9	3	4	2	0	0	k__Bacteria; p__Bacteroidetes; c__Bacteroidia; o__Bacteroidales; f__Marinilabiaceae; g__Carboxylicivirga; s__uncultured_bacterium
1267	0	0	0	0	0	0	9	4	19	3	1	0	k__Bacteria; p__Bacteroidetes; c__Flavobacteriia; o__Flavobacteriales; f__Flavobacteriaceae; g__uncultured; s__Bacteroidetes_bacterium_T4_KAD_str1
1226	0	0	0	0	0	0	0	0	0	0	5	0	k__Bacteria; p__Bacteroidetes; c__Bacteroidia; o__Bacteroidales; f__Prevotellaceae; g__Prevotellaceae_UCG_001; s__uncultured_bacterium
1637	0	0	0	0	0	0	0	0	0	7	1	0	k__Bacteria; p__Bacteroidetes; c__Bacteroidia; o__Bacteroidales; f__Prevotellaceae
1268	0	0	0	0	0	3	43	39	49	64	18	9	k__Bacteria; p__Proteobacteria; c__Gammaproteobacteria; o__Vibrionales; f__Vibrionaceae; g__Photobacterium; Ambiguous_taxa
1227	0	0	0	0	0	0	0	0	0	0	1	0	k__Bacteria; p__Firmicutes; c__Clostridia; o__Clostridiales; f__Lachnospiraceae; g__Acetatifactor; s__uncultured_bacterium
1638	0	0	0	1	2	0	4	0	0	0	0	0	k__Bacteria; p__Proteobacteria; c__Gammaproteobacteria; o__Order_Incertae_Sedis; f__Family_Incertae_Sedis; g__Marinicella; s__uncultured_bacterium
1228	0	0	0	0	0	0	0	0	0	0	1	0	k__Bacteria; p__Firmicutes; c__Bacilli; o__Bacillales; f__Planococcaceae; g__Sporosarcina; s__uncultured_bacterium
1639	0	0	0	0	0	0	0	6	2	0	0	0	k__Bacteria; p__Proteobacteria; c__Deltaproteobacteria; o__Desulfobacterales; f__Desulfobacteraceae
1229	0	0	0	1	1	0	1	0	0	1	6	7	k__Bacteria; p__Firmicutes; c__Clostridia; o__Clostridiales;

													f__Lachnospiraceae; g__uncultured; s__uncultured_bacterium
890	0	79	0	0	0	0	0	0	0	0	0	0	k__Bacteria; p__Proteobacteria; c__Alphaproteobacteria; o__Rhodospirillales; f__Rhodospirillaceae; g__uncultured; s__uncultured_soil_bacterium
480	0	0	0	1	78	0	0	0	91	0	15	10	k__Bacteria; p__Firmicutes; c__Clostridia; o__Clostridiales; f__Lachnospiraceae; g__uncultured; s__uncultured_bacterium
891	0	7	17	1	0	0	3	0	0	0	0	0	k__Bacteria; p__Proteobacteria; c__Betaproteobacteria; o__Methylophilales; f__Methylophilaceae; g__Methylotenera
850	2	2	7	0	0	0	1	2	6	1	0	0	k__Bacteria; p__Bacteroidetes; c__Bacteroidia; o__Bacteroidales; f__Marinilabiaceae; g__Carboxylicivirga; s__uncultured_bacterium
481	0	18	5	1	11	0	0	0	0	0	0	0	k__Bacteria; p__Spirochaetae; c__Spirochaetes; o__Spirochaetales; f__Spirochaetaceae; g__Spirochaeta_2; Ambiguous_taxa
440	0	0	0	10	0	0	0	0	0	0	1	0	k__Bacteria; p__Chlorobi; c__Chlorobia; o__Chlorobiales; f__OPB56
892	0	97	0	0	0	0	0	0	0	0	0	0	k__Bacteria; p__Firmicutes; c__Clostridia; o__Clostridiales; f__Lachnospiraceae
851	7	10	11	1	0	1	1	10	1	0	0	0	k__Bacteria; p__Bacteroidetes; c__Flavobacteriia; o__Flavobacteriales; f__Flavobacteriaceae; g__Pseudofulvibacter; s__uncultured_bacterium
810	12	2	1	0	0	0	0	0	0	0	0	0	k__Bacteria; p__Tenericutes; c__Mollicutes; o__Mycoplasmatales; f__Mycoplasmataceae; g__uncultured; s__uncultured_bacterium
482	0	0	0	0	23	0	0	0	0	8	5	0	k__Bacteria; p__Firmicutes; c__Clostridia; o__Clostridiales;

													f__Lachnospiraceae
441	0	0	1	17	62	132	40	0	0	13	33	2	k__Bacteria; p__Firmicutes; c__Clostridia; o__Clostridiales; f__Lachnospiraceae
400	9	38	72	13	2	12	0	11	4	0	0	0	k__Bacteria; p__Bacteroidetes; c__Bacteroidetes_BD2_2; Ambiguous_taxa; Ambiguous_taxa; Ambiguous_taxa; Ambiguous_taxa
893	0	19	0	0	0	0	1	2	0	2	0	0	k__Bacteria; p__Proteobacteria; c__Alphaproteobacteria; o__Rhodospirillales; f__Rhodospirillaceae
852	1	0	1	0	0	0	0	0	0	0	0	0	k__Bacteria; p__Proteobacteria; c__Alphaproteobacteria; o__Caulobacterales; f__Hyphomonadaceae; g__uncultured; Ambiguous_taxa
811	7	0	1	0	0	0	0	0	0	0	0	0	k__Bacteria; p__Bacteroidetes; c__Flavobacteriia; o__Flavobacteriales; f__Flavobacteriaceae; g__Tenacibaculum
483	448	99	30	0	195	82	96	198	48	51	118	51	k__Bacteria; p__Firmicutes; c__Clostridia; o__Clostridiales; f__Ruminococcaceae
442	0	0	0	9	0	0	0	0	0	0	0	0	k__Bacteria; p__Proteobacteria; c__Gammaproteobacteria; o__Aeromonadales; f__Aeromonadaceae; g__Aeromonas
401	0	0	1	42	106	1	44	0	38	55	51	2	k__Bacteria; p__Bacteroidetes; c__Bacteroidia; o__Bacteroidales; f__Rikenellaceae; g__Rikenella
894	0	44	52	1	1	0	0	0	0	0	0	0	k__Bacteria; p__Bacteroidetes; c__Sphingobacteriia; o__Sphingobacteriales; f__Saprospiraceae
853	19	51	109	5	1	0	10	1	0	1	0	0	k__Bacteria; p__Bacteroidetes; c__Sphingobacteriia; o__Sphingobacteriales; f__Saprospiraceae; g__uncultured; s__uncultured_bacterium
812	4	0	0	0	0	0	0	0	0	0	0	0	k__Bacteria; p__Proteobacteria; c__Gammaproteobacteria; o__Arenicellales; f__Arenicellaceae; g__Arenicella

484	0	0	0	0	38	0	0	0	0	0	0	0	k__Bacteria; p__Proteobacteria; c__TA18; o__uncultured_bacterium; f__uncultured_bacterium; g__uncultured_bacterium; s__uncultured_bacterium
443	0	0	0	11	4	0	0	0	0	0	0	0	k__Bacteria; p__Proteobacteria; c__Gammaproteobacteria; o__Oceanospirillales; f__Oceanospirillaceae; g__Pseudospirillum; Ambiguous_taxa
402	0	0	0	1	5	6	0	0	0	0	0	0	k__Bacteria; p__Proteobacteria; c__Gammaproteobacteria; o__Gammaproteobacteria_Incertae_Sedis; f__Unknown_Family; g__Sedimenticola
895	1	10	1	0	0	0	166	50	150	1	0	0	k__Bacteria; p__Fibrobacteres; c__Fibrobacteria; o__Fibrobacteria_Incertae_Sedis; f__Unknown_Family; g__possible_genus_03; s__uncultured_bacterium
854	8	5	10	0	0	0	0	0	0	0	0	0	k__Bacteria; p__Bacteroidetes; c__Flavobacteriia; o__Flavobacteriales; f__Cryomorphaceae; g__Owenweeksia; s__uncultured_bacterium
813	4	2	2	0	0	0	0	0	0	0	0	0	k__Bacteria; p__TA06
485	0	0	0	0	52	0	1	0	0	11	0	3	k__Bacteria; p__Firmicutes; c__Clostridia; o__Clostridiales; f__Ruminococcaceae; g__Ruminococcus_1
444	261	0	84	12	30	225	90	35	76	79	49	6	k__Bacteria; p__Firmicutes; c__Clostridia; o__Clostridiales; f__Lachnospiraceae; g__Lachnospiraceae_NK4A136_group
403	0	0	3	7	0	1	0	0	0	0	0	0	k__Bacteria; p__Proteobacteria; c__Alphaproteobacteria; o__Rhizobiales
896	0	7	4	0	0	0	0	0	0	0	0	0	k__Bacteria; p__Proteobacteria; c__Deltaproteobacteria; o__Desulfuromonadales; f__GR_WP33_58
855	1	51	26	2	0	0	0	0	0	0	0	0	k__Bacteria; p__Bacteroidetes; c__Flavobacteriia; o__Flavobacteriales; f__Flavobacteriaceae;

													g__NS3a_marine_group
814	21	1	19	0	0	7	0	0	0	0	0	0	k__Bacteria; p__Proteobacteria; c__Gammaproteobacteria; o__Cellvibrionales; f__Halieaceae; Ambiguous_taxa; Ambiguous_taxa
486	0	0	35	0	24	0	0	0	0	6	5	4	k__Bacteria; p__Firmicutes; c__Erysipelotrichia; o__Erysipelotrichales; f__Erysipelotrichaceae; g__Erysipelatoclostridium; s__uncultured_bacterium
445	1	20	2	4	4	0	0	0	0	0	0	0	k__Bacteria; p__Bacteroidetes; c__Sphingobacteriia; o__Sphingobacteriales; f__Saprospiraceae
404	25	120	157	36	3	11	0	0	1	0	0	0	k__Bacteria; p__Proteobacteria; c__Gammaproteobacteria; o__Thiotrichales; f__Thiotrichaceae; g__uncultured
897	0	68	0	0	0	0	0	0	0	0	0	0	k__Bacteria; p__Actinobacteria; c__Actinobacteria
856	28	0	0	0	0	1	0	0	0	0	0	0	k__Bacteria; p__Proteobacteria; c__Deltaproteobacteria; o__Sh765B_TzT_29
815	8	0	0	0	0	0	0	1	0	0	0	0	k__Bacteria; p__Actinobacteria; c__Actinobacteria; o__Micrococcales; f__Intrasporangiaceae; g__Terrabacter; Ambiguous_taxa
487	0	0	0	1	164	0	0	77	0	23	23	7	k__Bacteria; p__Firmicutes; c__Clostridia; o__Clostridiales; f__Lachnospiraceae; g__Lachnospiraceae_UCG_001; s__uncultured_bacterium
446	0	4	9	9	1	1	0	0	0	0	0	0	k__Bacteria; p__Proteobacteria; c__Gammaproteobacteria; o__HOC36; f__uncultured_gamma_proteobacterium; g__uncultured_gamma_proteobacterium; s__uncultured_gamma_proteobacterium
405	0	0	0	20	4	2	23	8	15	0	0	0	k__Bacteria; p__Proteobacteria; c__Epsilonproteobacteria;

													o__Campylobacterales; f__Helicobacteraceae; g__Sulfurimonas
898	0	5	2	0	0	0	0	0	0	0	0	0	k__Bacteria; p__Bacteroidetes; c__Flavobacteriia; o__Flavobacteriales; f__Cryomorphaceae; g__Owenweeksia; Ambiguous_taxa
857	2	0	0	0	0	0	0	0	0	0	0	0	k__Bacteria; p__Proteobacteria; c__Gammaproteobacteria; o__Alteromonadales; f__Alteromonadaceae; g__Marinobacter; Ambiguous_taxa
816	36	94	169	0	1	0	0	0	0	0	0	0	k__Bacteria; p__Proteobacteria; c__Alphaproteobacteria; o__Rhodobacterales; f__Rhodobacteraceae; g__Shimia; Ambiguous_taxa
488	0	0	0	21	20	0	0	72	0	10	14	7	k__Bacteria; p__Firmicutes; c__Clostridia; o__Clostridiales; f__Lachnospiraceae; g__Lachnospiraceae_NK4A136_group; s__uncultured_bacterium
447	0	0	0	2	0	1	0	0	3	0	0	0	k__Bacteria; p__Proteobacteria; c__Gammaproteobacteria
406	0	4	7	11	13	9	0	0	0	0	0	0	k__Bacteria; p__Proteobacteria; c__Deltaproteobacteria; o__Desulfobacterales; f__Desulfobulbaceae
899	0	86	0	0	0	0	0	0	0	0	0	0	k__Bacteria; p__Proteobacteria; c__Betaproteobacteria; o__Nitrosomonadales; f__Nitrosomonadaceae; g__uncultured; s__uncultured_Burkholderiaceae_bacterium
858	3	12	27	0	0	0	0	0	0	0	0	0	k__Bacteria; p__Proteobacteria; c__Alphaproteobacteria
817	3	25	19	6	0	0	0	0	0	0	0	0	k__Bacteria; p__Gemmatimonadetes; c__Gemmatimonadetes; o__PAUC43f_marine_benthic_group; Ambiguous_taxa; Ambiguous_taxa; Ambiguous_taxa
489	0	0	0	4	8	8	4	9	16	1	0	0	k__Bacteria; p__Proteobacteria; c__Gammaproteobacteria; o__Gammaproteobacteria_Incertae_Sedis; f__Unknown_Family;

													g__Thiohalophilus; Ambiguous_taxa
448	0	1	0	1	0	1	0	0	0	0	0	0	k__Bacteria; p__Bacteroidetes; c__Flavobacteriia; o__Flavobacteriales; f__Flavobacteriaceae
407	2	14	54	5	2	9	106	62	105	1	0	0	k__Bacteria; p__Proteobacteria; c__Gammaproteobacteria; o__Thiotrichales; f__Thiotrichaceae; g__uncultured
859	17	0	0	0	0	0	0	0	0	0	0	0	k__Bacteria; p__Actinobacteria; c__Thermoleophilia; Ambiguous_taxa; Ambiguous_taxa; Ambiguous_taxa; Ambiguous_taxa
818	1	0	3	0	0	0	0	0	0	0	0	0	k__Bacteria; p__Proteobacteria; c__Deltaproteobacteria; o__Desulfobacterales; f__Desulfobacteraceae
449	1	0	1	15	6	2	0	0	0	0	0	0	k__Bacteria; p__Proteobacteria; c__Deltaproteobacteria; o__Desulfobacterales; f__Desulfobulbaceae; g__Desulfopila; s__uncultured_bacterium
408	0	5	47	9	0	5	11	1	7	1	0	0	k__Bacteria; p__Proteobacteria; c__Gammaproteobacteria
819	14	3	24	1	0	0	0	0	0	0	0	0	k__Bacteria; p__Proteobacteria; c__Deltaproteobacteria; o__Desulfobacterales; f__Desulfobulbaceae; g__Desulforhopalus; s__uncultured_bacterium
409	0	0	0	40	0	0	0	0	0	0	4	3	k__Bacteria; p__Firmicutes; c__Clostridia; o__Clostridiales; f__Lachnospiraceae
1190	0	0	14	0	0	0	0	0	0	0	0	0	k__Bacteria; p__Proteobacteria; c__Gammaproteobacteria; o__Pseudomonadales; f__Moraxellaceae; g__Acinetobacter
1560	0	0	0	0	0	0	9	0	1	0	0	0	k__Bacteria; p__Proteobacteria; c__Alphaproteobacteria; o__Rhodospirillales; f__Rhodospirillaceae; g__Defluviicoccus; s__uncultured_bacterium
1191	0	0	38	0	0	0	0	0	0	0	0	0	k__Bacteria; p__Bacteroidetes; c__Bacteroidia;

													o__Bacteroidales; f__Bacteroidales_S24_7_group
1150	0	0	9	0	0	0	0	0	0	0	0	0	k__Bacteria; p__Proteobacteria; c__Deltaproteobacteria; o__Sh765B_TzT_29; f__uncultured_bacterium; g__uncultured_bacterium; s__uncultured_bacterium
1561	0	0	0	0	0	0	11	0	0	0	0	0	k__Bacteria; p__Chlorobi; c__Chlorobia; o__Chlorobiales; f__OPB56; g__uncultured_sediment_bacterium; s__uncultured_sediment_bacterium
1520	0	0	0	0	0	0	76	54	78	0	0	0	k__Bacteria; p__Proteobacteria; c__Deltaproteobacteria; o__Desulfobacterales; f__Desulfobacteraceae
1192	0	0	11	0	0	0	0	0	0	0	0	0	k__Bacteria; p__Firmicutes; c__Clostridia; o__Clostridiales; f__Defluviitaleaceae; g__Defluviitaleaceae_UCG_011
1151	0	0	3	0	0	0	0	0	0	0	0	0	k__Bacteria; p__Gracilibacteria
1110	0	5	18	0	0	0	3	0	0	0	0	0	k__Bacteria; p__Proteobacteria; c__Gammaproteobacteria; o__Alteromonadales; f__Pseudoalteromonadaceae; g__Pseudoalteromonas; s__Pseudoalteromonas_ruthenica
1562	0	0	0	0	0	0	3	0	8	0	0	0	k__Bacteria; p__Chloroflexi; c__Dehalococcoidia
1521	0	0	0	0	0	0	27	36	43	0	0	0	k__Bacteria; p__Proteobacteria; c__Epsilonproteobacteria; o__Campylobacterales; f__Campylobacteraceae; g__Arcobacter
1193	0	0	10	0	0	0	0	0	0	0	0	0	k__Bacteria; p__Proteobacteria
1152	0	0	3	0	0	0	0	0	0	0	0	0	k__Bacteria; p__Bacteroidetes; c__Cytophagia; o__Cytophagales; f__Flammeovirgaceae
1111	0	2	2	0	1	0	0	0	0	0	0	0	k__Bacteria; p__Bacteroidetes; c__Cytophagia; o__Cytophagales; f__Flammeovirgaceae; g__Marinoscillum
1563	0	0	0	0	0	0	4	0	0	0	0	0	k__Bacteria; p__Proteobacteria; c__Alphaproteobacteria; o__DB1_14; f__uncultured_organism; g__uncultured_organism; s__uncultured_organism

1522	0	0	0	0	0	0	24	4	1	0	0	0	k__Bacteria; p__TM6
1194	0	0	2	0	1	0	0	0	3	0	0	0	k__Bacteria; p__Proteobacteria; c__Alphaproteobacteria; o__Rhodospirillales; f__Rhodospirillaceae
1153	0	0	20	0	0	0	0	0	9	0	0	0	k__Bacteria; p__Proteobacteria; c__Gammaproteobacteria; o__Oceanospirillales; f__Alcanivoracaceae; g__Kangiella; s__uncultured_gamma_proteobacterium
1112	0	0	18	0	0	0	0	0	0	0	0	0	k__Bacteria; p__Bacteroidetes; c__Sphingobacteriia; o__Sphingobacteriales; f__Saprospiraceae; g__uncultured
1564	0	0	11	0	0	0	23	21	28	0	0	0	k__Bacteria; p__Cyanobacteria; c__Cyanobacteria; o__SubsectionI; f__FamilyI; g__Synechococcus
1523	0	0	2	0	0	0	6	9	0	0	0	0	k__Bacteria; p__Proteobacteria; c__Gammaproteobacteria; o__Cellvibrionales; f__Cellvibrionaceae; g__Umboniibacter; Ambiguous_taxa
1195	0	0	5	0	0	0	2	3	3	0	0	0	k__Bacteria; p__Proteobacteria; c__Epsilonproteobacteria; o__Campylobacterales; f__Campylobacteraceae; g__Arcobacter
1154	0	0	4	0	0	0	0	0	0	0	0	0	k__Bacteria; p__Proteobacteria; c__Deltaproteobacteria; o__Bdellovibrionales; f__Bacteriovoracaceae
1113	4	0	7	0	0	0	0	0	0	0	0	0	k__Bacteria; p__Bacteroidetes; c__Cytophagia; o__Cytophagales; f__Flammeovirgaceae
1565	0	0	0	0	0	9	27	1	0	0	0	0	k__Bacteria; p__Firmicutes; c__Clostridia; o__Clostridiales; f__Clostridiaceae_1
1524	0	0	0	0	0	0	17	13	3	0	0	0	k__Bacteria; p__Bacteroidetes; c__Bacteroidia; o__Bacteroidia_Incertae_Sedis; f__Draconibacteriaceae; g__Draconibacterium
1196	0	0	6	0	0	0	0	0	0	0	0	0	k__Bacteria; p__Proteobacteria; c__Gammaproteobacteria; o__Thiotrichales; f__Piscirickettsiaceae; g__endosymbionts

1155	2	4	20	0	0	0	28	19	15	0	0	0	k__Bacteria; p__Proteobacteria; c__Proteobacteria_Incertae_Sedis; o__Unknown_Order; f__Unknown_Family; g__Candidatus_Thiobios
1114	0	0	9	0	0	0	0	0	0	0	0	0	k__Bacteria; p__Proteobacteria; c__Deltaproteobacteria; o__Myxococcales; f__UASB_TL25; g__uncultured_bacterium; s__uncultured_bacterium
1566	0	0	0	0	0	0	48	5	1	0	0	0	k__Bacteria; p__Proteobacteria; c__Gammaproteobacteria; o__Alteromonadales; f__Alteromonadaceae; g__Catenovulum; Ambiguous_taxa
1525	0	0	1	0	1	0	17	0	13	0	1	0	k__Bacteria; p__Proteobacteria; c__Deltaproteobacteria; o__Desulfuromonadales; f__Geobacteraceae; g__Geothermobacter
1197	0	0	10	0	0	0	0	0	0	0	0	0	k__Bacteria; p__Proteobacteria; c__Deltaproteobacteria; o__Desulfobacterales; f__Desulfobacteraceae; g__Desulfatitalea
1156	0	0	5	0	0	0	19	0	0	0	0	0	k__Bacteria; p__Proteobacteria; c__Deltaproteobacteria; o__Desulfobacterales; f__Desulfobacteraceae
1115	0	0	24	0	0	0	0	0	0	2	0	0	k__Bacteria; p__Proteobacteria; c__Betaproteobacteria; o__Burkholderiales; f__Comamonadaceae
1567	0	0	0	0	0	0	28	1	4	0	0	0	k__Bacteria; p__Proteobacteria; c__Deltaproteobacteria; o__Desulfuromonadales; f__GR_WP33_58
1526	0	0	0	0	0	0	16	11	26	1	0	0	k__Bacteria; p__Bacteroidetes; c__Bacteroidia; o__Bacteroidales; f__Marinilabiaceae; g__Marinifilum
1198	0	1	12	0	0	0	0	1	1	0	1	0	k__Bacteria; p__Spirochaetae; c__Spirochaetes; o__Spirochaetales; f__Spirochaetaceae; g__Spirochaeta_2
1157	3	0	10	0	0	0	0	0	0	0	0	0	k__Bacteria; p__Bacteroidetes; c__Sphingobacteriia; o__Sphingobacteriales; f__Saprospiraceae;

													g__Phaeodactylibacter; s__uncultured_Bacteroidetes_bacterium
1116	0	0	11	0	0	0	0	0	0	0	0	0	k__Bacteria; p__Bacteroidetes; c__Sphingobacteriia; o__Sphingobacteriales; f__Chitinophagaceae; g__Ferruginibacter; Ambiguous_taxa
1568	0	0	3	0	3	0	40	13	28	0	0	0	k__Bacteria; p__Proteobacteria; c__Gammaproteobacteria
1527	0	0	0	0	0	0	11	0	1	0	0	0	k__Bacteria; p__Proteobacteria; c__Deltaproteobacteria; o__Bdellovibrionales; f__Bacteriovoracaceae; g__Peredibacter
1199	0	0	13	0	0	0	1	0	0	0	0	0	k__Bacteria; p__Proteobacteria; c__Deltaproteobacteria; o__Myxococcales; f__UASB_TL25
1158	1	1	13	0	0	0	0	0	0	0	0	0	k__Bacteria; p__Bacteroidetes
1117	0	1	10	0	0	0	0	0	0	0	0	0	k__Bacteria; p__Proteobacteria; c__Deltaproteobacteria; o__Myxococcales; f__PS_B29; g__uncultured_organism; s__uncultured_organism
1569	0	0	3	0	0	0	16	0	0	0	0	0	k__Bacteria; p__Bacteroidetes; c__Cytophagia; o__Order_II; f__Rhodothermaceae; g__uncultured; Ambiguous_taxa
1528	0	0	0	0	0	0	5	7	6	0	0	0	k__Bacteria; p__Proteobacteria; c__Gammaproteobacteria; o__Chromatiales; f__Ectothiorhodospiraceae; g__Thioalkalispira
1159	0	0	5	0	0	0	0	0	0	0	0	0	k__Bacteria
1118	0	0	4	0	0	0	0	0	0	0	0	0	k__Bacteria; p__Proteobacteria; c__Alphaproteobacteria; o__Rickettsiales; f__uncultured; g__uncultured_bacterium; s__uncultured_bacterium
1529	0	0	4	0	0	0	6	1	0	0	0	0	k__Bacteria; p__Tenericutes; c__Mollicutes; o__NB1_n
1119	0	0	5	0	0	0	0	0	0	0	0	0	k__Bacteria; p__Acidobacteria; c__Subgroup_22
780	77	0	0	0	0	0	0	0	0	0	4	0	k__Bacteria; p__Firmicutes; c__Erysipelotrichia; o__Erysipelotrichales; f__Erysipelotrichaceae;

													g__uncultured_bacterium; s__uncultured_bacterium
370	0	61	0	84	2	0	0	0	0	41	16	20	k__Bacteria; p__Firmicutes; c__Clostridia; o__Clostridiales; f__Ruminococcaceae; g__Intestinimonas; s__uncultured_bacterium
781	6	0	5	0	0	1	0	0	0	0	0	0	k__Bacteria; p__Bacteroidetes; c__Sphingobacteriia; o__Sphingobacteriales; f__Saprospiraceae; g__uncultured
740	0	23	0	0	0	0	0	0	0	0	0	0	k__Bacteria; p__Bacteroidetes; c__Cytophagia; o__Order_III; f__uncultured; Ambiguous_taxa; Ambiguous_taxa
371	0	15	0	4	0	0	0	0	0	0	0	0	k__Bacteria; p__Bacteroidetes
330	303	292	32	189	116	74	44	0	153	33	52	7	k__Bacteria; p__Firmicutes; c__Clostridia; o__Clostridiales; f__Lachnospiraceae; g__Lachnospiraceae_UCG_001; s__uncultured_bacterium
782	7	11	10	0	0	0	0	0	0	0	0	0	k__Bacteria; p__Bacteroidetes; c__Flavobacteriia; o__Flavobacteriales; f__Flavobacteriaceae; g__Flavobacterium
741	1	15	37	3	0	4	1	14	5	0	0	0	k__Bacteria; p__Fibrobacteres; c__Fibrobacteria; o__Fibrobacteria_Incertae_Sedis; f__Unknown_Family; g__possible_genus_03; s__uncultured_bacterium
372	152	152	42	62	56	88	59	117	66	860	123	48	k__Bacteria; p__Firmicutes; c__Clostridia; o__Clostridiales; f__Lachnospiraceae; g__Lachnospiraceae_NK4A136_group
331	0	4	33	37	41	1	0	89	46	1	29	3	k__Bacteria; p__Firmicutes; c__Clostridia; o__Clostridiales; f__Lachnospiraceae; g__uncultured; s__unidentified
783	30	6	23	0	0	0	8	0	0	0	0	0	k__Bacteria; p__Acidobacteria; c__Holophagae; o__Subgroup_10; f__Sva0725; Ambiguous_taxa; Ambiguous_taxa
742	0	12	28	1	0	1	12	9	2	5	2	1	k__Bacteria; p__Proteobacteria; c__Gammaproteobacteria;

													o__Oceanospirillales; f__Oceanospirillaceae; g__Neptuniibacter; Ambiguous_taxa
701	33	72	183	1	0	0	1	2	4	0	0	0	k__Bacteria; p__Proteobacteria; c__Deltaproteobacteria; o__Desulfobacterales; f__Desulfobulbaceae; g__uncultured; Ambiguous_taxa
373	109	0	0	76	0	0	0	0	0	26	2	0	k__Bacteria; p__Firmicutes; c__Clostridia; o__Clostridiales; f__Lachnospiraceae; g__Dorea; s__uncultured_bacterium
332	1	40	39	46	20	9	9	2	6	1	0	2	k__Bacteria; p__Bacteroidetes; c__Cytophagia; o__Cytophagales; f__Flammeovirgaceae; g__Reichenbachiella; Ambiguous_taxa
784	7	2	5	0	1	4	2	8	2	0	0	0	k__Bacteria; p__Proteobacteria; c__Alphaproteobacteria; o__Rhodobacterales; f__Rhodobacteraceae; g__Thioclava
743	0	6	2	0	0	0	0	0	0	0	0	0	k__Bacteria; p__Proteobacteria; c__Gammaproteobacteria; o__Cellvibrionales; f__Halieaceae; g__Haliea
702	0	0	6	0	0	0	0	0	0	0	0	0	k__Bacteria; p__Proteobacteria; c__Gammaproteobacteria; o__Oceanospirillales; f__Halomonadaceae
374	27	14	31	18	1	14	0	1	2	0	0	0	k__Bacteria; p__Proteobacteria; c__Alphaproteobacteria; o__SAR11_clade; f__Surface_1; g__Candidatus_Pelagibacter; s__uncultured_bacterium
333	0	0	0	57	0	0	0	0	0	0	0	0	k__Bacteria; p__Bacteroidetes; c__Bacteroidia; o__Bacteroidales; f__Rikenellaceae; g__Alistipes; s__uncultured_bacterium
785	70	0	0	0	0	0	46	0	0	7	3	3	k__Bacteria; p__Firmicutes; c__Bacilli; o__Lactobacillales; f__Lactobacillaceae; g__Lactobacillus
744	0	5	15	0	0	0	0	0	0	0	1	0	k__Bacteria; p__Proteobacteria; c__Alphaproteobacteria; o__Rhodospirillales; f__Rhodospirillaceae; g__uncultured

703	0	1	11	0	0	0	0	0	0	0	0	0	k__Bacteria; p__Proteobacteria; c__Epsilonproteobacteria; o__Campylobacterales; f__Campylobacteraceae; g__Arcobacter
375	0	0	0	16	0	66	0	0	48	4	57	116	k__Bacteria; p__Firmicutes; c__Clostridia; o__Clostridiales; f__Lachnospiraceae; g__Roseburia
334	0	0	0	33	0	0	0	0	0	9	3	6	k__Bacteria; p__Firmicutes; c__Clostridia; o__Clostridiales; f__Ruminococcaceae; g__Ruminiclostridium_5; s__uncultured_Clostridiales_bacterium
786	6	1	0	0	0	0	41	26	20	0	0	0	k__Bacteria; p__Proteobacteria; c__Gammaproteobacteria; o__Thiotrichales; f__Thiotrichaceae; g__uncultured
745	18	67	101	0	0	0	0	0	0	0	0	0	k__Bacteria; p__Bacteroidetes; c__Flavobacteriia; o__Flavobacteriales; f__Cryomorphaceae; g__Fluviicola; s__unidentified_marine_bacterioplankton
704	1	26	42	0	0	0	0	0	2	3	0	0	k__Bacteria; p__Firmicutes; c__Clostridia; o__Clostridiales
376	491	0	44	42	15	73	46	45	68	8	40	47	k__Bacteria; p__Firmicutes; c__Clostridia; o__Clostridiales; f__Lachnospiraceae; g__Blautia; s__Lachnospiraceae_bacterium_615
335	0	0	0	48	0	0	0	0	0	0	1	0	k__Bacteria; p__Firmicutes; c__Clostridia; o__Clostridiales; f__Family_XIII; g__Family_XIII_UCG_001; s__uncultured_bacterium
787	34	7	23	1	2	0	0	0	0	0	0	0	k__Bacteria; p__Bacteroidetes; c__Flavobacteriia; o__Flavobacteriales; f__Flavobacteriaceae
746	10	47	29	0	0	4	3	5	5	0	0	0	k__Bacteria; p__Bacteroidetes; c__Flavobacteriia; o__Flavobacteriales; f__Cryomorphaceae
705	40	68	68	0	0	0	0	0	0	0	0	0	k__Bacteria; p__Proteobacteria; c__Gammaproteobacteria; o__Oceanospirillales; f__Oceanospirillaceae; g__Marinobacterium

377	0	0	1	49	0	1	18	0	35	5	42	4	k__Bacteria; p__Firmicutes; c__Clostridia; o__Clostridiales; f__Clostridiales_vadinBB60_group; g__uncultured_bacterium; s__uncultured_bacterium
336	0	0	0	10	0	0	0	0	0	0	0	0	k__Bacteria; p__Bacteroidetes; c__Sphingobacteriia; o__Sphingobacteriales; f__Saprospiraceae; g__uncultured
788	4	0	2	0	0	0	0	0	0	0	0	0	k__Bacteria; p__Proteobacteria; c__Alphaproteobacteria; o__Sphingomonadales; f__Sphingomonadaceae
747	1	8	14	0	0	0	0	0	0	0	0	0	k__Bacteria; p__Bacteroidetes; c__Sphingobacteriia; o__Sphingobacteriales; f__Saprospiraceae; g__Lewinella
706	7	13	82	1	0	2	1	2	2	0	0	0	k__Bacteria; p__Actinobacteria; c__Acidimicrobiia; o__Acidimicrobiales; f__Acidimicrobiaceae; g__Illumatobacter; Ambiguous_taxa
378	0	0	0	37	21	0	0	59	0	0	17	0	k__Bacteria; p__Bacteroidetes; c__Bacteroidia; o__Bacteroidales; f__Rikenellaceae; g__Alistipes; s__uncultured_bacterium
337	0	14	21	11	5	0	11	8	6	11	7	4	k__Bacteria; p__Proteobacteria; c__Epsilonproteobacteria; o__Campylobacterales; f__Campylobacteraceae; g__Arcobacter
789	163	0	0	0	0	0	0	0	0	0	0	0	k__Bacteria; p__Actinobacteria; c__Coriobacteriia; o__Coriobacteriales; f__Coriobacteriaceae; g__Enterorhabdus; s__uncultured_bacterium
748	0	1	2	0	0	0	0	0	0	0	0	0	k__Bacteria; p__Bacteroidetes; c__Cytophagia; o__Order_III; f__Unknown_Family; g__Balneola; Ambiguous_taxa
707	10	11	2	4	0	0	2	0	1	0	0	0	k__Bacteria; p__Bacteroidetes; c__Sphingobacteriia; o__Sphingobacteriales; f__Saprospiraceae; g__uncultured; s__uncultured_Bacteroidetes_bacterium
379	0	0	0	8	0	0	0	1	1	0	0	0	k__Bacteria; p__Proteobacteria; c__Gammaproteobacteria;

													o__Oceanospirillales; f__Oceanospirillaceae
338	0	0	1	3	1	0	0	0	0	0	0	0	k__Bacteria; p__Proteobacteria; c__Gammaproteobacteria; o__Oceanospirillales; f__Alcanivoracaceae; g__Kangiella
749	0	7	11	1	0	0	0	0	0	0	0	0	k__Bacteria; p__Bacteroidetes; c__Bacteroidetes_BD2_2
708	4	20	30	1	0	1	0	0	0	0	0	0	k__Bacteria; p__Bacteroidetes; c__Flavobacteriia; o__Flavobacteriales; f__Flavobacteriaceae; g__Aquibacter
339	1	11	30	6	3	0	10	0	2	0	0	0	k__Bacteria; p__Bacteroidetes; c__Flavobacteriia; o__Flavobacteriales; f__Flavobacteriaceae; g__Lutibacter; Ambiguous_taxa
709	1	25	22	6	0	0	0	0	0	1	0	0	k__Bacteria; p__Bacteroidetes; c__Flavobacteriia; o__Flavobacteriales; f__Flavobacteriaceae; g__Lutibacter; Ambiguous_taxa
1490	0	0	0	0	0	0	18	0	3	0	0	0	k__Bacteria; p__Proteobacteria; c__Deltaproteobacteria; o__Myxococcales; f__Nannocystaceae; g__Pseudenhygromyxa
1080	0	0	43	0	0	0	0	0	0	0	14	6	k__Bacteria; p__Firmicutes; c__Clostridia; o__Clostridiales; f__Lachnospiraceae; g__Lachnospiraceae_UCG_001; s__uncultured_bacterium
1491	0	0	0	0	0	0	5	1	23	0	0	0	k__Bacteria; p__Bacteroidetes; c__Bacteroidia; o__Bacteroidales; f__Marinilabiaceae; g__uncultured; s__uncultured_Bacteroidetes_bacterium
1450	0	0	0	0	0	0	0	0	0	12	0	0	k__Bacteria; p__Actinobacteria; c__Coriobacteriia; o__Coriobacteriales; f__Coriobacteriaceae
1081	0	0	2	0	0	0	0	0	0	0	0	0	k__Bacteria; p__Bacteroidetes; c__Sphingobacteriia; o__Sphingobacteriales; f__Saprospiraceae; g__Portibacter; s__uncultured_Bacteroidetes_bacterium
1040	0	8	10	0	0	0	0	0	0	0	0	0	k__Bacteria; p__Proteobacteria; c__Alphaproteobacteria;

													o__Rhodobacterales; f__Rhodobacteraceae
1492	0	0	0	0	0	0	5	0	0	0	0	0	k__Bacteria; p__Bacteroidetes; c__Sphingobacteriia; o__Sphingobacteriales; f__Chitinophagaceae; g__Sediminibacterium; Ambiguous_taxa
1451	0	0	0	0	0	0	0	0	0	3	3	0	k__Bacteria; p__Firmicutes; c__Clostridia; o__Clostridiales; f__Ruminococcaceae; g__Subdoligranulum; s__uncultured_bacterium
1410	0	0	0	0	0	0	0	0	1	169	0	0	k__Bacteria; p__Firmicutes; c__Erysipelotrichia; o__Erysipelotrichales; f__Erysipelotrichaceae; g__Solobacterium
1082	0	9	26	0	0	0	0	0	0	0	0	0	k__Bacteria; p__Gracilibacteria
1041	4	33	22	0	1	1	3	2	0	0	1	0	k__Bacteria; p__Proteobacteria; c__Gammaproteobacteria; o__Alteromonadales; f__Alteromonadaceae
1000	0	5	9	0	0	0	0	0	0	0	0	0	k__Bacteria; p__Gemmatimonadetes; c__Gemmatimonadetes; o__PAUC43f_marine_benthic_group; Ambiguous_taxa; Ambiguous_taxa; Ambiguous_taxa
1493	0	0	0	0	1	0	186	88	153	0	0	0	k__Bacteria; p__Proteobacteria; c__Epsilonproteobacteria; o__Campylobacterales; f__Helicobacteraceae; g__Sulfurimonas; s__uncultured_epsilon_proteobacterium
1452	0	0	0	0	0	0	0	0	0	14	0	0	k__Bacteria; p__Firmicutes; c__Clostridia; o__Clostridiales; f__Lachnospiraceae; g__Lachnospiraceae_ND3007_group; s__uncultured_bacterium
1411	0	0	0	0	0	0	0	0	0	37	0	0	k__Bacteria; p__Bacteroidetes; c__Bacteroidia; o__Bacteroidales; f__Prevotellaceae; g__Prevotella_1; s__uncultured_bacterium
1083	0	7	49	0	0	0	0	0	3	0	0	0	k__Bacteria; p__Proteobacteria; c__Alphaproteobacteria;

													o__Sphingomonadales; f__Erythrobacteraceae
1042	0	10	14	0	0	0	5	1	1	2	0	0	k__Bacteria; p__Proteobacteria; c__Gammaproteobacteria; o__Alteromonadales; f__Colwelliaceae; g__Thalassotalea; s__uncultured_bacterium
1001	0	14	5	0	0	0	0	0	0	0	0	0	k__Bacteria; p__Proteobacteria; c__Gammaproteobacteria
1494	0	1	3	0	0	0	4	0	0	0	0	0	k__Bacteria; p__Proteobacteria; c__Gammaproteobacteria; o__Oceanospirillales; f__Alcanivoracaceae; g__Kangiella
1453	0	0	0	0	0	0	0	0	1	29	5	0	k__Bacteria; p__Firmicutes; c__Clostridia; o__Clostridiales; f__Ruminococcaceae; g__Oscillibacter
1412	0	0	0	0	0	0	0	0	0	19	0	0	k__Bacteria; p__Firmicutes; c__Clostridia; o__Clostridiales; f__Ruminococcaceae; g__[Eubacterium]_coprostanoligenes_group
1084	2	0	13	0	0	0	0	0	0	1	0	0	k__Bacteria; p__Proteobacteria; c__Gammaproteobacteria; o__Cellvibrionales; f__Halieaceae
1043	0	11	8	1	0	0	0	0	0	0	0	0	k__Bacteria; p__Bacteroidetes; c__Flavobacteriia; o__Flavobacteriales; f__Cryomorphaceae; g__Crocinitomix; Ambiguous_taxa
1002	3	16	31	0	0	0	50	35	28	0	0	0	k__Bacteria; p__Proteobacteria; c__Gammaproteobacteria; o__Oceanospirillales; f__Oceanospirillaceae; g__Reinekea
1495	0	0	0	0	0	0	14	0	0	0	0	0	k__Bacteria; p__Cyanobacteria; c__ML635J_21
1454	0	0	0	0	0	0	0	0	0	7	0	0	k__Bacteria; p__Firmicutes; c__Clostridia; o__Clostridiales; f__Ruminococcaceae
1413	0	0	0	0	0	0	0	0	0	52	0	0	k__Bacteria; p__Firmicutes; c__Clostridia; o__Clostridiales; f__Lachnospiraceae; g__Roseburia; Ambiguous_taxa
1085	0	0	57	0	0	0	0	0	0	14	10	0	k__Bacteria; p__Firmicutes; c__Clostridia; o__Clostridiales; f__Lachnospiraceae; g__Acetatifactor; s__uncultured_bacterium

1044	0	2	0	0	0	0	0	0	0	0	0	0	k__Bacteria; p__Proteobacteria; c__Deltaproteobacteria; o__Sh765B_TzT_29
1003	0	5	0	0	1	0	0	0	0	0	0	0	k__Bacteria; p__Bacteroidetes; c__Flavobacteriia; o__Flavobacteriales; f__Flavobacteriaceae
1496	0	0	0	0	0	0	10	12	10	0	0	0	k__Bacteria; p__Proteobacteria; c__Gammaproteobacteria; o__Oceanospirillales; f__Alcanivoracaceae; g__Kangiella; Ambiguous_taxa
1455	0	0	0	0	0	0	0	0	0	2	0	0	k__Bacteria; p__Firmicutes; c__Clostridia; o__Clostridiales; f__Peptostreptococcaceae; g__Tepidibacter; s__uncultured_bacterium
1414	0	0	0	0	0	0	0	0	0	40	0	0	k__Bacteria; p__Firmicutes; c__Clostridia; o__Clostridiales; f__Lachnospiraceae
1086	1	0	10	0	0	0	0	0	0	0	0	0	k__Bacteria; p__Acidobacteria; c__Holophagae; o__Subgroup_23
1045	0	17	20	0	0	0	0	0	0	0	0	0	k__Bacteria; p__Bacteroidetes; c__Flavobacteriia; o__Flavobacteriales; f__Flavobacteriaceae; g__Ornithobacterium; s__uncultured_bacterium
1004	0	7	13	0	0	0	0	0	0	0	0	0	k__Bacteria; p__Bacteroidetes; c__Flavobacteriia; o__Flavobacteriales; f__NS7_marine_group; g__uncultured_bacterium; s__uncultured_bacterium
1497	0	0	0	0	0	0	59	0	0	0	0	0	k__Bacteria; p__Firmicutes; c__Clostridia; o__Clostridiales; f__Clostridiales_vadinBB60_group; g__uncultured_bacterium; s__uncultured_bacterium
1456	0	0	0	0	0	0	0	0	0	5	0	0	k__Bacteria; p__Cyanobacteria; c__Cyanobacteria; o__SubsectionI; f__FamilyI

1415	0	0	0	0	0	0	0	0	0	20	0	0	k__Bacteria; p__Firmicutes; c__Clostridia; o__Clostridiales; f__Ruminococcaceae; g__Ruminococcaceae_UCG_005
1087	0	0	41	3	7	79	0	0	0	1	1	0	k__Bacteria; p__Firmicutes; c__Clostridia; o__Clostridiales; f__Ruminococcaceae
1046	0	12	12	0	0	0	0	0	0	0	0	0	k__Bacteria; p__Fibrobacteres; c__Fibrobacteria; o__Fibrobacterales; f__Fibrobacteraceae
1005	0	14	0	0	0	0	0	0	0	0	0	0	k__Bacteria; p__Bacteroidetes; c__Cytophagia; o__Cytophagales; f__Flammeovirgaceae; g__uncultured
1498	0	0	0	0	0	0	4	7	12	0	0	0	k__Bacteria; p__Proteobacteria; c__Gammaproteobacteria
1457	0	0	0	0	0	0	0	0	0	2	0	0	k__Bacteria; p__Bacteroidetes
1416	0	0	0	0	0	0	0	0	0	7	0	0	k__Bacteria; p__Firmicutes; c__Clostridia; o__Clostridiales; f__Lachnospiraceae; g__Howardella; s__uncultured_bacterium
1088	0	1	8	0	0	0	0	0	0	0	0	0	k__Bacteria; p__Proteobacteria; c__Gammaproteobacteria; o__Arenicellales; f__Arenicellaceae; g__Arenicella; s__uncultured_bacterium
1047	0	8	16	0	0	0	0	0	1	0	0	0	k__Bacteria; p__Proteobacteria; c__Alphaproteobacteria; o__DB1_14; f__uncultured_bacterium; g__uncultured_bacterium; s__uncultured_bacterium
1006	0	18	9	0	0	0	10	7	0	1	0	0	k__Bacteria; p__Spirochaetae; c__Spirochaetes; o__Spirochaetales; f__Spirochaetaceae; g__Spirochaeta_2
1499	0	0	0	0	0	0	12	0	0	0	0	0	k__Bacteria; p__Acidobacteria; c__Subgroup_22; o__uncultured_Acidobacteria_bacterium; f__uncultured_Acidobacteria_bacterium; g__uncultured_Acidobacteria_bacterium; s__uncultured_Acidobacteria_bacterium

1458	0	0	0	0	4	0	0	0	0	33	0	0	k__Bacteria; p__Firmicutes; c__Clostridia; o__Clostridiales; f__Clostridiaceae_1; g__Clostridium_sensu_stricto_1
1417	0	0	0	0	0	0	0	0	0	15	10	6	k__Bacteria; p__Firmicutes; c__Clostridia; o__Clostridiales; f__Ruminococcaceae; g__Anaerotruncus
1089	0	0	8	0	0	0	0	0	0	0	0	0	k__Bacteria; p__Proteobacteria; c__Alphaproteobacteria; o__Rhodospirillales; f__Rhodospirillaceae; g__Azospirillum; s__uncultured_bacterium
1048	0	3	7	0	0	0	0	0	0	0	0	0	k__Bacteria; p__Proteobacteria; c__Gammaproteobacteria; o__Oceanospirillales; f__Hahellaceae; g__Hahella; s__uncultured_gamma_proteobacterium
1007	3	7	7	0	0	0	5	1	4	0	0	0	k__Bacteria; p__Latescibacteria
1459	0	0	0	0	0	0	0	0	0	7	0	0	k__Bacteria; p__Proteobacteria; c__Deltaproteobacteria; o__Desulfovibrionales; f__Desulfovibrionaceae; g__Desulfovibrio
1418	0	0	0	0	0	0	0	0	1	33	5	6	k__Bacteria; p__Firmicutes; c__Clostridia; o__Clostridiales; f__Lachnospiraceae
1049	0	7	0	0	1	0	0	0	0	0	0	0	k__Bacteria; p__Bacteroidetes; c__Sphingobacteriia; o__Sphingobacteriales; f__Saprospiraceae; g__Portibacter; s__uncultured_Bacteroidetes_bacterium
1008	0	8	0	0	0	0	0	0	0	0	0	0	k__Bacteria; p__Proteobacteria; c__Deltaproteobacteria; o__Desulfovibrionales; f__Desulfovibrionaceae; g__Desulfovibrio
1419	0	0	0	0	0	0	0	0	0	9	0	6	k__Bacteria; p__Firmicutes; c__Clostridia; o__Clostridiales; f__Lachnospiraceae; g__Lachnoclostridium; s__human_gut_metagenome
1009	0	25	0	0	0	0	0	0	0	0	0	0	k__Bacteria; p__Bacteroidetes; c__Cytophagia;

													o__Cytophagales; f__Flammeovirgaceae
670	66	154	170	0	2	8	0	1	2	1	0	0	k__Bacteria; p__Bacteroidetes; c__Flavobacteriia; o__Flavobacteriales; f__Flavobacteriaceae; g__Tenacibaculum; s__uncultured_Flavobacteriales_bacterium
260	0	0	0	0	0	0	0	0	0	16	2	2	k__Bacteria; p__Firmicutes; c__Clostridia; o__Clostridiales; f__Lachnospiraceae; g__uncultured; s__uncultured_bacterium
671	5	8	32	0	0	0	15	42	14	0	0	0	k__Bacteria
630	0	0	41	0	0	0	1	0	2	31	9	0	k__Bacteria; p__Firmicutes; c__Clostridia; o__Clostridiales; f__Lachnospiraceae
261	0	0	6	9	4	1	761	651	830	148	32	13	k__Bacteria; p__Proteobacteria; c__Gammaproteobacteria; o__Alteromonadales; f__Alteromonadaceae; g__Neiella; Ambiguous_taxa
220	382	0	0	115	94	172	29	101	0	63	83	9	k__Bacteria; p__Firmicutes; c__Clostridia; o__Clostridiales; f__Ruminococcaceae; g__uncultured; s__uncultured_bacterium
672	0	0	2	1	2	4	8	14	5	2	0	1	k__Bacteria; p__Proteobacteria; c__Gammaproteobacteria; o__Pseudomonadales; f__Pseudomonadaceae; g__Pseudomonas; s__Pseudomonas_fluorescens
631	0	0	0	1	0	0	1	0	0	12	1	0	k__Bacteria; p__Firmicutes; c__Clostridia; o__Clostridiales; f__Lachnospiraceae; g__Lachnospira; s__uncultured_bacterium
262	0	0	19	15	23	9	22	15	15	5	0	0	k__Bacteria; p__Firmicutes; c__Bacilli; o__Bacillales; f__Bacillaceae; g__Bacillus; s__Bacillus_pumilus
673	8	6	15	2	0	2	18	9	23	0	0	0	k__Bacteria; p__Bacteroidetes; c__Cytophagia; o__Cytophagales; f__Flammeovirgaceae; g__Reichenbachiella
632	0	0	0	0	0	0	1	0	0	0	0	0	k__Bacteria; p__Bacteroidetes; c__Bacteroidia; o__Bacteroidales; f__Prevotellaceae
263	0	191	42	187	246	290	175	100	97	85	101	38	k__Bacteria; p__Firmicutes; c__Bacilli; o__Lactobacillales;

													f__Lactobacillaceae; g__Lactobacillus
222	1	24	30	18	6	4	17	3	8	218	50	39	k__Bacteria; p__Bacteroidetes; c__Bacteroidia; o__Bacteroidia_Incertae_Sedis; f__Prolixibacteraceae; g__Prolixibacter; Ambiguous_taxa
674	15	89	26	0	0	0	24	7	20	15	2	2	k__Bacteria; p__Bacteroidetes; c__Bacteroidia; o__Bacteroidales; f__Marinilabiaceae; g__Marinifilum; Ambiguous_taxa
633	184	125	122	102	124	128	131	162	94	108	321	424	k__Bacteria; p__Bacteroidetes; c__Bacteroidia; o__Bacteroidales; f__Rikenellaceae; g__Alistipes
264	0	0	0	0	0	0	1	0	0	72	7	4	k__Bacteria; p__Firmicutes; c__Clostridia; o__Clostridiales; f__Ruminococcaceae; g__Oscillospira
223	0	0	0	0	1	0	0	0	0	23	6	4	k__Bacteria; p__Firmicutes; c__Clostridia; o__Clostridiales; f__Lachnospiraceae
675	4	1	31	0	0	0	0	0	0	1	0	0	k__Bacteria; p__Firmicutes; c__Clostridia; o__Clostridiales; f__Defluviitaleaceae; g__Defluviitaleaceae_UCG_011; Ambiguous_taxa
634	0	9	0	0	36	0	0	0	0	8	16	9	k__Bacteria; p__Firmicutes; c__Clostridia; o__Clostridiales; f__Ruminococcaceae; g__Ruminococcaceae_UCG_004; s__uncultured_bacterium
265	21	59	99	39	27	17	0	0	0	0	0	1	k__Bacteria; p__Bacteroidetes; c__Bacteroidia; o__Bacteroidia_Incertae_Sedis; f__Draconibacteriaceae; g__Draconibacterium; s__uncultured_bacterium
224	0	0	29	41	1	118	75	0	0	33	7	1	k__Bacteria; p__Bacteroidetes; c__Bacteroidia; o__Bacteroidales; f__Prevotellaceae
676	0	14	12	0	2	0	0	0	0	2	0	0	k__Bacteria; p__Acidobacteria; c__Holophagae; o__Subgroup_10; f__CA002

635	0	0	3	0	0	41	0	14	2	7	47	15	k__Bacteria; p__Firmicutes; c__Clostridia; o__Clostridiales; f__Lachnospiraceae; g__Lachnospiraceae_NK4A136_group; s__uncultured_bacterium
266	0	0	0	35	0	0	0	0	0	28	3	0	k__Bacteria; p__Firmicutes; c__Clostridia; o__Clostridiales; f__Ruminococcaceae; g__Anaerotruncus
225	0	1	0	5	1	3	36	17	20	3	1	1	k__Bacteria; p__Proteobacteria; c__Gammaproteobacteria; o__Oceanospirillales; f__Oceanospirillaceae; g__Marinobacterium
677	0	0	0	0	0	0	0	0	0	13	2	0	k__Bacteria; p__Firmicutes; c__Clostridia; o__Clostridiales; f__Ruminococcaceae; g__uncultured; Ambiguous_taxa
636	0	0	0	0	0	0	0	22	0	11	3	0	k__Bacteria; p__Firmicutes; c__Clostridia; o__Clostridiales; f__Lachnospiraceae; g__Roseburia; s__Eubacterium_sp._14_2
267	116	711	710	2	2	0	0	14	10	29	15	8	k__Bacteria; p__Proteobacteria; c__Epsilonproteobacteria; o__Campylobacterales; f__Campylobacteraceae; g__Arcobacter
226	0	0	0	0	0	0	0	0	0	19	0	0	k__Bacteria; p__Firmicutes; c__Clostridia; o__Clostridiales; f__Lachnospiraceae
678	10	67	70	1	1	3	15	9	11	0	0	0	k__Bacteria; p__Proteobacteria; c__Gammaproteobacteria; o__Thiotrichales; f__Thiotrichaceae; g__uncultured
637	0	0	1	0	0	0	0	0	0	0	21	67	k__Bacteria; p__Firmicutes; c__Clostridia; o__Clostridiales; f__Lachnospiraceae
268	0	0	0	78	48	56	0	0	1	24	13	3	k__Bacteria; p__Firmicutes; c__Clostridia; o__Clostridiales; f__Lachnospiraceae; g__Coprococcus_1
227	0	0	0	1	0	0	1	0	1	37	2	0	k__Bacteria; p__Firmicutes; c__Clostridia; o__Clostridiales; f__Lachnospiraceae; g__Lachnospiraceae_UCG_004; Ambiguous_taxa

638	0	0	0	0	0	0	77	0	0	18	11	3	k__Bacteria; p__Firmicutes; c__Clostridia; o__Clostridiales; f__Lachnospiraceae; g__Roseburia; s__uncultured_bacterium
269	125	0	56	0	0	73	0	0	0	1	24	2	k__Bacteria; p__Firmicutes; c__Clostridia; o__Clostridiales; f__Lachnospiraceae; g__Lachnospiraceae_NK4A136_group
228	0	0	1	15	0	1	0	0	0	0	0	0	k__Bacteria; p__Proteobacteria; c__Betaproteobacteria; o__Burkholderiales; f__Oxalobacteraceae; g__Janthinobacterium; s__uncultured_bacterium
639	0	8	0	0	0	0	43	0	0	10	40	15	k__Bacteria; p__Bacteroidetes; c__Bacteroidia; o__Bacteroidales; f__Prevotellaceae
229	0	59	0	31	6	64	51	0	12	2	18	7	k__Bacteria; p__Proteobacteria; c__Epsilonproteobacteria; o__Campylobacterales; f__Campylobacteraceae; g__Campylobacter
1790	0	0	0	0	0	0	0	3	7	0	0	0	k__Bacteria; p__Bacteroidetes; c__Sphingobacteriia; o__Sphingobacteriales; f__Chitinophagaceae; g__uncultured; s__uncultured_Bacteroidetes/Chlorobi_group_bacterium
1791	0	0	0	0	0	0	0	0	9	0	0	0	k__Bacteria; p__Candidate_division_SR1
1750	0	0	0	0	0	0	1	7	0	0	0	0	k__Bacteria; p__Proteobacteria; c__Gammaproteobacteria; o__Oceanospirillales; f__Oceanospirillales_Incertae_Sedis; g__Gynuella; s__uncultured_bacterium
1381	0	0	0	0	0	0	0	56	0	0	3	4	k__Bacteria; p__Bacteroidetes; c__Bacteroidia; o__Bacteroidales; f__Rikenellaceae; g__Alistipes; s__uncultured_bacterium
1340	0	0	0	0	0	0	0	0	35	2	0	0	k__Bacteria; p__Firmicutes; c__Clostridia; o__Clostridiales; f__Lachnospiraceae; g__Lachnospiraceae_UCG_010; s__uncultured_bacterium

1792	0	0	0	0	0	1	0	0	39	0	6	0	k__Bacteria; p__Firmicutes; c__Erysipelotrichia; o__Erysipelotrichales; f__Erysipelotrichaceae; g__Candidatus_Stoquefichus; s__uncultured_bacterium
1751	0	0	0	0	0	0	9	11	5	0	0	0	k__Bacteria; p__Bacteroidetes; c__Bacteroidetes_BD2_2; Ambiguous_taxa; Ambiguous_taxa; Ambiguous_taxa; Ambiguous_taxa
1710	0	0	0	0	0	0	1	2	3	0	0	0	k__Bacteria; p__Proteobacteria; c__Alphaproteobacteria; o__Rhodospirillales; f__Rhodospirillaceae; g__Defluviicoccus
1341	0	0	0	0	0	0	0	0	0	10	1	0	k__Bacteria; p__Proteobacteria; c__Gammaproteobacteria; o__Pasteurellales; f__Pasteurellaceae; g__Actinobacillus
1300	0	4	0	0	0	0	0	0	0	0	0	0	k__Bacteria; p__Firmicutes; c__Clostridia; o__Clostridiales; f__Family_XIII
1793	0	0	0	0	0	0	0	1	10	0	0	0	k__Bacteria; p__Actinobacteria; c__Actinobacteria; o__Corynebacteriales; f__Mycobacteriaceae; g__Mycobacterium; Ambiguous_taxa
1752	0	0	0	0	0	0	1	10	1	0	0	0	k__Bacteria; p__Proteobacteria; c__Gammaproteobacteria
1711	0	0	1	0	0	1	2	0	0	1	0	0	k__Bacteria; p__Proteobacteria; c__Deltaproteobacteria; o__Desulfobacterales; f__Desulfobulbaceae; g__Desulfobulbus
1383	0	0	0	0	0	0	0	0	0	1	0	0	k__Bacteria; p__Bacteroidetes
1794	0	0	0	0	0	0	0	0	7	0	0	0	k__Bacteria; p__Proteobacteria; c__TA18
1753	0	0	0	0	0	0	0	6	0	0	0	0	k__Bacteria; p__Proteobacteria; c__Gammaproteobacteria; o__Order_Incertae_Sedis; f__Family_Incertae_Sedis; g__Marinicella
1384	0	6	0	0	0	0	0	0	0	0	0	0	k__Bacteria; p__Tenericutes; c__Mollicutes; o__NB1_n
1343	0	0	0	0	0	0	0	0	0	1	0	0	k__Bacteria; p__Proteobacteria; c__Deltaproteobacteria; o__Desulfobacterales; f__Desulfobulbaceae

1302	0	0	0	1	0	0	1	0	0	0	0	0	k__Bacteria; p__Proteobacteria; c__Betaproteobacteria; o__Burkholderiales; f__Alcaligenaceae; g__MWH_UniP1_aquatic_group; s__uncultured_bacterium
1795	0	0	0	0	0	0	0	0	15	0	0	0	k__Bacteria; p__Bacteroidetes; c__Sphingobacteriia; o__Sphingobacteriales; f__WCHB1_69; g__uncultured_prokaryote; s__uncultured_prokaryote
1754	0	0	0	0	0	0	0	7	0	0	0	0	k__Bacteria; p__Proteobacteria; c__Gammaproteobacteria; o__Alteromonadales; f__Alteromonadaceae
1344	0	0	0	0	0	0	0	0	1	20	2	0	k__Bacteria; p__Firmicutes; c__Negativicutes; o__Selenomonadales; f__Veillonellaceae; g__Allisonella; s__uncultured_bacterium
1303	0	1	8	0	0	0	13	13	22	4	1	0	k__Bacteria; p__Bacteroidetes; c__Bacteroidia; o__Bacteroidia_Incertae_Sedis; f__Draconibacteriaceae; g__Draconibacterium; s__uncultured_bacterium
1796	0	0	0	0	0	0	0	0	4	0	0	0	k__Bacteria
1755	0	0	0	0	0	0	0	5	0	0	0	0	k__Bacteria; p__Proteobacteria; c__Gammaproteobacteria; o__Alteromonadales; f__Alteromonadaceae; g__Bowmanella; Ambiguous_taxa
1714	0	0	0	0	0	0	6	1	2	0	0	0	k__Bacteria; p__Actinobacteria; c__Actinobacteria; o__PeM15
1386	0	0	0	0	0	0	0	0	0	12	4	0	k__Bacteria; p__Firmicutes; c__Clostridia; o__Clostridiales; f__Ruminococcaceae; g__Ruminococcaceae_UCG_010
1345	0	1	0	0	0	0	18	0	4	0	0	0	k__Bacteria; p__Bacteroidetes; c__Bacteroidia; o__Bacteroidia_Incertae_Sedis; f__Draconibacteriaceae; g__Draconibacterium; s__uncultured_bacterium
1304	0	0	0	0	0	0	0	0	0	6	7	1	k__Bacteria; p__Proteobacteria; c__Deltaproteobacteria; o__Bdellovibrionales; f__Bacteriovoracaceae

1797	0	0	0	0	0	0	0	0	2	0	0	0	k__Bacteria; p__Proteobacteria; c__Gammaproteobacteria
1756	0	0	2	0	0	0	2	10	13	0	0	0	k__Bacteria; p__Tenericutes; c__Mollicutes; o__Mycoplasmatales; f__Mycoplasmataceae; g__uncultured; s__uncultured_bacterium
1715	0	0	2	1	0	0	0	0	0	0	0	0	k__Bacteria; p__Proteobacteria; c__Deltaproteobacteria; o__Bdellovibrionales; f__Bacteriovoracaceae
1387	0	0	0	0	0	0	6	0	0	1	1	0	k__Bacteria; p__Firmicutes; c__Clostridia; o__Clostridiales; f__Family_XII; g__Fusibacter; s__uncultured_bacterium
1305	0	0	0	0	0	0	0	0	0	11	3	0	k__Bacteria
1798	0	0	0	0	0	0	0	0	7	0	0	0	k__Bacteria; p__Cloacimonetes; c__MSBL8
1757	2	0	0	4	3	0	2	13	3	0	0	0	k__Bacteria; p__Proteobacteria; c__Deltaproteobacteria; o__Sva0485
1716	0	15	2	0	0	0	0	0	11	0	0	0	k__Bacteria; p__Proteobacteria; c__Gammaproteobacteria; o__Oceanospirillales; f__OM182_clade; g__uncultured_organism; s__uncultured_organism
1388	0	0	5	0	0	0	0	0	0	1	1	0	k__Bacteria; p__Proteobacteria; c__Deltaproteobacteria; o__Desulfovibrionales; f__Desulfovibrionaceae; g__Desulfovibrio
1306	0	0	0	0	0	0	1	0	0	8	0	0	k__Bacteria; p__Bacteroidetes
1799	0	0	0	0	0	0	0	0	7	0	0	0	k__Bacteria; p__Spirochaetae; c__Spirochaetes; o__Spirochaetales; f__Spirochaetaceae; g__Spirochaeta_2
1758	0	0	0	0	0	0	0	17	0	0	0	0	k__Bacteria; p__Proteobacteria; c__Gammaproteobacteria; o__Thiotrichales; f__Thiotrichaceae; g__uncultured
1717	0	0	4	0	0	0	0	0	0	0	0	0	k__Bacteria; p__Gemmatimonadetes; c__Gemmatimonadetes; o__BD2_11_terrestrial_group

1389	0	0	0	0	0	0	0	0	0	0	2	0	k__Bacteria; p__Proteobacteria; c__Deltaproteobacteria; o__Desulfuromonadales; f__Geobacteraceae; g__Geothermobacter; s__uncultured_bacterium
1348	7	31	93	0	0	0	3	6	14	16	2	1	k__Bacteria; p__Firmicutes; c__Clostridia; o__Clostridiales; f__Family_XII; g__Fusibacter; Ambiguous_taxa
1307	0	0	0	0	0	0	0	0	0	1	2	1	k__Bacteria; p__Proteobacteria; c__Gammaproteobacteria; o__Oceanospirillales; f__Hahellaceae; g__Endozoicomonas
1759	0	0	0	0	0	0	1	10	0	0	0	0	k__Bacteria; p__Chlorobi; c__Chlorobia; o__Chlorobiales; f__OPB56; g__uncultured_bacterium; s__uncultured_bacterium
1718	0	0	0	0	0	0	6	11	17	0	0	0	k__Bacteria; p__Proteobacteria; c__Deltaproteobacteria; o__Desulfobacterales; f__Desulfobacteraceae
1349	0	2	15	1	0	3	11	14	1	1	0	1	k__Bacteria; p__Proteobacteria; c__Deltaproteobacteria; o__Desulfobacterales; f__Desulfobacteraceae; g__Sva0081_sediment_group; Ambiguous_taxa
1308	0	0	0	0	0	0	0	1	0	0	0	0	k__Bacteria; p__Proteobacteria
1719	0	0	3	0	0	0	0	0	1	0	0	0	k__Bacteria; p__Tenericutes; c__Mollicutes; o__NB1_n; f__bacterium_enrichment_culture_clone_R4_81B; g__bacterium_enrichment_culture_clone_R4_81B; s__bacterium_enrichment_culture_clone_R4_81B
1309	0	0	0	0	3	1	0	0	0	0	0	0	k__Bacteria; p__Proteobacteria; c__Gammaproteobacteria
80	0	0	0	0	17	0	0	0	0	0	9	0	k__Bacteria; p__Firmicutes; c__Bacilli; o__Lactobacillales; f__Lactobacillaceae; g__Lactobacillus; s__Lactobacillus_mucosae
81	1	79	0	0	0	0	0	1	1	42	3	1	k__Bacteria; p__Firmicutes; c__Clostridia; o__Clostridiales; f__Lachnospiraceae; g__Lachnospira; s__uncultured_bacterium

40	593	660	176	727	701	767	294	651	414	401	656	192	k__Bacteria; p__Firmicutes; c__Bacilli; o__Lactobacillales; f__Lactobacillaceae; g__Lactobacillus; s__Lactobacillus_johnsonii
190	279	136	1	110	355	99	66	254	69	36	116	25	k__Bacteria; p__Bacteroidetes; c__Bacteroidia; o__Bacteroidales; f__Bacteroidales_S24_7_group; g__uncultured_bacterium; s__uncultured_bacterium
970	0	9	0	0	0	0	0	0	0	0	0	0	k__Bacteria; p__Proteobacteria; c__Gammaproteobacteria; o__NKB5
82	1	296	164	127	51	50	121	105	122	44	66	1	k__Bacteria; p__Bacteroidetes; c__Bacteroidia; o__Bacteroidales; f__Prevotellaceae; g__Prevotellaceae_NK3B31_group; s__uncultured_bacterium
560	0	0	0	0	0	27	0	0	0	0	0	0	k__Bacteria; p__Firmicutes; c__Bacilli; o__Bacillales; f__Bacillaceae; g__Oceanobacillus; s__uncultured_bacterium
41	0	117	3	164	223	43	52	136	31	24	48	10	k__Bacteria; p__Bacteroidetes; c__Bacteroidia; o__Bacteroidales; f__Bacteroidaceae; g__Bacteroides
191	0	0	1	146	145	176	0	0	0	0	0	0	k__Bacteria; p__Proteobacteria; c__Gammaproteobacteria; o__Vibrionales; f__Vibrionaceae; g__Vibrio
150	0	0	22	0	33	0	0	0	0	4	1	0	k__Bacteria; p__Firmicutes; c__Clostridia; o__Clostridiales; f__Clostridiales_vadinBB60_group; g__uncultured_bacterium; s__uncultured_bacterium
971	0	5	19	0	1	0	0	0	0	0	0	0	k__Bacteria; p__Bacteroidetes; c__Bacteroidetes_VC2.1_Bac22; o__uncultured_Bacteroidetes_bacterium; f__uncultured_Bacteroidetes_bacterium; g__uncultured_Bacteroidetes_bacterium; s__uncultured_Bacteroidetes_bacterium
930	0	8	5	0	0	0	0	0	0	0	0	0	k__Bacteria; p__TA06

83	909	480	427	1020	1110	852	496	1126	675	936	1012	287	k__Bacteria; p__Firmicutes; c__Bacilli; o__Bacillales; f__Staphylococcaceae; g__Staphylococcus; s__Staphylococcus_sp._SV3
561	0	0	0	0	0	43	0	0	0	0	4	1	k__Bacteria; p__Firmicutes; c__Clostridia; o__Clostridiales; f__Lachnospiraceae; g__Roseburia; s__uncultured_bacterium
520	0	5	18	3	8	13	0	0	0	0	0	0	k__Bacteria; p__Bacteroidetes; c__Flavobacteriia; o__Flavobacteriales; f__Flavobacteriaceae; g__Lutimonas; s__uncultured_Bacteroidetes_bacterium
42	80	150	1	0	0	97	1	55	77	372	28	6	k__Bacteria; p__Firmicutes; c__Clostridia; o__Clostridiales; f__Ruminococcaceae; g__Ruminococcaceae_UCG_014
192	0	0	1	0	0	7	0	0	0	0	0	0	k__Bacteria; p__Bacteroidetes; c__Bacteroidetes_VC2.1_Bac22
151	0	0	0	42	36	0	3	71	113	26	13	2	k__Bacteria; p__Firmicutes; c__Clostridia; o__Clostridiales; f__Ruminococcaceae; g__Subdoligranulum; s__uncultured_bacterium
110	0	0	0	0	0	0	0	0	0	0	10	1	k__Bacteria; p__Firmicutes; c__Clostridia; o__Clostridiales; f__Lachnospiraceae; g__Butyrivibrio; s__uncultured_bacterium
972	6	46	28	0	0	0	0	0	0	0	0	0	k__Bacteria; p__Proteobacteria; c__Deltaproteobacteria; o__Desulfobacterales; f__Desulfobulbaceae
931	0	8	0	0	0	0	0	0	0	0	0	0	k__Bacteria; p__Gracilibacteria
84	0	0	0	0	0	0	0	0	0	0	6	11	k__Bacteria; p__Firmicutes; c__Clostridia; o__Clostridiales; f__Lachnospiraceae
562	0	0	0	0	0	47	2	0	2	7	3	0	k__Bacteria; p__Bacteroidetes; c__Bacteroidia; o__Bacteroidales; f__Rikenellaceae; g__Rikenellaceae_RC9_gut_group; s__uncultured_bacterium
521	0	0	36	0	9	0	0	0	0	11	17	22	k__Bacteria; p__Firmicutes; c__Clostridia; o__Clostridiales; f__Ruminococcaceae

43	0	0	0	0	0	0	1	1	4	40	0	0	k__Bacteria; p__Bacteroidetes; c__Bacteroidia; o__Bacteroidales; f__Prevotellaceae; g__Prevotellaceae_NK3B31_group; s__uncultured_bacterium
193	0	0	0	109	0	1	28	108	148	230	28	4	k__Bacteria; p__Bacteroidetes; c__Bacteroidia; o__Bacteroidales; f__Prevotellaceae; g__Alloprevotella; s__uncultured_bacterium
152	0	0	1	0	0	0	0	0	0	31	0	0	k__Bacteria; p__Firmicutes; c__Clostridia; o__Clostridiales; f__Ruminococcaceae; g__Ruminococcaceae_UCG_005
111	1	46	49	50	8	21	28	68	84	14	1	3	k__Bacteria; p__Firmicutes; c__Bacilli; o__Lactobacillales; f__Streptococcaceae; g__Streptococcus; s__Streptococcus_salivarius_subsp._thermophilus
973	0	3	3	0	0	0	1	0	0	0	0	0	k__Bacteria; p__Spirochaetae; c__Spirochaetes; o__Spirochaetales; f__Spirochaetaceae; g__Spirochaeta_2
932	0	7	0	0	0	0	0	0	0	0	0	0	k__Bacteria; p__Aminicenantes; c__uncultured_delta_proteobacterium; o__uncultured_delta_proteobacterium; f__uncultured_delta_proteobacterium; g__uncultured_delta_proteobacterium; s__uncultured_delta_proteobacterium
85	0	0	0	0	114	0	2	22	1	5	11	0	k__Bacteria; p__Firmicutes; c__Clostridia; o__Clostridiales; f__Ruminococcaceae; g__Ruminococcus_1
563	0	0	0	0	0	66	0	0	0	61	0	0	k__Bacteria; p__Firmicutes; c__Clostridia; o__Clostridiales; f__Ruminococcaceae; g__Ruminococcaceae_UCG_014; Ambiguous_taxa
522	0	0	0	0	33	0	0	0	0	1	14	4	k__Bacteria; p__Proteobacteria; c__Deltaproteobacteria; o__Desulfovibrionales; f__Desulfovibrionaceae

44	0	0	0	0	0	122	0	0	0	0	12	11	k__Bacteria; p__Firmicutes; c__Clostridia; o__Clostridiales; f__Ruminococcaceae; g__Anaerotruncus; s__uncultured_bacterium
194	7	0	0	0	0	20	0	1	0	17	0	0	k__Bacteria; p__Firmicutes; c__Clostridia; o__Clostridiales; f__Ruminococcaceae; g__Oscillibacter
153	0	0	0	0	0	0	1	0	1	72	1	0	k__Bacteria; p__Firmicutes; c__Erysipelotrichia; o__Erysipelotrichales; f__Erysipelotrichaceae; g__uncultured; s__uncultured_bacterium
112	0	0	0	0	0	0	0	1	17	0	3	0	k__Bacteria; p__Firmicutes; c__Clostridia; o__Clostridiales; f__Lachnospiraceae
974	0	4	2	0	0	0	0	0	2	0	0	0	k__Bacteria; p__Bacteroidetes; c__Bacteroidia; o__Bacteroidales; f__Marinilabiaceae; g__uncultured
933	1	3	20	0	0	0	0	1	3	0	1	0	k__Bacteria; p__Firmicutes; c__Clostridia; o__Clostridiales; f__Defluviitaleaceae; g__Defluviitaleaceae_UCG_011; s__Clostridia_bacterium_S710(2)_1
86	328	554	194	52	107	60	64	56	102	237	7	5	k__Bacteria; p__Bacteroidetes; c__Bacteroidia; o__Bacteroidales; f__Prevotellaceae; g__Prevotella_1; s__uncultured_bacterium
564	0	0	0	0	1	54	0	0	0	6	14	20	k__Bacteria; p__Firmicutes; c__Clostridia; o__Clostridiales; f__Lachnospiraceae
523	2	102	30	0	54	193	28	0	113	57	57	35	k__Bacteria; p__Firmicutes; c__Clostridia; o__Clostridiales; f__Ruminococcaceae; g__Oscillibacter; s__uncultured_bacterium
45	0	0	0	19	87	0	0	0	60	13	36	12	k__Bacteria; p__Bacteroidetes; c__Bacteroidia; o__Bacteroidales; f__Bacteroidales_S24_7_group; g__uncultured_bacterium; s__uncultured_bacterium

195	0	0	0	0	0	0	0	0	0	0	5	0	k__Bacteria; p__Firmicutes; c__Clostridia; o__Clostridiales
154	0	0	0	0	16	0	0	37	0	2	2	0	k__Bacteria; p__Firmicutes; c__Clostridia; o__Clostridiales; f__Ruminococcaceae; g__Oscillospira
113	0	0	0	120	142	0	0	1	0	94	37	3	k__Bacteria; p__Firmicutes; c__Clostridia; o__Clostridiales; f__Lachnospiraceae; g__uncultured; s__uncultured_bacterium
975	0	11	7	1	0	0	0	0	0	0	0	0	k__Bacteria; p__Proteobacteria; c__Gammaproteobacteria; o__Xanthomonadales; f__uncultured; g__uncultured_gamma_proteobacterium; s__uncultured_gamma_proteobacterium
934	0	14	0	0	0	0	0	0	0	0	0	0	k__Bacteria; p__Gracilibacteria
87	0	1	43	37	104	0	69	0	0	22	29	26	k__Bacteria; p__Firmicutes; c__Clostridia; o__Clostridiales; f__Ruminococcaceae; g__Anaerotruncus; s__Anaerotruncus_sp._G3(2012)
565	0	2	0	0	0	44	0	0	0	2	0	0	k__Bacteria; p__Firmicutes; c__Clostridia; o__Clostridiales; f__Ruminococcaceae; g__Ruminococcaceae_UCG_005; Ambiguous_taxa
524	0	0	0	0	37	0	0	0	0	0	2	0	k__Bacteria; p__Firmicutes; c__Clostridia; o__Clostridiales; f__Christensenellaceae; g__Christensenellaceae_R_7_group; Ambiguous_taxa
46	1167	867	224	972	895	1268	720	729	637	367	1574	611	k__Bacteria; p__Bacteroidetes; c__Bacteroidia; o__Bacteroidales; f__Prevotellaceae; g__Alloprevotella; s__uncultured_bacterium
196	0	0	1	0	0	61	1	0	0	270	0	0	k__Bacteria; p__Bacteroidetes; c__Bacteroidia; o__Bacteroidales; f__Bacteroidales_S24_7_group; g__uncultured_bacterium; s__uncultured_bacterium

155	1	198	34	178	164	83	230	43	102	96	539	170	k__Bacteria; p__Bacteroidetes; c__Bacteroidia; o__Bacteroidales; f__Bacteroidales_S24_7_group; Ambiguous_taxa; Ambiguous_taxa
114	433	161	0	7	151	190	1	29	74	38	56	4	k__Bacteria; p__Bacteroidetes; c__Bacteroidia; o__Bacteroidales; f__Bacteroidales_S24_7_group; g__uncultured_bacterium; s__uncultured_bacterium
976	0	18	9	0	0	0	0	0	0	0	0	0	k__Bacteria; p__Bacteroidetes; c__Bacteroidetes_BD2_2
935	2	24	9	3	2	1	0	1	0	0	0	0	k__Bacteria; p__Bacteroidetes; c__Bacteroidia; o__Bacteroidales; f__Marinilabiaceae; g__uncultured
88	230	662	66	341	285	220	192	257	111	320	982	1979	k__Bacteria; p__Firmicutes; c__Clostridia; o__Clostridiales; f__Lachnospiraceae; g__Lachnospiraceae_NK4A136_group; s__uncultured_bacterium
566	0	8	2	0	0	47	0	0	1	0	0	0	k__Bacteria; p__Proteobacteria; c__Betaproteobacteria; o__Burkholderiales; f__Comamonadaceae; g__Hydrogenophaga
525	0	0	28	0	21	66	0	1	0	8	4	3	k__Bacteria; p__Firmicutes; c__Clostridia; o__Clostridiales; f__Lachnospiraceae
47	0	0	0	36	0	125	65	129	0	43	50	52	k__Bacteria; p__Firmicutes; c__Clostridia; o__Clostridiales; f__Lachnospiraceae; g__Tyzzerella; s__uncultured_bacterium
197	0	0	46	99	69	2	0	1	0	34	18	0	k__Bacteria; p__Firmicutes; c__Clostridia; o__Clostridiales; f__Lachnospiraceae
156	0	0	0	48	1	130	0	0	46	13	54	62	k__Bacteria; p__Firmicutes; c__Clostridia; o__Clostridiales; f__Clostridiales_vadinBB60_group; Ambiguous_taxa; Ambiguous_taxa
115	0	0	0	0	33	141	3	2	1	17	11	0	k__Bacteria; p__Bacteroidetes; c__Bacteroidia; o__Bacteroidales; f__Prevotellaceae; g__Prevotellaceae_NK3B31_group

977	0	6	0	0	0	0	0	0	0	0	0	0	k__Bacteria; p__Actinobacteria; c__Actinobacteria; o__PeM15; f__uncultured_bacterium; g__uncultured_bacterium; s__uncultured_bacterium
936	0	5	0	0	0	0	0	0	0	0	0	0	k__Bacteria; p__Bacteroidetes; c__Bacteroidia; o__Bacteroidales; f__Marinilabiaceae; g__Saccharicrinis
89	0	8	72	120	126	138	3	102	53	16	56	62	k__Bacteria; p__Firmicutes; c__Clostridia; o__Clostridiales; f__Ruminococcaceae
567	0	0	1	6	0	13	0	0	0	0	0	0	k__Bacteria; p__Bacteroidetes
526	159	0	2	2	45	1	0	0	0	11	21	20	k__Bacteria; p__Firmicutes; c__Clostridia; o__Clostridiales; f__Lachnospiraceae; g__Lachnospiraceae_NK4A136_group
48	394	0	0	35	127	122	0	80	3	160	27	7	k__Bacteria; p__Firmicutes; c__Negativicutes; o__Selenomonadales; f__Veillonellaceae; g__Dialister; s__uncultured_bacterium
198	0	0	0	0	0	1	0	0	0	0	0	0	k__Bacteria; p__Firmicutes; c__Clostridia; o__Clostridiales; f__Lachnospiraceae
157	0	0	0	0	0	0	0	5	0	56	0	0	k__Bacteria; p__Firmicutes; c__Clostridia; o__Clostridiales; f__Ruminococcaceae; g__Ruminiclostridium_9
116	945	344	167	0	82	0	241	202	204	228	161	30	k__Bacteria; p__Firmicutes; c__Clostridia; o__Clostridiales; f__Lachnospiraceae
978	0	6	5	0	0	0	0	0	0	0	0	0	k__Bacteria; p__Bacteroidetes; c__Sphingobacteriia; o__Sphingobacteriales; f__Saprospiraceae; g__uncultured
937	0	1	0	0	0	0	61	97	57	9	2	0	k__Bacteria; p__Proteobacteria; c__Gammaproteobacteria; o__Oceanospirillales; f__Oceanospirillaceae; g__Neptuniibacter; Ambiguous_taxa
568	16	20	48	4	2	5	0	0	0	0	0	0	k__Bacteria; p__Bacteroidetes; c__Sphingobacteriia; o__Sphingobacteriales; f__Saprospiraceae; g__uncultured

527	0	0	0	0	6	0	0	0	0	0	0	0	k__Bacteria; p__Firmicutes; c__Clostridia; o__Clostridiales; f__Christensenellaceae; g__uncultured; s__bacterium_enrichment_culture_clone_51
49	756	857	180	562	771	659	353	396	448	457	396	60	k__Bacteria; p__Bacteroidetes; c__Bacteroidia; o__Bacteroidales; f__Bacteroidales_S24_7_group; Ambiguous_taxa; Ambiguous_taxa
158	1	113	0	0	0	0	0	0	0	4	0	0	k__Bacteria; p__Proteobacteria; c__Betaproteobacteria; o__Burkholderiales; f__Alcaligenaceae; g__Sutterella
117	0	0	0	0	0	0	0	0	0	15	0	0	k__Bacteria; p__Actinobacteria; c__Coriobacteriia; o__Coriobacteriales; f__Coriobacteriaceae; g__Collinsella; s__uncultured_bacterium
979	2	8	11	0	0	0	0	0	0	0	0	0	k__Bacteria; p__Proteobacteria; c__Gammaproteobacteria; o__Oceanospirillales; f__Oceanospirillaceae
938	0	9	7	0	0	0	0	0	0	0	0	0	k__Bacteria; p__Bacteroidetes; c__Bacteroidetes_VC2.1_Bac22
569	0	0	0	0	0	80	1	94	87	24	3	0	k__Bacteria; p__Firmicutes; c__Clostridia; o__Clostridiales; f__Ruminococcaceae; g__Ruminococcus_1
528	0	0	0	0	32	0	0	0	0	0	0	0	k__Bacteria; p__Firmicutes; c__Clostridia; o__Clostridiales; f__Ruminococcaceae; g__Ruminococcaceae_UCG_014
159	125	0	24	68	14	37	32	0	0	16	67	9	k__Bacteria; p__Bacteroidetes; c__Bacteroidia; o__Bacteroidales; f__Bacteroidales_S24_7_group; g__uncultured_bacterium; s__uncultured_bacterium
118	1	150	1	66	62	0	17	40	131	79	47	4	k__Bacteria; p__Firmicutes; c__Clostridia; o__Clostridiales; f__Lachnospiraceae; g__Blautia; s__Lachnospiraceae_bacterium_615
939	2	28	15	0	0	0	44	28	46	0	0	0	k__Bacteria; p__Fibrobacteres; c__Fibrobacteria; o__Fibrobacteria_Incertae_Sedis; f__Unknown_Family;

													g__possible_genus_03; s__uncultured_bacterium
529	0	0	0	0	16	0	0	0	1	66	2	0	k__Bacteria; p__Firmicutes; c__Clostridia; o__Clostridiales; f__Clostridiaceae_1; g__Clostridium_sensu_stricto_1
119	445	597	195	118	296	349	170	122	154	126	679	332	k__Bacteria; p__Bacteroidetes; c__Bacteroidia; o__Bacteroidales; f__Prevotellaceae; g__Prevotellaceae_UCG_001
1680	0	0	0	0	29	0	0	0	0	0	1	0	k__Bacteria; p__Firmicutes; c__Clostridia; o__Clostridiales; f__Lachnospiraceae
1270	0	0	0	0	0	0	0	0	0	6	7	0	k__Bacteria; p__Proteobacteria; c__Gammaproteobacteria; o__Oceanospirillales; f__Oceanospirillaceae; g__Marinomonas
1271	0	0	1	0	0	0	0	0	0	1	0	0	k__Bacteria; p__Bacteroidetes; c__Bacteroidia; o__Bacteroidales; f__Marinilabiaceae; g__Marinifilum
1230	0	0	0	0	0	0	42	1	0	6	6	4	k__Bacteria; p__Firmicutes; c__Clostridia; o__Clostridiales; f__Ruminococcaceae; g__uncultured; s__unidentified
1641	0	0	0	0	0	0	2	1	9	0	0	0	k__Bacteria; p__Proteobacteria; c__Deltaproteobacteria; o__Desulfobacterales; f__Desulfobulbaceae; g__Desulfobulbus
1600	0	0	0	0	0	0	1	0	4	0	0	0	k__Bacteria; p__Proteobacteria; c__Gammaproteobacteria; o__Cellvibrionales; f__Spongiibacteraceae; g__BD1_7_clade; s__uncultured_gamma_proteobacterium
1272	0	0	0	0	0	0	0	1	0	0	0	0	k__Bacteria; p__Proteobacteria; c__Gammaproteobacteria
1231	0	0	1	0	0	0	0	0	0	0	0	0	k__Bacteria; p__Actinobacteria; c__Acidimicrobiia; o__Acidimicrobiales; f__Sva0996_marine_group; g__uncultured_bacterium; s__uncultured_bacterium
1642	0	0	0	0	0	1	0	0	0	0	0	0	k__Bacteria; p__Bacteroidetes; c__Bacteroidia; o__Bacteroidales; f__Prevotellaceae;

													g__Prevotellaceae_NK3B31_group; s__uncultured_bacterium
1601	0	0	0	0	0	0	3	20	6	0	0	0	k__Bacteria; p__Proteobacteria; c__Gammaproteobacteria; o__Cellvibrionales; f__Halieaceae; g__Haliea; s__uncultured_gamma_proteobacterium
1232	0	0	0	0	0	0	0	0	0	0	5	4	k__Bacteria; p__Firmicutes; c__Clostridia; o__Clostridiales; f__Ruminococcaceae
1684	0	0	0	0	0	0	3	6	0	0	0	0	k__Bacteria; p__Gracilibacteria; c__uncultured_epsilon_proteobacterium; o__uncultured_epsilon_proteobacterium; f__uncultured_epsilon_proteobacterium; g__uncultured_epsilon_proteobacterium; s__uncultured_epsilon_proteobacterium
1643	0	0	0	0	0	0	0	0	3	0	0	0	k__Bacteria; p__Bacteroidetes; c__Sphingobacteriia; o__Sphingobacteriales; f__WCHB1_69
1602	0	0	0	0	0	0	7	0	8	0	0	0	k__Bacteria; p__Proteobacteria; c__Alphaproteobacteria; o__SAR11_clade; f__Chesapeake_Delaware_Bay
1233	0	0	0	0	0	0	0	0	0	4	6	0	k__Bacteria; p__Firmicutes; c__Clostridia; o__Clostridiales; f__Ruminococcaceae; g__Ruminococcaceae_UCG_014
1685	0	0	0	0	0	0	0	0	8	0	0	0	k__Bacteria; p__Proteobacteria; c__Alphaproteobacteria; o__Magnetococcales; f__Magnetococcaceae; g__Magnetococcus
1644	0	0	2	0	0	0	3	5	4	0	0	0	k__Bacteria; p__Proteobacteria; c__Gammaproteobacteria; o__Alteromonadales; f__Colwelliaceae; g__Thalassotalea; s__uncultured_bacterium
1603	0	0	0	0	0	0	5	0	0	0	0	0	k__Bacteria; p__Proteobacteria; c__Gammaproteobacteria
1275	0	0	0	0	0	0	0	0	0	0	5	0	k__Bacteria; p__Firmicutes; c__Clostridia; o__Clostridiales;

													f__Lachnospiraceae; g__Roseburia; s__uncultured_Clostridiales_bacterium
1234	0	0	0	0	0	0	0	0	0	8	0	0	k__Bacteria; p__Firmicutes; c__Clostridia; o__Clostridiales; f__Lachnospiraceae; g__uncultured
1686	0	0	0	0	0	0	3	6	0	4	0	0	k__Bacteria; p__Proteobacteria; c__Gammaproteobacteria; o__Vibrionales; f__Vibrionaceae; g__Photobacterium
1645	0	0	0	0	0	0	2	4	0	0	0	0	k__Bacteria; p__Spirochaetae; c__Spirochaetes; o__Spirochaetales; f__Spirochaetaceae; g__Spirochaeta_2
1604	0	0	0	0	0	0	5	0	2	0	0	0	k__Bacteria; p__Proteobacteria; c__Deltaproteobacteria; o__Desulfobacterales; f__Desulfobacteraceae
1276	0	0	0	0	0	0	0	0	0	8	4	1	k__Bacteria; p__Proteobacteria; c__Gammaproteobacteria; o__Aeromonadales; f__Aeromonadaceae; g__Oceanimonas; Ambiguous_taxa
1235	0	0	0	0	0	0	0	0	0	0	12	4	k__Bacteria; p__Firmicutes; c__Clostridia; o__Clostridiales; f__Ruminococcaceae; g__Anaerotruncus; s__uncultured_bacterium
1605	0	0	0	0	0	0	5	2	7	0	0	0	k__Bacteria; p__Proteobacteria; c__Gammaproteobacteria; o__Xanthomonadales; f__uncultured
1277	0	0	0	0	0	0	1	0	0	5	1	0	k__Bacteria; p__Proteobacteria; c__Gammaproteobacteria; o__Aeromonadales; f__Succinivibrionaceae; g__Succinivibrio; s__uncultured_bacterium
1236	0	1	0	0	0	0	0	0	0	0	9	20	k__Bacteria; p__Firmicutes; c__Clostridia; o__Clostridiales; f__Lachnospiraceae; g__uncultured
1606	0	0	0	0	0	0	9	10	0	0	0	0	k__Bacteria; p__Proteobacteria; c__Gammaproteobacteria
1278	0	0	0	3	2	0	0	0	1	3	0	1	k__Bacteria; p__Bacteroidetes
1237	0	0	0	0	0	0	4	5	0	6	1	0	k__Bacteria; p__Actinobacteria; c__Actinobacteria;

													o__Micrococcales; f__Micrococcaceae; g__Micrococcus
1689	8	1	12	0	0	0	2	0	0	0	0	0	k__Bacteria; p__Proteobacteria; c__Deltaproteobacteria; o__Desulfobacterales; f__Desulfobacteraceae; g__Sva0081_sediment_group; s__uncultured_delta_proteobacterium
1607	0	0	0	0	0	0	9	0	2	0	0	0	k__Bacteria; p__Bacteroidetes; c__Flavobacteriia; o__Flavobacteriales; f__Cryomorphaceae; g__Owenweeksia
1279	0	0	0	0	0	0	21	29	16	0	0	0	k__Bacteria; p__Fibrobacteres; c__Fibrobacteria; o__Fibrobacteria_Incertae_Sedis; f__Unknown_Family; g__possible_genus_03; s__uncultured_bacterium
1238	0	0	0	0	0	0	55	0	0	2	3	6	k__Bacteria; p__Firmicutes; c__Clostridia; o__Clostridiales; f__Lachnospiraceae
1608	0	0	0	0	0	0	12	7	13	0	0	0	k__Bacteria; p__Proteobacteria; c__Deltaproteobacteria; o__Desulfobacterales; f__Desulfobacteraceae
1239	0	0	0	0	4	0	0	0	0	13	10	6	k__Bacteria; p__Firmicutes; c__Clostridia; o__Clostridiales; f__Lachnospiraceae; g__Lachnospiraceae_FCS020_group; s__unidentified
1609	0	0	0	0	0	0	6	3	4	0	0	0	k__Bacteria; p__Bacteroidetes; c__Flavobacteriia; o__Flavobacteriales; f__Cryomorphaceae; g__Crocinitomix
490	0	0	0	3	17	7	0	0	0	0	0	0	k__Bacteria; p__Proteobacteria; c__Gammaproteobacteria; o__Thiotrichales; f__Piscirickettsiaceae; g__endosymbionts; s__endosymbiont_of_Alviniconcha_sp._type1
860	5	0	0	0	0	0	2	0	0	0	0	0	k__Bacteria; p__Proteobacteria; c__Deltaproteobacteria; o__Desulfobacterales; f__Desulfobacteraceae; g__uncultured
491	0	0	0	0	31	0	0	0	0	5	20	16	k__Bacteria; p__Firmicutes; c__Clostridia; o__Clostridiales; f__Lachnospiraceae

450	10	4	45	3	29	7	0	0	0	0	0	0	k__Bacteria; p__Proteobacteria; c__Gammaproteobacteria; o__Chromatiales; f__Ectothiorhodospiraceae; g__Thiogranum; Ambiguous_taxa
861	4	47	4	0	0	0	0	0	0	0	0	0	k__Bacteria; p__Proteobacteria; c__Gammaproteobacteria; o__Oceanospirillales; f__Alcanivoracaceae; g__Pleionea; Ambiguous_taxa
820	24	0	0	0	0	0	0	0	0	0	0	0	k__Bacteria; p__Proteobacteria; c__Deltaproteobacteria; o__Desulfobacterales; f__Desulfobulbaceae
492	0	0	0	0	16	0	0	0	0	2	4	11	k__Bacteria; p__Firmicutes; c__Clostridia; o__Clostridiales; f__Clostridiales_vadinBB60_group; g__uncultured_bacterium; s__uncultured_bacterium
451	20	114	140	14	4	30	0	0	0	0	0	0	k__Bacteria; p__Bacteroidetes; c__Flavobacteriia; o__Flavobacteriales; f__Flavobacteriaceae; g__Polaribacter; Ambiguous_taxa
410	0	0	0	41	0	0	0	0	0	0	0	0	k__Bacteria; p__Firmicutes; c__Clostridia; o__Clostridiales; f__Lachnospiraceae
862	6	44	22	0	2	1	0	0	0	0	0	0	k__Bacteria; p__Bacteroidetes; c__Flavobacteriia; o__Flavobacteriales; f__Flavobacteriaceae; g__Muriicola; s__uncultured_Bacteroidetes_bacterium
821	1	0	1	0	0	0	0	0	0	0	0	0	k__Bacteria; p__Proteobacteria; c__Gammaproteobacteria
493	0	0	0	0	35	0	0	0	0	0	2	1	k__Bacteria; p__Firmicutes; c__Clostridia; o__Clostridiales; f__Ruminococcaceae; g__Ruminococcaceae_UCG_014
452	0	0	0	2	0	0	0	0	0	0	0	0	k__Bacteria; p__Proteobacteria
411	0	0	0	45	18	52	0	31	0	4	3	0	k__Bacteria; p__Bacteroidetes; c__Bacteroidia; o__Bacteroidales; f__Bacteroidales_S24_7_group; g__uncultured_bacterium; s__uncultured_bacterium

863	6	0	15	10	0	0	0	0	0	0	0	0	k__Bacteria; p__Proteobacteria; c__Gammaproteobacteria; o__Order_Incertae_Sedis; f__Family_Incertae_Sedis; g__Marinicella
822	19	0	0	0	0	0	0	0	0	0	0	0	k__Bacteria; p__Bacteroidetes; c__Sphingobacteriia; o__Sphingobacteriales; f__Chitinophagaceae; g__Chitinophaga; s__Terrimonas_sp._16_45A
494	1	91	0	0	19	0	0	0	0	0	11	15	k__Bacteria; p__Firmicutes; c__Clostridia; o__Clostridiales; f__Ruminococcaceae; g__Anaerotruncus; s__uncultured_bacterium
453	8	45	41	22	2	2	0	0	0	0	0	0	k__Bacteria; p__Proteobacteria; c__Gammaproteobacteria; o__BD7_8_marine_group
412	5	20	61	16	0	1	0	0	1	0	0	0	k__Bacteria; p__Proteobacteria; c__Gammaproteobacteria; o__Cellvibrionales; f__Halieaceae; g__Haliea
864	8	48	19	0	0	0	0	0	1	0	0	0	k__Bacteria; p__Proteobacteria; c__Gammaproteobacteria; o__Alteromonadales; f__Pseudoalteromonadaceae
823	9	18	46	3	0	0	11	7	10	0	0	0	k__Bacteria; p__Fibrobacteres; c__Fibrobacteria; o__Fibrobacteria_Incertae_Sedis; f__Unknown_Family; g__possible_genus_03; s__uncultured_bacterium
495	0	0	0	1	3	0	0	0	0	0	0	0	k__Bacteria; p__Proteobacteria; c__Deltaproteobacteria; o__Bdellovibrionales; f__Bdellovibrionaceae; g__Bdellovibrio
454	208	635	1172	10	17	27	344	250	242	0	0	0	k__Bacteria; p__Proteobacteria; c__Epsilonproteobacteria; o__Campylobacterales; f__Helicobacteraceae; g__Sulfurovum
413	32	85	90	2	0	10	0	2	0	0	0	0	k__Bacteria; p__Bacteroidetes; c__Sphingobacteriia; o__Sphingobacteriales; f__Saprospiraceae; g__uncultured
865	1	2	1	1	0	0	0	0	0	0	0	0	k__Bacteria; p__Proteobacteria; c__Deltaproteobacteria; o__Desulfobacterales; f__Desulfobulbaceae

824	19	0	2	3	0	0	0	0	0	0	0	0	k__Bacteria; p__Acidobacteria; c__Holophagae; o__Subgroup_10; f__Sva0725
496	502	301	0	3	43	0	0	175	57	79	39	23	k__Bacteria; p__Firmicutes; c__Clostridia; o__Clostridiales; f__Lachnospiraceae; g__Lachnospiraceae_NK4A136_group; Ambiguous_taxa
455	2	3	36	17	3	0	0	0	0	0	0	0	k__Bacteria; p__Acidobacteria; c__Holophagae; o__Subgroup_10; f__Sva0725; g__uncultured_Acidobacteria_bacterium; s__uncultured_Acidobacteria_bacterium
414	0	0	0	43	0	0	1	1	0	0	0	0	k__Bacteria; p__Firmicutes; c__Clostridia; o__Clostridiales; f__Ruminococcaceae; g__Ruminiclostridium_6; Ambiguous_taxa
866	15	51	68	1	0	0	0	0	0	3	4	1	k__Bacteria; p__Firmicutes; c__Clostridia; o__Clostridiales; f__Family_XII; g__Fusibacter; Ambiguous_taxa
825	13	0	6	0	0	0	0	0	0	0	0	0	k__Bacteria; p__Proteobacteria; c__Alphaproteobacteria; o__Rhodospirillales; f__Rhodospirillaceae; g__uncultured; s__alpha_proteobacterium_SOGA29
497	0	0	0	1	5	0	30	58	0	15	53	30	k__Bacteria; p__Bacteroidetes; c__Bacteroidia; o__Bacteroidales; f__Bacteroidales_S24_7_group; g__uncultured_bacterium; s__uncultured_bacterium
456	2	33	15	11	3	9	0	0	0	0	0	0	k__Bacteria; p__Bacteroidetes; c__Flavobacteriia; o__Flavobacteriales; f__Flavobacteriaceae
415	0	0	10	3	0	1	0	0	0	0	0	0	k__Bacteria; p__Proteobacteria; c__Deltaproteobacteria; o__Desulfobacterales; f__Desulfobulbaceae; g__Desulforhopalus; s__uncultured_bacterium

867	5	11	7	0	0	0	0	0	0	0	0	0	k__Bacteria; p__Bacteroidetes; c__Flavobacteriia; o__Flavobacteriales; f__Flavobacteriaceae; g__Psychroflexus; s__uncultured_organism
826	8	0	2	0	0	0	0	0	0	0	0	0	k__Bacteria; p__Proteobacteria; c__Deltaproteobacteria; o__Myxococcales; f__Haliangiaceae; g__Haliangium
498	0	0	0	0	39	0	0	0	0	20	13	0	k__Bacteria; p__Firmicutes; c__Clostridia; o__Clostridiales; f__Lachnospiraceae
457	92	484	457	8	2	3	0	5	0	0	0	0	k__Bacteria; p__Proteobacteria; c__Epsilonproteobacteria; o__Campylobacterales; f__Campylobacteraceae; g__Arcobacter; Ambiguous_taxa
416	0	0	0	47	0	0	0	0	0	0	0	0	k__Bacteria; p__Actinobacteria; c__Coriobacteriia; o__Coriobacteriales; f__Coriobacteriaceae; g__Senegalimassilia; s__uncultured_bacterium
868	14	0	0	0	0	0	0	0	0	0	0	0	k__Bacteria; p__Proteobacteria; c__Alphaproteobacteria; o__Sphingomonadales; f__Erythrobacteraceae; g__Erythrobacter; Ambiguous_taxa
827	4	3	13	0	0	0	0	0	2	0	0	0	k__Bacteria; p__Tenericutes; c__Mollicutes; o__NB1_n; Ambiguous_taxa; Ambiguous_taxa; Ambiguous_taxa
499	0	0	0	0	53	0	0	0	56	0	0	0	k__Bacteria; p__Firmicutes; c__Clostridia; o__Clostridiales; f__Lachnospiraceae; g__Lachnospiraceae_NK4B4_group; s__uncultured_bacterium
458	0	3	3	5	0	0	0	0	0	16	1	1	k__Bacteria; p__Bacteroidetes; c__Bacteroidia; o__Bacteroidales; f__Marinilabiaceae; g__Saccharicrinis; Ambiguous_taxa
417	0	0	0	42	31	0	0	0	0	0	2	0	k__Bacteria; p__Firmicutes; c__Clostridia; o__Clostridiales; f__Ruminococcaceae; g__Ruminiclostridium_9

869	6	42	43	1	0	0	0	2	4	0	0	0	k__Bacteria; p__Proteobacteria; c__Deltaproteobacteria; o__Desulfobacterales; f__Desulfobulbaceae
828	51	121	159	0	0	0	94	87	107	7	0	0	k__Bacteria; p__Proteobacteria; c__Gammaproteobacteria; o__Vibrionales; f__Vibrionaceae; g__Vibrio
459	12	15	10	5	12	35	0	0	0	0	0	0	k__Bacteria; p__Proteobacteria; c__Deltaproteobacteria; o__Desulfobacterales; f__Desulfobulbaceae
418	0	99	0	28	0	0	0	0	0	20	29	13	k__Bacteria; p__Firmicutes; c__Clostridia; o__Clostridiales; f__Ruminococcaceae; g__Anaerotruncus; Ambiguous_taxa
829	11	0	0	0	0	0	0	0	0	0	0	0	k__Bacteria
419	0	0	0	12	0	0	0	0	0	0	0	0	k__Bacteria; p__Firmicutes; c__Bacilli; o__Lactobacillales; f__Lactobacillaceae; g__Lactobacillus; s__Lactobacillus_curvatus
1570	9	0	6	0	0	0	5	1	15	0	0	0	k__Bacteria; p__Proteobacteria; c__Gammaproteobacteria; o__Arenicellales; f__Arenicellaceae; g__uncultured_gamma_proteobacterium; s__uncultured_gamma_proteobacterium
1160	0	0	4	1	0	0	7	0	0	0	0	0	k__Bacteria; p__Acidobacteria; c__Holophagae; o__Acanthopleuribacterales; f__Acanthopleuribacteraceae; g__Acanthopleuribacter; s__uncultured_bacterium
1571	0	0	0	0	0	0	16	0	20	0	0	0	k__Bacteria; p__Proteobacteria; c__Gammaproteobacteria
1530	0	0	0	0	0	0	54	15	41	0	0	0	k__Bacteria; p__Proteobacteria; c__Epsilonproteobacteria; o__Campylobacterales; f__Helicobacteraceae; g__Sulfurimonas
1161	1	0	7	0	1	0	0	0	0	0	0	0	k__Bacteria; p__Proteobacteria; c__Gammaproteobacteria; o__Gammaproteobacteria_Incertae_Sedis; f__Unknown_Family; g__Sedimenticola
1120	0	3	30	0	0	0	0	7	0	0	0	0	k__Bacteria; p__Proteobacteria; c__Deltaproteobacteria;

													o__Desulfobacterales; f__Desulfobacteraceae
1572	0	0	0	0	0	0	2	2	8	0	0	0	k__Bacteria; p__Proteobacteria; c__Deltaproteobacteria; o__Myxococcales; f__MidBa8
1531	0	0	0	0	0	0	64	0	0	0	0	0	k__Bacteria; p__Proteobacteria; c__Gammaproteobacteria; o__Xanthomonadales; f__Xanthomonadales_Incertae_Sedis; g__Acidibacter; s__uncultured_bacterium
1162	0	0	11	0	0	0	0	0	0	0	0	0	k__Bacteria; p__Proteobacteria; c__Deltaproteobacteria
1121	0	0	4	0	0	0	0	0	0	0	0	0	k__Bacteria; p__Bacteroidetes; c__Sphingobacteriia; o__Sphingobacteriales; f__Saprospiraceae; g__uncultured; s__uncultured_bacterium
1573	0	0	0	0	0	0	2	4	1	0	0	0	k__Bacteria; p__Actinobacteria; c__Actinobacteria
1532	0	0	0	0	0	0	10	10	20	0	0	0	k__Bacteria; p__Proteobacteria; c__Gammaproteobacteria
1163	0	0	5	0	0	0	0	0	0	0	0	0	k__Bacteria; p__Bacteroidetes; c__Flavobacteriia; o__Flavobacteriales; f__Cryomorphaceae; g__Owenweeksia
1122	0	0	7	0	0	0	0	0	0	0	0	0	k__Bacteria; p__Proteobacteria; c__Deltaproteobacteria; o__Desulfarculales; f__Desulfarculaceae; g__Desulfatiglans
1574	0	0	0	0	0	0	46	0	0	0	1	0	k__Bacteria; p__Firmicutes; c__Clostridia; o__Clostridiales; f__Lachnospiraceae; g__Roseburia
1533	0	0	0	0	0	0	14	10	7	0	0	0	k__Bacteria; p__Proteobacteria; c__Deltaproteobacteria; o__Desulfobacterales; f__Desulfobacteraceae
1164	0	0	13	0	0	0	0	0	0	0	0	0	k__Bacteria; p__Bacteroidetes; c__Sphingobacteriia; o__Sphingobacteriales; f__Chitinophagaceae; g__Flavisolibacter
1123	0	0	8	0	1	2	0	0	0	0	0	0	k__Bacteria; p__Proteobacteria; c__Gammaproteobacteria; o__Cellvibrionales; f__Spongiibacteraceae; g__BD1_7_clade; s__uncultured_bacterium

1575	0	0	0	0	0	0	20	0	0	0	0	0	k__Bacteria; p__Bacteroidetes; c__Sphingobacteriia; o__Sphingobacteriales; f__Saprospiraceae; g__Lewinella; Ambiguous_taxa
1534	0	0	0	0	0	0	12	14	10	0	0	0	k__Bacteria; p__Bacteroidetes; c__Bacteroidia; o__Bacteroidia_Incertae_Sedis; f__Draconibacteriaceae; g__Draconibacterium
1165	0	0	27	0	0	0	0	0	0	7	0	0	k__Bacteria; p__Firmicutes; c__Clostridia; o__Clostridiales; f__Clostridiales_vadinBB60_group; g__uncultured_bacterium; s__uncultured_bacterium
1124	0	6	4	0	0	0	0	0	0	0	1	0	k__Bacteria; p__Proteobacteria; c__Gammaproteobacteria; o__Vibrionales; f__Vibrionaceae; g__Vibrio
1576	0	0	0	0	0	0	12	0	1	0	0	0	k__Bacteria; p__Proteobacteria; c__Epsilonproteobacteria; o__Campylobacterales; f__Helicobacteraceae; g__Sulfurimonas
1535	0	0	0	0	0	0	4	12	0	0	0	0	k__Bacteria; p__Proteobacteria; c__Alphaproteobacteria; o__Rhodospirillales; f__Rhodospirillaceae; g__uncultured
1166	1	0	7	0	0	0	0	0	0	0	1	4	k__Bacteria; p__Proteobacteria; c__Gammaproteobacteria; o__Oceanospirillales; f__Oceanospirillaceae; g__Oleibacter
1125	0	1	17	0	0	0	0	0	0	0	0	0	k__Bacteria; p__Proteobacteria; c__Gammaproteobacteria; o__Alteromonadales; f__Alteromonadaceae; g__Neiella; Ambiguous_taxa
1577	0	0	0	0	0	0	15	3	0	0	0	0	k__Bacteria; p__Proteobacteria; c__Alphaproteobacteria; o__Rhodospirillales; f__Rhodospirillaceae
1536	0	0	0	0	0	0	20	21	3	0	0	0	k__Bacteria; p__Proteobacteria; c__Gammaproteobacteria; o__Cellvibrionales; f__Spongiibacteraceae; g__BD1_7_clade
1167	0	0	6	0	0	0	0	0	0	0	0	0	k__Bacteria; p__Chlorobi; c__Ignavibacteria; o__Ignavibacteriales; f__IheB3_7; g__uncultured_bacterium;

													s__uncultured_bacterium
1126	6	36	47	5	5	0	3	0	0	0	0	0	k__Bacteria; p__Proteobacteria; c__Alphaproteobacteria; o__Rhodobacterales; f__Rhodobacteraceae; g__Sulfitobacter
1578	0	5	0	0	0	0	14	34	9	0	0	0	k__Bacteria; p__Proteobacteria; c__Gammaproteobacteria; o__Oceanospirillales; f__Oleiphilaceae; g__Oleiphilus
1537	0	0	0	0	0	0	27	1	15	0	1	0	k__Bacteria; p__Bacteroidetes; c__Cytophagia; o__Cytophagales; f__Flammeovirgaceae; g__Reichenbachiella; Ambiguous_taxa
1168	0	0	6	0	0	0	0	0	0	0	0	0	k__Bacteria; p__Proteobacteria; c__Epsilonproteobacteria; o__Campylobacterales; f__Helicobacteraceae; g__Rs_M59_termite_group; s__uncultured_bacterium
1127	0	0	7	0	0	0	0	0	0	0	0	0	k__Bacteria; p__Proteobacteria; c__Deltaproteobacteria; o__Desulfuromonadales; f__GR_WP33_58
1579	0	0	0	0	0	0	5	10	3	0	0	0	k__Bacteria; p__Proteobacteria; c__Milano_WF1B_44; o__uncultured_gamma_proteobacterium; f__uncultured_gamma_proteobacterium; g__uncultured_gamma_proteobacterium; s__uncultured_gamma_proteobacterium
1538	0	0	0	1	0	0	11	0	0	0	0	0	k__Bacteria; p__Proteobacteria; c__Alphaproteobacteria; o__Rhizobiales
1169	0	0	5	0	0	0	0	0	0	0	0	0	k__Bacteria; p__Proteobacteria; c__Deltaproteobacteria; o__Desulfobacterales; f__Desulfobulbaceae; g__Desulfobulbus
1128	0	0	6	0	0	0	0	0	0	0	0	0	k__Bacteria; p__Bacteroidetes; c__Cytophagia; o__Cytophagales; f__Flammeovirgaceae; g__Cesiribacter; Ambiguous_taxa

1539	0	0	0	0	0	0	12	10	13	0	0	0	k__Bacteria; p__Proteobacteria; c__Epsilonproteobacteria; o__Campylobacterales; f__Helicobacteraceae; g__Sulfurimonas
1129	0	2	4	0	0	0	0	0	0	0	0	0	k__Bacteria; p__Proteobacteria; c__Deltaproteobacteria; o__Desulfuromonadales
790	3	0	5	0	0	0	5	15	11	0	0	0	k__Bacteria; p__Proteobacteria; c__Epsilonproteobacteria; o__Campylobacterales; f__Helicobacteraceae; g__Sulfurimonas
380	0	0	0	41	0	0	40	0	0	15	18	10	k__Bacteria; p__Proteobacteria; c__Deltaproteobacteria; o__Desulfovibrionales; f__Desulfovibrionaceae; g__Desulfovibrio; s__unidentified
791	16	28	11	0	0	0	7	11	15	1	0	0	k__Bacteria; p__Fibrobacteres; c__Fibrobacteria; o__Fibrobacteria_Incertae_Sedis; f__Unknown_Family; g__possible_genus_03
750	0	4	35	1	0	0	14	30	18	10	3	2	k__Bacteria; p__Bacteroidetes; c__Bacteroidia; o__Bacteroidales; f__Marinilabiaceae; g__Carboxylicivirga; s__uncultured_bacterium
381	0	0	0	59	0	0	0	0	36	33	71	7	k__Bacteria; p__Firmicutes; c__Clostridia; o__Clostridiales; f__Lachnospiraceae
340	0	1	6	15	0	0	0	0	0	0	0	0	k__Bacteria; p__Proteobacteria; c__Gammaproteobacteria; o__Chromatiales; f__Ectothiorhodospiraceae; Ambiguous_taxa; Ambiguous_taxa
792	16	14	46	2	4	2	3	0	0	0	0	0	k__Bacteria; p__Proteobacteria; c__Deltaproteobacteria; o__Myxococcales; f__Sandaracinaceae; g__uncultured
751	0	9	1	0	0	0	0	0	0	0	0	0	k__Bacteria; p__Proteobacteria; c__Deltaproteobacteria; o__Myxococcales; f__Blfdi19; g__uncultured_delta_proteobacterium; s__uncultured_delta_proteobacterium

710	100	237	214	2	19	2	10	16	16	0	0	0	k__Bacteria; p__Bacteroidetes; c__Flavobacteriia; o__Flavobacteriales; f__Flavobacteriaceae; g__Tenacibaculum
382	0	0	1	66	36	0	0	1	0	26	13	3	k__Bacteria; p__Firmicutes; c__Clostridia; o__Clostridiales; f__Ruminococcaceae; g__Ruminiclostridium_9; s__unidentified
341	0	0	33	33	37	0	0	1	0	34	53	2	k__Bacteria; p__Proteobacteria; c__Gammaproteobacteria; o__Enterobacteriales; f__Enterobacteriaceae; g__Proteus; s__Proteus_mirabilis
300	159	0	19	151	81	98	93	108	84	0	13	5	k__Bacteria; p__Bacteroidetes; c__Bacteroidia; o__Bacteroidales; f__Porphyromonadaceae; g__Odoribacter; s__uncultured_bacterium
793	5	2	61	0	2	0	3	1	11	0	0	0	k__Bacteria; p__Proteobacteria; c__Deltaproteobacteria; o__Desulfuromonadales; f__Desulfuromonadaceae
752	0	4	2	0	0	0	0	0	0	0	0	0	k__Bacteria; p__Proteobacteria; c__Gammaproteobacteria
711	6	2	50	0	0	0	0	0	0	0	0	0	k__Bacteria; p__Proteobacteria; c__Gammaproteobacteria; o__Chromatiales; f__Ectothiorhodospiraceae; g__uncultured_gamma_proteobacterium; s__uncultured_gamma_proteobacterium
383	0	0	1	7	1	0	2	0	1	0	0	0	k__Bacteria; p__Proteobacteria; c__Gammaproteobacteria; o__Chromatiales; f__Granulosicoccaceae; g__Granulosicoccus; Ambiguous_taxa
342	0	0	0	65	0	97	0	0	0	36	18	36	k__Bacteria; p__Firmicutes; c__Clostridia; o__Clostridiales; f__Lachnospiraceae; g__uncultured
301	103	0	38	0	0	69	0	0	45	3	27	7	k__Bacteria; p__Firmicutes; c__Clostridia; o__Clostridiales; f__Lachnospiraceae; g__Lachnospiraceae_NK4A136_group; s__uncultured_bacterium
794	4	3	19	0	0	0	8	0	0	0	0	0	k__Bacteria; p__Bacteroidetes; c__Sphingobacteriia;

													o__Sphingobacteriales; f__WCHB1_69
753	3	73	45	0	0	0	3	4	0	0	0	0	k__Bacteria; p__Bacteroidetes; c__Flavobacteriia; o__Flavobacteriales; f__Cryomorphaceae; g__Crocinitomix; s__uncultured_bacterium
712	123	651	691	0	0	0	1	0	0	0	0	0	k__Bacteria; p__Bacteroidetes; c__Flavobacteriia; o__Flavobacteriales; f__Flavobacteriaceae; g__Lutibacter; Ambiguous_taxa
384	0	0	0	3	0	0	0	0	0	1	0	1	k__Bacteria; p__Proteobacteria; c__Alphaproteobacteria; o__Rhodospirillales; f__Rhodospirillaceae; g__Thalassospira; s__Blattella_germanica_(German_cockroach)
343	0	88	1	121	169	23	180	0	181	56	100	46	k__Bacteria; p__Bacteroidetes; c__Bacteroidia; o__Bacteroidales; f__Bacteroidales_S24_7_group; g__uncultured_bacterium; s__uncultured_bacterium
302	1	0	0	5	14	12	0	0	0	0	0	0	k__Bacteria; p__Proteobacteria; c__Gammaproteobacteria; o__Alteromonadales; f__Colwelliaceae; g__Thalassotalea; Ambiguous_taxa
795	16	8	24	0	3	0	0	1	1	0	0	0	k__Bacteria; p__Proteobacteria; c__Gammaproteobacteria; o__Gammaproteobacteria_Incertae_Sedis; f__Unknown_Family; g__Sedimenticola; Ambiguous_taxa
754	181	498	561	0	1	0	317	268	379	18	9	1	k__Bacteria; p__Proteobacteria; c__Gammaproteobacteria; o__Vibrionales; f__Vibrionaceae; g__Vibrio
713	79	309	317	2	0	1	3	0	1	28	10	7	k__Bacteria; p__Proteobacteria; c__Epsilonproteobacteria; o__Campylobacterales; f__Campylobacteraceae; g__Arcobacter; Ambiguous_taxa
385	1	1	9	6	0	7	0	0	0	0	0	0	k__Bacteria; p__Bacteroidetes; c__Flavobacteriia; o__Flavobacteriales; f__Cryomorphaceae

344	0	0	0	65	0	0	0	0	0	0	0	0	k__Bacteria; p__Firmicutes; c__Clostridia; o__Clostridiales; f__Lachnospiraceae
303	0	2	29	44	13	5	13	29	8	2	1	2	k__Bacteria; p__Proteobacteria; c__Gammaproteobacteria; o__Pseudomonadales; f__Pseudomonadaceae; g__Pseudomonas
796	3	17	6	0	3	3	0	0	0	0	0	0	k__Bacteria; p__Acidobacteria; c__Subgroup_22
755	0	0	3	0	0	0	0	0	0	0	0	0	k__Bacteria; p__Proteobacteria; c__Alphaproteobacteria; o__Rhodobacterales; f__Rhodobacteraceae
714	1	17	15	0	0	0	0	0	0	0	0	0	k__Bacteria; p__Proteobacteria; c__Deltaproteobacteria; o__Desulfobacterales; f__Desulfobulbaceae; g__uncultured; s__uncultured_delta_proteobacterium
386	0	0	0	5	0	2	0	0	0	0	0	0	k__Bacteria; p__Bacteroidetes
345	0	0	0	31	0	0	0	0	1	6	9	16	k__Bacteria; p__Firmicutes; c__Clostridia; o__Clostridiales; f__Ruminococcaceae; g__Oscillibacter; s__uncultured_bacterium
304	14	16	56	15	17	7	0	0	0	0	0	0	k__Bacteria; p__Proteobacteria; c__Gammaproteobacteria; o__Oceanospirillales; f__Hahellaceae; g__Endozoicomonas
797	2	0	0	0	0	0	0	0	0	0	0	0	k__Bacteria; p__Proteobacteria; c__Deltaproteobacteria; o__Bdellovibrionales; f__Bdellovibrionaceae; g__Bdellovibrio; s__uncultured_bacterium
756	1	6	35	0	0	0	0	0	0	0	0	0	k__Bacteria; p__Proteobacteria; c__Deltaproteobacteria; o__Bdellovibrionales; f__Bdellovibrionaceae; g__OM27_clade
715	53	63	132	0	0	0	20	9	16	0	0	0	k__Bacteria; p__Proteobacteria; c__Gammaproteobacteria
387	234	215	75	285	147	55	14	391	134	541	45	19	k__Bacteria; p__Bacteroidetes; c__Bacteroidia; o__Bacteroidales; f__Prevotellaceae; g__Prevotella_9
346	0	0	40	39	0	0	81	0	0	0	13	0	k__Bacteria; p__Firmicutes; c__Clostridia; o__Clostridiales; f__Ruminococcaceae; g__Ruminococcaceae_UCG_014

305	1	0	2	15	0	4	0	0	1	0	0	0	k__Bacteria; p__Proteobacteria; c__Gammaproteobacteria; o__Gammaproteobacteria_Incertae_Sedis; f__Unknown_Family; g__Sedimenticola; Ambiguous_taxa
798	1	1	12	0	4	0	0	0	0	0	0	0	k__Bacteria; p__Proteobacteria; c__Gammaproteobacteria; o__Xanthomonadales; f__JTB255_marine_benthic_group; g__uncultured_sediment_bacterium; s__uncultured_sediment_bacterium
757	0	6	0	2	0	3	0	0	0	0	0	0	k__Bacteria; p__Bacteroidetes; c__Flavobacteriia; o__Flavobacteriales; f__Flavobacteriaceae; g__Maritimimonas; Ambiguous_taxa
716	23	58	94	7	4	13	18	29	33	3	0	0	k__Bacteria; p__Proteobacteria; c__Gammaproteobacteria; o__Alteromonadales; f__Colwelliaceae; g__Thalassotalea; Ambiguous_taxa
388	27	30	62	18	20	41	8	3	7	0	0	0	k__Bacteria; p__Proteobacteria; c__Deltaproteobacteria; o__Desulfobacterales; f__Desulfobacteraceae; g__Sva0081_sediment_group
347	479	290	0	95	153	104	0	62	66	31	142	21	k__Bacteria; p__Bacteroidetes; c__Bacteroidia; o__Bacteroidales; f__Bacteroidales_S24_7_group; g__uncultured_bacterium; s__uncultured_bacterium
306	0	0	5	0	0	11	0	0	0	0	0	0	k__Bacteria; p__Bacteroidetes; c__Sphingobacteriia; o__Sphingobacteriales; f__Saprospiraceae; g__Portibacter; s__uncultured_Bacteroidetes_bacterium
799	22	17	15	5	3	0	0	0	0	0	0	0	k__Bacteria; p__Bacteroidetes; c__Flavobacteriia; o__Flavobacteriales; f__Cryomorphaceae; g__Fluviicola
758	20	0	4	0	0	1	0	0	0	0	0	0	k__Bacteria; p__Gracilibacteria

717	26	66	85	2	1	0	1	0	0	0	0	0	k__Bacteria; p__Fibrobacteres; c__Fibrobacteria; o__Fibrobacteria_Incertae_Sedis; f__Unknown_Family; g__possible_genus_03; s__uncultured_bacterium
389	0	0	32	88	0	0	0	0	0	0	6	0	k__Bacteria; p__Firmicutes; c__Clostridia; o__Clostridiales; f__Ruminococcaceae; g__Ruminococcaceae_NK4A214_group
348	0	0	0	57	0	51	0	0	0	8	0	0	k__Bacteria; p__Firmicutes; c__Clostridia; o__Clostridiales; f__Ruminococcaceae; g__Oscillibacter
307	0	24	35	12	1	2	0	0	5	0	0	0	k__Bacteria; p__Bacteroidetes; c__Bacteroidia; o__Bacteroidia_Incertae_Sedis; f__Draconibacteriaceae
759	2	19	41	1	2	15	6	20	6	1	0	0	k__Bacteria; p__Proteobacteria; c__Gammaproteobacteria; o__Cellvibrionales; f__Halieaceae
718	21	117	118	0	0	6	0	0	2	0	0	0	k__Bacteria; p__Bacteroidetes; c__Flavobacteriia; o__Flavobacteriales; f__Flavobacteriaceae
349	0	121	0	107	65	0	0	0	81	0	30	13	k__Bacteria; p__Firmicutes; c__Erysipelotrichia; o__Erysipelotrichales; f__Erysipelotrichaceae; g__uncultured; Ambiguous_taxa
308	1	2	4	0	0	0	2	2	9	0	0	0	k__Bacteria; p__Fibrobacteres; c__Fibrobacteria; o__Fibrobacteria_Incertae_Sedis; f__Unknown_Family; g__possible_genus_03
719	0	20	15	5	0	1	0	0	0	0	0	0	k__Bacteria; p__Bacteroidetes; c__Flavobacteriia; o__Flavobacteriales; f__Flavobacteriaceae
309	0	0	39	0	0	51	0	0	0	3	5	0	k__Bacteria; p__Firmicutes; c__Clostridia; o__Clostridiales; f__Lachnospiraceae; g__Roseburia; s__uncultured_bacterium
1090	0	0	7	0	0	0	0	0	0	0	0	0	k__Bacteria; p__Proteobacteria; c__Alphaproteobacteria; o__Rhodospirillales; f__Rhodospirillaceae; g__uncultured; Ambiguous_taxa

1460	0	0	0	0	0	0	0	0	0	3	0	0	k__Bacteria; p__Proteobacteria; c__Betaproteobacteria; o__Burkholderiales; f__Burkholderiaceae; g__Lautropia; Ambiguous_taxa
1050	0	7	4	0	0	0	0	0	0	0	0	0	k__Bacteria; p__Gracilibacteria; c__uncultured_deep_sea_bacterium; o__uncultured_deep_sea_bacterium; f__uncultured_deep_sea_bacterium; g__uncultured_deep_sea_bacterium; s__uncultured_deep_sea_bacterium
1091	0	0	6	0	0	0	0	0	0	0	0	0	k__Bacteria; p__Bacteroidetes; c__Bacteroidetes_BD2_2
1461	0	0	0	0	0	0	0	0	0	22	0	0	k__Bacteria; p__Firmicutes; c__Clostridia; o__Clostridiales; f__Lachnospiraceae
1420	0	0	0	0	0	0	0	0	0	22	0	0	k__Bacteria; p__Firmicutes; c__Clostridia; o__Clostridiales; f__Ruminococcaceae; g__Anaerofilum; s__uncultured_bacterium
1051	0	2	5	0	0	0	0	0	0	0	0	0	k__Bacteria; p__Proteobacteria; c__Gammaproteobacteria; o__Chromatiales; f__Chromatiaceae; g__Rheinheimera; Ambiguous_taxa
1010	0	7	9	0	0	0	0	0	0	0	0	0	k__Bacteria; p__Proteobacteria; c__Deltaproteobacteria; o__Syntrophobacterales; f__Syntrophobacteraceae; g__uncultured; Ambiguous_taxa
1092	0	0	17	0	0	0	0	0	0	0	0	0	k__Bacteria; p__Bacteroidetes; c__Sphingobacteriia; o__Sphingobacteriales; f__E6aC02; g__uncultured_Bacteroidetes_bacterium; s__uncultured_Bacteroidetes_bacterium
1462	0	0	0	0	0	0	0	1	0	14	1	1	k__Bacteria; p__Firmicutes; c__Clostridia; o__Clostridiales;

													f__Lachnospiraceae
1421	0	0	0	0	0	0	0	0	0	26	0	0	k__Bacteria; p__Firmicutes; c__Clostridia; o__Clostridiales; f__Ruminococcaceae; g__Ruminococcaceae_UCG_003
1052	0	5	7	0	0	0	0	0	0	0	0	0	k__Bacteria; p__Acidobacteria; c__Holophagae; o__Subgroup_10; f__Sva0725
1011	0	10	16	0	0	0	0	0	0	0	0	0	k__Bacteria; p__Acidobacteria; c__Holophagae; o__Subgroup_10; f__Sva0725
1093	0	0	4	0	0	0	0	0	0	0	0	0	k__Bacteria; p__Bacteroidetes; c__Sphingobacteriia; o__Sphingobacteriales; f__WCHB1_69; g__uncultured_Bacteroidetes_bacterium; s__uncultured_Bacteroidetes_bacterium
1463	0	0	0	0	0	0	75	61	79	1	0	0	k__Bacteria; p__Proteobacteria; c__Gammaproteobacteria; o__Oceanospirillales; f__SS1_B_06_26
1422	0	0	4	0	0	0	34	54	33	1	0	0	k__Bacteria; p__Bacteroidetes; c__Flavobacteriia; o__Flavobacteriales; f__Cryomorphaceae; g__Owenweeksia; Ambiguous_taxa
1053	0	2	2	0	0	0	3	0	0	1	0	0	k__Bacteria; p__Proteobacteria; c__Alphaproteobacteria; o__Rhodobacterales; f__Rhodobacteraceae; g__Labrenzia; Ambiguous_taxa
1012	0	12	53	0	0	0	0	1	0	0	0	0	k__Bacteria; p__Proteobacteria; c__Gammaproteobacteria; o__Order_Incertae_Sedis; f__Family_Incertae_Sedis; g__Marinicella; s__gamma_proteobacterium_N2yML2
1094	0	0	16	0	0	0	3	0	0	0	0	0	k__Bacteria; p__Chloroflexi; c__uncultured
1464	0	0	0	0	0	0	0	27	0	23	2	3	k__Bacteria; p__Firmicutes; c__Clostridia; o__Clostridiales; f__Ruminococcaceae

1423	0	0	0	0	0	1	0	1	1	64	4	0	k__Bacteria; p__Firmicutes; c__Clostridia; o__Clostridiales; f__Lachnospiraceae; g__Coprococcus_3; s__uncultured_bacterium
1013	0	11	0	0	0	0	5	0	0	0	0	0	k__Bacteria; p__Bacteroidetes; c__Sphingobacteriia; o__Sphingobacteriales; f__Saprospiraceae; g__uncultured
1095	0	0	13	0	0	0	0	0	0	0	0	0	k__Bacteria; p__Proteobacteria; c__Gammaproteobacteria
1054	1	9	25	0	0	3	1	0	0	0	0	0	k__Bacteria; p__Proteobacteria; c__Gammaproteobacteria
1465	0	0	0	0	0	0	0	0	0	8	0	0	k__Bacteria; p__Firmicutes; c__Clostridia; o__Clostridiales; f__Ruminococcaceae
1424	0	0	0	0	0	0	0	0	0	7	0	0	k__Bacteria; p__Firmicutes; c__Clostridia; o__Clostridiales; f__Ruminococcaceae; g__Ruminococcaceae_UCG_005
1014	0	14	6	0	0	0	2	24	1	0	0	0	k__Bacteria; p__Proteobacteria; c__Alphaproteobacteria; o__Rhodospirillales; f__Rhodospirillaceae; g__Pelagibius
1096	0	0	9	0	0	0	0	0	2	0	0	0	k__Bacteria; p__Proteobacteria; c__Alphaproteobacteria; o__Rhizobiales; f__Hyphomicrobiaceae
1055	0	6	1	0	0	0	0	0	0	0	0	0	k__Bacteria; p__Bacteroidetes; c__Sphingobacteriia; o__Sphingobacteriales; f__Saprospiraceae; g__uncultured
1466	0	0	1	1	0	0	0	0	0	46	0	2	k__Bacteria; p__Firmicutes; c__Clostridia; o__Clostridiales; f__Lachnospiraceae; g__Lachnospiraceae_NK4A136_group
1425	0	0	0	0	0	0	0	0	0	28	0	4	k__Bacteria; p__Firmicutes; c__Clostridia; o__Clostridiales; f__Lachnospiraceae
1015	0	12	0	0	0	0	0	0	0	0	0	0	k__Bacteria
1097	0	0	18	0	0	0	0	0	0	0	0	0	k__Bacteria; p__Proteobacteria; c__Deltaproteobacteria; o__Syntrophobacterales; f__Syntrophaceae; g__Desulfomonile
1056	0	9	26	0	0	0	0	0	0	0	0	0	k__Bacteria; p__Proteobacteria; c__Deltaproteobacteria; o__Desulfobacterales; f__Desulfobulbaceae; g__uncultured

1467	0	0	0	0	0	0	1	0	0	114	0	0	k__Bacteria; p__Firmicutes; c__Clostridia; o__Clostridiales; f__Lachnospiraceae
1426	0	0	0	0	0	0	0	0	0	19	0	0	k__Bacteria; p__Bacteroidetes; c__Bacteroidia; o__Bacteroidales; f__Bacteroidales_S24_7_group; g__uncultured_bacterium; s__uncultured_bacterium
1016	0	6	0	0	0	0	0	0	0	0	0	0	k__Bacteria; p__Proteobacteria; c__Zetaproteobacteria; o__Mariprofundales; f__Mariprofundaceae; g__Mariprofundus; Ambiguous_taxa
1098	0	0	5	0	0	0	4	0	3	0	0	0	k__Bacteria; p__Proteobacteria; c__Deltaproteobacteria; o__Bdellovibrionales; f__Bacteriovoracaceae; g__Peredibacter
1057	0	4	7	0	0	0	0	0	0	0	0	0	k__Bacteria; p__Bacteroidetes; c__Bacteroidia; o__Bacteroidales; f__Porphyromonadaceae; g__uncultured; s__uncultured_Bacteroidetes_bacterium
1468	0	0	0	0	0	0	0	0	0	12	0	0	k__Bacteria; p__Proteobacteria; c__Betaproteobacteria; o__Burkholderiales; f__Alcaligenaceae; g__Sutterella; s__uncultured_organism
1427	0	0	0	0	0	0	0	0	0	7	0	0	k__Bacteria; p__Firmicutes; c__Clostridia; o__Clostridiales; f__Ruminococcaceae; g__Ruminococcus_1
1017	0	29	17	1	0	0	29	10	17	5	1	0	k__Bacteria; p__Proteobacteria; c__Gammaproteobacteria; o__Oceanospirillales; f__Oceanospirillaceae; g__Oceanospirillum; Ambiguous_taxa
1099	3	5	36	0	0	0	25	38	45	2	0	0	k__Bacteria; p__Proteobacteria; c__Gammaproteobacteria; o__Oceanospirillales; f__Oleiphilaceae; g__Oleiphilus; s__uncultured_bacterium
1058	4	13	14	0	0	0	0	0	0	0	0	0	k__Bacteria; p__Proteobacteria; c__Gammaproteobacteria; o__Thiotrichales; f__Piscirickettsiaceae; g__endosymbionts

1469	0	0	0	0	0	0	60	92	0	0	7	3	k__Bacteria; p__Firmicutes; c__Clostridia; o__Clostridiales; f__Lachnospiraceae; g__uncultured; s__uncultured_bacterium
1428	0	0	0	0	0	0	0	0	0	20	0	0	k__Bacteria; p__Firmicutes; c__Clostridia; o__Clostridiales; f__Ruminococcaceae
1018	0	15	12	0	0	0	0	0	0	3	1	1	k__Bacteria; p__CKC4; Ambiguous_taxa; Ambiguous_taxa; Ambiguous_taxa; Ambiguous_taxa; Ambiguous_taxa
1059	5	79	36	0	1	0	0	0	0	1	0	0	k__Bacteria; p__Bacteroidetes; c__Flavobacteriia; o__Flavobacteriales; f__Flavobacteriaceae; g__Lutibacter; Ambiguous_taxa
1429	0	0	0	0	0	0	0	0	0	7	0	0	k__Bacteria; p__Bacteroidetes; c__Bacteroidia; o__Bacteroidales; f__Bacteroidales_S24_7_group; g__uncultured_organism; s__uncultured_organism
1019	0	17	2	0	0	0	0	0	0	0	0	0	k__Bacteria; p__Bacteroidetes; c__Sphingobacteriia; o__Sphingobacteriales; f__Saprospiraceae
680	2	4	7	0	0	0	0	13	7	0	0	0	k__Bacteria; p__Fibrobacteres; c__Fibrobacteria; o__Fibrobacteria_Incertae_Sedis; f__Unknown_Family; g__possible_genus_03; s__uncultured_bacterium
270	0	0	0	8	4	6	0	0	0	0	0	0	k__Bacteria; p__Proteobacteria; c__Gammaproteobacteria; o__Alteromonadales; f__Alteromonadaceae; g__Paraglaciecola
681	6	24	59	0	0	0	3	0	7	0	0	0	k__Bacteria; p__Proteobacteria; c__Alphaproteobacteria; o__Rhodobacterales; f__Rhodobacteraceae
271	0	0	0	19	0	0	0	0	0	0	0	0	k__Bacteria; p__Bacteroidetes; c__Bacteroidia; o__Bacteroidales; f__Prevotellaceae; g__Prevotellaceae_UCG_003
230	22	3	16	9	5	9	21	5	20	1	1	0	k__Bacteria; p__Proteobacteria; c__Alphaproteobacteria; o__Rhodobacterales; f__Rhodobacteraceae; g__Oceanicella

682	22	93	146	0	0	0	4	0	0	6	1	1	k__Bacteria; p__Firmicutes; c__Clostridia; o__Clostridiales; f__Family_XII; g__Fusibacter; s__uncultured_low_G+C_Gram_positive_bacterium
600	0	0	1	1	1	10	0	0	0	0	0	0	k__Bacteria; p__Bacteroidetes; c__Flavobacteriia; o__Flavobacteriales; f__Cryomorphaceae; g__Owenweeksia
272	50	23	54	43	17	42	49	56	43	3	0	0	k__Bacteria; p__Proteobacteria; c__Gammaproteobacteria; o__Vibrionales; f__Vibrionaceae; g__Vibrio; s__Vibrio_tapetis
231	73	5	40	0	131	136	100	86	122	12	129	3	k__Bacteria; p__Firmicutes; c__Bacilli; o__Lactobacillales; f__Streptococcaceae; g__Lactococcus; Ambiguous_taxa
683	7	83	85	0	0	0	421	350	296	2	0	0	k__Bacteria; p__Proteobacteria; c__Gammaproteobacteria
642	5	50	13	0	0	0	0	0	0	0	0	0	k__Bacteria; p__Proteobacteria; c__Gammaproteobacteria; o__Cellvibrionales; f__Cellvibrionaceae
601	0	0	0	0	0	12	0	0	0	0	0	0	k__Bacteria; p__Spirochaetae; c__Spirochaetes; o__Spirochaetales; f__Spirochaetaceae; g__Spirochaeta_2; s__uncultured_bacterium
273	1	0	32	0	48	0	1	0	0	13	0	0	k__Bacteria; p__Firmicutes; c__Clostridia; o__Clostridiales; f__Lachnospiraceae
684	41	190	65	0	0	0	10	4	11	0	0	0	k__Bacteria; p__Bacteroidetes; c__Flavobacteriia; o__Flavobacteriales; f__Flavobacteriaceae; g__Tenacibaculum; Ambiguous_taxa
643	0	0	0	0	0	0	0	0	0	3	8	0	k__Bacteria; p__Firmicutes; c__Clostridia; o__Clostridiales; f__Ruminococcaceae; g__Ruminiclostridium; s__uncultured_bacterium
602	19	134	132	1	0	16	0	0	0	0	0	0	k__Bacteria; p__Proteobacteria; c__Gammaproteobacteria; o__Oceanospirillales; f__Oceanospirillaceae; g__Marinobacterium; Ambiguous_taxa

274	8	30	50	12	12	13	0	0	0	0	0	0	k__Bacteria; p__Proteobacteria; c__Gammaproteobacteria; o__Order_Incertae_Sedis; f__Family_Incertae_Sedis; g__Marinicella; s__uncultured_bacterium
233	138	0	0	44	57	0	0	0	23	7	13	2	k__Bacteria; p__Bacteroidetes; c__Bacteroidia; o__Bacteroidales; f__Rikenellaceae; g__Alistipes
644	0	0	54	0	0	0	0	0	0	19	14	1	k__Bacteria; p__Actinobacteria; c__Coriobacteriia; o__Coriobacteriales; f__Coriobacteriaceae; g__Enterorhabdus; s__uncultured_bacterium
603	36	55	267	0	2	10	10	5	12	2	0	0	k__Bacteria; p__Proteobacteria; c__Gammaproteobacteria; o__Xanthomonadales; f__JTB255_marine_benthic_group; g__uncultured_sediment_bacterium; s__uncultured_sediment_bacterium
234	0	0	0	0	0	0	0	0	1	19	6	0	k__Bacteria; p__Firmicutes; c__Clostridia; o__Clostridiales; f__Family_XIII; g__Family_XIII_AD3011_group
686	7	35	27	0	0	0	2	0	4	0	0	0	k__Bacteria; p__Bacteroidetes; c__Cytophagia; o__Cytophagales; f__Flammeovirgaceae; g__Reichenbachiella; Ambiguous_taxa
645	160	0	0	0	0	0	0	0	0	0	0	0	k__Bacteria; p__Bacteroidetes; c__Bacteroidia; o__Bacteroidales; f__Prevotellaceae; g__Prevotellaceae_NK3B31_group; s__uncultured_bacterium
604	0	0	0	0	0	6	0	0	0	0	0	0	k__Bacteria; p__Proteobacteria; c__Gammaproteobacteria; o__Oceanospirillales; f__Oceanospirillaceae; g__Reinekea; Ambiguous_taxa
276	0	0	4	0	0	0	0	1	0	4	0	0	k__Bacteria; p__Actinobacteria; c__Actinobacteria; o__Micrococcales; f__Microbacteriaceae; g__Candidatus_Aquiluna; s__uncultured_bacterium

235	0	0	0	0	0	1	15	0	0	4	12	0	k__Bacteria; p__Firmicutes; c__Clostridia; o__Clostridiales; f__Lachnospiraceae
687	15	19	40	3	1	2	0	1	1	0	0	0	k__Bacteria; p__Proteobacteria; c__Alphaproteobacteria; o__Rhodobacterales; f__Rhodobacteraceae; g__Boseongicola; Ambiguous_taxa
646	0	0	0	0	0	0	0	0	0	0	7	6	k__Bacteria; p__Firmicutes; c__Clostridia; o__Clostridiales; f__Clostridiaceae_1; g__Clostridium_sensu_stricto_1
605	0	0	0	0	0	2	0	0	0	0	0	0	k__Bacteria; p__Cyanobacteria; c__Chloroplast; o__Pseudendoclonium_akinetum; f__Pseudendoclonium_akinetum; g__Pseudendoclonium_akinetum; s__Pseudendoclonium_akinetum
277	0	96	0	0	0	0	0	0	0	30	11	2	k__Bacteria; p__Firmicutes; c__Clostridia; o__Clostridiales; f__Lachnospiraceae
236	0	0	0	0	0	68	1	1	1	63	1	0	k__Bacteria; p__Bacteroidetes; c__Bacteroidia; o__Bacteroidales; f__Prevotellaceae; g__Prevotellaceae_UCG_003; s__uncultured_bacterium
688	1	19	27	0	0	0	16	0	0	1	0	0	k__Bacteria; p__Proteobacteria; c__Alphaproteobacteria; o__Rhodobacterales; f__Rhodobacteraceae; g__uncultured; s__uncultured_Marinovum_sp.
647	0	11	24	0	0	1	0	0	0	0	0	0	k__Bacteria; p__Proteobacteria; c__Alphaproteobacteria
606	0	0	3	0	3	2	0	0	2	0	0	0	k__Bacteria; p__Proteobacteria; c__Gammaproteobacteria; o__Thiotrichales; f__Thiotrichaceae; g__uncultured; s__uncultured_bacterium
237	733	183	91	84	81	168	1	159	35	111	66	18	k__Bacteria; p__Firmicutes; c__Clostridia; o__Clostridiales; f__Lachnospiraceae; g__Lachnospiraceae_UCG_001;

													s__uncultured_bacterium
689	26	97	146	0	0	0	49	39	52	5	2	0	k__Bacteria; p__Proteobacteria; c__Gammaproteobacteria; o__Vibrionales; f__Vibrionaceae; g__Vibrio
648	0	4	8	0	0	0	0	0	0	0	0	0	k__Bacteria; p__Bacteroidetes; c__Flavobacteriia; o__Flavobacteriales
607	9	16	22	12	1	11	1	2	5	1	0	0	k__Bacteria; p__Proteobacteria; c__Deltaproteobacteria; o__Desulfobacterales; f__Desulfobacteraceae; g__Sva0081_sediment_group
279	0	0	1	62	45	1	0	0	0	0	3	0	k__Bacteria; p__Firmicutes; c__Clostridia; o__Clostridiales; f__Lachnospiraceae
238	0	0	0	0	0	0	0	0	0	21	3	0	k__Bacteria; p__Bacteroidetes; c__Bacteroidia; o__Bacteroidales; f__Prevotellaceae; g__Prevotellaceae_NK3B31_group; s__uncultured_bacterium
649	39	176	109	0	1	0	0	0	0	0	0	0	k__Bacteria; p__Bacteroidetes; c__Flavobacteriia; o__Flavobacteriales; f__Flavobacteriaceae; g__Tenacibaculum
608	0	0	0	3	2	7	0	0	0	0	0	0	k__Bacteria; p__Proteobacteria; c__Gammaproteobacteria; o__Alteromonadales; f__Alteromonadaceae; g__Aliiglaciecola
239	367	0	0	79	104	167	44	73	44	63	79	57	k__Bacteria; p__Firmicutes; c__Clostridia; o__Clostridiales; f__Lachnospiraceae
609	2	16	22	0	1	5	0	0	0	0	0	0	k__Bacteria; p__Bacteroidetes; c__Sphingobacteriia; o__Sphingobacteriales; f__Saprospiraceae; g__uncultured; s__uncultured_bacterium
1390	0	0	0	0	0	0	1	0	1	0	0	0	k__Bacteria; p__Bacteroidetes
1760	0	0	0	0	0	0	0	5	0	0	0	0	k__Bacteria; p__Proteobacteria; c__Deltaproteobacteria; o__Desulfarculales; f__Desulfarculaceae; g__Desulfatiglans;

													s__uncultured_organism
1761	0	0	0	0	0	0	7	3	8	0	0	0	k__Bacteria; p__Proteobacteria; c__Gammaproteobacteria; o__Oceanospirillales; f__SS1_B_06_26; g__uncultured_bacterium; s__uncultured_bacterium
1720	0	0	0	0	0	0	0	0	1	0	0	0	k__Bacteria; p__Bacteroidetes; c__Sphingobacteriia; o__Sphingobacteriales; f__Saprospiraceae; g__uncultured; s__uncultured_Bacteroidetes_bacterium
1310	0	3	1	0	0	0	0	0	9	5	3	0	k__Bacteria; p__Proteobacteria; c__Gammaproteobacteria; o__Oceanospirillales; f__Oceanospirillaceae; g__Marinobacterium; Ambiguous_taxa
1762	0	0	0	0	0	0	1	3	0	0	0	0	k__Bacteria; p__Proteobacteria; c__Gammaproteobacteria; o__Cellvibrionales; f__Halieaceae
1393	6	25	63	0	0	0	5	1	14	0	0	0	k__Bacteria; p__Proteobacteria; c__Alphaproteobacteria; o__Rhodobacterales; f__Rhodobacteraceae; g__Ruegeria
1352	0	0	0	0	0	0	0	0	0	1	0	0	k__Bacteria; p__Proteobacteria; c__Deltaproteobacteria; o__Desulfobacterales; f__Desulfobacteraceae
1311	0	0	0	0	0	0	2	0	0	5	3	0	k__Bacteria; p__Proteobacteria; c__Deltaproteobacteria; o__Desulfobacterales; f__Desulfobulbaceae; g__Desulfopila; s__uncultured_bacterium
1763	0	0	0	0	0	0	0	2	1	0	0	0	k__Bacteria; p__Spirochaetae; c__Spirochaetes; o__Spirochaetales; f__PL_11B10; Ambiguous_taxa; Ambiguous_taxa
1394	0	0	0	0	0	0	8	2	2	8	1	0	k__Bacteria; p__Proteobacteria; c__Gammaproteobacteria; o__Oceanospirillales; f__Oceanospirillaceae; g__Neptuniibacter; Ambiguous_taxa

1312	0	0	0	0	0	0	0	0	0	7	0	0	k__Bacteria; p__Proteobacteria; c__Gammaproteobacteria; o__Pasteurellales; f__Pasteurellaceae; g__Actinobacillus; s__Actinobacillus_porcinus
1764	0	0	0	0	2	1	0	44	0	0	2	0	k__Bacteria; p__Bacteroidetes; c__Bacteroidia; o__Bacteroidales; f__Bacteroidales_S24_7_group
1723	0	0	0	6	0	0	2	2	4	0	0	0	k__Bacteria; p__Proteobacteria; c__Alphaproteobacteria; o__Rhodospirillales; f__Rhodospirillaceae; g__uncultured
1395	0	0	0	0	0	0	0	0	0	7	0	0	k__Bacteria; p__Firmicutes; c__Clostridia; o__Clostridiales; f__Lachnospiraceae
1354	0	0	0	0	0	0	0	0	0	0	1	0	k__Bacteria; p__Bacteroidetes; c__Flavobacteriia; o__Flavobacteriales; f__Cryomorphaceae; g__Fluviicola; s__uncultured_Bacteroidetes_bacterium
1765	0	0	1	0	0	0	2	6	8	0	0	0	k__Bacteria; p__Proteobacteria; c__Alphaproteobacteria; o__Rhodobacterales; f__Rhodobacteraceae
1724	0	3	4	0	0	0	0	0	4	0	0	0	k__Bacteria; p__Bacteroidetes; c__Bacteroidetes_BD2_2; o__uncultured_Cytophagales_bacterium; f__uncultured_Cytophagales_bacterium; g__uncultured_Cytophagales_bacterium; s__uncultured_Cytophagales_bacterium
1396	0	1	0	0	0	0	0	0	0	42	0	0	k__Bacteria; p__Firmicutes; c__Clostridia; o__Clostridiales; f__Lachnospiraceae; g__Lachnospiraceae_NK4A136_group
1766	0	0	0	0	0	0	0	7	0	0	0	0	k__Bacteria; p__Bacteroidetes; c__Cytophagia; o__Cytophagales
1725	0	0	0	0	0	0	0	0	3	0	0	0	k__Bacteria; p__Proteobacteria; c__Alphaproteobacteria; o__Rhodospirillales; f__Rhodospirillaceae; g__Thalassospira

1397	0	0	0	0	0	0	0	0	0	37	0	0	k__Bacteria; p__Bacteroidetes; c__Bacteroidia; o__Bacteroidales; f__Prevotellaceae; g__Prevotellaceae_UCG_001; s__uncultured_bacterium
1356	0	0	0	0	0	0	0	50	0	3	4	0	k__Bacteria; p__Bacteroidetes; c__Bacteroidia; o__Bacteroidales; f__Rikenellaceae; g__Alistipes
1315	0	0	0	0	2	0	6	0	0	0	0	0	k__Bacteria; p__Proteobacteria; c__Gammaproteobacteria; o__Chromatiales; f__Ectothiorhodospiraceae; g__Thiogranum
1767	0	0	1	0	0	0	0	3	0	0	0	0	k__Bacteria; p__Proteobacteria; c__Alphaproteobacteria; o__Rhodobacterales; f__Rhodobacteraceae
1726	0	0	0	0	0	0	0	0	2	0	0	0	k__Bacteria; p__Proteobacteria; c__Gammaproteobacteria; o__Legionellales; f__Coxiellaceae; g__Aquicella
1398	0	0	0	0	0	0	1	83	0	188	12	6	k__Bacteria; p__Firmicutes; c__Clostridia; o__Clostridiales; f__Ruminococcaceae; g__[Eubacterium]_coprostanoligenes_group
1357	0	0	0	0	0	0	0	0	0	17	0	0	k__Bacteria; p__Bacteroidetes; c__Bacteroidia; o__Bacteroidales; f__Bacteroidaceae; g__Bacteroides; s__uncultured_bacterium
1316	0	0	0	0	0	0	0	0	0	3	0	0	k__Bacteria; p__Firmicutes; c__Clostridia; o__Clostridiales; f__Family_XII; g__Fusibacter; s__uncultured_bacterium
1768	0	0	0	0	0	0	0	5	0	0	0	0	k__Bacteria; p__TM6
1727	4	37	35	1	1	0	16	29	6	6	0	2	k__Bacteria; p__Proteobacteria; c__Deltaproteobacteria; o__Desulfuromonadales; f__Sva1033
1399	0	0	0	0	0	0	0	0	0	10	0	0	k__Bacteria; p__Firmicutes; c__Clostridia; o__Clostridiales; f__Ruminococcaceae; g__Ruminococcaceae_UCG_004
1358	0	1	0	0	0	0	0	0	0	2	1	0	k__Bacteria; p__Tenericutes; c__Mollicutes; o__NB1_n

1317	0	0	0	0	0	0	0	0	0	1	0	0	k__Bacteria; p__Proteobacteria; c__Gammaproteobacteria; o__Oceanospirillales; f__Hahellaceae; g__Endozoicomonas; s__uncultured_bacterium
1769	0	0	0	0	0	0	4	6	14	0	0	0	k__Bacteria; p__Proteobacteria; c__Gammaproteobacteria; o__SS1_B_09_64
1728	0	0	0	0	0	0	5	0	0	0	0	0	k__Bacteria; p__Proteobacteria
1318	0	0	3	0	0	0	29	38	15	9	3	2	k__Bacteria; p__Bacteroidetes; c__Bacteroidia; o__Bacteroidia_Incertae_Sedis; f__Draconibacteriaceae; g__Draconibacterium
1319	0	0	23	0	0	25	0	151	38	5	25	10	k__Bacteria; p__Firmicutes; c__Clostridia; o__Clostridiales; f__Lachnospiraceae; g__Lachnospiraceae_NK4A136_group; s__uncultured_bacterium
90	0	0	34	83	36	2	1	2	46	124	9	0	k__Bacteria; p__Bacteroidetes; c__Bacteroidia; o__Bacteroidales; f__Prevotellaceae; g__Prevotella_2; s__uncultured_bacterium
91	0	0	0	51	36	0	41	0	0	6	6	0	k__Bacteria; p__Actinobacteria; c__Coriobacteriia; o__Coriobacteriales; f__Coriobacteriaceae; g__Enterorhabdus; s__uncultured_bacterium
50	438	156	1	159	64	161	0	1	49	44	212	69	k__Bacteria; p__Bacteroidetes; c__Bacteroidia; o__Bacteroidales; f__Bacteroidales_S24_7_group
980	0	6	2	0	2	0	0	0	0	0	0	0	k__Bacteria; p__Bacteroidetes
92	0	110	0	2	161	237	0	75	0	63	120	136	k__Bacteria; p__Firmicutes; c__Clostridia; o__Clostridiales; f__Lachnospiraceae
570	1	0	0	0	0	35	0	56	0	13	1	0	k__Bacteria; p__Firmicutes; c__Erysipelotrichia; o__Erysipelotrichales; f__Erysipelotrichaceae; g__Allobaculum; s__uncultured_bacterium

51	1121	361	96	368	617	448	479	436	154	261	355	112	k__Bacteria; p__Firmicutes; c__Bacilli; o__Bacillales; f__Staphylococcaceae; g__Staphylococcus; Ambiguous_taxa
160	62	0	162	172	102	134	69	6	64	99	53	8	k__Bacteria; p__Bacteroidetes; c__Bacteroidia; o__Bacteroidales; f__Prevotellaceae; g__Prevotella_9; s__uncultured_bacterium
10	567	0	29	155	277	378	229	192	264	154	72	8	k__Bacteria; p__Bacteroidetes; c__Bacteroidia; o__Bacteroidales; f__Prevotellaceae; g__uncultured; s__uncultured_bacterium
981	0	17	0	0	0	0	0	0	0	0	0	0	k__Bacteria; p__Proteobacteria; c__Gammaproteobacteria; o__Cellvibrionales; f__Cellvibrionaceae; g__Simiduia
940	0	5	12	4	0	0	2	0	7	0	0	0	k__Bacteria; p__Proteobacteria; c__Gammaproteobacteria
93	0	0	73	86	46	172	25	75	46	42	41	75	k__Bacteria; p__Proteobacteria; c__Gammaproteobacteria; o__Enterobacteriales; f__Enterobacteriaceae; g__Escherichia_Shigella
571	0	6	0	0	0	10	0	0	0	0	0	0	k__Bacteria; p__Deferribacteres; c__Deferribacteres_Incertae_Sedis; o__Unknown_Order; f__Unknown_Family; g__Caldithrix; s__uncultured_organism
530	298	162	0	0	30	0	0	0	3	54	5	1	k__Bacteria; p__Firmicutes; c__Clostridia; o__Clostridiales; f__Ruminococcaceae; g__Ruminococcaceae_UCG_002; Ambiguous_taxa
52	705	587	75	337	334	254	206	334	357	207	642	243	k__Bacteria; p__Bacteroidetes; c__Bacteroidia; o__Bacteroidales; f__Bacteroidaceae; g__Bacteroides
161	0	0	0	50	31	0	3	0	0	0	13	0	k__Bacteria; p__Bacteroidetes; c__Bacteroidia; o__Bacteroidales; f__Prevotellaceae
11	290	1	1	128	130	89	1	235	62	59	74	19	k__Bacteria; p__Firmicutes; c__Clostridia; o__Clostridiales; f__Ruminococcaceae; g__Ruminococcus_1

982	1	12	7	0	0	0	0	3	0	0	0	0	k__Bacteria; p__Bacteroidetes; c__Bacteroidia; o__Bacteroidales; f__Marinilabiaceae; g__Saccharicrinis; Ambiguous_taxa
94	0	0	0	0	1	111	0	31	0	11	14	5	k__Bacteria; p__Firmicutes; c__Clostridia; o__Clostridiales; f__Ruminococcaceae; g__Ruminococcaceae_UCG_014; s__uncultured_bacterium
941	2	12	24	1	0	0	0	0	5	0	0	0	k__Bacteria; p__Proteobacteria; c__Gammaproteobacteria; o__Sva0071; f__uncultured_bacterium; g__uncultured_bacterium; s__uncultured_bacterium
900	0	144	0	0	0	0	0	33	0	0	8	0	k__Bacteria; p__Firmicutes; c__Clostridia; o__Clostridiales; f__Ruminococcaceae; g__Ruminococcaceae_UCG_014; s__uncultured_bacterium
572	0	0	0	0	3	47	33	41	0	5	1	0	k__Bacteria; p__Firmicutes; c__Clostridia; o__Clostridiales; f__Clostridiales_vadinBB60_group; g__uncultured_bacterium; s__uncultured_bacterium
53	0	0	12	1	0	0	0	1	0	5	2	3	k__Bacteria; p__Firmicutes; c__Clostridia; o__Clostridiales; f__Lachnospiraceae
531	0	0	0	0	23	0	0	1	0	8	0	2	k__Bacteria; p__Firmicutes; c__Clostridia; o__Clostridiales; f__Lachnospiraceae; g__[Ruminococcus]_gauvreauii_group; s__uncultured_bacterium
162	0	0	0	6	0	0	0	37	0	4	5	0	k__Bacteria; p__Firmicutes; c__Clostridia; o__Clostridiales; f__Clostridiales_vadinBB60_group; g__uncultured_bacterium; s__uncultured_bacterium
12	592	354	150	421	342	322	291	155	523	195	503	385	k__Bacteria; p__Proteobacteria; c__Epsilonproteobacteria; o__Campylobacterales; f__Helicobacteraceae; g__Helicobacter; Ambiguous_taxa

121	0	0	0	0	1	54	2	0	0	3	1	0	k__Bacteria; p__Spirochaetae; c__Spirochaetes; o__Spirochaetales; f__Spirochaetaceae; g__Treponema_2; s__Treponema_berlinense
983	0	3	0	0	0	0	2	0	0	0	0	0	k__Bacteria; p__Proteobacteria; c__Zetaproteobacteria; o__Mariprofundales; f__Mariprofundaceae; g__Mariprofundus
95	223	86	20	0	103	240	93	77	54	86	33	6	k__Bacteria; p__Deferribacteres; c__Deferribacteres; o__Deferribacterales; f__Deferribacteraceae; g__Mucispirillum
942	0	18	2	0	0	0	0	0	0	0	1	0	k__Bacteria; p__Firmicutes; c__Clostridia; o__Clostridiales; f__Family_XII; g__Fusibacter; s__uncultured_bacterium
901	0	9	1	0	0	2	0	0	0	0	0	0	k__Bacteria; p__Bacteroidetes; c__Flavobacteriia; o__Flavobacteriales; f__Flavobacteriaceae; g__Maribacter; Ambiguous_taxa
573	0	0	0	0	0	62	0	0	0	0	4	0	k__Bacteria; p__Firmicutes; c__Clostridia; o__Clostridiales; f__Ruminococcaceae; g__Ruminococcaceae_UCG_014
532	142	0	0	0	24	0	0	0	36	0	6	0	k__Bacteria; p__Firmicutes; c__Clostridia; o__Clostridiales; f__Peptococcaceae; g__uncultured; s__uncultured_bacterium
163	0	81	0	1	2	32	0	0	40	25	5	1	k__Bacteria; p__Firmicutes; c__Clostridia; o__Clostridiales; f__Lachnospiraceae
13	0	250	102	138	128	314	79	142	127	1734	81	29	k__Bacteria; p__Firmicutes; c__Clostridia; o__Clostridiales; f__Ruminococcaceae; g__Faecalibacterium
122	244	269	71	288	410	475	192	286	254	143	339	136	k__Bacteria; p__Bacteroidetes; c__Bacteroidia; o__Bacteroidales; f__Bacteroidales_S24_7_group; g__uncultured_Bacteroidales_bacterium; s__uncultured_Bacteroidales_bacterium
984	0	1	7	2	0	3	0	0	0	0	0	0	k__Bacteria; p__Bacteroidetes; c__SB_5

96	0	0	0	76	46	0	0	0	0	15	9	0	k__Bacteria; p__Firmicutes; c__Clostridia; o__Clostridiales; f__Lachnospiraceae; g__Lachnospiraceae_NK4A136_group
943	0	13	0	0	0	0	0	0	0	0	0	0	k__Bacteria; p__Bacteroidetes
902	30	84	243	13	3	5	11	10	5	2	0	0	k__Bacteria; p__Proteobacteria; c__Alphaproteobacteria; o__Rhodobacterales; f__Rhodobacteraceae
574	0	0	0	0	0	77	0	0	0	9	11	3	k__Bacteria; p__Firmicutes; c__Clostridia; o__Clostridiales; f__Lachnospiraceae; g__uncultured
55	149	586	1477	1361	614	705	824	1551	1626	128	29	30	k__Bacteria; p__Firmicutes; c__Bacilli; o__Lactobacillales; f__Streptococcaceae; g__Lactococcus
533	0	0	0	1	5	12	0	2	0	0	0	0	k__Bacteria; p__Proteobacteria; c__Gammaproteobacteria; o__Gammaproteobacteria_Incertae_Sedis; f__Unknown_Family; g__Sedimenticola
164	277	0	39	51	20	284	0	35	25	24	35	29	k__Bacteria; p__Bacteroidetes; c__Bacteroidia; o__Bacteroidales; f__Porphyromonadaceae; g__Parabacteroides; Ambiguous_taxa
14	215	1	136	112	108	350	104	251	0	222	190	61	k__Bacteria; p__Firmicutes; c__Clostridia; o__Clostridiales; f__Lachnospiraceae; g__Lachnospiraceae_NK4A136_group
123	59	0	0	0	0	0	0	0	0	0	0	0	k__Bacteria; p__Firmicutes; c__Clostridia; o__Clostridiales; f__Ruminococcaceae; g__[Eubacterium]_coprostanoligenes_group; s__uncultured_organism
985	0	7	13	0	0	0	1	0	0	1	0	0	k__Bacteria; p__Gemmatimonadetes; c__Gemmatimonadetes; o__BD2_11_terrestrial_group
97	650	229	159	171	374	176	260	186	303	170	464	141	k__Bacteria; p__Bacteroidetes; c__Bacteroidia; o__Bacteroidales; f__Bacteroidales_S24_7_group; g__uncultured_Bacteroidales_bacterium;

													s__uncultured_Bacteroidales_bacterium
944	0	14	0	0	0	0	0	0	0	0	0	0	k__Bacteria; p__Proteobacteria; c__Deltaproteobacteria; o__Sh765B_TzT_29
903	0	24	0	0	0	0	0	0	0	0	0	0	k__Bacteria; p__Cloacimonetes; c__LK_44f; o__uncultured_bacterium; f__uncultured_bacterium; g__uncultured_bacterium; s__uncultured_bacterium
575	0	0	106	0	9	58	0	64	0	15	20	65	k__Bacteria; p__Firmicutes; c__Clostridia; o__Clostridiales; f__Lachnospiraceae
56	872	0	0	154	192	358	127	65	192	75	71	15	k__Bacteria; p__Proteobacteria; c__Gammaproteobacteria; o__Aeromonadales; f__Succinivibrionaceae; g__Succinivibrio; s__uncultured_bacterium
534	12	6	9	3	11	0	0	0	0	0	0	0	k__Bacteria; p__Bacteroidetes; c__Flavobacteriia; o__Flavobacteriales; f__Flavobacteriaceae; g__Lutibacter
165	0	0	14	3	6	11	0	7	0	0	0	0	k__Bacteria; p__Chloroflexi; c__uncultured
15	222	1	98	72	126	1	218	241	0	3	35	8	k__Bacteria; p__Bacteroidetes; c__Bacteroidia; o__Bacteroidales; f__Prevotellaceae; g__Prevotella_7; s__uncultured_bacterium
124	0	0	45	62	57	0	0	0	0	40	7	10	k__Bacteria; p__Firmicutes; c__Clostridia; o__Clostridiales; f__Lachnospiraceae
98	204	0	83	151	41	0	60	96	1	82	24	11	k__Bacteria; p__Bacteroidetes; c__Bacteroidia; o__Bacteroidales; f__Prevotellaceae; g__uncultured; s__uncultured_bacterium
986	0	11	1	0	0	0	0	0	0	0	0	0	k__Bacteria; p__Proteobacteria; c__Alphaproteobacteria; o__DB1_14
945	0	37	0	0	0	0	0	1	0	0	0	0	k__Bacteria; p__Gemmatimonadetes; c__Gemmatimonadetes;

													o__Gemmatimonadales; f__Gemmatimonadaceae; g__uncultured
904	0	21	1	0	0	0	1	0	0	0	0	0	k__Bacteria; p__Proteobacteria; c__Gammaproteobacteria; o__Chromatiales; f__Chromatiaceae; g__Halochromatium
57	0	0	40	0	0	55	1	0	2	9	2	0	k__Bacteria; p__Bacteroidetes; c__Bacteroidia; o__Bacteroidales; f__Rikenellaceae; g__Rikenellaceae_RC9_gut_group; s__uncultured_bacterium
576	0	0	0	0	0	12	0	0	2	0	0	0	k__Bacteria; p__Spirochaetae; c__Spirochaetes; o__Spirochaetales; f__Spirochaetaceae; g__Spirochaeta_2; s__uncultured_Spirochaeta_sp.
535	0	0	0	0	1	8	0	0	0	0	0	0	k__Bacteria; p__Proteobacteria; c__Epsilonproteobacteria; o__Campylobacterales; f__Helicobacteraceae; g__Sulfurovum; s__uncultured_Bathymodiolus_platifrons_gill_symbiont
16	130	0	1	34	41	29	0	0	0	120	14	1	k__Bacteria; p__Firmicutes; c__Clostridia; o__Clostridiales; f__Ruminococcaceae; g__Ruminiclostridium_6
166	92	116	106	83	77	52	8	7	18	7	5	1	k__Bacteria; p__Proteobacteria; c__Gammaproteobacteria; o__Alteromonadales; f__Pseudoalteromonadaceae; g__Pseudoalteromonas
125	0	0	1	475	204	370	0	0	0	0	0	0	k__Bacteria; p__Proteobacteria; c__Gammaproteobacteria; o__Alteromonadales; f__Colwelliaceae; g__Colwellia
99	0	0	0	0	0	0	2	0	60	50	1	0	k__Bacteria; p__Firmicutes; c__Clostridia; o__Clostridiales; f__Lachnospiraceae; g__[Eubacterium]_ruminantium_group; s__uncultured_bacterium
987	0	18	8	0	1	0	0	0	0	0	0	0	k__Bacteria; p__Proteobacteria; c__Gammaproteobacteria; o__Xanthomonadales; f__JTB255_marine_benthic_group
946	0	13	0	0	0	0	0	0	0	0	0	0	k__Bacteria; p__Bacteroidetes; c__Flavobacteriia;

													o__Flavobacteriales; f__Flavobacteriaceae; g__Gilvibacter
905	0	37	21	0	0	0	0	0	0	0	0	0	k__Bacteria; p__Bacteroidetes; c__Flavobacteriia; o__Flavobacteriales; f__Flavobacteriaceae; g__Kordia; Ambiguous_taxa
58	27	63	140	151	48	95	82	269	127	29	2	6	k__Bacteria; p__Firmicutes; c__Bacilli; o__Lactobacillales; f__Streptococcaceae; g__Lactococcus; s__Lactococcus_lactis
577	0	0	0	0	0	4	0	0	0	0	0	0	k__Bacteria; p__Proteobacteria; c__Deltaproteobacteria; o__SAR324_clade(Marine_group_B); f__uncultured_deep_sea_bacterium; g__uncultured_deep_sea_bacterium; s__uncultured_deep_sea_bacterium
536	0	0	0	0	28	0	0	0	0	0	3	0	k__Bacteria; p__Firmicutes; c__Clostridia; o__Clostridiales; f__Christensenellaceae; g__uncultured; s__uncultured_bacterium
17	0	5	135	369	201	47	92	288	0	100	206	126	k__Bacteria; p__Firmicutes; c__Clostridia; o__Clostridiales; f__Ruminococcaceae; g__Ruminiclostridium_9
167	0	0	71	49	100	51	25	58	46	42	77	56	k__Bacteria; p__Firmicutes; c__Clostridia; o__Clostridiales; f__Lachnospiraceae; g__Lachnospiraceae_UCG_006; s__uncultured_bacterium
126	30	24	47	7	0	8	9	3	2	0	0	0	k__Bacteria; p__Proteobacteria; c__Alphaproteobacteria; o__Rhodobacterales; f__Rhodobacteraceae
988	0	19	1	0	0	0	0	0	0	0	0	0	k__Bacteria; p__Bacteroidetes; c__Sphingobacteriia; o__Sphingobacteriales; f__NS11_12_marine_group; g__uncultured_bacterium; s__uncultured_bacterium
947	0	2	4	0	0	0	9	18	22	0	0	0	k__Bacteria; p__Bacteroidetes; c__Flavobacteriia; o__Flavobacteriales; f__Flavobacteriaceae; g__uncultured

906	0	14	21	1	0	0	0	0	0	0	0	0	k__Bacteria; p__Proteobacteria; c__Gammaproteobacteria; o__Chromatiales; f__Ectothiorhodospiraceae
59	718	803	110	193	376	288	94	110	106	198	266	28	k__Bacteria; p__Bacteroidetes; c__Bacteroidia; o__Bacteroidales; f__Bacteroidales_S24_7_group
578	0	0	0	9	7	14	0	0	0	0	0	0	k__Bacteria; p__Proteobacteria; c__Gammaproteobacteria; o__Alteromonadales; f__Colwelliaceae; g__Colwellia
537	0	110	34	0	16	77	0	23	4	18	118	39	k__Bacteria; p__Firmicutes; c__Clostridia; o__Clostridiales; f__Lachnospiraceae; g__Incertae_Sedis; s__uncultured_bacterium
18	220	0	24	64	71	84	0	0	0	106	28	6	k__Bacteria; p__Firmicutes; c__Clostridia; o__Clostridiales; f__Lachnospiraceae
168	191	4	44	1	76	172	58	55	124	44	124	65	k__Bacteria; p__Bacteroidetes; c__Bacteroidia; o__Bacteroidales; f__Rikenellaceae; g__Rikenella; Ambiguous_taxa
127	0	0	0	0	0	0	63	2	0	20	7	14	k__Bacteria; p__Firmicutes; c__Clostridia; o__Clostridiales; f__Lachnospiraceae
989	0	15	0	0	0	0	0	0	0	0	0	0	k__Bacteria; p__Proteobacteria; c__Deltaproteobacteria; o__Myxococcales; f__BIrii41; g__uncultured_bacterium; s__uncultured_bacterium
948	0	47	0	0	0	0	0	0	0	0	0	0	k__Bacteria; p__Proteobacteria; c__Betaproteobacteria; o__Burkholderiales; f__Burkholderiaceae; g__Polynucleobacter; Ambiguous_taxa
907	0	4	0	0	0	0	0	0	0	6	2	2	k__Bacteria; p__Firmicutes; c__Clostridia; o__Clostridiales
579	48	31	70	1	3	14	4	0	0	0	0	0	k__Bacteria; p__Actinobacteria; c__Acidimicrobiia; o__Acidimicrobiales; f__OM1_clade
538	6	25	12	4	9	0	4	0	0	0	0	0	k__Bacteria; p__Bacteroidetes; c__Bacteroidia;

													o__Bacteroidales; f__Marinilabiaceae; g__uncultured
19	668	1	61	99	205	59	170	105	107	81	130	23	k__Bacteria; p__Bacteroidetes; c__Bacteroidia; o__Bacteroidales; f__Bacteroidales_S24_7_group; g__uncultured_bacterium; s__uncultured_bacterium
169	0	0	0	1	75	0	44	14	0	20	65	33	k__Bacteria; p__Bacteroidetes; c__Bacteroidia; o__Bacteroidales; f__Bacteroidales_S24_7_group; g__uncultured_bacterium; s__uncultured_bacterium
128	0	111	0	0	62	91	0	1	25	130	61	4	k__Bacteria; p__Firmicutes; c__Clostridia; o__Clostridiales; f__Lachnospiraceae; g__Lachnospiraceae_NK4A136_group
949	0	1	1	4	0	0	0	0	3	0	0	0	k__Bacteria; p__Proteobacteria; c__Gammaproteobacteria; o__Pseudomonadales; f__Moraxellaceae; g__Moraxella; s__Solanum_melongena_(eggplant)
908	1	36	44	6	1	0	0	0	0	0	0	0	k__Bacteria; p__Gracilibacteria; c__bioreactor_metagenome; o__bioreactor_metagenome; f__bioreactor_metagenome; g__bioreactor_metagenome; s__bioreactor_metagenome
539	58	1	0	0	8	0	42	37	19	7	3	0	k__Bacteria; p__Firmicutes; c__Erysipelotrichia; o__Erysipelotrichales; f__Erysipelotrichaceae; g__Erysipelatoclostridium
129	15	144	105	24	0	1	41	35	32	151	92	56	k__Bacteria; p__Proteobacteria; c__Epsilonproteobacteria; o__Campylobacterales; f__Campylobacteraceae; g__Arcobacter; Ambiguous_taxa
909	0	107	0	0	0	0	0	0	0	0	0	0	k__Bacteria; p__Bacteroidetes; c__Cytophagia; o__Cytophagales; f__Cytophagaceae; g__uncultured
1690	0	0	0	0	0	0	2	1	7	0	0	0	k__Bacteria; p__Cyanobacteria; c__Chloroplast; o__uncultured_marine_eukaryote; f__uncultured_marine_eukaryote;

													g__uncultured_marine_eukaryote; s__uncultured_marine_eukaryote
1280	0	0	0	0	0	0	0	0	0	1	1	0	k__Bacteria; p__Gracilibacteria
1691	0	0	0	0	0	0	0	0	7	0	0	0	k__Bacteria; p__Spirochaetae; c__Spirochaetes; o__Spirochaetales; f__Spirochaetaceae; g__Spirochaeta_2
1650	0	0	0	0	0	0	1	0	1	0	0	0	k__Bacteria; p__Proteobacteria; c__Gammaproteobacteria; o__Xanthomonadales; f__uncultured; g__uncultured_gamma_proteobacterium; s__uncultured_gamma_proteobacterium
1281	0	0	0	0	0	1	0	0	0	0	0	0	k__Bacteria; p__Proteobacteria; c__Alphaproteobacteria; o__Rhodospirillales; f__Rhodospirillaceae; g__Magnetospira; s__uncultured_alpha_proteobacterium
1240	0	0	0	0	0	0	0	0	0	0	6	3	k__Bacteria; p__Firmicutes; c__Clostridia; o__Clostridiales
1651	0	0	0	0	0	0	1	0	8	0	0	0	k__Bacteria; p__Proteobacteria; c__pItb_vmat_80
1610	0	0	0	0	0	0	9	0	0	0	0	0	k__Bacteria; p__Bacteroidetes
1282	5	0	2	0	0	0	0	0	0	6	0	1	k__Bacteria; p__Tenericutes; c__Mollicutes; o__NB1_n; Ambiguous_taxa; Ambiguous_taxa; Ambiguous_taxa
1241	0	0	0	0	0	0	0	0	0	5	11	2	k__Bacteria; p__Firmicutes; c__Clostridia; o__Clostridiales; f__Ruminococcaceae; g__Anaerotruncus; s__uncultured_bacterium
1200	0	0	4	0	0	0	0	0	0	0	0	0	k__Bacteria; p__Proteobacteria; c__Deltaproteobacteria; o__Myxococcales
1693	0	0	0	0	0	0	0	1	0	0	0	0	k__Bacteria; p__Proteobacteria; c__Deltaproteobacteria; o__Desulfobacterales; f__Desulfobacteraceae
1652	0	0	0	0	0	0	1	0	0	0	0	0	k__Bacteria; p__Bacteroidetes; c__Sphingobacteriia; o__Sphingobacteriales; f__Saprospiraceae

1611	0	0	0	0	0	0	15	11	2	0	0	0	k__Bacteria; p__Proteobacteria; c__Deltaproteobacteria; o__Bdellovibrionales; f__Bacteriovoracaceae
1242	0	0	0	0	0	0	1	0	0	0	0	1	k__Bacteria; p__Bacteroidetes; c__Bacteroidia; o__Bacteroidales; f__Prevotellaceae; g__uncultured; s__uncultured_bacterium
1201	0	0	4	0	0	0	6	2	0	4	0	0	k__Bacteria; p__Proteobacteria; c__Deltaproteobacteria; o__Desulfovibrionales; f__Desulfovibrionaceae; g__Desulfovibrio
1694	0	0	0	0	0	0	0	1	0	0	0	0	k__Bacteria; p__Latescibacteria
1653	0	0	0	0	0	0	1	0	0	0	0	0	k__Bacteria; p__Proteobacteria; c__Alphaproteobacteria; o__Rhodospirillales; f__MSB_1E8; g__uncultured_bacterium; s__uncultured_bacterium
1612	0	0	0	0	0	0	2	4	0	0	0	0	k__Bacteria; p__Bacteroidetes
1284	0	0	0	0	0	0	0	0	0	4	0	1	k__Bacteria; p__Fibrobacteres; c__Fibrobacteria; o__possible_order_07; f__uncultured_bacterium; g__uncultured_bacterium; s__uncultured_bacterium
1243	0	0	0	0	0	0	0	0	0	2	5	7	k__Bacteria; p__Firmicutes; c__Clostridia; o__Clostridiales; f__Clostridiales_vadinBB60_group; g__uncultured_bacterium; s__uncultured_bacterium
1202	10	30	17	0	2	0	8	6	2	5	5	2	k__Bacteria; p__Proteobacteria; c__Epsilonproteobacteria; o__Campylobacterales; f__Campylobacteraceae; g__Arcobacter
1695	0	0	0	0	0	0	0	4	2	0	0	0	k__Bacteria; p__Bacteroidetes; c__Cytophagia; o__Cytophagales; f__Flammeovirgaceae; g__uncultured; s__uncultured_bacterium
1654	0	0	0	0	0	0	0	0	2	0	0	0	k__Bacteria; p__Proteobacteria; c__Deltaproteobacteria; o__Desulfobacterales; f__Desulfobacteraceae

1613	0	0	0	1	0	0	3	5	22	0	0	0	k__Bacteria; p__Bacteroidetes
1244	0	0	0	0	0	1	0	0	0	14	0	1	k__Bacteria; p__Firmicutes; c__Clostridia; o__Clostridiales; f__Clostridiales_vadinBB60_group; g__uncultured_bacterium; s__uncultured_bacterium
1203	1	0	10	0	1	0	4	0	0	0	0	0	k__Bacteria; p__Proteobacteria; c__Gammaproteobacteria; o__Cellvibrionales; f__Halieaceae; g__OM60(NOR5)_clade
1696	0	0	0	0	0	0	0	2	1	0	0	0	k__Bacteria; p__Firmicutes; c__Clostridia; o__Clostridiales; f__JTB215; Ambiguous_taxa; Ambiguous_taxa
1655	0	1	1	0	0	0	1	11	2	0	0	0	k__Bacteria; p__Proteobacteria; c__Deltaproteobacteria; o__Desulfobacterales; f__Desulfobulbaceae; g__Desulfobulbus
1614	0	0	0	0	0	0	4	7	31	0	0	0	k__Bacteria; p__Proteobacteria; c__Gammaproteobacteria; o__Alteromonadales
1286	0	0	3	1	0	0	0	0	0	8	3	0	k__Bacteria; p__Bacteroidetes; c__Bacteroidia; o__Bacteroidales; f__Marinilabiaceae; g__Marinifilum
1245	1	0	0	0	0	0	0	0	0	0	0	2	k__Bacteria; p__Firmicutes; c__Bacilli; o__Bacillales; f__Staphylococcaceae; g__Jeotgalicoccus; s__uncultured_bacterium
1204	0	0	9	1	0	0	0	0	0	0	0	0	k__Bacteria; p__Proteobacteria; c__Alphaproteobacteria; o__Rickettsiales; f__SAR116_clade; Ambiguous_taxa; Ambiguous_taxa
1697	0	0	0	0	0	0	4	7	6	0	0	0	k__Bacteria; p__Proteobacteria; c__Deltaproteobacteria; o__Desulfobacterales; f__Desulfobacteraceae; g__Desulfofrigus; Ambiguous_taxa
1615	0	0	0	0	0	0	9	0	0	0	0	0	k__Bacteria; p__Proteobacteria; c__Gammaproteobacteria
1287	0	0	0	0	0	0	66	0	0	4	6	0	k__Bacteria; p__Firmicutes; c__Clostridia; o__Clostridiales; f__Ruminococcaceae; g__Ruminiclostridium_5;

													Ambiguous_taxa
1246	0	132	42	0	0	0	0	0	0	5	3	3	k__Bacteria; p__Firmicutes; c__Clostridia; o__Clostridiales; f__Ruminococcaceae
1205	0	0	2	0	0	0	0	0	0	0	0	0	k__Bacteria
1698	0	0	0	0	0	0	5	1	1	0	0	0	k__Bacteria; p__Proteobacteria; c__Deltaproteobacteria; o__Desulfobacterales; f__Desulfobacteraceae
1616	0	0	0	0	0	0	5	8	5	0	0	0	k__Bacteria; p__Proteobacteria; c__Gammaproteobacteria
1288	0	0	0	0	0	0	1	0	0	2	0	0	k__Bacteria
1247	0	0	0	0	0	0	0	0	0	13	9	2	k__Bacteria; p__Firmicutes; c__Clostridia; o__Clostridiales; f__Lachnospiraceae; g__[Eubacterium]_ventriosum_group
1206	0	1	8	0	0	0	9	0	0	0	0	0	k__Bacteria; p__Proteobacteria; c__Gammaproteobacteria; o__Cellvibrionales; f__Halieaceae; g__Halioglobus; s__uncultured_bacterium
1699	0	0	0	0	0	0	7	4	5	0	0	0	k__Bacteria; p__Proteobacteria; c__Gammaproteobacteria; o__Alteromonadales; f__Alteromonadaceae; g__Glaciecola; s__uncultured_bacterium
1658	0	0	0	0	0	0	0	0	2	0	0	0	k__Bacteria; p__Bacteroidetes
1617	0	0	0	0	0	0	5	15	27	0	0	0	k__Bacteria; p__Proteobacteria; c__Gammaproteobacteria; o__Alteromonadales; f__Alteromonadaceae
1289	0	0	0	0	0	0	0	0	0	27	0	0	k__Bacteria; p__Proteobacteria; c__Betaproteobacteria; o__Burkholderiales; f__Alcaligenaceae; g__Sutterella; s__uncultured_bacterium
1248	0	0	0	0	0	0	0	0	0	0	16	0	k__Bacteria; p__Firmicutes; c__Clostridia; o__Clostridiales; f__Lachnospiraceae
1207	0	5	4	0	0	0	0	0	0	0	0	0	k__Bacteria; p__Acidobacteria; c__Subgroup_22;

													o__uncultured_bacterium; f__uncultured_bacterium; g__uncultured_bacterium; s__uncultured_bacterium
1659	0	0	0	0	0	0	6	0	1	0	0	0	k__Bacteria; p__Proteobacteria; c__Epsilonproteobacteria; o__Campylobacterales
1618	0	0	7	0	0	0	3	0	0	0	0	0	k__Bacteria; p__Cyanobacteria; c__Chloroplast
1208	0	3	23	1	0	2	0	0	0	0	0	0	k__Bacteria; p__Proteobacteria; c__Deltaproteobacteria; o__Desulfobacterales; f__Desulfobacteraceae; g__Desulfobacula
1619	0	0	0	0	0	0	9	5	13	0	0	0	k__Bacteria; p__Bacteroidetes; c__Cytophagia; o__Cytophagales; f__Flammeovirgaceae; g__Reichenbachiella
1209	0	1	6	0	1	0	8	12	32	3	0	1	k__Bacteria; p__Bacteroidetes; c__Bacteroidia; o__Bacteroidales; f__Marinilabiaceae; g__uncultured
870	2	2	0	0	0	0	10	2	0	1	0	0	k__Bacteria; p__Actinobacteria; c__Actinobacteria; o__Micrococcales; f__Micrococcaceae; g__Arthrobacter; Ambiguous_taxa
460	0	0	3	15	0	0	0	0	0	0	0	0	k__Bacteria; p__Proteobacteria; c__Alphaproteobacteria; o__Caulobacterales; f__Caulobacteraceae; g__Brevundimonas
871	14	8	9	0	0	0	2	0	0	0	0	0	k__Bacteria; p__Proteobacteria; c__Gammaproteobacteria; o__Cellvibrionales; f__Halieaceae; g__Halioglobus; s__uncultured_bacterium
830	6	11	17	5	0	0	3	0	0	0	0	0	k__Bacteria; p__Proteobacteria; c__Gammaproteobacteria; o__Chromatiales; f__Ectothiorhodospiraceae; Ambiguous_taxa; Ambiguous_taxa
461	16	19	11	5	1	1	3	0	1	15	4	2	k__Bacteria; p__Bacteroidetes; c__Bacteroidia; o__Bacteroidales; f__Porphyromonadaceae; g__uncultured; s__uncultured_Bacteroidetes_bacterium

420	0	12	0	12	0	0	0	0	0	0	2	1	k__Bacteria; p__Bacteroidetes; c__Bacteroidia; o__Bacteroidales; f__Porphyromonadaceae; g__Parabacteroides; Ambiguous_taxa
872	20	0	0	0	0	0	0	0	0	0	0	0	k__Bacteria; p__Bacteroidetes; c__Sphingobacteriia; o__Sphingobacteriales; f__Saprospiraceae
831	28	0	0	0	0	0	0	0	0	0	0	0	k__Bacteria; p__Proteobacteria; c__Deltaproteobacteria; o__Desulfuromonadales; f__GR_WP33_58; g__uncultured_bacterium_GR_WP33_58; s__uncultured_bacterium_GR_WP33_58
462	2	2	8	9	0	1	3	0	2	0	0	1	k__Bacteria; p__Proteobacteria; c__Deltaproteobacteria; o__Desulfobacterales; f__Desulfobacteraceae; g__Desulfococcus
421	0	0	0	12	6	15	0	0	0	0	0	0	k__Bacteria; p__Proteobacteria; c__Gammaproteobacteria; o__Alteromonadales; f__Colwelliaceae; g__Thalassotalea; s__uncultured_bacterium
873	6	4	5	0	0	0	0	0	0	0	0	0	k__Bacteria; p__Bacteroidetes; c__Flavobacteriia; o__Flavobacteriales; f__Flavobacteriaceae
832	17	0	36	0	0	0	7	3	1	0	0	0	k__Bacteria; p__Proteobacteria; c__Gammaproteobacteria; o__Alteromonadales; f__Pseudoalteromonadaceae
463	0	0	0	1	49	0	0	76	0	0	5	1	k__Bacteria; p__Actinobacteria; c__Coriobacteriia; o__Coriobacteriales; f__Coriobacteriaceae; g__Enterorhabdus; s__uncultured_bacterium
422	0	0	0	3	0	0	0	0	0	0	0	0	k__Bacteria; p__Proteobacteria; c__Gammaproteobacteria; o__Xanthomonadales; f__uncultured; g__uncultured_gamma_proteobacterium; s__uncultured_gamma_proteobacterium

874	7	2	6	0	1	7	1	0	0	0	0	0	k__Bacteria; p__Firmicutes; c__Clostridia; o__Clostridiales; f__Ruminococcaceae
833	12	1	10	0	0	0	0	0	0	0	0	0	k__Bacteria; p__Proteobacteria; c__Deltaproteobacteria; o__Desulfobacterales; f__Desulfobulbaceae
464	202	472	546	15	17	14	125	97	121	14	10	1	k__Bacteria; p__Proteobacteria; c__Gammaproteobacteria; o__Alteromonadales; f__Alteromonadaceae; g__uncultured; s__uncultured_bacterium
423	0	0	0	5	0	2	0	0	0	0	0	0	k__Bacteria; p__Bacteroidetes; c__Flavobacteriia; o__Flavobacteriales; f__Flavobacteriaceae; g__Aquimarina; Ambiguous_taxa
875	2	6	8	0	0	0	0	0	5	0	0	0	k__Bacteria; p__Bacteroidetes; c__Bacteroidetes_VC2.1_Bac22
834	7	15	6	2	0	0	0	0	0	0	0	0	k__Bacteria; p__Bacteroidetes; c__Flavobacteriia; o__Flavobacteriales; f__Flavobacteriaceae; g__Maribacter; Ambiguous_taxa
465	1	1	9	6	0	8	0	0	0	2	0	0	k__Bacteria; p__Proteobacteria
424	0	0	0	4	0	0	0	0	0	0	0	0	k__Bacteria; p__Proteobacteria; c__Gammaproteobacteria; o__Cellvibrionales; f__Halieaceae
876	7	20	9	0	0	0	55	31	29	10	0	0	k__Bacteria; p__Bacteroidetes; c__Flavobacteriia; o__Flavobacteriales; f__Flavobacteriaceae; g__uncultured; s__Bacteroidetes_bacterium_T4_KAD_str1
835	1	0	8	0	1	0	0	0	0	0	0	0	k__Bacteria; p__Aminicenantes; c__uncultured_bacterium; o__uncultured_bacterium; f__uncultured_bacterium; g__uncultured_bacterium; s__uncultured_bacterium
466	0	1	2	2	0	0	0	4	4	0	0	0	k__Bacteria; p__Proteobacteria; c__Gammaproteobacteria; o__KI89A_clade

425	0	0	0	14	0	1	0	0	0	0	0	0	k__Bacteria; p__Proteobacteria; c__Gammaproteobacteria; o__Thiotrichales; f__Thiotrichaceae; g__uncultured
877	8	21	88	2	0	0	0	0	0	0	0	0	k__Bacteria; p__Proteobacteria; c__Gammaproteobacteria; o__Gammaproteobacteria_Incertae_Sedis; f__Unknown_Family; g__Sedimenticola
836	5	35	3	2	0	0	2	5	5	0	0	0	k__Bacteria; p__Bacteroidetes; c__Bacteroidetes_BD2_2; o__uncultured_prokaryote; f__uncultured_prokaryote; g__uncultured_prokaryote; s__uncultured_prokaryote
467	12	18	88	4	3	10	0	2	0	0	0	0	k__Bacteria; p__Proteobacteria; c__Betaproteobacteria; o__Rhodocyclales; f__Rhodocyclaceae; g__Azospira; s__uncultured_actinobacterium
426	0	0	0	56	0	0	0	0	0	0	0	0	k__Bacteria; p__Firmicutes; c__Clostridia; o__Clostridiales; f__Lachnospiraceae
878	6	9	0	0	1	0	0	0	0	0	0	0	k__Bacteria; p__Acidobacteria; c__Holophagae; o__Subgroup_23
837	25	40	62	2	1	12	0	0	0	0	0	0	k__Bacteria; p__Proteobacteria; c__Gammaproteobacteria; o__Alteromonadales; f__Alteromonadaceae; g__Glaciecola
468	0	0	0	6	3	0	0	0	0	0	0	0	k__Bacteria; p__Proteobacteria; c__Alphaproteobacteria; o__Rhodobacterales; f__Rhodobacteraceae; g__uncultured
427	0	0	4	6	1	0	0	0	0	0	0	0	k__Bacteria; p__Proteobacteria; c__Deltaproteobacteria; o__Desulfobacterales; f__Desulfobulbaceae; g__Desulfocapsa
879	2	7	12	0	0	0	0	0	0	0	0	0	k__Bacteria; p__Proteobacteria; c__Gammaproteobacteria; o__Pseudomonadales; f__Moraxellaceae; g__Acinetobacter; s__Acinetobacter_calcoaceticus
838	1	35	21	0	0	0	0	0	0	0	0	0	k__Bacteria; p__Bacteroidetes; c__Sphingobacteriia; o__Sphingobacteriales; f__Saprospiraceae

469	0	4	6	4	5	2	1	0	9	0	0	0	k__Bacteria; p__Firmicutes; c__Bacilli; o__Lactobacillales; f__Streptococcaceae; g__Streptococcus; s__Streptococcus_parauberis
428	0	26	11	13	4	6	1	5	6	0	0	0	k__Bacteria; p__Firmicutes; c__Bacilli; o__Bacillales; f__Paenibacillaceae; g__Paenibacillus; s__Paenibacillus_amylolyticus
839	6	0	0	0	0	0	0	0	0	0	0	0	k__Bacteria; p__Proteobacteria; c__Deltaproteobacteria; o__Sh765B_TzT_29; f__uncultured_delta_proteobacterium; g__uncultured_delta_proteobacterium; s__uncultured_delta_proteobacterium
429	103	232	452	9	8	29	13	7	9	1	0	0	k__Bacteria; p__Proteobacteria; c__Gammaproteobacteria; o__Xanthomonadales; f__JTB255_marine_benthic_group
1580	2	2	3	1	0	0	5	5	17	0	0	0	k__Bacteria; p__Proteobacteria; c__Deltaproteobacteria; o__Desulfobacterales; f__Desulfobulbaceae; g__uncultured; s__uncultured_Desulfobulbaceae_bacterium
1170	0	0	5	0	0	0	0	0	0	0	0	0	k__Bacteria; p__Bacteroidetes; c__Sphingobacteriia; o__Sphingobacteriales; f__Saprospiraceae; g__uncultured; s__uncultured_Sphingobacteriales_bacterium
1581	0	10	2	0	0	0	44	18	41	0	0	0	k__Bacteria; p__Proteobacteria; c__Gammaproteobacteria; o__Alteromonadales; f__Alteromonadaceae; g__Aestuariibacter; s__uncultured_bacterium
1540	0	0	0	0	0	0	24	0	15	0	0	0	k__Bacteria; p__Proteobacteria; c__Gammaproteobacteria
1171	0	0	7	0	0	0	0	0	0	0	0	0	k__Bacteria; p__Bacteroidetes; c__Bacteroidia; o__Bacteroidia_Incertae_Sedis; f__Draconibacteriaceae; g__Draconibacterium

1130	0	0	3	0	0	0	0	0	0	0	0	0	k__Bacteria; p__Proteobacteria; c__Gammaproteobacteria; o__Oceanospirillales; f__Alcanivoracaceae; g__Pleionea; Ambiguous_taxa
1582	0	0	0	0	0	0	9	3	2	0	0	0	k__Bacteria; p__Proteobacteria; c__Epsilonproteobacteria; o__Campylobacterales; f__Helicobacteraceae; g__Sulfurimonas
1541	0	0	0	0	0	0	9	0	3	0	0	0	k__Bacteria; p__Proteobacteria; c__Alphaproteobacteria; o__Rhodospirillales; f__Rhodospirillaceae
1500	0	0	0	0	0	0	14	2	6	0	0	0	k__Bacteria; p__Proteobacteria; c__Alphaproteobacteria; o__Rhodospirillales; f__Rhodospirillales_Incertae_Sedis; g__Candidatus_Alysiosphaera
1172	0	0	2	0	0	0	0	0	0	0	0	0	k__Bacteria; p__Firmicutes; c__Bacilli; o__Lactobacillales; f__Lactobacillaceae; g__Lactobacillus; s__Lactobacillus_paracasei
1131	0	6	11	0	0	0	0	4	0	0	0	1	k__Bacteria; p__Proteobacteria; c__Gammaproteobacteria; o__Aeromonadales; f__Aeromonadaceae; g__uncultured
1583	0	0	0	0	0	0	3	3	0	0	0	0	k__Bacteria; p__Bacteroidetes
1542	0	0	0	0	0	0	23	1	5	2	0	0	k__Bacteria; p__Tenericutes; c__Mollicutes; o__NB1_n; f__uncultured_organism; g__uncultured_organism; s__uncultured_organism
1501	0	0	6	0	0	0	8	12	20	0	0	0	k__Bacteria; p__Proteobacteria; c__Gammaproteobacteria
1173	0	0	6	0	0	0	0	0	0	0	0	0	k__Bacteria; p__Proteobacteria
1132	0	0	15	0	0	0	0	0	0	0	0	0	k__Bacteria; p__Firmicutes; c__Clostridia; o__Clostridiales; f__Lachnospiraceae
1584	0	0	0	0	0	0	6	0	0	0	0	0	k__Bacteria; p__Proteobacteria; c__Deltaproteobacteria
1543	0	0	0	0	0	0	17	16	5	0	0	0	k__Bacteria; p__Proteobacteria; c__Epsilonproteobacteria; o__Campylobacterales; f__Helicobacteraceae; g__Sulfurimonas;

													s__uncultured_prokaryote
1502	1	0	0	2	0	3	30	21	29	0	0	0	k__Bacteria; p__Proteobacteria; c__Deltaproteobacteria; o__Desulfobacterales; f__Desulfobacteraceae; g__Desulfosarcina; Ambiguous_taxa
1174	19	158	182	5	6	0	0	0	0	0	0	0	k__Bacteria; p__Proteobacteria; c__Gammaproteobacteria; o__Alteromonadales; f__Pseudoalteromonadaceae; g__Psychrosphaera
1133	0	1	7	0	0	0	0	0	0	0	0	0	k__Bacteria; p__Bacteroidetes; c__Bacteroidia; o__Bacteroidales; f__Marinilabiaceae; g__uncultured; s__uncultured_marine_bacterium
1585	0	0	0	0	0	0	15	11	0	0	0	0	k__Bacteria; p__Proteobacteria; c__Epsilonproteobacteria; o__Campylobacterales; f__Helicobacteraceae
1544	0	0	0	0	0	0	9	0	3	0	0	0	k__Bacteria; p__Bacteroidetes; c__Flavobacteriia; o__Flavobacteriales; f__Cryomorphaceae; g__Owenweeksia
1503	0	0	0	0	0	0	20	9	23	0	0	0	k__Bacteria; p__Bacteroidetes; c__Flavobacteriia; o__Flavobacteriales; f__Cryomorphaceae; g__Owenweeksia
1175	0	0	6	0	0	0	0	0	0	0	0	0	k__Bacteria; p__Proteobacteria; c__Gammaproteobacteria; o__Cellvibrionales
1134	0	0	12	0	0	0	0	0	0	0	0	0	k__Bacteria; p__Proteobacteria; c__Deltaproteobacteria; o__Desulfobacterales; f__Desulfobacteraceae
1586	0	0	0	1	0	0	7	0	1	1	1	0	k__Bacteria; p__Proteobacteria; c__Deltaproteobacteria; o__Desulfobacterales; f__Desulfobulbaceae
1545	0	2	2	0	0	0	10	1	6	1	0	0	k__Bacteria; p__Bacteroidetes; c__Cytophagia; o__Cytophagales; f__Flammeovirgaceae; g__Fabibacter; Ambiguous_taxa

1504	0	0	0	0	0	0	5	6	18	0	0	0	k__Bacteria; p__Proteobacteria; c__Gammaproteobacteria; o__Chromatiales; f__Ectothiorhodospiraceae
1176	0	0	11	0	0	0	0	0	0	0	0	0	k__Bacteria; p__Proteobacteria; c__Alphaproteobacteria; o__Rhodobacterales; f__Rhodobacteraceae
1135	0	0	3	0	0	0	4	0	0	0	0	0	k__Bacteria; p__Spirochaetae; c__Spirochaetes; o__Spirochaetales; f__Spirochaetaceae; g__Spirochaeta_2; s__Spirochaeta_isovalerica
1587	0	0	0	0	0	0	20	21	13	0	0	0	k__Bacteria; p__Bacteroidetes; c__Flavobacteriia; o__Flavobacteriales; f__Cryomorphaceae; g__Owenweeksia; Ambiguous_taxa
1546	0	0	0	0	0	0	3	0	6	0	0	0	k__Bacteria; p__Spirochaetae; c__Spirochaetes; o__Spirochaetales; f__Spirochaetaceae; g__Spirochaeta_2; s__uncultured_alpha_proteobacterium
1505	0	0	0	0	0	0	35	0	0	0	0	0	k__Bacteria; p__Actinobacteria; c__Acidimicrobiia; o__Acidimicrobiales; f__Acidimicrobiaceae; g__CL500_29_marine_group
1177	0	0	3	0	0	0	0	0	0	0	0	0	k__Bacteria; p__Proteobacteria; c__Gammaproteobacteria; o__Cellvibrionales; f__Halieaceae; g__OM60(NOR5)_clade
1136	0	0	3	0	0	0	0	0	0	0	0	0	k__Bacteria; p__Acidobacteria; c__Holophagae; o__Subgroup_10; f__NS72; g__uncultured_bacterium; s__uncultured_bacterium
1588	0	0	0	0	0	0	2	0	0	0	0	0	k__Bacteria; p__Bacteroidetes; c__Cytophagia; o__Cytophagales; f__Flammeovirgaceae; g__Reichenbachiella
1547	0	0	0	0	0	0	13	27	54	0	0	0	k__Bacteria; p__Proteobacteria; c__Gammaproteobacteria; o__JPC_34; Ambiguous_taxa; Ambiguous_taxa; Ambiguous_taxa

1506	0	0	0	1	9	2	55	1	29	9	18	1	k__Bacteria; p__Firmicutes; c__Clostridia; o__Clostridiales; f__Lachnospiraceae; g__Lachnospiraceae_NK4A136_group; s__unidentified
1178	2	0	28	0	0	0	0	0	0	0	0	0	k__Bacteria; p__Proteobacteria; c__Deltaproteobacteria; o__Bdellovibrionales; f__Bdellovibrionaceae; g__OM27_clade
1137	0	0	6	0	0	1	17	5	2	0	0	0	k__Bacteria; p__Proteobacteria; c__Gammaproteobacteria
1589	0	0	0	0	0	0	3	0	0	0	0	0	k__Bacteria; p__Bacteroidetes; c__Bacteroidetes_BD2_2
1548	0	0	0	0	0	0	3	1	8	0	0	1	k__Bacteria; p__Proteobacteria; c__Gammaproteobacteria; o__Order_Incertae_Sedis; f__Family_Incertae_Sedis; g__Marinicella
1507	0	0	0	0	0	0	75	35	33	0	0	0	k__Bacteria; p__Proteobacteria; c__Epsilonproteobacteria; o__Campylobacterales; f__Helicobacteraceae; g__Sulfurimonas
1179	0	4	3	0	0	0	0	0	0	0	0	0	k__Bacteria; p__Proteobacteria; c__Deltaproteobacteria; o__Desulfuromonadales; f__GR_WP33_58; g__uncultured_delta_proteobacterium; s__uncultured_delta_proteobacterium
1138	2	0	9	1	0	0	0	0	0	0	0	0	k__Bacteria; p__Acidobacteria; c__Subgroup_22
1549	1	45	51	0	0	0	7	1	3	2	0	0	k__Bacteria; p__Proteobacteria; c__Gammaproteobacteria; o__Vibrionales; f__Vibrionaceae; g__Vibrio; s__Vibrio_vulnificus
1508	0	0	0	0	0	1	2	0	1	0	0	0	k__Bacteria; p__Proteobacteria; c__Deltaproteobacteria; o__Desulfobacterales; f__Desulfobacteraceae
1139	0	0	8	0	0	0	0	0	0	0	0	0	k__Bacteria; p__Bacteroidetes; c__Sphingobacteriia; o__Sphingobacteriales; f__Saprospiraceae
1509	0	0	0	0	0	0	52	63	72	0	0	0	k__Bacteria; p__Proteobacteria; c__Gammaproteobacteria; o__Alteromonadales; f__Pseudoalteromonadaceae;

													g__uncultured; s__uncultured_gamma_proteobacterium
390	0	0	6	1	4	14	0	0	0	0	0	0	k__Bacteria; p__Proteobacteria; c__Deltaproteobacteria; o__Desulfobacterales; f__Desulfobacteraceae
760	1	18	32	10	1	5	0	0	0	0	0	0	k__Bacteria; p__Bacteroidetes; c__Flavobacteriia; o__Flavobacteriales; f__Cryomorphaceae; g__Crocinitomix; s__uncultured_Crocinitomix_sp.
391	0	1	2	13	3	15	0	0	0	0	0	0	k__Bacteria; p__Proteobacteria; c__Gammaproteobacteria; o__Alteromonadales; f__Alteromonadaceae; g__Agarivorans; Ambiguous_taxa
350	0	3	0	34	8	5	0	0	0	0	0	0	k__Bacteria; p__Proteobacteria; c__Gammaproteobacteria; o__Alteromonadales; f__Alteromonadaceae
761	16	17	19	0	0	0	2	7	7	0	0	0	k__Bacteria; p__Proteobacteria; c__Gammaproteobacteria
720	10	34	270	0	0	0	0	0	1	0	0	0	k__Bacteria; p__Proteobacteria; c__Gammaproteobacteria; o__Vibrionales; f__Vibrionaceae; g__Vibrio
392	0	0	29	55	0	69	0	0	0	0	12	2	k__Bacteria; p__Firmicutes; c__Clostridia; o__Clostridiales; f__Lachnospiraceae; g__uncultured; s__uncultured_bacterium
351	0	0	0	26	41	0	106	0	0	0	0	0	k__Bacteria; p__Bacteroidetes; c__Bacteroidia; o__Bacteroidales; f__Prevotellaceae; g__Prevotellaceae_NK3B31_group; s__uncultured_bacterium
310	0	3	29	18	10	7	11	54	5	1	1	0	k__Bacteria; p__Firmicutes; c__Bacilli; o__Bacillales; f__Bacillaceae; g__Geobacillus; Ambiguous_taxa
762	0	3	9	0	0	0	6	3	1	0	0	0	k__Bacteria; p__Bacteroidetes; c__Cytophagia; o__Cytophagales; f__Flammeovirgaceae; g__uncultured; s__uncultured_sediment_bacterium
721	18	82	113	2	1	5	0	0	0	0	0	0	k__Bacteria; p__Bacteroidetes; c__Flavobacteriia;

													o__Flavobacteriales; f__Flavobacteriaceae; g__Maribacter; s__uncultured_bacterium
393	0	0	0	26	0	0	1	20	1	0	1	0	k__Bacteria; p__Firmicutes; c__Clostridia; o__Clostridiales; f__Ruminococcaceae; g__Ruminococcaceae_UCG_002; s__uncultured_organism
352	0	0	0	41	30	0	0	0	0	10	34	35	k__Bacteria; p__Firmicutes; c__Clostridia; o__Clostridiales; f__Lachnospiraceae
311	0	0	39	24	22	18	39	24	17	26	1	0	k__Bacteria; p__Proteobacteria; c__Gammaproteobacteria; o__Enterobacteriales; f__Enterobacteriaceae; g__Enterobacter; s__unidentified_marine_bacterioplankton
763	81	292	290	2	9	0	0	0	0	0	0	0	k__Bacteria; p__Bacteroidetes; c__Flavobacteriia; o__Flavobacteriales; f__Flavobacteriaceae
722	12	62	110	0	0	0	1	0	1	0	1	0	k__Bacteria; p__Proteobacteria; c__Alphaproteobacteria; o__Rhodobacterales; f__Rhodobacteraceae
394	0	2	2	4	0	1	0	0	0	0	0	0	k__Bacteria; p__Proteobacteria; c__Alphaproteobacteria; o__Rhodobacterales; f__Rhodobacteraceae
353	149	0	59	121	54	0	1	92	103	74	19	5	k__Bacteria; p__Firmicutes; c__Clostridia; o__Clostridiales; f__Lachnospiraceae; g__uncultured
764	46	92	70	0	0	0	1	0	0	0	0	0	k__Bacteria; p__Bacteroidetes; c__Flavobacteriia; o__Flavobacteriales; f__Flavobacteriaceae; g__Tenacibaculum; Ambiguous_taxa
723	0	6	1	1	0	2	8	6	0	0	0	0	k__Bacteria; p__Proteobacteria; c__Deltaproteobacteria; o__Desulfobacterales; f__Desulfobacteraceae; g__Sva0081_sediment_group; s__uncultured_delta_proteobacterium

395	0	0	1	14	0	0	0	0	0	0	0	0	k__Bacteria; p__Bacteroidetes; c__Cytophagia; o__Cytophagales; f__Flammeovirgaceae; g__Flexithrix; s__uncultured_bacterium
354	0	0	0	5	0	5	0	0	0	0	0	0	k__Bacteria; p__Bacteroidetes; c__Flavobacteriia; o__Flavobacteriales; f__Flavobacteriaceae; g__Wenyingzhuangia
313	2	182	35	47	127	102	158	167	104	133	26	3	k__Bacteria; p__Deferribacteres; c__Deferribacteres; o__Deferribacterales; f__Deferribacteraceae; g__Mucispirillum; Ambiguous_taxa
765	31	19	31	0	0	0	0	0	0	1	0	0	k__Bacteria; p__Proteobacteria; c__Gammaproteobacteria; o__Vibrionales; f__Vibrionaceae; g__Vibrio
724	0	0	2	0	0	0	0	0	0	0	0	0	k__Bacteria; p__Proteobacteria; c__Deltaproteobacteria; o__Bdellovibrionales; f__Bdellovibrionaceae; g__OM27_clade
396	0	0	49	36	0	0	0	0	0	0	5	0	k__Bacteria; p__Firmicutes; c__Clostridia; o__Clostridiales; f__Lachnospiraceae; g__uncultured
355	0	70	0	121	33	0	0	0	52	9	17	18	k__Bacteria; p__Firmicutes; c__Erysipelotrichia; o__Erysipelotrichales; f__Erysipelotrichaceae
314	0	0	2	0	0	2	0	0	0	0	0	0	k__Bacteria; p__Bacteroidetes
766	235	0	0	0	0	0	0	0	0	0	0	4	k__Bacteria; p__Firmicutes; c__Clostridia; o__Clostridiales; f__Ruminococcaceae; g__Ruminococcaceae_UCG_010
725	15	16	15	0	0	2	0	0	0	0	0	0	k__Bacteria; p__Proteobacteria; c__Gammaproteobacteria; o__Thiotrichales; f__Piscirickettsiaceae; g__endosymbionts
397	111	0	0	37	56	65	0	74	66	32	31	13	k__Bacteria; p__Firmicutes; c__Clostridia; o__Clostridiales; f__Lachnospiraceae; g__Lachnospiraceae_NK4A136_group; s__uncultured_bacterium

356	0	0	48	17	0	0	0	0	0	0	0	0	k__Bacteria; p__Bacteroidetes; c__Bacteroidia; o__Bacteroidales; f__Prevotellaceae; g__Alloprevotella; s__uncultured_bacterium
315	0	2	35	27	18	45	20	47	58	8	1	0	k__Bacteria; p__Proteobacteria; c__Gammaproteobacteria; o__Enterobacteriales; f__Enterobacteriaceae; g__Cronobacter; s__Cronobacter_sakazakii
767	161	0	0	0	0	0	49	0	0	1	8	3	k__Bacteria; p__Firmicutes; c__Clostridia; o__Clostridiales; f__Lachnospiraceae
726	12	70	70	0	0	0	0	0	0	0	0	0	k__Bacteria; p__Bacteroidetes; c__Flavobacteriia; o__Flavobacteriales; f__Flavobacteriaceae; g__uncultured
398	0	0	29	56	0	73	81	0	54	22	55	0	k__Bacteria; p__Firmicutes; c__Clostridia; o__Clostridiales; f__Lachnospiraceae; g__uncultured
357	133	512	614	35	32	54	9	8	7	1	0	0	k__Bacteria; p__Bacteroidetes; c__Flavobacteriia; o__Flavobacteriales; f__Flavobacteriaceae; g__Lutimonas
316	375	1161	1056	15	4	8	0	0	0	0	0	0	k__Bacteria; p__Bacteroidetes; c__Flavobacteriia; o__Flavobacteriales; f__Flavobacteriaceae; g__Aquibacter
768	166	0	0	0	0	0	0	0	0	0	0	0	k__Bacteria; p__Firmicutes; c__Clostridia; o__Clostridiales; f__Lachnospiraceae
727	0	0	11	0	0	0	0	0	0	0	0	0	k__Bacteria; p__Bacteroidetes; c__Sphingobacteriia; o__Sphingobacteriales; f__Saprospiraceae; g__Lewinella; s__Lewinella_nigricans
399	1	67	38	1	42	0	0	0	0	12	13	3	k__Bacteria; p__Firmicutes; c__Clostridia; o__Clostridiales; f__Lachnospiraceae
358	0	0	0	36	41	0	0	1	56	14	6	10	k__Bacteria; p__Firmicutes; c__Clostridia; o__Clostridiales; f__Lachnospiraceae; g__Acetatifactor; s__uncultured_bacterium

317	0	0	0	1	2	1	0	0	0	0	2	0	k__Bacteria; p__Proteobacteria; c__Alphaproteobacteria; o__Rhodospirillales; f__Acetobacteraceae; g__Saccharibacter; Ambiguous_taxa
769	180	0	0	0	0	0	1	0	0	4	0	0	k__Bacteria; p__Firmicutes; c__Clostridia; o__Clostridiales; f__Ruminococcaceae; g__Ruminococcaceae_UCG_014
728	0	2	0	0	0	0	0	0	0	0	0	0	k__Bacteria; p__Proteobacteria; c__Gammaproteobacteria; o__Cellvibrionales; f__Spongiibacteraceae
359	0	0	0	38	76	163	0	137	0	10	15	28	k__Bacteria; p__Firmicutes; c__Clostridia; o__Clostridiales; f__Lachnospiraceae; g__uncultured
318	34	76	240	52	18	25	51	7	21	1	0	0	k__Bacteria; p__Proteobacteria; c__Deltaproteobacteria; o__Desulfobacterales; f__Desulfobacteraceae; g__Desulfosarcina; Ambiguous_taxa
729	1	44	66	0	0	0	7	18	1	1	0	0	k__Bacteria; p__Proteobacteria; c__Gammaproteobacteria; o__Order_Incertae_Sedis; f__Family_Incertae_Sedis; g__Marinicella
319	0	0	0	0	0	43	64	0	20	0	2	2	k__Bacteria; p__Bacteroidetes; c__Bacteroidia; o__Bacteroidales; f__Rikenellaceae; g__Rikenella; Ambiguous_taxa
1470	0	0	0	0	0	0	0	0	0	0	7	0	k__Bacteria; p__Firmicutes; c__Clostridia; o__Clostridiales; f__Ruminococcaceae; g__Ruminococcaceae_UCG_010
1060	3	13	21	0	0	0	36	25	19	0	0	0	k__Bacteria; p__Proteobacteria; c__Gammaproteobacteria; o__Oceanospirillales; f__Oceanospirillaceae
1471	0	0	1	0	0	0	0	0	0	0	4	0	k__Bacteria; p__Firmicutes; c__Clostridia; o__Clostridiales; f__Lachnospiraceae; g__Lachnospiraceae_UCG_006; s__uncultured_bacterium

1430	0	0	1	0	0	0	0	0	0	19	0	0	k__Bacteria; p__Firmicutes; c__Clostridia; o__Clostridiales; f__Ruminococcaceae; g__Ruminococcaceae_UCG_008; s__uncultured_bacterium
1061	6	6	16	1	0	3	5	0	2	0	0	0	k__Bacteria; p__Proteobacteria; c__Gammaproteobacteria; o__Pseudomonadales; f__Moraxellaceae; g__Acinetobacter; s__Acinetobacter_sp.
1020	0	5	26	0	0	0	3	0	0	0	0	0	k__Bacteria; p__Acidobacteria; c__Holophagae; o__Subgroup_23
1800	0	0	5	0	0	0	0	2	8	0	0	0	k__Bacteria; p__Cyanobacteria; c__Cyanobacteria; o__SubsectionI; f__FamilyI; g__Synechococcus
1472	0	0	1	1	0	0	0	0	0	0	5	0	k__Bacteria; p__Proteobacteria; c__Betaproteobacteria; o__Burkholderiales; f__Burkholderiaceae; g__Limnobacter; s__uncultured_beta_proteobacterium
1431	0	0	0	0	0	0	0	0	0	2	0	0	k__Bacteria; p__Proteobacteria; c__Gammaproteobacteria; o__Xanthomonadales; f__JTB255_marine_benthic_group; g__uncultured_gamma_proteobacterium; s__uncultured_gamma_proteobacterium
1062	0	6	0	0	0	0	0	0	0	0	0	0	k__Bacteria; p__Bacteroidetes; c__Flavobacteriia; o__Flavobacteriales; f__Flavobacteriaceae; g__Aquimarina; Ambiguous_taxa
1021	0	9	0	0	0	0	0	0	0	0	0	0	k__Bacteria; p__Bacteroidetes; c__Cytophagia; o__Order_III; f__B2706_C7; g__uncultured_organism; s__uncultured_organism
1801	0	1	0	0	0	0	6	10	5	1	0	0	k__Bacteria; p__Spirochaetae; c__Spirochaetes; o__Spirochaetales; f__Spirochaetaceae; g__Spirochaeta_2; s__Spirochaeta_isovalerica

1473	0	0	0	0	0	0	0	0	0	5	3	0	k__Bacteria; p__Firmicutes; c__Clostridia; o__Clostridiales; f__Ruminococcaceae; g__uncultured
1432	1	0	0	0	0	0	0	0	0	19	0	0	k__Bacteria; p__Firmicutes; c__Clostridia; o__Clostridiales; f__Ruminococcaceae; g__Ruminococcaceae_UCG_014
1063	0	11	0	0	0	0	0	0	0	0	0	0	k__Bacteria; p__Proteobacteria; c__Deltaproteobacteria; o__Desulfobacterales; f__Desulfobulbaceae
1022	0	2	5	2	1	6	0	0	0	0	0	0	k__Bacteria; p__Bacteroidetes; c__Flavobacteriia; o__Flavobacteriales; f__Flavobacteriaceae; g__Lutibacter; Ambiguous_taxa
1802	0	0	0	0	0	0	0	0	12	0	0	0	k__Bacteria; p__Fusobacteria; c__Fusobacteriia; o__Fusobacteriales; f__Leptotrichiaceae; g__uncultured; s__uncultured_Fusobacterium_sp.
1474	0	0	0	0	0	0	0	0	0	0	3	1	k__Bacteria; p__Firmicutes; c__Clostridia; o__Clostridiales; f__Ruminococcaceae
1433	30	12	45	3	0	2	0	1	3	6	5	0	k__Bacteria; p__Proteobacteria; c__Alphaproteobacteria; o__Rhodobacterales; f__Rhodobacteraceae; g__Marivita; Ambiguous_taxa
1064	0	23	22	7	2	0	0	0	0	0	0	0	k__Bacteria; p__Proteobacteria; c__Gammaproteobacteria; o__Xanthomonadales; f__JTB255_marine_benthic_group; g__uncultured_sediment_bacterium; s__uncultured_sediment_bacterium
1023	0	7	25	2	1	7	0	0	0	0	1	0	k__Bacteria; p__Proteobacteria; c__Deltaproteobacteria; o__Desulfobacterales; f__Desulfobacteraceae
1803	0	0	0	0	0	0	0	0	16	0	0	0	k__Bacteria; p__PAUC34f
1475	0	0	0	0	85	69	36	86	1	53	52	60	k__Bacteria; p__Firmicutes; c__Clostridia; o__Clostridiales; f__Lachnospiraceae

1434	0	0	0	0	0	0	0	0	1	66	0	0	k__Bacteria; p__Firmicutes; c__Clostridia; o__Clostridiales; f__Ruminococcaceae; g__Ruminococcaceae_UCG_005; s__uncultured_organism
1065	0	4	4	0	1	0	2	6	1	0	0	0	k__Bacteria; p__Proteobacteria; c__Gammaproteobacteria; o__Cellvibrionales; f__Halieaceae; g__Haliea; s__uncultured_bacterium
1024	1	27	19	0	0	0	0	0	0	0	0	0	k__Bacteria; p__Bacteroidetes; c__Bacteroidetes_BD2_2
1804	0	0	0	0	0	0	3	0	9	0	0	0	k__Bacteria; p__Proteobacteria; c__Gammaproteobacteria; o__Alteromonadales; f__Colwelliaceae; g__Thalassotalea
1476	0	0	1	0	0	0	0	0	0	3	0	12	k__Bacteria; p__Firmicutes; c__Clostridia; o__Clostridiales; f__Lachnospiraceae; g__uncultured; s__unidentified
1435	0	0	0	0	0	0	0	0	0	3	0	0	k__Bacteria; p__Firmicutes; c__Clostridia; o__Clostridiales; f__Lachnospiraceae; g__Syntrophococcus; s__uncultured_bacterium
1066	1	22	41	0	0	0	8	1	4	0	0	0	k__Bacteria; p__Proteobacteria; c__Alphaproteobacteria; o__Rhodobacterales; f__Rhodobacteraceae
1025	0	8	0	0	1	0	0	0	0	0	0	0	k__Bacteria; p__Proteobacteria; c__Deltaproteobacteria; o__Desulfobacterales; f__Desulfobulbaceae; g__Desulfobulbus
1477	0	0	0	0	0	0	474	559	533	0	0	0	k__Bacteria; p__Bacteroidetes; c__Flavobacteriia; o__Flavobacteriales; f__Cryomorphaceae; g__Owenweeksia
1436	0	0	0	0	0	0	0	0	0	33	1	0	k__Bacteria; p__Firmicutes; c__Clostridia; o__Clostridiales; f__Ruminococcaceae; g__Ruminococcaceae_NK4A214_group
1067	0	0	5	0	0	0	0	0	0	0	0	0	k__Bacteria; p__Bacteroidetes; c__Flavobacteriia; o__Flavobacteriales; f__Cryomorphaceae; g__Owenweeksia
1026	0	11	0	0	0	0	0	0	0	0	0	0	k__Bacteria; p__Actinobacteria; c__Coriobacteriia; o__Coriobacteriales; f__Coriobacteriaceae; g__uncultured

1478	0	0	0	0	0	0	15	38	21	0	0	0	k__Bacteria; p__Bacteroidetes
1437	0	0	0	0	0	0	0	0	0	2	0	0	k__Bacteria; p__Bacteroidetes
1068	0	2	12	0	0	0	3	0	1	0	0	0	k__Bacteria; p__Proteobacteria; c__Gammaproteobacteria; o__Cellvibrionales; f__BD2_7; g__uncultured_bacterium; s__uncultured_bacterium
1027	0	2	19	0	4	0	0	0	0	0	0	0	k__Bacteria; p__Proteobacteria; c__Deltaproteobacteria; o__Desulfobacterales; f__Desulfobulbaceae
1479	0	0	0	0	0	0	28	31	47	0	0	0	k__Bacteria; p__Proteobacteria; c__Deltaproteobacteria; o__Desulfobacterales; f__Desulfobacteraceae; g__Desulfobacterium
1438	0	0	0	0	0	0	0	0	0	30	0	0	k__Bacteria; p__Bacteroidetes; c__Bacteroidia; o__Bacteroidales; f__Bacteroidales_S24_7_group; g__uncultured_bacterium; s__uncultured_bacterium
1069	0	0	38	0	0	0	1	0	0	4	0	0	k__Bacteria; p__Firmicutes; c__Clostridia; o__Clostridiales; f__Ruminococcaceae; g__Ruminococcaceae_UCG_002
1028	0	5	12	0	0	1	0	0	0	0	0	0	k__Bacteria; p__Tenericutes; c__Mollicutes; o__NB1_n
1439	0	0	0	0	0	0	0	0	0	37	0	0	k__Bacteria; p__Bacteroidetes; c__Bacteroidia; o__Bacteroidales; f__Bacteroidales_S24_7_group; g__uncultured_bacterium; s__uncultured_bacterium
1029	2	12	25	0	0	0	0	0	0	0	0	0	k__Bacteria; p__Chlorobi; c__Ignavibacteria; o__Ignavibacteriales; f__IheB3_7; Ambiguous_taxa; Ambiguous_taxa
690	3	23	36	0	0	0	0	0	0	0	0	0	k__Bacteria; p__Proteobacteria; c__Gammaproteobacteria; o__Cellvibrionales; f__Halieaceae
280	0	0	0	0	0	0	0	0	0	0	2	0	k__Bacteria; p__Bacteroidetes; c__Bacteroidia; o__Bacteroidales; f__Prevotellaceae; g__Prevotella_9;

													s__uncultured_bacterium
691	46	197	146	2	4	3	0	2	0	0	0	0	k__Bacteria; p__Bacteroidetes; c__Flavobacteriia; o__Flavobacteriales; f__Flavobacteriaceae; g__Psychroserpens; Ambiguous_taxa
281	0	203	0	32	0	39	1	0	0	23	0	0	k__Bacteria; p__Bacteroidetes; c__Bacteroidia; o__Bacteroidales; f__Prevotellaceae; g__Alloprevotella; s__uncultured_bacterium
692	31	8	20	0	2	0	7	2	3	1	0	0	k__Bacteria; p__Proteobacteria; c__Gammaproteobacteria; o__Cellvibrionales; f__Halieaceae; g__Pseudohaliea; s__uncultured_organism
610	0	0	0	0	0	6	0	0	0	0	0	0	k__Bacteria; p__Proteobacteria; c__Gammaproteobacteria; o__Alteromonadales; f__Colwelliaceae
241	325	335	1	159	39	82	127	0	40	42	92	103	k__Bacteria; p__Bacteroidetes; c__Bacteroidia; o__Bacteroidales; f__Porphyromonadaceae; g__Odoribacter; s__uncultured_bacterium
200	0	259	0	0	25	60	0	0	45	11	29	20	k__Bacteria; p__Firmicutes; c__Clostridia; o__Clostridiales; f__Lachnospiraceae; g__Marvinbryantia; s__uncultured_bacterium
693	5	11	26	0	0	0	0	0	0	0	0	0	k__Bacteria; p__Bacteroidetes; c__Flavobacteriia; o__Flavobacteriales; f__Flavobacteriaceae
652	0	0	25	0	0	1	0	0	0	4	12	4	k__Bacteria; p__Firmicutes; c__Clostridia; o__Clostridiales; f__Lachnospiraceae; g__Roseburia; s__uncultured_bacterium
611	2	30	39	4	1	8	0	1	0	0	0	0	k__Bacteria; p__Proteobacteria; c__Gammaproteobacteria; o__Cellvibrionales; f__Halieaceae; g__Halioglobus; Ambiguous_taxa

242	0	0	35	0	0	0	1	1	0	29	0	0	k__Bacteria; p__Bacteroidetes; c__Bacteroidia; o__Bacteroidales; f__Prevotellaceae; g__Prevotellaceae_NK3B31_group; s__uncultured_bacterium
201	0	0	0	20	41	30	0	0	1	32	2	8	k__Bacteria; p__Firmicutes; c__Negativicutes; o__Selenomonadales; f__Veillonellaceae; g__Mitsuokella; s__uncultured_bacterium
694	43	111	209	3	1	2	2	14	8	0	0	0	k__Bacteria; p__Proteobacteria; c__Alphaproteobacteria; o__Rhodobacterales; f__Rhodobacteraceae
612	1	6	8	3	0	8	0	0	0	0	0	0	k__Bacteria; p__Bacteroidetes; c__Bacteroidetes_VC2.1_Bac22
284	0	0	0	1	27	0	0	0	0	2	5	0	k__Bacteria; p__Bacteroidetes; c__Bacteroidia; o__Bacteroidales; f__Bacteroidales_S24_7_group; g__uncultured_bacterium; s__uncultured_bacterium
243	0	4	20	0	2	0	0	0	0	0	0	0	k__Bacteria; p__Acidobacteria; c__Holophagae; o__Subgroup_23; Ambiguous_taxa; Ambiguous_taxa; Ambiguous_taxa
202	0	0	0	41	0	63	0	0	0	6	2	0	k__Bacteria; p__Firmicutes; c__Clostridia; o__Clostridiales; f__Ruminococcaceae; g__Ruminiclostridium_5; s__uncultured_bacterium
695	0	0	6	0	0	0	0	0	0	0	0	0	k__Bacteria; p__Proteobacteria; c__Gammaproteobacteria; o__Arenicellales; f__Arenicellaceae; g__Arenicella; s__uncultured_bacterium
654	0	0	0	0	0	0	0	0	0	30	0	0	k__Bacteria; p__Bacteroidetes; c__Bacteroidia; o__Bacteroidales; f__Prevotellaceae; g__Prevotellaceae_NK3B31_group; s__uncultured_bacterium
613	0	0	0	0	0	2	0	0	0	0	0	0	k__Bacteria; p__Proteobacteria; c__Deltaproteobacteria; o__Desulfobacterales; f__Desulfobacteraceae

285	0	0	0	0	0	25	0	0	0	0	9	0	k__Bacteria; p__Firmicutes; c__Clostridia; o__Clostridiales; f__Clostridiales_vadinBB60_group; g__uncultured_bacterium; s__uncultured_bacterium
244	146	0	0	0	0	0	1	0	0	1	22	5	k__Bacteria; p__Bacteroidetes; c__Bacteroidia; o__Bacteroidales; f__Bacteroidales_S24_7_group; Ambiguous_taxa; Ambiguous_taxa
203	0	1	5	28	0	6	3	16	0	0	0	0	k__Bacteria; p__Firmicutes; c__Bacilli; o__Lactobacillales; f__Streptococcaceae; g__Lactococcus
696	0	22	2	0	0	0	0	0	0	0	0	0	k__Bacteria; p__Proteobacteria; c__Gammaproteobacteria; o__Cellvibrionales; f__Halieaceae
655	20	36	69	24	4	0	0	0	0	0	0	0	k__Bacteria; p__Proteobacteria; c__Gammaproteobacteria; o__Order_Incertae_Sedis; f__Family_Incertae_Sedis; g__Marinicella; s__bacterium_enrichment_culture_clone_25(2013)
614	69	253	207	11	17	42	2	0	0	0	0	0	k__Bacteria; p__Bacteroidetes; c__Flavobacteriia; o__Flavobacteriales; f__Flavobacteriaceae; g__Formosa
286	0	0	0	99	0	73	0	0	0	10	12	16	k__Bacteria; p__Firmicutes; c__Clostridia; o__Clostridiales; f__Clostridiales_vadinBB60_group; Ambiguous_taxa; Ambiguous_taxa
245	0	0	0	0	1	0	47	1	65	11	6	0	k__Bacteria; p__Firmicutes; c__Negativicutes; o__Selenomonadales; f__Veillonellaceae; g__Anaerovibrio
697	12	73	135	2	0	2	1	14	2	1	0	0	k__Bacteria; p__Proteobacteria; c__Alphaproteobacteria; o__Rhodobacterales; f__Rhodobacteraceae
656	0	0	0	0	0	0	0	0	0	6	8	0	k__Bacteria; p__Firmicutes; c__Clostridia; o__Clostridiales; f__Lachnospiraceae; g__uncultured

615	0	0	11	0	0	5	5	2	11	0	0	0	k__Bacteria; p__Proteobacteria; c__Gammaproteobacteria; o__Order_Incertae_Sedis; f__Family_Incertae_Sedis; g__Marinicella
287	4	3	12	0	0	2	1	2	0	0	0	0	k__Bacteria; p__Proteobacteria; c__Gammaproteobacteria; o__Chromatiales; f__Granulosicoccaceae; g__Granulosicoccus; s__uncultured_gamma_proteobacterium
246	0	2	11	7	1	11	0	0	0	0	0	0	k__Bacteria; p__Spirochaetae; c__Spirochaetes; o__Spirochaetales; f__Spirochaetaceae; g__Spirochaeta_2
205	0	0	1	48	34	122	0	195	87	41	72	29	k__Bacteria; p__Bacteroidetes; c__Bacteroidia; o__Bacteroidales; f__Bacteroidales_S24_7_group
698	0	0	9	4	1	7	39	1	7	0	0	0	k__Bacteria; p__Proteobacteria; c__Gammaproteobacteria; o__Oceanospirillales; f__Oceanospirillaceae; g__Reinekea; s__uncultured_gamma_proteobacterium
657	0	4	11	0	0	0	0	0	0	0	0	0	k__Bacteria; p__Bacteroidetes; c__Flavobacteriia; o__Flavobacteriales; f__Flavobacteriaceae; g__Tenacibaculum
616	0	0	2	0	0	11	1	11	1	1	0	0	k__Bacteria; p__Proteobacteria; c__Deltaproteobacteria; o__Desulfobacterales; f__Desulfobacteraceae; g__Desulfobacula
288	180	0	3	18	41	149	114	0	85	87	123	94	k__Bacteria; p__Proteobacteria; c__Deltaproteobacteria; o__Desulfovibrionales; f__Desulfovibrionaceae; g__Desulfovibrio; s__uncultured_bacterium
247	0	0	0	0	0	0	0	0	0	29	0	0	k__Bacteria; p__Bacteroidetes; c__Bacteroidia; o__Bacteroidales; f__Porphyromonadaceae; g__Parabacteroides; s__Porphyromonadaceae_bacterium_DJF_B175
206	0	0	0	0	0	0	57	94	0	0	3	4	k__Bacteria; p__Firmicutes; c__Bacilli; o__Bacillales; f__Family_XI; g__Gemella; Ambiguous_taxa
699	50	90	127	0	0	0	0	0	0	0	0	0	k__Bacteria; p__Proteobacteria; c__Alphaproteobacteria;

													o__Rhodobacterales; f__Rhodobacteraceae
658	0	0	8	0	0	0	0	0	0	0	0	0	k__Bacteria; p__Proteobacteria; c__Deltaproteobacteria; o__Bdellovibrionales; f__Bdellovibrionaceae; g__OM27_clade; s__uncultured_bacterium
617	0	0	6	4	1	9	1	0	0	0	0	0	k__Bacteria; p__Proteobacteria; c__Deltaproteobacteria; o__SAR324_clade(Marine_group_B)
289	0	0	23	0	2	67	0	0	0	44	19	12	k__Bacteria; p__Firmicutes; c__Clostridia; o__Clostridiales; f__Lachnospiraceae
248	0	1	9	23	18	26	0	0	0	0	0	0	k__Bacteria; p__Proteobacteria; c__Gammaproteobacteria; o__Thiotrichales; f__Thiotrichaceae; g__uncultured
207	0	0	0	0	0	0	0	0	0	38	0	0	k__Bacteria; p__Firmicutes; c__Clostridia; o__Clostridiales; f__Ruminococcaceae; g__Butyricicoccus; Ambiguous_taxa
618	0	0	0	1	38	61	0	38	0	34	12	15	k__Bacteria; p__Firmicutes; c__Clostridia; o__Clostridiales; f__Lachnospiraceae
249	0	0	0	0	0	1	0	0	0	98	4	5	k__Bacteria; p__Firmicutes; c__Clostridia; o__Clostridiales; f__Lachnospiraceae; g__Blautia
208	0	0	0	0	16	0	22	0	2	26	0	0	k__Bacteria; p__Proteobacteria; c__Betaproteobacteria; o__Neisseriales; f__Neisseriaceae; g__Leeia; s__uncultured_bacterium
619	0	0	0	0	0	0	0	28	2	29	7	3	k__Bacteria; p__Proteobacteria; c__Deltaproteobacteria; o__Desulfovibrionales; f__Desulfovibrionaceae; g__Desulfovibrio; s__uncultured_Desulfovibrio_sp.
209	0	0	1	0	0	0	27	38	0	18	8	1	k__Bacteria; p__Firmicutes; c__Clostridia; o__Clostridiales; f__Lachnospiraceae; g__Roseburia; s__uncultured_bacterium
1770	0	0	0	0	0	0	0	1	56	0	0	0	k__Bacteria; p__Bacteroidetes; c__Sphingobacteriia; o__Sphingobacteriales; f__LiUU_11_161;

													g__uncultured_bacterium; s__uncultured_bacterium
1360	0	0	0	0	3	0	0	0	13	0	2	1	k__Bacteria; p__Fusobacteria; c__Fusobacteriia; o__Fusobacteriales; f__Fusobacteriaceae; g__Psychrilyobacter
1771	0	0	0	0	0	0	0	0	41	0	0	0	k__Bacteria; p__Firmicutes; c__Clostridia; o__Clostridiales; f__Lachnospiraceae
1361	0	0	0	0	0	0	0	0	0	0	1	0	k__Bacteria; p__Proteobacteria; c__Gammaproteobacteria; o__Aeromonadales; f__Succinivibrionaceae; g__Anaerobiospirillum; Ambiguous_taxa
1320	0	0	0	0	0	0	0	0	0	2	1	1	k__Bacteria; p__Fusobacteria; c__Fusobacteriia; o__Fusobacteriales
1772	0	0	1	0	0	0	0	0	4	0	0	0	k__Bacteria; p__Bacteroidetes; c__Sphingobacteriia; o__Sphingobacteriales; f__Saprospiraceae; g__uncultured
1731	0	0	0	0	0	0	4	6	4	0	0	0	k__Bacteria; p__Bacteroidetes; c__Cytophagia; o__Cytophagales; f__Flammeovirgaceae; g__Reichenbachiella
1362	0	2	0	0	0	0	0	0	0	0	0	0	k__Bacteria; p__Proteobacteria; c__Alphaproteobacteria; o__Sphingomonadales; f__Sphingomonadaceae; g__Sphingomonas
1321	0	0	0	2	0	0	4	1	0	0	0	0	k__Bacteria; p__Proteobacteria; c__Deltaproteobacteria; o__Desulfobacterales; f__Desulfobulbaceae; g__Desulfobulbus
1773	0	0	0	0	0	0	0	0	32	0	0	0	k__Bacteria
1363	0	0	0	0	0	1	0	0	2	38	5	2	k__Bacteria; p__Firmicutes; c__Clostridia; o__Clostridiales; f__Clostridiaceae_1; g__Clostridium_sensu_stricto_6; Ambiguous_taxa
1322	2	1	14	1	0	2	5	4	11	1	1	0	k__Bacteria; p__Proteobacteria; c__Gammaproteobacteria; o__Alteromonadales; f__Shewanellaceae; g__Shewanella

1774	0	0	0	0	0	0	0	0	51	0	0	0	k__Bacteria; p__Bacteroidetes; c__Bacteroidia; o__Bacteroidales; f__Bacteroidaceae; g__Bacteroides
1733	0	0	0	0	0	0	16	0	7	0	1	0	k__Bacteria; p__Proteobacteria; c__Gammaproteobacteria; o__Alteromonadales; f__Ferrimonadaceae; g__Ferrimonas; Ambiguous_taxa
1364	0	0	1	0	0	0	4	3	9	2	0	1	k__Bacteria; p__Proteobacteria; c__Alphaproteobacteria; o__Rhodospirillales; f__Rhodospirillaceae; g__Thalassospira; Ambiguous_taxa
1323	0	0	0	0	0	0	9	5	3	10	0	1	k__Bacteria; p__Bacteroidetes; c__Bacteroidia; o__Bacteroidales; f__Marinilabiaceae; g__Marinifilum; s__uncultured_bacterium
1775	0	0	0	0	0	0	0	3	7	0	0	0	k__Bacteria; p__Gemmatimonadetes; c__Gemmatimonadetes; o__Gemmatimonadales; f__Gemmatimonadaceae; g__uncultured; s__uncultured_actinobacterium
1734	0	0	0	0	0	0	0	0	10	0	0	0	k__Bacteria; p__Bacteroidetes; c__Flavobacteriia; o__Flavobacteriales; f__Flavobacteriaceae; g__Aquimarina; s__Flavobacteriaceae_bacterium_1ta6
1324	0	0	0	0	0	0	0	0	0	0	1	0	k__Bacteria; p__Proteobacteria; c__Gammaproteobacteria; o__Oceanospirillales; f__Oceanospirillaceae; g__Marinobacterium
1776	0	0	0	0	0	0	0	0	8	0	0	0	k__Bacteria; p__Proteobacteria; c__Deltaproteobacteria; o__Desulfobacterales; f__Desulfobulbaceae; g__Desulfobulbus
1735	0	0	0	0	0	0	2	0	11	0	0	0	k__Bacteria; p__Proteobacteria; c__Gammaproteobacteria; o__CK_1C4_49; f__gamma_proteobacterium_SS_5; g__gamma_proteobacterium_SS_5; s__gamma_proteobacterium_SS_5

1366	29	106	86	0	0	0	319	217	290	7	1	3	k__Bacteria; p__Proteobacteria; c__Gammaproteobacteria; o__Vibrionales; f__Vibrionaceae; g__Vibrio
1325	0	3	0	0	0	0	0	0	0	0	0	0	k__Bacteria; p__Bacteroidetes; c__Flavobacteriia; o__Flavobacteriales; f__Cryomorphaceae; g__Owenweeksia; s__uncultured_Bacteroidetes_bacterium
1777	0	0	1	0	6	0	0	0	12	0	0	0	k__Bacteria; p__Actinobacteria; c__Acidimicrobiia; o__Acidimicrobiales; f__OM1_clade; g__uncultured_bacterium; s__uncultured_bacterium
1736	0	0	0	0	0	0	0	111	0	10	3	0	k__Bacteria; p__Firmicutes; c__Clostridia; o__Clostridiales; f__Lachnospiraceae; g__Incertae_Sedis; s__uncultured_bacterium
1326	3	23	41	11	1	1	0	0	0	7	5	1	k__Bacteria; p__Proteobacteria; c__Gammaproteobacteria; o__Vibrionales; f__Vibrionaceae; g__Vibrio
1778	0	0	0	0	0	0	1	1	14	0	0	0	k__Bacteria; p__Proteobacteria; c__Deltaproteobacteria; o__Oligoflexales; f__Oligoflexaceae; g__uncultured_bacterium; s__uncultured_bacterium
1737	0	0	0	0	0	0	0	4	0	0	0	0	k__Bacteria; p__Firmicutes; c__Clostridia; o__Clostridiales; f__Family_XIII; g__[Eubacterium]_nodatum_group
1368	0	0	0	0	0	1	2	5	0	0	0	0	k__Bacteria; p__Firmicutes; c__Bacilli
1327	0	0	0	0	0	0	0	0	1	0	0	0	k__Bacteria; p__Spirochaetae; c__Spirochaetes; o__Spirochaetales; f__Spirochaetaceae; g__Spirochaeta_2; s__uncultured_bacterium
1779	0	0	0	0	0	0	0	0	8	0	0	0	k__Bacteria; p__Spirochaetae; c__Spirochaetes; o__Spirochaetales; f__Spirochaetaceae; g__Spirochaeta_2
1738	0	0	0	0	0	0	2	52	0	0	4	0	k__Bacteria; p__Firmicutes; c__Erysipelotrichia; o__Erysipelotrichales; f__Erysipelotrichaceae;

													g__Solobacterium; s__uncultured_bacterium
1369	0	0	0	0	0	0	0	54	0	8	9	0	k__Bacteria; p__Firmicutes; c__Erysipelotrichia; o__Erysipelotrichales; f__Erysipelotrichaceae; g__uncultured; Ambiguous_taxa
1739	0	0	0	0	0	0	0	3	0	0	0	0	k__Bacteria; p__Proteobacteria; c__FGL7S; o__uncultured_bacterium; f__uncultured_bacterium; g__uncultured_bacterium; s__uncultured_bacterium
1329	0	0	0	0	0	0	0	0	0	1	1	0	k__Bacteria; p__Bacteroidetes
60	179	0	41	28	28	53	0	0	0	24	19	1	k__Bacteria; p__Firmicutes; c__Clostridia; o__Clostridiales; f__Clostridiales_vadinBB60_group; g__uncultured_bacterium; s__uncultured_bacterium
990	0	17	8	0	0	0	0	0	0	0	0	0	k__Bacteria; p__Actinobacteria; c__Acidimicrobiia; o__Acidimicrobiales; f__OM1_clade; g__uncultured_bacterium; s__uncultured_bacterium
61	405	169	32	1	248	339	40	0	120	127	88	42	k__Bacteria; p__Firmicutes; c__Clostridia; o__Clostridiales; f__Lachnospiraceae; g__Lachnospiraceae_NK4A136_group; s__unidentified
580	0	0	6	6	5	16	0	0	0	0	0	0	k__Bacteria; p__Proteobacteria; c__Alphaproteobacteria; o__Rhodobacterales; f__Rhodobacteraceae; g__Pseudophaeobacter; Ambiguous_taxa
20	930	657	389	552	746	412	536	372	472	382	726	355	k__Bacteria; p__Bacteroidetes; c__Bacteroidia; o__Bacteroidales; f__Bacteroidales_S24_7_group; g__uncultured_Bacteroidales_bacterium; s__uncultured_Bacteroidales_bacterium
170	0	0	2	50	114	4	0	80	1	22	79	68	k__Bacteria; p__Bacteroidetes; c__Bacteroidia;

													o__Bacteroidales; f__Rikenellaceae; g__Alistipes; s__uncultured_bacterium
991	4	26	35	1	4	6	15	8	15	3	0	0	k__Bacteria; p__Proteobacteria; c__Gammaproteobacteria; o__Alteromonadales; f__Alteromonadaceae; g__Aestuariibacter; Ambiguous_taxa
950	1	9	2	0	0	0	0	1	0	0	0	0	k__Bacteria; p__Bacteroidetes; c__Sphingobacteriia; o__Sphingobacteriales; f__NS11_12_marine_group; Ambiguous_taxa; Ambiguous_taxa
62	758	114	186	252	369	302	172	231	320	152	516	206	k__Bacteria; p__Bacteroidetes; c__Bacteroidia; o__Bacteroidales; f__Bacteroidales_S24_7_group; g__uncultured_bacterium; s__uncultured_bacterium
581	1	139	0	0	0	24	0	0	0	0	0	0	k__Bacteria; p__Firmicutes; c__Clostridia; o__Clostridiales; f__Lachnospiraceae
540	93	94	376	3	7	16	6	15	15	1	0	0	k__Bacteria; p__Proteobacteria; c__Proteobacteria_Incertae_Sedis; o__Unknown_Order; f__Unknown_Family; g__Candidatus_Thiobios; s__uncultured_gamma_proteobacterium
21	2948	3526	777	3193	3039	2594	1316	2093	1181	984	3430	1940	k__Bacteria; p__Firmicutes; c__Bacilli; o__Lactobacillales
171	0	65	0	97	0	0	22	96	35	116	22	6	k__Bacteria; p__Bacteroidetes; c__Bacteroidia; o__Bacteroidales; f__Prevotellaceae; g__Prevotellaceae_NK3B31_group; s__Prevotella_sp._P4_76
130	0	105	0	52	24	73	0	0	47	20	15	0	k__Bacteria; p__Firmicutes; c__Clostridia; o__Clostridiales; f__Ruminococcaceae; g__Ruminococcaceae_NK4A214_group; Ambiguous_taxa
992	1	90	0	0	0	0	0	1	0	556	6	1	k__Bacteria; p__Firmicutes; c__Clostridia; o__Clostridiales; f__Ruminococcaceae; g__Ruminococcaceae_UCG_014;

													s__uncultured_rumen_bacterium
951	0	4	2	0	0	0	0	0	0	0	0	0	k__Bacteria; p__Acidobacteria; c__Holophagae; o__Subgroup_23
910	0	29	0	0	0	0	0	0	0	0	0	0	k__Bacteria; p__Proteobacteria; c__Deltaproteobacteria; o__Desulfuromonadales; f__GR_WP33_58
63	0	282	101	30	156	210	104	132	144	259	58	16	k__Bacteria; p__Firmicutes; c__Clostridia; o__Clostridiales; f__Lachnospiraceae; g__Lachnospiraceae_NK4A136_group; s__uncultured_bacterium
582	0	0	0	0	12	66	0	0	0	0	5	3	k__Bacteria; p__Proteobacteria; c__Deltaproteobacteria; o__Desulfovibrionales; f__Desulfovibrionaceae; g__Bilophila; s__uncultured_bacterium
541	0	33	29	1	2	12	0	0	0	0	0	0	k__Bacteria; p__Bacteroidetes; c__Sphingobacteriia; o__Sphingobacteriales; f__Saprospiraceae; g__Lewinella; s__uncultured_Bacteroidetes_bacterium
500	0	0	0	0	30	165	201	0	75	2	5	5	k__Bacteria; p__Bacteroidetes; c__Bacteroidia; o__Bacteroidales; f__Bacteroidales_S24_7_group; g__uncultured_bacterium; s__uncultured_bacterium
22	195	0	0	177	90	74	41	84	110	32	45	42	k__Bacteria; p__Firmicutes; c__Clostridia; o__Clostridiales; f__Lachnospiraceae; g__Lachnospiraceae_NK4A136_group; s__uncultured_bacterium
172	428	0	0	1	74	1	0	0	1	11	79	104	k__Bacteria; p__Bacteroidetes; c__Bacteroidia; o__Bacteroidales; f__Rikenellaceae; g__Rikenellaceae_RC9_gut_group; s__uncultured_bacterium
131	95	336	81	154	249	276	195	291	271	75	91	10	k__Bacteria; p__Bacteroidetes; c__Bacteroidia; o__Bacteroidales; f__Prevotellaceae; g__Prevotella_7;

													s__uncultured_bacterium
993	0	10	1	0	0	0	0	0	0	0	0	0	k__Bacteria; p__Proteobacteria; c__Gammaproteobacteria; o__Thiotrichales; f__Thiotrichales_Incertae_Sedis; g__Candidatus_Endoecteinascidia; s__uncultured_bacterium
952	0	11	3	1	0	0	0	0	0	0	0	0	k__Bacteria; p__Acidobacteria; c__Subgroup_22; Ambiguous_taxa; Ambiguous_taxa; Ambiguous_taxa; Ambiguous_taxa
911	0	16	24	0	2	0	0	0	0	0	0	0	k__Bacteria; p__Bacteroidetes; c__Flavobacteriia; o__Flavobacteriales; f__Flavobacteriaceae
64	0	0	42	126	59	2	87	1	83	23	146	36	k__Bacteria; p__Bacteroidetes; c__Bacteroidia; o__Bacteroidales
583	0	0	0	6	0	12	0	0	1	0	0	0	k__Bacteria; p__Proteobacteria; c__Gammaproteobacteria; o__Oceanospirillales; f__OM182_clade
542	4	0	5	1	11	0	0	0	0	0	0	0	k__Bacteria; p__Proteobacteria; c__Deltaproteobacteria; o__Desulfobacterales; f__Desulfobulbaceae; g__uncultured; Ambiguous_taxa
501	0	253	50	34	111	75	92	49	0	42	52	30	k__Bacteria; p__Firmicutes; c__Clostridia; o__Clostridiales; f__Ruminococcaceae
23	0	1	86	0	0	0	0	0	0	0	3	0	k__Bacteria; p__Actinobacteria; c__Coriobacteriia; o__Coriobacteriales; f__Coriobacteriaceae; g__Parvibacter; Ambiguous_taxa
173	0	0	0	0	0	60	2	0	1	13	4	0	k__Bacteria; p__Firmicutes; c__Clostridia; o__Clostridiales; f__Lachnospiraceae; g__Fusicatenibacter
132	0	0	0	0	0	0	35	0	0	109	2	0	k__Bacteria; p__Firmicutes; c__Clostridia; o__Clostridiales; f__Ruminococcaceae; g__Ruminiclostridium_5;

													Ambiguous_taxa
994	0	5	0	0	0	0	0	0	0	0	0	0	k__Bacteria; p__Bacteroidetes; c__Bacteroidetes_BD2_2
953	0	37	2	0	0	0	0	0	0	0	0	0	k__Bacteria; p__Proteobacteria
912	0	93	0	0	0	0	0	0	0	0	3	5	k__Bacteria; p__Firmicutes; c__Clostridia; o__Clostridiales; f__Peptococcaceae; g__Peptococcus; s__uncultured_bacterium
65	268	141	46	97	238	192	0	22	43	80	146	20	k__Bacteria; p__Actinobacteria; c__Coriobacteriia; o__Coriobacteriales; f__Coriobacteriaceae; g__Enterorhabdus
584	0	0	0	1	0	6	0	0	0	0	0	0	k__Bacteria; p__Bacteroidetes; c__Flavobacteriia; o__Flavobacteriales; f__Flavobacteriaceae; g__NS4_marine_group
543	0	0	0	0	22	0	73	0	0	27	6	3	k__Bacteria; p__Firmicutes; c__Clostridia; o__Clostridiales; f__Lachnospiraceae; g__Roseburia; s__uncultured_bacterium
502	252	155	52	0	35	84	0	0	1	14	40	8	k__Bacteria; p__Bacteroidetes; c__Bacteroidia; o__Bacteroidales; f__Bacteroidales_S24_7_group
24	356	517	57	329	224	322	203	0	72	179	245	178	k__Bacteria; p__Firmicutes; c__Clostridia; o__Clostridiales; f__Lachnospiraceae; g__Lachnoclostridium
174	105	0	0	0	0	0	0	1	1	62	0	0	k__Bacteria; p__Firmicutes; c__Clostridia; o__Clostridiales; f__Ruminococcaceae; g__Oscillospira; Ambiguous_taxa
133	29	374	158	192	278	113	87	193	215	83	66	20	k__Bacteria; p__Firmicutes; c__Bacilli; o__Lactobacillales; f__Enterococcaceae; g__Enterococcus
995	1	4	5	0	0	0	0	0	0	0	0	0	k__Bacteria; p__Proteobacteria; c__Deltaproteobacteria; o__Myxococcales; f__P3OB_42; g__uncultured_bacterium; s__uncultured_bacterium
954	0	9	2	1	0	2	0	0	0	0	0	0	k__Bacteria; p__Bacteroidetes; c__Bacteroidia; o__Bacteroidales; f__Marinilabiaceae; g__Marinifilum;

													Ambiguous_taxa
913	2	19	19	1	0	0	2	0	0	14	5	1	k__Bacteria; p__Proteobacteria; c__Gammaproteobacteria; o__Oceanospirillales; f__Oceanospirillaceae; g__Marinomonas; Ambiguous_taxa
66	106	0	149	47	0	75	3	113	8	173	5	1	k__Bacteria; p__Bacteroidetes; c__Bacteroidia; o__Bacteroidales; f__Bacteroidales_S24_7_group; Ambiguous_taxa; Ambiguous_taxa
585	2	3	7	2	0	7	0	0	0	0	0	0	k__Bacteria; p__Proteobacteria; c__Gammaproteobacteria; o__Pseudomonadales; f__Moraxellaceae; g__Acinetobacter
544	13	4	0	0	6	5	0	0	1	0	0	0	k__Bacteria; p__Actinobacteria; c__Acidimicrobiia; o__Acidimicrobiales; f__Acidimicrobiaceae; g__Illumatobacter; Ambiguous_taxa
503	0	0	0	0	33	66	0	0	0	9	26	6	k__Bacteria; p__Firmicutes; c__Clostridia; o__Clostridiales; f__Lachnospiraceae; g__Roseburia; s__uncultured_Clostridiales_bacterium
25	1149	498	180	166	366	313	318	438	412	139	99	8	k__Bacteria; p__Bacteroidetes; c__Bacteroidia; o__Bacteroidales; f__Prevotellaceae; g__Prevotella_1
175	13	4	3	4	0	69	0	0	1	38	1	0	k__Bacteria; p__Firmicutes; c__Clostridia; o__Clostridiales; f__Peptostreptococcaceae; g__Terrisporobacter; s__uncultured_bacterium
134	0	114	0	65	31	1	2	55	0	103	4	1	k__Bacteria; p__Bacteroidetes; c__Bacteroidia; o__Bacteroidales; f__Prevotellaceae; g__Prevotellaceae_NK3B31_group; s__uncultured_bacterium
996	2	3	14	0	0	0	13	1	14	0	0	0	k__Bacteria; p__Proteobacteria; c__Gammaproteobacteria; o__Oceanospirillales; f__Alcanivoracaceae; g__Alcanivorax;

													s__uncultured_gamma_proteobacterium
955	0	108	0	0	0	0	0	0	0	3	0	0	k__Bacteria; p__Firmicutes; c__Clostridia; o__Clostridiales; f__Ruminococcaceae; g__Anaerotruncus
914	0	12	0	0	0	0	0	0	0	0	0	0	k__Bacteria; p__Bacteroidetes; c__Flavobacteriia; o__Flavobacteriales; f__Flavobacteriaceae
67	334	0	0	0	24	0	0	0	0	8	37	29	k__Bacteria; p__Bacteroidetes; c__Bacteroidia; o__Bacteroidales; f__Rikenellaceae; g__Alistipes; Ambiguous_taxa
586	0	1	4	0	0	23	0	0	0	0	0	0	k__Bacteria; p__Bacteroidetes; c__Flavobacteriia; o__Flavobacteriales; f__Flavobacteriaceae; g__Ulvibacter; s__uncultured_bacterium
545	9	26	42	0	9	5	0	0	0	0	0	0	k__Bacteria; p__Bacteroidetes; c__Flavobacteriia; o__Flavobacteriales; f__Flavobacteriaceae; g__Maritimimonas; s__uncultured_Bacteroidetes_bacterium
504	0	0	0	0	12	4	0	0	0	0	0	0	k__Bacteria; p__Bacteroidetes; c__Cytophagia; o__Cytophagales; f__Flammeovirgaceae; g__Reichenbachiella
26	669	425	254	248	353	537	181	590	339	141	297	50	k__Bacteria; p__Bacteroidetes; c__Bacteroidia; o__Bacteroidales; f__Bacteroidales_S24_7_group
176	1	59	41	78	36	60	1	0	1	0	0	0	k__Bacteria; p__Proteobacteria; c__Gammaproteobacteria; o__Alteromonadales; f__Pseudoalteromonadaceae; g__Psychrosphaera; Ambiguous_taxa
135	392	1	48	136	223	187	30	99	41	200	171	23	k__Bacteria; p__Firmicutes; c__Clostridia; o__Clostridiales; f__Lachnospiraceae; g__Lachnospiraceae_NK4A136_group
997	15	24	0	1	0	0	0	0	0	0	0	0	k__Bacteria; p__Chlorobi; c__Ignavibacteria; o__Ignavibacteriales; f__IheB3_7; g__uncultured_bacterium;

													s__uncultured_bacterium
956	0	17	6	0	0	0	0	0	0	0	1	1	k__Bacteria; p__Firmicutes; c__Clostridia; o__Clostridiales; f__Clostridiaceae_1; g__uncultured
915	0	11	18	0	0	1	0	0	0	0	0	0	k__Bacteria; p__Bacteroidetes; c__Sphingobacteriia; o__Sphingobacteriales; f__Saprospiraceae; g__Portibacter; s__uncultured_Bacteroidetes_bacterium
68	0	0	0	63	5	0	1	2	0	25	6	0	k__Bacteria; p__Firmicutes; c__Clostridia; o__Clostridiales; f__Lachnospiraceae
587	0	0	0	0	0	25	0	0	0	77	4	0	k__Bacteria; p__Firmicutes; c__Clostridia; o__Clostridiales; f__Lachnospiraceae
546	0	0	0	0	1	79	1	0	0	4	5	0	k__Bacteria; p__Firmicutes; c__Clostridia; o__Clostridiales; f__Ruminococcaceae; g__Ruminococcaceae_UCG_013; s__uncultured_organism
505	0	0	0	0	36	0	0	0	0	4	3	0	k__Bacteria; p__Firmicutes; c__Clostridia; o__Clostridiales; f__Ruminococcaceae; g__Ruminococcaceae_UCG_014; s__uncultured_bacterium
27	0	0	0	0	77	95	3	116	55	795	7	2	k__Bacteria; p__Bacteroidetes; c__Bacteroidia; o__Bacteroidales; f__Prevotellaceae; g__Prevotella_1; s__uncultured_bacterium
177	86	0	0	10	5	54	0	50	29	36	19	1	k__Bacteria; p__Firmicutes; c__Clostridia; o__Clostridiales; f__Ruminococcaceae; g__uncultured
136	0	0	0	0	0	82	0	0	0	126	3	1	k__Bacteria; p__Firmicutes; c__Clostridia; o__Clostridiales; f__Ruminococcaceae; g__[Eubacterium]_coprostanoligenes_group; Ambiguous_taxa
998	0	21	0	0	0	0	0	0	0	0	0	0	k__Bacteria; p__Proteobacteria; c__Betaproteobacteria;

													o__Hydrogenophilales; f__Hydrogenophilaceae; g__uncultured; s__uncultured_beta_proteobacterium
957	7	34	18	3	0	7	0	0	0	0	0	0	k__Bacteria; p__Bacteroidetes; c__Flavobacteriia; o__Flavobacteriales; f__Flavobacteriaceae; g__Dokdonia; Ambiguous_taxa
916	21	70	103	1	0	0	8	7	22	0	0	0	k__Bacteria; p__Proteobacteria; c__Alphaproteobacteria; o__Rhodobacterales; f__Rhodobacteraceae
69	311	320	1	67	263	240	182	16	43	99	179	61	k__Bacteria; p__Bacteroidetes; c__Bacteroidia; o__Bacteroidales; f__Porphyromonadaceae; g__Odoribacter; s__unidentified
588	0	0	0	0	0	14	0	0	0	0	0	0	k__Bacteria; p__Bacteroidetes; c__Flavobacteriia; o__Flavobacteriales; f__Cryomorphaceae
547	0	0	4	6	1	30	0	0	0	0	0	0	k__Bacteria; p__Bacteroidetes; c__Sphingobacteriia; o__Sphingobacteriales; f__Saprospiraceae
506	0	0	0	0	39	0	0	0	0	0	0	0	k__Bacteria; p__Firmicutes; c__Clostridia; o__Clostridiales; f__Ruminococcaceae; g__Ruminococcaceae_UCG_014
28	456	0	103	337	336	317	248	88	162	131	234	39	k__Bacteria; p__Bacteroidetes; c__Bacteroidia; o__Bacteroidales; f__Rikenellaceae; g__Rikenellaceae_RC9_gut_group
178	0	178	0	79	46	0	0	0	0	22	72	13	k__Bacteria; p__Firmicutes; c__Clostridia; o__Clostridiales; f__Lachnospiraceae
137	21	18	77	47	32	77	0	0	0	0	0	0	k__Bacteria; p__Proteobacteria; c__Gammaproteobacteria; o__E01_9C_26_marine_group
999	11	19	24	0	1	0	1	1	2	0	0	0	k__Bacteria; p__Proteobacteria; c__Alphaproteobacteria; o__Rhodobacterales; f__Rhodobacteraceae

958	0	18	1	0	0	0	0	0	0	0	0	0	k__Bacteria; p__Bacteroidetes; c__Bacteroidetes_VC2.1_Bac22; o__uncultured_Bacteroidetes_bacterium; f__uncultured_Bacteroidetes_bacterium; g__uncultured_Bacteroidetes_bacterium; s__uncultured_Bacteroidetes_bacterium
917	0	1	5	0	0	0	0	0	0	0	0	0	k__Bacteria; p__Cloacimonetes; c__MSBL8; o__uncultured_bacterium; f__uncultured_bacterium; g__uncultured_bacterium; s__uncultured_bacterium
589	0	0	0	0	0	64	41	0	65	16	6	1	k__Bacteria; p__Bacteroidetes; c__Bacteroidia; o__Bacteroidales; f__Prevotellaceae; g__Alloprevotella; s__uncultured_bacterium
548	0	0	0	0	0	81	0	0	0	0	8	2	k__Bacteria; p__Firmicutes; c__Clostridia; o__Clostridiales; f__Ruminococcaceae; g__Ruminococcaceae_UCG_014; Ambiguous_taxa
507	0	50	1	0	27	1	49	0	0	9	77	40	k__Bacteria; p__Firmicutes; c__Clostridia; o__Clostridiales; f__Clostridiales_vadinBB60_group; g__uncultured_bacterium; s__uncultured_bacterium
29	39	55	156	112	82	130	115	171	120	25	3	0	k__Bacteria; p__Proteobacteria; c__Alphaproteobacteria; o__Sphingomonadales; f__Sphingomonadaceae; g__Sphingomonas
179	29	82	151	119	70	71	68	167	154	12	7	2	k__Bacteria; p__Firmicutes; c__Bacilli; o__Lactobacillales; f__Streptococcaceae; g__Streptococcus
138	230	2	0	43	31	30	0	97	0	33	128	35	k__Bacteria; p__Bacteroidetes; c__Bacteroidia; o__Bacteroidales; f__Bacteroidales_S24_7_group; g__uncultured_bacterium; s__uncultured_bacterium
959	0	4	12	0	0	0	1	1	15	0	0	0	k__Bacteria; p__Bacteroidetes; c__Bacteroidia;

													o__Bacteroidales; f__Marinilabiaceae; g__Carboxylicivirga
918	0	17	0	7	1	0	6	0	6	0	0	0	k__Bacteria; p__Bacteroidetes; c__Sphingobacteriia; o__Sphingobacteriales
549	1	1	45	1	0	131	43	53	34	134	64	8	k__Bacteria; p__Firmicutes; c__Clostridia; o__Clostridiales; f__Lachnospiraceae; g__Lachnospiraceae_NK4A136_group; s__uncultured_bacterium
508	0	0	47	0	40	0	0	0	0	5	5	0	k__Bacteria; p__Firmicutes; c__Clostridia; o__Clostridiales; f__Lachnospiraceae
139	330	186	42	86	156	288	246	137	182	162	259	32	k__Bacteria; p__Bacteroidetes; c__Bacteroidia; o__Bacteroidales; f__Bacteroidales_S24_7_group
919	0	130	0	0	0	0	0	0	0	0	3	0	k__Bacteria; p__Bacteroidetes; c__Bacteroidia; o__Bacteroidales; f__Prevotellaceae
509	132	0	68	0	77	1	60	0	0	35	48	34	k__Bacteria; p__Firmicutes; c__Clostridia; o__Clostridiales; f__Lachnospiraceae; g__uncultured; s__uncultured_bacterium
1290	5	0	2	0	0	6	2	57	79	15	6	1	k__Bacteria; p__Firmicutes; c__Clostridia; o__Clostridiales; f__Lachnospiraceae; g__Lachnospiraceae_NK4A136_group; s__uncultured_bacterium
1660	0	0	0	0	0	0	3	0	0	0	0	0	k__Bacteria; p__Bacteroidetes; c__Bacteroidetes_BD2_2
1291	0	0	0	0	0	0	0	3	4	9	2	3	k__Bacteria; p__Firmicutes; c__Clostridia; o__Clostridiales; f__Clostridiaceae_1
1250	0	0	0	0	0	0	0	0	0	0	0	2	k__Bacteria; p__Firmicutes; c__Clostridia; o__Clostridiales; f__Ruminococcaceae; g__Ruminiclostridium_5
1661	0	0	0	0	0	0	0	13	3	0	0	0	k__Bacteria; p__Bacteroidetes; c__Flavobacteriia; o__Flavobacteriales; f__Cryomorphaceae; g__Crocinitomix; s__uncultured_bacterium

1620	11	0	0	1	4	0	3	0	3	0	0	0	k__Bacteria; p__Proteobacteria; c__Gammaproteobacteria; o__Cellvibrionales; f__Halieaceae; g__Luminiphilus; s__uncultured_gamma_proteobacterium
1292	0	0	0	0	0	0	0	2	0	3	0	0	k__Bacteria; p__Firmicutes; c__Clostridia; o__Clostridiales; f__Clostridiaceae_1; g__Oceanirhabdus; Ambiguous_taxa
1251	0	0	0	0	0	0	0	0	0	0	9	2	k__Bacteria; p__Firmicutes; c__Clostridia; o__Clostridiales; f__Lachnospiraceae
1210	0	0	6	2	0	0	0	0	0	0	0	0	k__Bacteria; p__Acidobacteria; c__Holophagae; o__Subgroup_10; f__CA002; g__uncultured_bacterium; s__uncultured_bacterium
1662	5	5	8	0	0	0	0	8	1	0	0	0	k__Bacteria; p__Proteobacteria; c__Gammaproteobacteria; o__Order_Incertae_Sedis; f__Family_Incertae_Sedis; g__Marinicella; s__uncultured_bacterium
1621	0	0	0	0	0	0	2	3	0	0	0	0	k__Bacteria; p__Proteobacteria; c__Gammaproteobacteria
1293	0	7	4	1	0	0	17	8	34	24	2	4	k__Bacteria; p__Bacteroidetes; c__Bacteroidia; o__Bacteroidales; f__Marinilabiaceae; g__Carboxylicivirga; s__uncultured_bacterium
1252	0	0	0	0	0	0	1	0	0	72	0	0	k__Bacteria; p__Firmicutes; c__Clostridia; o__Clostridiales; f__Ruminococcaceae
1211	1	3	10	1	0	0	0	0	0	0	0	0	k__Bacteria; p__Bacteroidetes; c__Sphingobacteriia; o__Sphingobacteriales; f__Saprospiraceae
1663	0	0	0	0	0	0	9	4	0	0	0	0	k__Bacteria; p__Fibrobacteres; c__Fibrobacteria; o__Fibrobacteria_Incertae_Sedis; f__Unknown_Family; g__possible_genus_03; s__uncultured_bacterium
1622	0	0	3	0	0	0	8	4	12	0	0	0	k__Bacteria; p__Proteobacteria; c__Epsilonproteobacteria; o__Campylobacterales; f__Helicobacteraceae; g__Sulfurimonas

1294	0	0	0	1	0	0	16	16	43	2	0	1	k__Bacteria; p__Proteobacteria; c__Gammaproteobacteria; o__Vibrionales; f__Vibrionaceae; g__Photobacterium
1253	0	0	0	0	0	0	0	0	0	33	0	2	k__Bacteria; p__Actinobacteria; c__Coriobacteriia; o__Coriobacteriales; f__Coriobacteriaceae; g__Coriobacteriaceae_UCG_002; s__uncultured_bacterium
1212	0	0	3	0	0	0	0	0	0	0	0	0	k__Bacteria; p__Proteobacteria; c__Gammaproteobacteria; o__Order_Incertae_Sedis; f__Family_Incertae_Sedis; g__Marinicella
1664	0	0	0	0	0	0	0	2	0	0	0	0	k__Bacteria; p__Bacteroidetes; c__Cytophagia; o__Cytophagales; f__Flammeovirgaceae; g__Marinoscillum
1623	0	3	12	0	0	0	4	1	4	0	0	0	k__Bacteria; p__Bacteroidetes; c__Bacteroidetes_VC2.1_Bac22
1295	3	3	6	0	0	0	16	9	8	3	0	0	k__Bacteria; p__Bacteroidetes; c__Cytophagia; o__Cytophagales; f__Flammeovirgaceae; g__Flammeovirga; Ambiguous_taxa
1254	0	0	0	0	0	0	0	0	0	23	15	31	k__Bacteria; p__Firmicutes; c__Clostridia; o__Clostridiales; f__Lachnospiraceae; g__uncultured
1213	0	0	1	0	1	0	0	0	0	0	1	0	k__Bacteria; p__Actinobacteria; c__Actinobacteria; o__Bifidobacteriales; f__Bifidobacteriaceae; g__Bifidobacterium
1665	0	0	0	0	0	0	0	5	3	0	0	0	k__Bacteria; p__Fibrobacteres; c__Fibrobacteria; o__Fibrobacteria_Incertae_Sedis; f__Unknown_Family; g__possible_genus_03; s__uncultured_bacterium
1624	0	0	0	0	1	1	1	0	0	4	0	0	k__Bacteria; p__Firmicutes; c__Erysipelotrichia; o__Erysipelotrichales; f__Erysipelotrichaceae; g__Allobaculum; Ambiguous_taxa

1296	0	0	0	0	0	0	21	13	18	7	0	1	k__Bacteria; p__Bacteroidetes; c__Bacteroidia; o__Bacteroidales; f__Marinilabiaceae; g__Mangroviflexus; Ambiguous_taxa
1255	0	0	0	0	0	0	0	1	0	0	2	0	k__Bacteria; p__Firmicutes; c__Clostridia; o__Clostridiales; f__Ruminococcaceae; g__Anaerotruncus; s__uncultured_organism
1214	0	0	4	0	0	0	0	0	0	0	0	0	k__Bacteria; p__Latescibacteria
1666	0	0	0	0	0	0	0	0	1	0	0	0	k__Bacteria
1625	0	1	0	0	0	0	19	20	16	0	0	0	k__Bacteria; p__Tenericutes; c__Mollicutes; o__Mycoplasmatales; f__Mycoplasmataceae; g__Mycoplasma
1256	0	0	0	0	0	0	1	0	0	1	8	2	k__Bacteria; p__Firmicutes; c__Clostridia; o__Clostridiales; f__Lachnospiraceae; g__uncultured; Ambiguous_taxa
1215	0	0	4	0	1	0	0	0	0	0	0	0	k__Bacteria; p__Actinobacteria; c__Actinobacteria; o__Micrococcales; f__Microbacteriaceae
1667	0	0	0	0	0	0	1	0	0	0	0	0	k__Bacteria; p__Proteobacteria; c__Deltaproteobacteria; o__Desulfobacterales; f__Desulfobulbaceae
1626	0	0	0	0	0	0	3	14	4	7	0	0	k__Bacteria; p__Chlorobi; c__Chlorobia; o__Chlorobiales; f__OPB56; g__uncultured_bacterium; s__uncultured_bacterium
1298	0	0	0	0	0	0	0	0	1	60	0	0	k__Bacteria; p__Bacteroidetes; c__Bacteroidia; o__Bacteroidales; f__Bacteroidales_S24_7_group; g__uncultured_bacterium; s__uncultured_bacterium
1257	0	0	0	0	0	0	0	0	1	0	3	0	k__Bacteria; p__Firmicutes; c__Clostridia; o__Clostridiales; f__Ruminococcaceae; g__Ruminococcaceae_UCG_014
1216	0	1	9	0	0	0	10	5	2	0	0	0	k__Bacteria; p__Bacteroidetes; c__Cytophagia; o__Cytophagales; f__Flammeovirgaceae; g__Ekhidna; Ambiguous_taxa

1668	0	0	0	0	0	0	0	0	0	0	1	0	k__Bacteria; p__Firmicutes; c__Erysipelotrichia; o__Erysipelotrichales; f__Erysipelotrichaceae; g__Solobacterium; Ambiguous_taxa
1627	0	0	0	0	0	0	0	7	4	0	0	0	k__Bacteria; p__Bacteroidetes; c__Bacteroidia; o__Bacteroidales; f__Marinilabiaceae; g__Carboxylicivirga
1299	0	0	0	2	1	0	0	1	0	10	3	3	k__Bacteria; p__Firmicutes; c__Clostridia; o__Clostridiales; f__Family_XII; g__Fusibacter
1258	0	0	0	0	0	0	1	0	0	3	0	0	k__Bacteria; p__Firmicutes; c__Clostridia; o__Clostridiales; f__Lachnospiraceae
1217	0	0	3	0	0	0	0	0	1	0	0	0	k__Bacteria; p__Proteobacteria; c__Gammaproteobacteria; o__Oceanospirillales; f__Oceanospirillaceae; g__Marinomonas
1669	0	0	0	0	0	0	0	1	0	0	0	0	k__Bacteria; p__Spirochaetae; c__Spirochaetes; o__Spirochaetales; f__Spirochaetaceae; g__Spirochaeta_2; s__uncultured_Spirochaeta_sp.
1628	0	0	0	0	0	0	0	9	5	0	0	0	k__Bacteria; p__Proteobacteria; c__Gammaproteobacteria; o__Alteromonadales; f__Alteromonadaceae
1259	0	0	0	0	0	0	0	0	0	0	2	4	k__Bacteria; p__Proteobacteria; c__Deltaproteobacteria; o__Desulfovibrionales; f__Desulfovibrionaceae; g__Desulfovibrio
1218	0	3	14	0	0	0	0	0	0	0	0	0	k__Bacteria; p__Actinobacteria; c__Acidimicrobiia; o__Acidimicrobiales; f__OM1_clade; g__uncultured_actinobacterium; s__uncultured_actinobacterium
1219	1	0	4	0	0	0	0	0	0	0	0	0	k__Bacteria; p__Proteobacteria; c__Gammaproteobacteria; o__Xanthomonadales; f__JTB255_marine_benthic_group; g__uncultured_bacterium; s__uncultured_bacterium

OTU_62	1058	923	723	874	k__Bacteria; p__Bacteroidetes; c__Bacteroidia; o__Bacteroidales; f__Bacteroidales_S24_7_group; g__uncultured_bacterium; s__uncultured_bacterium
OTU_106	608	598	310	394	k__Bacteria; p__Bacteroidetes; c__Bacteroidia; o__Bacteroidales; f__Bacteroidales_S24_7_group; g__uncultured_bacterium; s__uncultured_bacterium
OTU_190	416	564	389	177	k__Bacteria; p__Bacteroidetes; c__Bacteroidia; o__Bacteroidales; f__Bacteroidales_S24_7_group; g__uncultured_bacterium; s__uncultured_bacterium
OTU_20	1976	1710	1380	1463	k__Bacteria; p__Bacteroidetes; c__Bacteroidia; o__Bacteroidales; f__Bacteroidales_S24_7_group; g__uncultured_Bacteroidales_bacterium; s__uncultured_Bacteroidales_bacterium
OTU_39	1960	2134	1284	1832	k__Bacteria; p__Bacteroidetes; c__Bacteroidia; o__Bacteroidales; f__Bacteroidales_S24_7_group; g__uncultured_Bacteroidales_bacterium; s__uncultured_Bacteroidales_bacterium
OTU_97	1038	721	749	775	k__Bacteria; p__Bacteroidetes; c__Bacteroidia; o__Bacteroidales; f__Bacteroidales_S24_7_group; g__uncultured_Bacteroidales_bacterium; s__uncultured_Bacteroidales_bacterium
OTU_122	584	1173	732	618	k__Bacteria; p__Bacteroidetes; c__Bacteroidia; o__Bacteroidales; f__Bacteroidales_S24_7_group; g__uncultured_Bacteroidales_bacterium; s__uncultured_Bacteroidales_bacterium
OTU_69	632	570	241	339	k__Bacteria; p__Bacteroidetes; c__Bacteroidia; o__Bacteroidales; f__Porphyromonadaceae; g__Odoribacter; s__unidentified
OTU_46	2258	3135	2086	2552	k__Bacteria; p__Bacteroidetes; c__Bacteroidia; o__Bacteroidales; f__Prevotellaceae; g__Alloprevotella; s__uncultured_bacterium
OTU_25	1827	845	1168	246	k__Bacteria; p__Bacteroidetes; c__Bacteroidia; o__Bacteroidales; f__Prevotellaceae; g__Prevotella_1
OTU_86	1076	219	222	249	k__Bacteria; p__Bacteroidetes; c__Bacteroidia; o__Bacteroidales; f__Prevotellaceae; g__Prevotella_1; s__uncultured_bacterium
OTU_5	411	319	221	105	k__Bacteria; p__Bacteroidetes; c__Bacteroidia; o__Bacteroidales; f__Prevotellaceae; g__Prevotella_2; s__uncultured_bacterium
OTU_131	512	679	757	176	k__Bacteria; p__Bacteroidetes; c__Bacteroidia; o__Bacteroidales; f__Prevotellaceae; g__Prevotella_7; s__uncultured_bacterium

OTU_387	524	487	539	605	k__Bacteria; p__Bacteroidetes; c__Bacteroidia; o__Bacteroidales; f__Prevotellaceae; g__Prevotella_9
OTU_6	745	528	455	5256	k__Bacteria; p__Bacteroidetes; c__Bacteroidia; o__Bacteroidales; f__Prevotellaceae; g__Prevotella_9; s__uncultured_bacterium
OTU_35	2543	3064	1602	1852	k__Bacteria; p__Bacteroidetes; c__Bacteroidia; o__Bacteroidales; f__Prevotellaceae; g__Prevotella_9; s__uncultured_bacterium
OTU_82	461	228	348	111	k__Bacteria; p__Bacteroidetes; c__Bacteroidia; o__Bacteroidales; f__Prevotellaceae; g__Prevotellaceae_NK3B31_group; s__uncultured_bacterium
OTU_119	1237	763	446	1137	k__Bacteria; p__Bacteroidetes; c__Bacteroidia; o__Bacteroidales; f__Prevotellaceae; g__Prevotellaceae_UCG_001
OTU_633	431	354	387	853	k__Bacteria; p__Bacteroidetes; c__Bacteroidia; o__Bacteroidales; f__Rikenellaceae; g__Alistipes
OTU_181	343	625	158	494	k__Bacteria; p__Bacteroidetes; c__Bacteroidia; o__Bacteroidales; f__Rikenellaceae; g__Alistipes; Ambiguous_taxa
OTU_168	239	249	237	233	k__Bacteria; p__Bacteroidetes; c__Bacteroidia; o__Bacteroidales; f__Rikenellaceae; g__Rikenella; Ambiguous_taxa
OTU_222	55	28	28	307	k__Bacteria; p__Bacteroidetes; c__Bacteroidia; o__Bacteroidia_Incertae_Sedis; f__Prolixibacteraceae; g__Prolixibacter; Ambiguous_taxa
OTU_313	219	276	429	162	k__Bacteria; p__Deferribacteres; c__Deferribacteres; o__Deferribacterales; f__Deferribacteraceae; g__Mucispirillum; Ambiguous_taxa
OTU_4	5385	6709	7972	464	k__Bacteria; p__Firmicutes; c__Bacilli; o__Bacillales; f__Bacillaceae; g__Bacillus; s__Bacillus_cereus
OTU_51	1578	1433	1069	728	k__Bacteria; p__Firmicutes; c__Bacilli; o__Bacillales; f__Staphylococcaceae; g__Staphylococcus; Ambiguous_taxa
OTU_83	1816	2982	2297	2235	k__Bacteria; p__Firmicutes; c__Bacilli; o__Bacillales; f__Staphylococcaceae; g__Staphylococcus; s__Staphylococcus_sp._SV3
OTU_21	7251	8826	4590	6354	k__Bacteria; p__Firmicutes; c__Bacilli; o__Lactobacillales
OTU_218	142	233	424	13	k__Bacteria; p__Firmicutes; c__Bacilli; o__Lactobacillales; f__Carnobacteriaceae; g__Carnobacterium; Ambiguous_taxa
OTU_133	561	583	495	169	k__Bacteria; p__Firmicutes; c__Bacilli; o__Lactobacillales; f__Enterococcaceae; g__Enterococcus
OTU_40	1429	2195	1359	1249	k__Bacteria; p__Firmicutes; c__Bacilli; o__Lactobacillales; f__Lactobacillaceae; g__Lactobacillus; s__Lactobacillus_johnsonii
OTU_9	957	2102	1637	1082	k__Bacteria; p__Firmicutes; c__Bacilli; o__Lactobacillales; f__Lactobacillaceae; g__Lactobacillus; s__Lactobacillus_vaginalis

OTU_55	2212	2680	4001	187	k__Bacteria; p__Firmicutes; c__Bacilli; o__Lactobacillales; f__Streptococcaceae; g__Lactococcus
OTU_58	230	294	478	37	k__Bacteria; p__Firmicutes; c__Bacilli; o__Lactobacillales; f__Streptococcaceae; g__Lactococcus; s__Lactococcus_lactis
OTU_179	262	260	389	21	k__Bacteria; p__Firmicutes; c__Bacilli; o__Lactobacillales; f__Streptococcaceae; g__Streptococcus
OTU_111	96	79	180	18	k__Bacteria; p__Firmicutes; c__Bacilli; o__Lactobacillales; f__Streptococcaceae; g__Streptococcus; s__Streptococcus_salivarius_subsp._thermophilus
OTU_3	993	670	361	435	k__Bacteria; p__Firmicutes; c__Clostridia; o__Clostridiales; f__Clostridiales_vadinBB60_group; Ambiguous_taxa; Ambiguous_taxa
OTU_135	441	546	170	394	k__Bacteria; p__Firmicutes; c__Clostridia; o__Clostridiales; f__Lachnospiraceae; g__Lachnospiraceae_NK4A136_group
OTU_372	346	206	242	1031	k__Bacteria; p__Firmicutes; c__Clostridia; o__Clostridiales; f__Lachnospiraceae; g__Lachnospiraceae_NK4A136_group
OTU_8	568	702	299	372	k__Bacteria; p__Firmicutes; c__Clostridia; o__Clostridiales; f__Lachnospiraceae; g__Lachnospiraceae_NK4A136_group; s__uncultured_bacterium
OTU_70	525	660	276	309	k__Bacteria; p__Firmicutes; c__Clostridia; o__Clostridiales; f__Lachnospiraceae; g__Lachnospiraceae_NK4A136_group; s__uncultured_bacterium
OTU_88	958	846	560	3281	k__Bacteria; p__Firmicutes; c__Clostridia; o__Clostridiales; f__Lachnospiraceae; g__Lachnospiraceae_NK4A136_group; s__uncultured_bacterium
OTU_625	971	1005	634	2386	k__Bacteria; p__Firmicutes; c__Clostridia; o__Clostridiales; f__Lachnospiraceae; g__Lachnospiraceae_NK4A136_group; s__uncultured_bacterium
OTU_237	1007	333	195	195	k__Bacteria; p__Firmicutes; c__Clostridia; o__Clostridiales; f__Lachnospiraceae; g__Lachnospiraceae_UCG_001; s__uncultured_bacterium
OTU_79	665	466	374	1748	k__Bacteria; p__Firmicutes; c__Clostridia; o__Clostridiales; f__Lachnospiraceae; g__Pseudobutyrivibrio; s__uncultured_bacterium
OTU_37	270	126	338	161	k__Bacteria; p__Firmicutes; c__Clostridia; o__Clostridiales; f__Lachnospiraceae; g__uncultured; s__uncultured_bacterium
OTU_11	292	347	298	152	k__Bacteria; p__Firmicutes; c__Clostridia; o__Clostridiales; f__Ruminococcaceae; g__Ruminococcus_1
OTU_2	260	127	75	41	k__Bacteria; p__Firmicutes; c__Negativicutes; o__Selenomonadales; f__Veillonellaceae; g__Anaerovibrio; s__uncultured_bacterium

OTU_296	3582	42	92	126	k__Bacteria; p__Proteobacteria; c__Epsilonproteobacteria; o__Campylobacterales; f__Campylobacteraceae; g__Arcobacter; Ambiguous_taxa
OTU_12	1096	1085	969	1083	k__Bacteria; p__Proteobacteria; c__Epsilonproteobacteria; o__Campylobacterales; f__Helicobacteraceae; g__Helicobacter; Ambiguous_taxa
OTU_464	1220	46	343	25	k__Bacteria; p__Proteobacteria; c__Gammaproteobacteria; o__Alteromonadales; f__Alteromonadaceae; g__uncultured; s__uncultured_bacterium
OTU_166	314	212	33	13	k__Bacteria; p__Proteobacteria; c__Gammaproteobacteria; o__Alteromonadales; f__Pseudoalteromonadaceae; g__Pseudoalteromonas
OTU_215	223	19	265	181	k__Bacteria; p__Proteobacteria; c__Gammaproteobacteria; o__Vibrionales; f__Vibrionaceae; g__Vibrio
OTU_553	326	11	24	20	k__Bacteria; p__Proteobacteria; c__Gammaproteobacteria; o__Vibrionales; f__Vibrionaceae; g__Vibrio
