# Supplementary material for: Ubiquitous flocculation activity and flocculation production basis of the conglutination mud from Ruditapes philippinarum along the coast of China
Source: PLoS One. 2021 Nov 18;16(11):e0256013. doi: 10.1371/journal.pone.0256013 (PMC8601509; doi:10.1371/journal.pone.0256013)
Supplement: S2 Table — WH, DL, ZS and ZJ represent bacterial communities from the Weihai, Dalian, Zhoushan and Zhanjiang RPMs respectively. The values are presented as mean ± SD (n = 3). (PDF) [file pone.0256013.s004.pdf]

S2 Table. Richness and diversity estimation of the 16S rRNA sequencing libraries from the MiSeq sequencing analysis. WH, DL, ZS and ZJ represent bacterial communities from the Weihai, Dalian, Zhoushan and Zhanjiang RPMs respectively. The values are presented as mean ± SD (n = 3).

Sample	Observed OTUs	Chao1	Shannon Wiener	Gini-Simpson	Coverage
WH	571±178	648±198	7.45±0.31	0.987±0.001	0.993±0.002
DL	439±24	530±52	7.07±0.11	0.982±0.004	0.994±0.001
ZS	546±39	640±12	7.30±0.22	0.983±0.004	0.993±0.000
