# Supplementary material for: Ubiquitous flocculation activity and flocculation production basis of the conglutination mud from Ruditapes philippinarum along the coast of China
Source: PLoS One. 2021 Nov 18;16(11):e0256013. doi: 10.1371/journal.pone.0256013 (PMC8601509; doi:10.1371/journal.pone.0256013)
Supplement: S3 Table — (PDF) [file pone.0256013.s005.pdf]

S3 Table. The taxonomical classification and abundance of all overlapping OTUs from RPMs of four Chinese coastal locations.

OTU_ID	WH	DL	ZS	ZJ	taxonomy
OTU_52	1367	925	897	1092	k__Bacteria; p__Bacteroidetes; c__Bacteroidia; o__Bacteroidales; f__Bacteroidaceae; g__Bacteroides
OTU_140	712	894	337	446	k__Bacteria; p__Bacteroidetes; c__Bacteroidia; o__Bacteroidales; f__Bacteroidaceae; g__Bacteroides
OTU_26	1348	1138	1110	488	k__Bacteria; p__Bacteroidetes; c__Bacteroidia; o__Bacteroidales; f__Bacteroidales_S24_7_group
OTU_59	1631	857	310	492	k__Bacteria; p__Bacteroidetes; c__Bacteroidia; o__Bacteroidales; f__Bacteroidales_S24_7_group
OTU_139	558	530	565	453	k__Bacteria; p__Bacteroidetes; c__Bacteroidia; o__Bacteroidales; f__Bacteroidales_S24_7_group
OTU_49	1793	1992	1197	913	k__Bacteria; p__Bacteroidetes; c__Bacteroidia; o__Bacteroidales; f__Bacteroidales_S24_7_group; Ambiguous_taxa; Ambiguous_taxa
OTU_155	233	425	375	805	k__Bacteria; p__Bacteroidetes; c__Bacteroidia; o__Bacteroidales; f__Bacteroidales_S24_7_group; Ambiguous_taxa; Ambiguous_taxa
OTU_19	730	363	382	234	k__Bacteria; p__Bacteroidetes; c__Bacteroidia; o__Bacteroidales; f__Bacteroidales_S24_7_group; g__uncultured_bacterium; s__uncultured_bacterium
OTU_31	415	446	395	200	k__Bacteria; p__Bacteroidetes; c__Bacteroidia; o__Bacteroidales; f__Bacteroidales_S24_7_group; g__uncultured_bacterium; s__uncultured_bacterium
OTU_62	1058	923	723	874	k__Bacteria; p__Bacteroidetes; c__Bacteroidia; o__Bacteroidales; f__Bacteroidales_S24_7_group; g__uncultured_bacterium; s__uncultured_bacterium
OTU_106	608	598	310	394	k__Bacteria; p__Bacteroidetes; c__Bacteroidia; o__Bacteroidales; f__Bacteroidales_S24_7_group; g__uncultured_bacterium; s__uncultured_bacterium
OTU_190	416	564	389	177	k__Bacteria; p__Bacteroidetes; c__Bacteroidia; o__Bacteroidales; f__Bacteroidales_S24_7_group; g__uncultured_bacterium; s__uncultured_bacterium
OTU_20	1976	1710	1380	1463	k__Bacteria; p__Bacteroidetes; c__Bacteroidia; o__Bacteroidales; f__Bacteroidales_S24_7_group; g__uncultured_Bacteroidales_bacterium; s__uncultured_Bacteroidales_bacterium
OTU_39	1960	2134	1284	1832	k__Bacteria; p__Bacteroidetes; c__Bacteroidia; o__Bacteroidales; f__Bacteroidales_S24_7_group; g__uncultured_Bacteroidales_bacterium; s__uncultured_Bacteroidales_bacterium

OTU_97	1038	721	749	775	k__Bacteria; p__Bacteroidetes; c__Bacteroidia; o__Bacteroidales; f__Bacteroidales_S24_7_group; g__uncultured_Bacteroidales_bacterium; s__uncultured_Bacteroidales_bacterium
OTU_122	584	1173	732	618	k__Bacteria; p__Bacteroidetes; c__Bacteroidia; o__Bacteroidales; f__Bacteroidales_S24_7_group; g__uncultured_Bacteroidales_bacterium; s__uncultured_Bacteroidales_bacterium
OTU_69	632	570	241	339	k__Bacteria; p__Bacteroidetes; c__Bacteroidia; o__Bacteroidales; f__Porphyromonadaceae; g__Odoribacter; s__unidentified
OTU_46	2258	3135	2086	2552	k__Bacteria; p__Bacteroidetes; c__Bacteroidia; o__Bacteroidales; f__Prevotellaceae; g__Alloprevotella; s__uncultured_bacterium
OTU_25	1827	845	1168	246	k__Bacteria; p__Bacteroidetes; c__Bacteroidia; o__Bacteroidales; f__Prevotellaceae; g__Prevotella_1
OTU_86	1076	219	222	249	k__Bacteria; p__Bacteroidetes; c__Bacteroidia; o__Bacteroidales; f__Prevotellaceae; g__Prevotella_1; s__uncultured_bacterium
OTU_5	411	319	221	105	k__Bacteria; p__Bacteroidetes; c__Bacteroidia; o__Bacteroidales; f__Prevotellaceae; g__Prevotella_2; s__uncultured_bacterium
OTU_131	512	679	757	176	k__Bacteria; p__Bacteroidetes; c__Bacteroidia; o__Bacteroidales; f__Prevotellaceae; g__Prevotella_7; s__uncultured_bacterium
OTU_387	524	487	539	605	k__Bacteria; p__Bacteroidetes; c__Bacteroidia; o__Bacteroidales; f__Prevotellaceae; g__Prevotella_9
OTU_6	745	528	455	5256	k__Bacteria; p__Bacteroidetes; c__Bacteroidia; o__Bacteroidales; f__Prevotellaceae; g__Prevotella_9; s__uncultured_bacterium
OTU_35	2543	3064	1602	1852	k__Bacteria; p__Bacteroidetes; c__Bacteroidia; o__Bacteroidales; f__Prevotellaceae; g__Prevotella_9; s__uncultured_bacterium
OTU_82	461	228	348	111	k__Bacteria; p__Bacteroidetes; c__Bacteroidia; o__Bacteroidales; f__Prevotellaceae; g__Prevotellaceae_NK3B31_group; s__uncultured_bacterium
OTU_119	1237	763	446	1137	k__Bacteria; p__Bacteroidetes; c__Bacteroidia; o__Bacteroidales; f__Prevotellaceae; g__Prevotellaceae_UCG_001
OTU_633	431	354	387	853	k__Bacteria; p__Bacteroidetes; c__Bacteroidia; o__Bacteroidales; f__Rikenellaceae; g__Alistipes
OTU_181	343	625	158	494	k__Bacteria; p__Bacteroidetes; c__Bacteroidia; o__Bacteroidales; f__Rikenellaceae; g__Alistipes; Ambiguous_taxa
OTU_168	239	249	237	233	k__Bacteria; p__Bacteroidetes; c__Bacteroidia; o__Bacteroidales; f__Rikenellaceae; g__Rikenella; Ambiguous_taxa

OTU_222	55	28	28	307	k__Bacteria; p__Bacteroidetes; c__Bacteroidia; o__Bacteroidia_Incertae_Sedis; f__Prolixibacteraceae; g__Prolixibacter; Ambiguous_taxa
OTU_313	219	276	429	162	k__Bacteria; p__Deferribacteres; c__Deferribacteres; o__Deferribacterales; f__Deferribacteraceae; g__Mucispirillum; Ambiguous_taxa
OTU_4	5385	6709	7972	464	k__Bacteria; p__Firmicutes; c__Bacilli; o__Bacillales; f__Bacillaceae; g__Bacillus; s__Bacillus_cereus
OTU_51	1578	1433	1069	728	k__Bacteria; p__Firmicutes; c__Bacilli; o__Bacillales; f__Staphylococcaceae; g__Staphylococcus; Ambiguous_taxa
OTU_83	1816	2982	2297	2235	k__Bacteria; p__Firmicutes; c__Bacilli; o__Bacillales; f__Staphylococcaceae; g__Staphylococcus; s__Staphylococcus_sp._SV3
OTU_21	7251	8826	4590	6354	k__Bacteria; p__Firmicutes; c__Bacilli; o__Lactobacillales
OTU_218	142	233	424	13	k__Bacteria; p__Firmicutes; c__Bacilli; o__Lactobacillales; f__Carnobacteriaceae; g__Carnobacterium; Ambiguous_taxa
OTU_133	561	583	495	169	k__Bacteria; p__Firmicutes; c__Bacilli; o__Lactobacillales; f__Enterococcaceae; g__Enterococcus
OTU_40	1429	2195	1359	1249	k__Bacteria; p__Firmicutes; c__Bacilli; o__Lactobacillales; f__Lactobacillaceae; g__Lactobacillus; s__Lactobacillus_johnsonii
OTU_9	957	2102	1637	1082	k__Bacteria; p__Firmicutes; c__Bacilli; o__Lactobacillales; f__Lactobacillaceae; g__Lactobacillus; s__Lactobacillus_vaginalis
OTU_55	2212	2680	4001	187	k__Bacteria; p__Firmicutes; c__Bacilli; o__Lactobacillales; f__Streptococcaceae; g__Lactococcus
OTU_58	230	294	478	37	k__Bacteria; p__Firmicutes; c__Bacilli; o__Lactobacillales; f__Streptococcaceae; g__Lactococcus; s__Lactococcus_lactis
OTU_179	262	260	389	21	k__Bacteria; p__Firmicutes; c__Bacilli; o__Lactobacillales; f__Streptococcaceae; g__Streptococcus
OTU_111	96	79	180	18	k__Bacteria; p__Firmicutes; c__Bacilli; o__Lactobacillales; f__Streptococcaceae; g__Streptococcus; s__Streptococcus_salivarius_subsp._thermophilus
OTU_3	993	670	361	435	k__Bacteria; p__Firmicutes; c__Clostridia; o__Clostridiales; f__Clostridiales_vadinBB60_group; Ambiguous_taxa; Ambiguous_taxa
OTU_135	441	546	170	394	k__Bacteria; p__Firmicutes; c__Clostridia; o__Clostridiales; f__Lachnospiraceae; g__Lachnospiraceae_NK4A136_group
OTU_372	346	206	242	1031	k__Bacteria; p__Firmicutes; c__Clostridia; o__Clostridiales; f__Lachnospiraceae; g__Lachnospiraceae_NK4A136_group
OTU_8	568	702	299	372	k__Bacteria; p__Firmicutes; c__Clostridia; o__Clostridiales; f__Lachnospiraceae; g__Lachnospiraceae_NK4A136_group; s__uncultured_bacterium

OTU_70	525	660	276	309	k__Bacteria; p__Firmicutes; c__Clostridia; o__Clostridiales; f__Lachnospiraceae; g__Lachnospiraceae_NK4A136_group; s__uncultured_bacterium
OTU_88	958	846	560	3281	k__Bacteria; p__Firmicutes; c__Clostridia; o__Clostridiales; f__Lachnospiraceae; g__Lachnospiraceae_NK4A136_group; s__uncultured_bacterium
OTU_625	971	1005	634	2386	k__Bacteria; p__Firmicutes; c__Clostridia; o__Clostridiales; f__Lachnospiraceae; g__Lachnospiraceae_NK4A136_group; s__uncultured_bacterium
OTU_237	1007	333	195	195	k__Bacteria; p__Firmicutes; c__Clostridia; o__Clostridiales; f__Lachnospiraceae; g__Lachnospiraceae_UCG_001; s__uncultured_bacterium
OTU_79	665	466	374	1748	k__Bacteria; p__Firmicutes; c__Clostridia; o__Clostridiales; f__Lachnospiraceae; g__Pseudobutyrivibrio; s__uncultured_bacterium
OTU_37	270	126	338	161	k__Bacteria; p__Firmicutes; c__Clostridia; o__Clostridiales; f__Lachnospiraceae; g__uncultured; s__uncultured_bacterium
OTU_11	292	347	298	152	k__Bacteria; p__Firmicutes; c__Clostridia; o__Clostridiales; f__Ruminococcaceae; g__Ruminococcus_1
OTU_2	260	127	75	41	k__Bacteria; p__Firmicutes; c__Negativicutes; o__Selenomonadales; f__Veillonellaceae; g__Anaerovibrio; s__uncultured_bacterium
OTU_296	3582	42	92	126	k__Bacteria; p__Proteobacteria; c__Epsilonproteobacteria; o__Campylobacterales; f__Campylobacteraceae; g__Arcobacter; Ambiguous_taxa
OTU_12	1096	1085	969	1083	k__Bacteria; p__Proteobacteria; c__Epsilonproteobacteria; o__Campylobacterales; f__Helicobacteraceae; g__Helicobacter; Ambiguous_taxa
OTU_464	1220	46	343	25	k__Bacteria; p__Proteobacteria; c__Gammaproteobacteria; o__Alteromonadales; f__Alteromonadaceae; g__uncultured; s__uncultured_bacterium
OTU_166	314	212	33	13	k__Bacteria; p__Proteobacteria; c__Gammaproteobacteria; o__Alteromonadales; f__Pseudoalteromonadaceae; g__Pseudoalteromonas
OTU_215	223	19	265	181	k__Bacteria; p__Proteobacteria; c__Gammaproteobacteria; o__Vibrionales; f__Vibrionaceae; g__Vibrio
OTU_553	326	11	24	20	k__Bacteria; p__Proteobacteria; c__Gammaproteobacteria; o__Vibrionales; f__Vibrionaceae; g__Vibrio
